# Multiparameter Optimization of *Pseudomonas aeruginosa* Elastase Inhibitors for Systemic Administration

**DOI:** 10.1021/acs.jmedchem.5c02788

**Published:** 2026-02-05

**Authors:** Ahmed S. Abdelsamie, Jelena Konstantinović, Andreas M. Kany, Christian Schütz, Dominik Kolling, Samira Speicher, Andreas Klein, Roya Shafiei, Mélodie Bouté, Katharina Mundry, Yu Mi Park, Brigitta Loretz, Rolf Müller, Jean-Michel Sallenave, Claus-Michael Lehr, Jesko Koehnke, Katharina Rox, Jörg Haupenthal, Anna K. H. Hirsch

**Affiliations:** 1 Helmholtz Institute for Pharmaceutical Research Saarland (HIPS) − Helmholtz Centre for Infection Research (HZI), Saarbrücken 66123, Germany; 2 PharmaScienceHub, Saarbrücken 66123, Germany; 3 Deutsches Zentrum für Infektionsforschung (DZIF) e.V., Partner site Braunschweig-Hannover, Braunschweig 38124, Germany; 4 Institute for Food Chemistry, Hannover 30167, Germany; 5 Department of Pharmacy, Saarbrücken 66123, Germany; 6 Laboratoire d’Excellence Inflamex, Institut National de la Santé et de la Recherche Medicale U1152, Physiopathologie et Épidémiologie des Maladies Respiratoires, Université Paris-Cité, Paris 75006, France; 7 Helmholtz International Lab for Anti-infectives, Saarbrücken 66123, Germany; 8 INSERM U1149, Centre de Recherche sur l’Inflammation, Hôpital Bichat, Université Paris-Cité, 16 rue Henri Huchard, Paris 75018, France; 9 Department of Chemical Biology (CBIO), Helmholtz Centre for Infection Research (HZI), Braunschweig 38124, Germany

## Abstract

Targeting the extracellular protease elastase (LasB) of the high-priority pathogen *Pseudomonas aeruginosa* is a promising strategy to develop second-generation, narrow-spectrum antibiotics with a novel mode of action. *P. aeruginosa* is responsible for a variety of infections, particularly of the lung. Herein, we report the structure-based optimization of a previously reported potent and selective phosphonate-based LasB inhibitor scaffold. Having improved the activity while maintaining high selectivity and favorable ADMET properties, we also demonstrate, for the first time within this scaffold, that intravenous administration leads to favorable lung retention. We could rationally align this with *in vitro* plasma protein binding. We further observed a link between physicochemical properties like logD_7.4_ and protein binding, including surfactant proteins that can impair compound activity in the lung. This multiparameter optimization paves the way for the exploration of additional indications requiring systemic treatment, such as hospital-acquired or ventilator-associated pneumonia.

## Graphical abstract

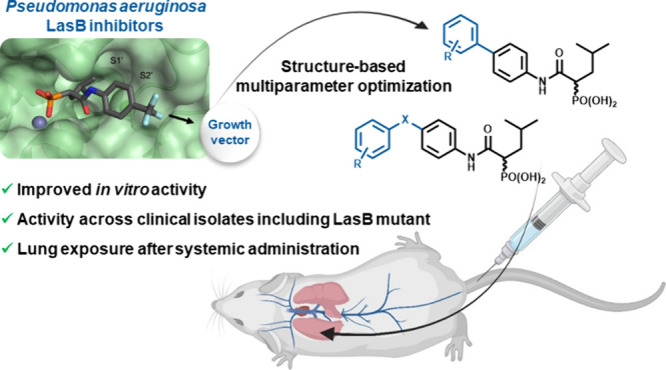

## Introduction

Representing one of the ESKAPE pathogens that is classified as high priority by the World Health Organization, *Pseudomonas aeruginosa* undoubtedly threatens public health.^1,2^ The situation is worsened by the rise of resistance against commonly used antibiotics urgently calling for the development of novel treatment options.^3–5^ In this regard, the concept of developing ‘pathoblockers’ targeting bacterial virulence is of particular interest as it offers several advantages: Given that bacteria are not killed but rather impaired in their detrimental effects on the host, selection pressure is reduced. Additionally, the human microbiome is spared as antivirulence targets tend to be species-specific, paving the way for narrow-spectrum antibiotics.^6–8^ While developing a novel pathoblocker against *P. aeruginosa* , we and others have focused on the development of elastase (LasB) inhibitors.^9–14^ The secreted zinc metalloprotease LasB plays a pivotal role in an infection with *P. aeruginosa* , facilitating invasion by cleaving components of the host connective tissue and simultaneously favoring immune evasion as several components of the immune system are substrates to LasB.^15–17^ Its extracellular localization constitutes an essential advantage considering the challenges associated with crossing the Gram-negative cell wall.^18^ During recent years, we described the hit discovery and -optimization of a thiol-based LasB inhibitor scaffold that we recently advanced significantly in terms of potency, drug metabolism and pharmacokinetics (DMPK) and drug likeness by replacing the thiol with a phosphonate.^19^ Having access to a rational pipeline of structure-based optimization guided by high-resolution cocrystal structures, *in vitro* and *ex vivo* potency assays, *in vitro* ADMET (absorption, distribution, metabolism, excretion and toxicity) and *in vivo* PK/PD, we are now optimizing the scaffold according to a specific target-lead and target-product profile (TLP/TPP).

*P. aeruginosa* is responsible for various diseases, particularly lung infections. This affects immunocompromised patients and people suffering from cystic fibrosis (CF)^20^ or noncystic fibrosis bronchiectasis (NCFB).^21^ Furthermore, hospital-acquired or ventilator-associated pneumonia (HAP/VAP) caused by *P. aeruginosa* pose a significant threat to patients, *e.g.*, in intensive-care units (ICU). Mortality in ICUs has also been connected to the presence of LasB.^22^ While CF and NCFB patients are capable of inhaling a drug, hospitalized HAP/VAP patients are treated via intravenous (IV) administration.^23^ Apart from these lung infections, a significant proportion of bacterial keratitis, especially contact lens-associated keratitis, where topical treatment is usually applied, is caused by **P. aeruginosa*.* ^24,25^

Having demonstrated *in vivo* proof-of-concept for combination treatment of a LasB inhibitor with a standard-of-care (SOC) antibiotic for both *P. aeruginosa* lung infection^19^ and *Pseudomonas* keratitis,^26^ this study aims at exploring the potential of systemic dosing of LasB inhibitors to prepare IV treatment of HAP/VAP patients. We achieved this challenge via multiparameter optimization combining structure-based optimization of the phosphonate scaffold with *in vitro* activity and ADMET profiling.

## Results

### Chemistry

Synthesis of the first derivatives commenced with a Suzuki cross-coupling reaction between corresponding bromoanilines and 4-chlorphenyl boronic acid. Subsequent EDC·HCl-mediated amide coupling with commercially available 2-chloro-4-methylpentanoic acid gave rise to the corresponding phosphonate precursors, which were subjected to an Arbuzov reaction, followed by TMSBr-mediated deprotection (Scheme 1).

**Scheme 1:**
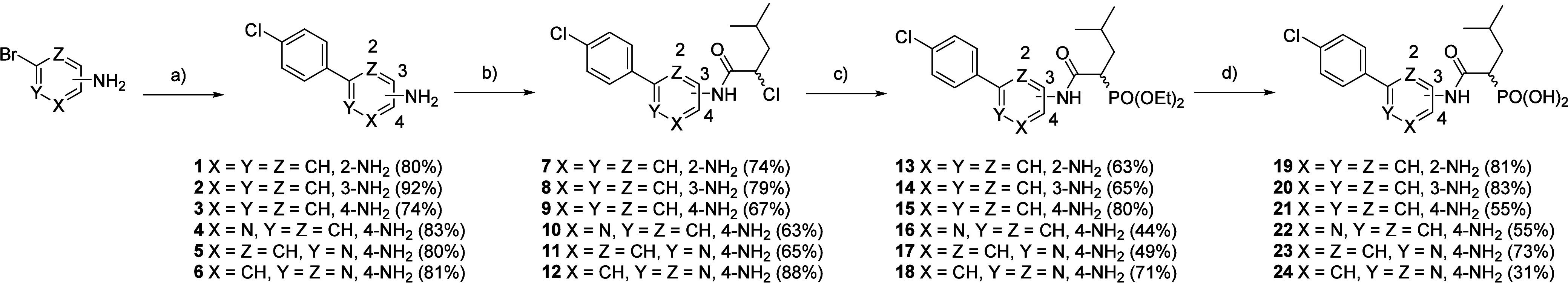
Synthesis of Compounds **19**–**24** ^a^

a Reagents and conditions: (a) 4-chlorophenylboronic acid, Na_2_CO_3_ (2 M), [Pd­(dppf)­Cl_2_], dioxane/H_2_O (4/1), microwave, 20 min; (b) 2-chloro-4-methylpentanoic acid, EDC·HCl, DCM, r.t.; (c) P­(OEt)_3_, 150 °C; d) TMSBr, DCM, r.t.

For further synthetic studies, we aimed for a more divergent strategy. Therefore, phosphonate building block **29** was synthesized. Conversion of commercially available racemic leucine into the corresponding α-bromo acid, followed by esterification and subsequent Arbuzov reaction delivered the desired building block after saponification in 35% overall yield (Scheme 2).^27^ This reaction can be performed on a large scale.

**Scheme 2:**
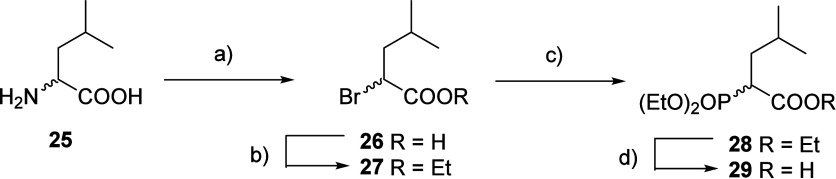
Synthesis of Phosphonate Building Block **29** ^a^

a Reagents and conditions: (a) 48% HBr, NaNO_2_, H_2_O, 0 °C to r.t., overnight, quant.; (b) H_2_SO_4_, EtOH, reflux, 2 h, 81%; (c) P­(OEt)_3_, 150 °C, 48 h, 44%; (d) NaOH, EtOH, H_2_O, r.t., overnight, 98%.

With this building block in hand, the *iso*-propoxy-substituted derivatives **35** and **36** were synthesized according to Scheme 3. Starting with a Suzuki reaction of 2-bromopyrimidin-5-amine and the corresponding boronic acids, the required arylamines were subjected to peptide coupling with building block **29**. Subsequent deprotection yielded the desired phosphonates.

**Scheme 3:**
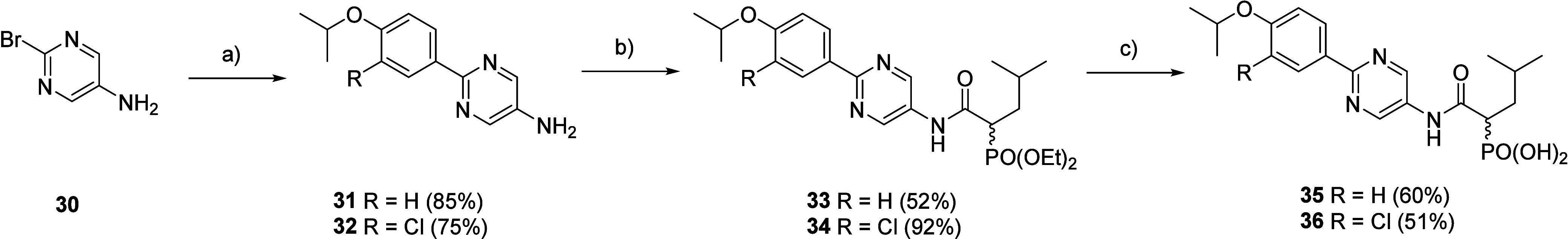
Synthesis of Compounds **35** & **36** ^a^

a Reagents and conditions: (a) 4-isopropoxyphenylboronic acid or 3-chloro-4-isopropoxyphenylboronic acid, Na_2_CO_3_ (2 M), [Pd­(dppf)­Cl_2_] or [Pd­(PPh_3_)_4_], dioxane/H_2_O (4/1), microwave, 20 min; (b) **29**, EDC·HCl, HOBt, DIPEA, DCM, r.t.; c) TMSBr, DCM, r.t.

Synthesis of heterocyclic analogues (Scheme 4) was achieved *via* a sequence of S_N_Ar, reduction and subsequent peptide coupling with the aforementioned phosphonate building block (Scheme 2) and subjection to phosphonate deprotection conditions as mentioned above (see Scheme 1).

**Scheme 4:**
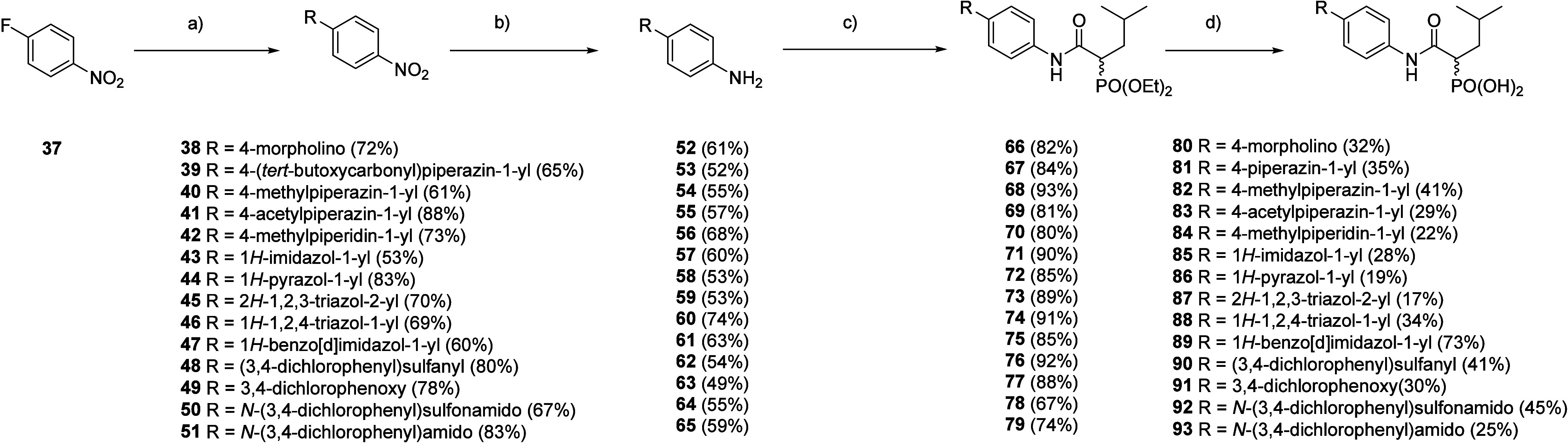
Synthesis of Hetaryl-Substituted Phosphonates **80**–**93** ^a^

a Reagents and conditions: (a) corresponding amine, K_2_CO_3_, DMF, 150 °C, overnight; (b) NH_4_Cl, Fe, EtOH/H_2_O (2/1), reflux, 2 h; (c) **29**, EDC·HCl, DCM, r.t.; (d) TMSBr, DCM, r.t.

For the linker installation, we started with synthesis of the desired substituted aniline-derived building blocks, as well as for evaluation of the aromatic residue in the Western part. Compounds **130** – **147** were synthesized from commercially available *tert*-butyl (4-aminophenyl) carbamate and corresponding acid- or sulfonyl chlorides, followed by Boc-deprotection (see for details). Subsequent coupling to phosphonate building block **29** with EDC·HCl and HOBt gave the desired phosphonates after deprotection using the established TMSBr protocol (Scheme 5).

**Scheme 5:**
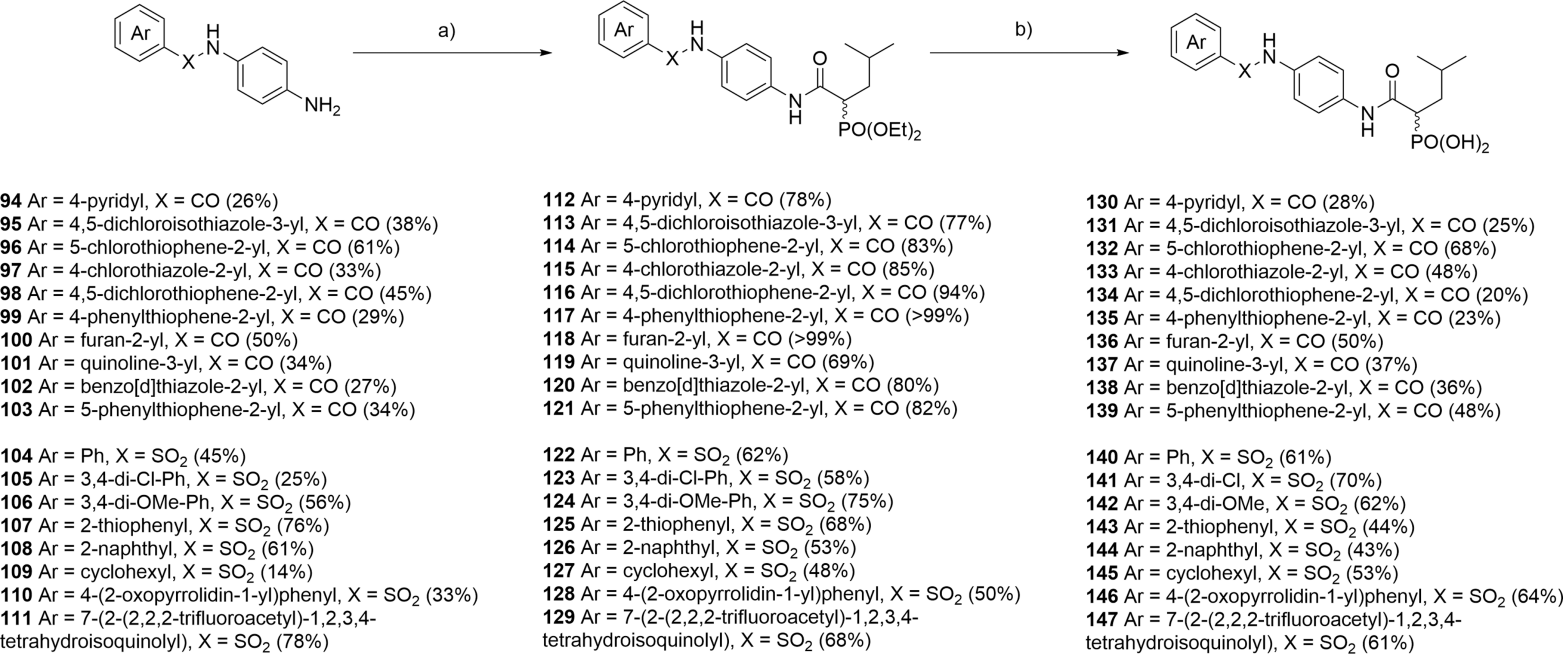
Synthesis of Compounds **130**–**147** with Sulfonamide or Amide Linker^a^

a Reagents and conditions: (a) **29**, EDC·HCl, HOBt, DCM, r.t.; (b) TMSBr, DCM, r.t.

Corresponding F- and CF_3_-substituted congeners **157** and **158** were synthesized in a similar fashion as mentioned before, relying on the established synthetic route consisting of amidation, Boc-deprotection, peptide coupling and phosphonate deprotection (Scheme 6).

**Scheme 6:**
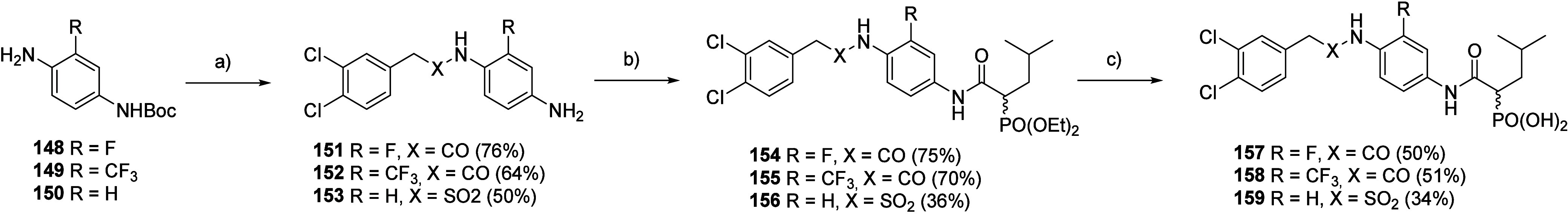
Synthesis of Compounds **157**–**159** ^a^

a Reagents and conditions: (a) (1) corresponding acid chloride, NEt_3_, DCM, r.t.; (2) TFA, DCM, r.t.; (b) **29**, EDC·HCl, DCM, r.t.; (c) TMSBr, DCM, r.t.

With respect to the monofunctional linkage, sulfur-containing compound **164** was synthesized by converting commercially available aminobenzylalcohol **160** into the corresponding phosphonate **161**, which was then transformed to benzyl chloride **162**. Nucleophilic substitution with 3,4-dichlorobenzenethiol, followed by phosphonate deprotection gave rise to the desired congener (Scheme 7).

**Scheme 7:**
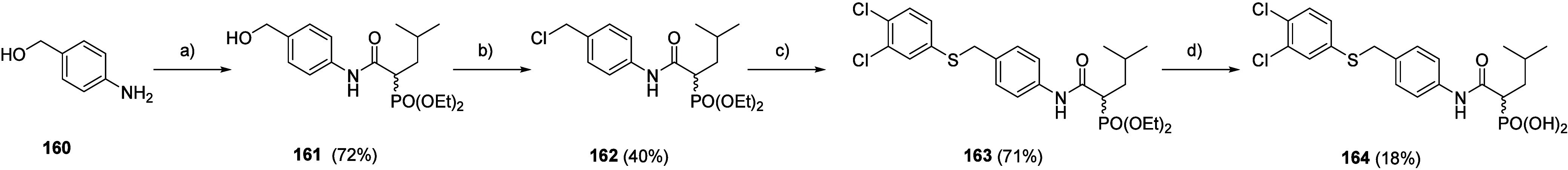
Synthesis of Compound **164** ^a^

a Reagents and conditions: (a) **29**, EDC·HCl, DCM, r.t.; (b) SOCl_2_, THF, r.t., 3 h; (c) 3,4-dichlorobenzenethiol, K_2_CO_3_, DMF, 150 °C, overnight; (d) TMSBr, DCM, r.t.

For the respective amino analogue **169**, *tert*-butyl 3,4-dichlorophenylcarbamate was reacted with commercially available benzyl bromide **165** and again our reliable sequence of reduction, peptide coupling and deprotection was applied (Scheme 8).

**Scheme 8:**
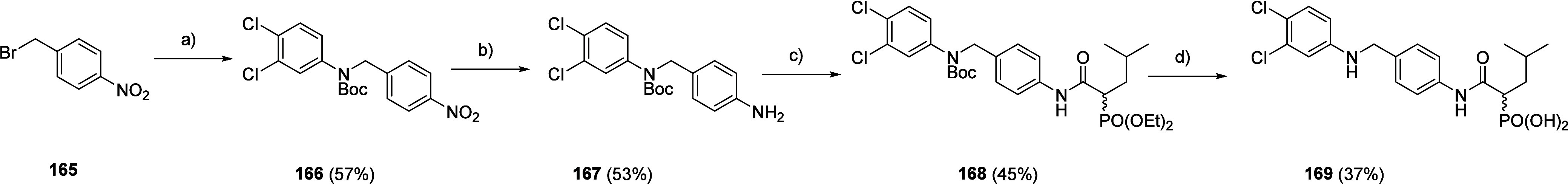
Synthesis of Compound **169** ^a^

a Reagents and conditions: (a) *tert*-butyl 3,4-dichlorophenylcarbamate, K_2_CO_3_, acetone, 60 °C, overnight, 57%; (b) NH_4_Cl, Fe, EtOH/H_2_O (2/1), reflux, 2 h, 53%; (c) **29**, EDC·HCl, DCM, r.t., 45%; (d) TMSBr, DCM, r.t., 37%.

In case of the oxygen linker, nucleophilic substitution of 4-(bromomethyl)-1,2-dichlorobenzene with Boc-protected amino phenol afforded intermediate **171**, which was then subjected to amide coupling with 2-bromo-4-methylpentanoic acid (Scheme 9). The same peptide coupling conditions were used with commercially available building blocks. Subsequent Arbuzov reaction, followed by treatment with TMSBr yielded the desired analogues.

**Scheme 9:**
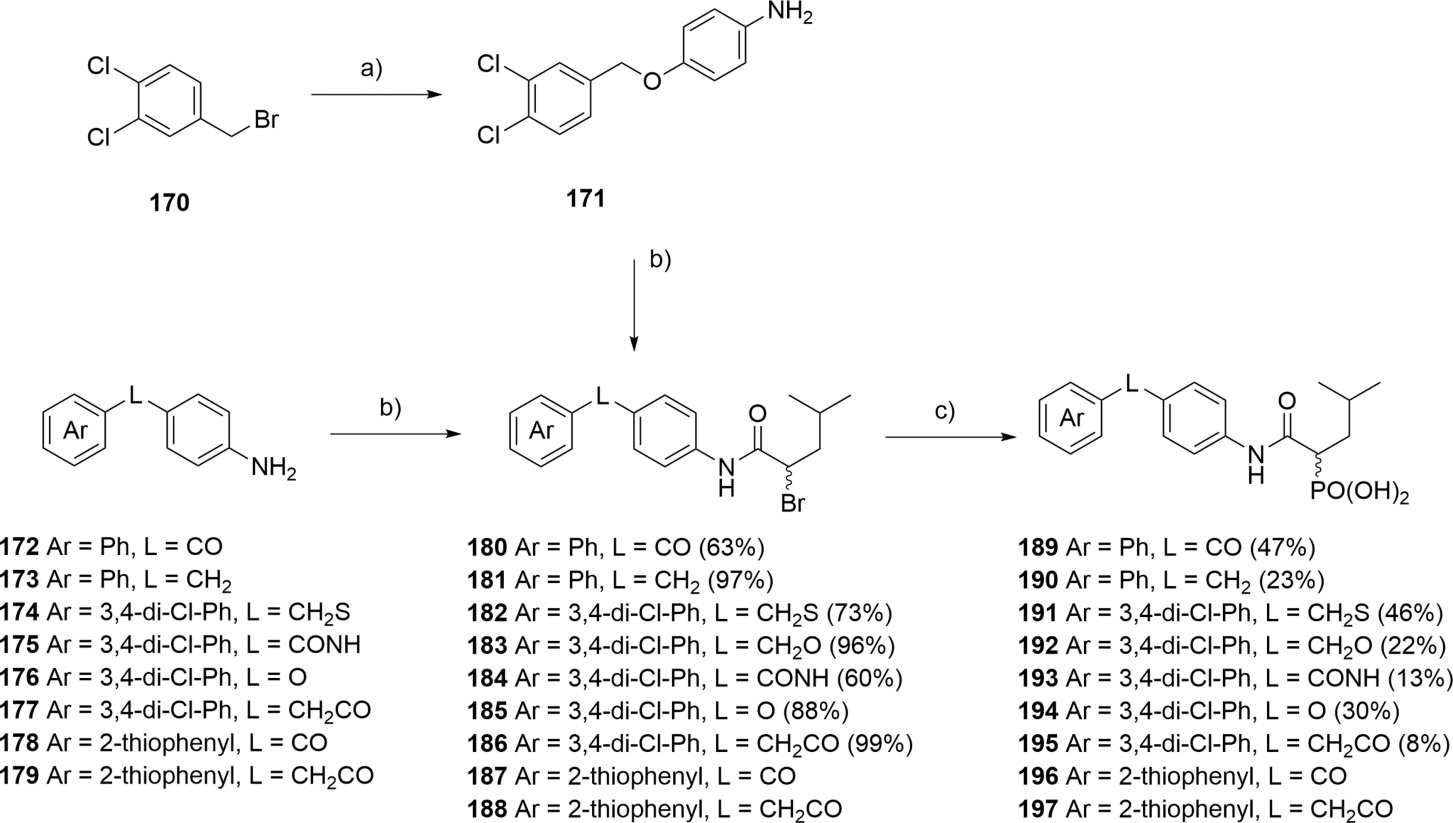
Synthesis of Compounds **189**–**197** ^a^

a Reagents and conditions: (a) (1) *tert*-butyl (4-hydroxyphenyl)­carbamate, K_2_CO_3_, DMF, r.t.; (2) TFA, DCM, r.t., 58% over 2 steps; (b) 2-bromo-4-methylpentanoic acid, EDC·HCl, DCM, r.t.; (c) (1) P­(OEt)_3_, 150 °C; (2) TMSBr, DCM, r.t.

Furthermore, we explored the attachment of *meta*-substituted residues to the phenyl core with compounds **204** and **205**. Synthesis commenced via the route already established for *para* derivatives using peptide coupling sequences (Scheme 10).

**Scheme 10:**
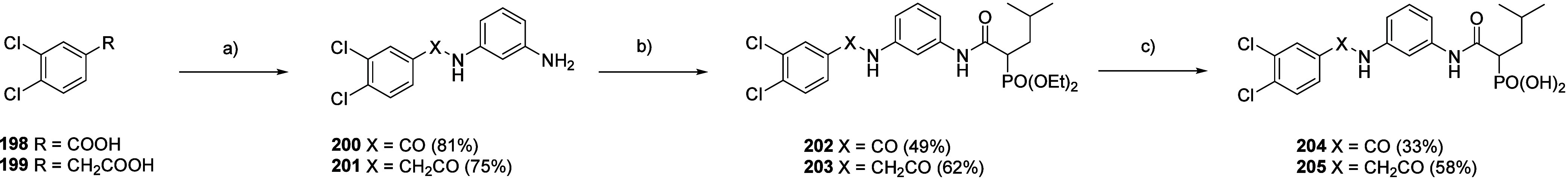
Synthesis of Compounds **204** & **205** ^a^

a Reagents and conditions: (a) (1) *tert*-butyl (3-aminophenyl)­carbamate, EDC·HCl, DCM, r.t.; (2) TFA, DCM, r.t.; (b) **29**, EDC·HCl, DCM, r.t.; (c) TMSBr, DCM, r.t.

Based on the recently obtained cocrystal structure of a first-generation phosphonate-based inhibitor (**206**) in complex with LasB (PDB code: 8CC4),^19^ we sought to explore a possible growth vector in position 4 of the aromatic ring (Figure 1). We incorporated different structural features to understand their impact on the on-target activity and on physicochemical properties that may drive *in vivo* exposure. Our synthetic endeavors started with incorporation of an additional aryl moiety attached directly to the phenyl core via a direct C–C linkage. All of the newly synthesized compounds were tested in a FRET-based inhibition assay, as established by Nishino and Powers.^28^ Compound **21** bearing the additional aryl moiety in 4-position proved to be the most active compound, displaying single-digit nanomolar activity (Table 1). The corresponding 3-substituted derivative **20** showed activity in the lower double-digit nanomolar range, whereas LasB inhibition dropped for *o* *rtho*-substituted compound **19**, which was already expected due to likely clashes in the binding pocket. Incorporation of a nitrogen atom in 3-position of the initial phenyl ring led to compound **23** in a similar activity range as the parent compound. When moving to the 2-position (compound **22**), activity decreased most likely due to a disadvantageous conformation as a result of the nitrogen lone pair being in close proximity to the carbonyl group. To further investigate the role of the binding angle between the two aryl moieties, we synthesized compound **24**, inspired by previous work from Hamed *et al.* ^29^ However, this led to a slight decrease in activity, comparable to compound **20**. Additionally, the chloro-substituent was exchanged for an electron-donating *iso*-propoxy-substituent (**35**), which gave a small improvement in LasB inhibition. When combining this motif with a chloro-substituent in 3-position (**36**), activity slightly decreased again, yet still in a gratifying range. In order to probe a heterocyclic substituent as a decoration in 4-position of the core structure, we started with various five-membered heteroaromatic compounds, this time with direct N–C connection. Addition of imidazole (compound **85**) led to increased activity in comparison to parent compound **206**. Corresponding pyrazole compound **86**, as well as the triazoles **87** and **88** displayed similar activities in between the parent compound (**206**) and **85**. Exchanging the 4-substituent to morpholine (compound **80**) resulted in no significant change in LasB inhibition. When we introduced a piperazine (compound **81**), however, the IC_50_ value increased to 174 nM. Methylation of the nitrogen atom to mask the hydrogen-bond donor led to a less than 2-fold change in IC_50_ (**82)**. When converted into the corresponding amide **83** with its hydrogen-bond acceptor function, a greater than 5-fold improved activity toward low double-digit nanomolar range was observed. A similar result was obtained when compound **84**, the piperidine analogue of **82**, was tested, prompting the question which role hydrogen bonding in this area might play in binding.

**Figure 1:**
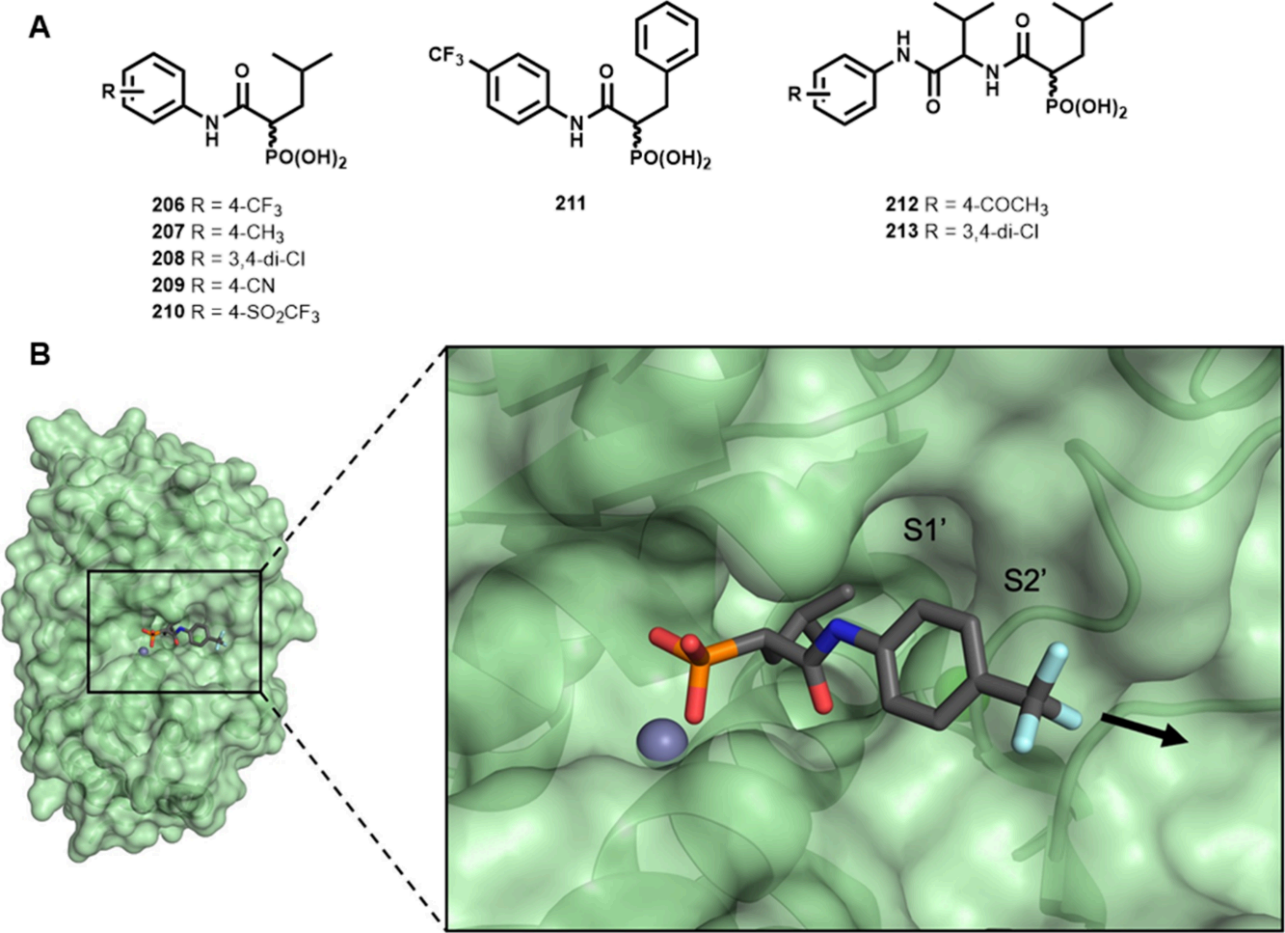
(A) Structures of phosphonate-based inhibitors published previously. **206**–**211** correspond to compounds **4b, 4a**, **4c**, **4k**, **4l**, and **4y** reported in Konstantinovic *et al.*;^19^ **212** and **213** correspond to compounds **30** and **9** in Kiefer *et al.* ^26^ (B) Co-crystal structure of LasB in complex with a recently published phosphonate inhibitor **206** (PDB code: 8CC4, adapted from Konstantinovic *et al.* ^19^). The potential growth vector for further structure-based optimization is indicated by an arrow.

**Table 1:**
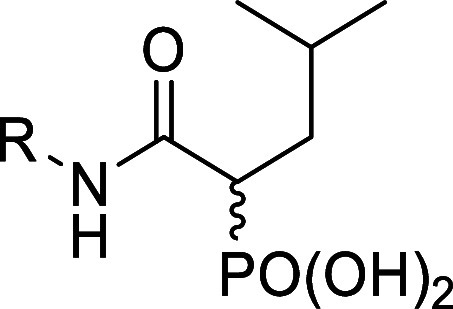

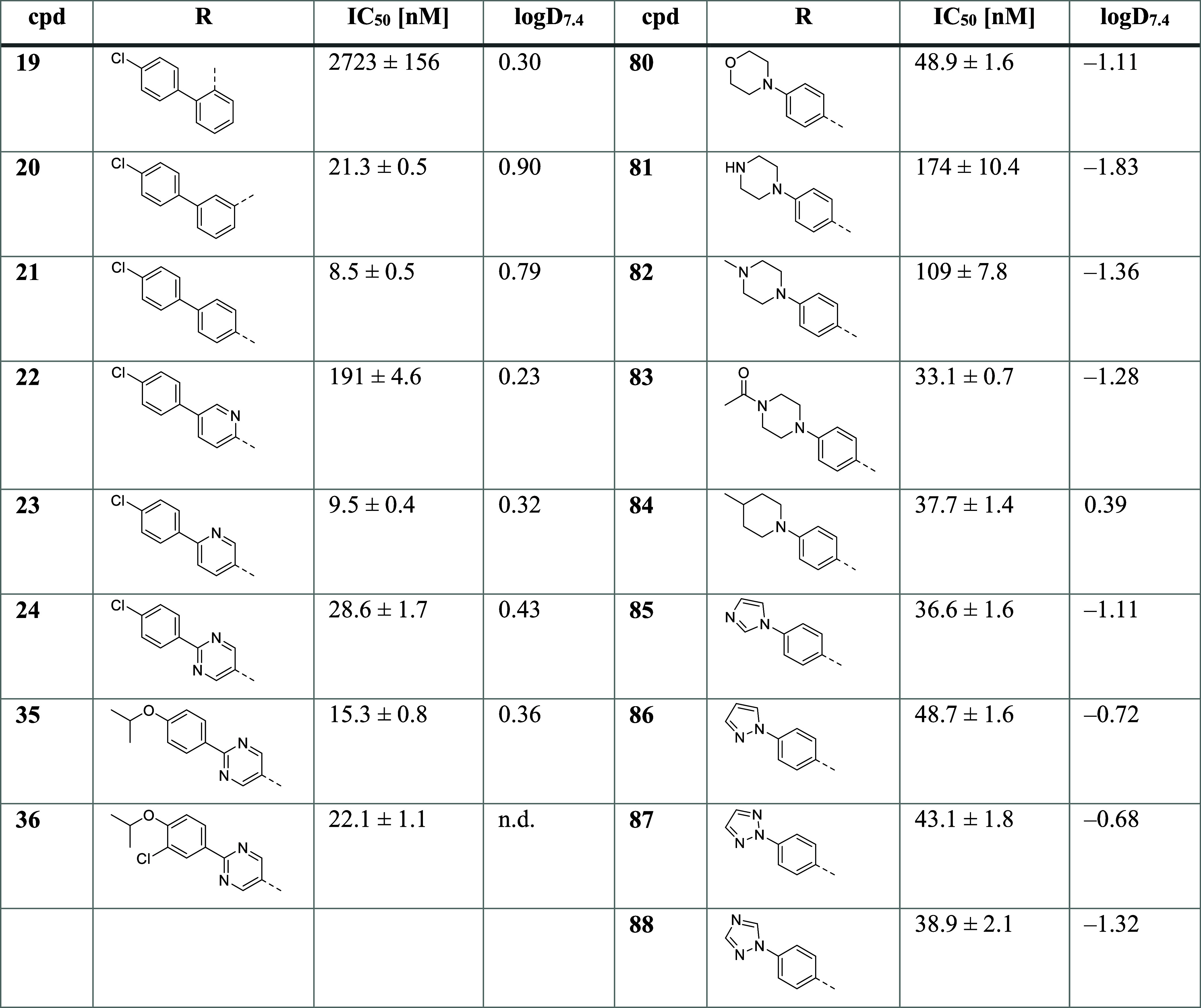
*Pseudomonas aeruginosa* LasB Inhibition in the FRET-Based Inhibition Assay and Chromatographic logD_7.4_ for Bicyclic Phosphonates^a^

a Means and SD of at least two independent experiments.

Based on the promising results from our first growth-vector exploration, we aimed to investigate the chemical space around the phenyl core. Our aim was to explore different linker moieties between the aromatic rings also comparing hydrogen bond donor/acceptor functions, rather than connecting them directly (Table 2), and to modify the Western aromatic part of the molecule (Table 3). Starting with introduction of bifunctional linkers, amide-connected compounds **93** and **193** were synthesized, both showing good double-digit nanomolar activity, with a preference for N–C linkers. Adding an additional methylene spacer did not affect the inhibitory activity (compound **195**). This compound was further modified to study the influence of conformational changes. When introducing a fluorine substituent in 3-position (compound **157**), activity was only slightly decreased, yet incorporation of a trifluoromethyl substituent (compound **158**) in the same position led to a more pronounced drop in potency. Since installation of the additional substituent to the core structure in 3-position still maintained good activity, this substitution pattern was combined with the corresponding linker units, delivering compounds **204** and **205**, showing a significant drop in activity. It is also worth mentioning that the methylene spacer on the amide function in 3-position yielded an almost 2.5-fold more potent LasB inhibitor (**205**). Bioisosteric replacement of the amide linker with a sulfonamide showed similar trends in structure–activity relationship for compounds **92**, **141** and **159**, yet with the sulfonamide linkage being more potent. Changing toward monofunctional linker units yielded compounds comparable to sulfonamide-containing inhibitors. Again, hydrogen-bond donor (compound **169**) or acceptor (*e.g.*, compounds **91**, **169**) functions, as well as an added methylene spacer did not significantly impact potency. Incorporation of a carbonyl linkage (compound **189**) or a simple methylene linker (compound **190**), however, resulted in decreased activity.

**Table 2:**
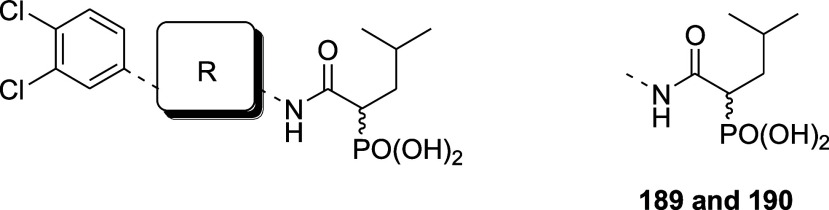

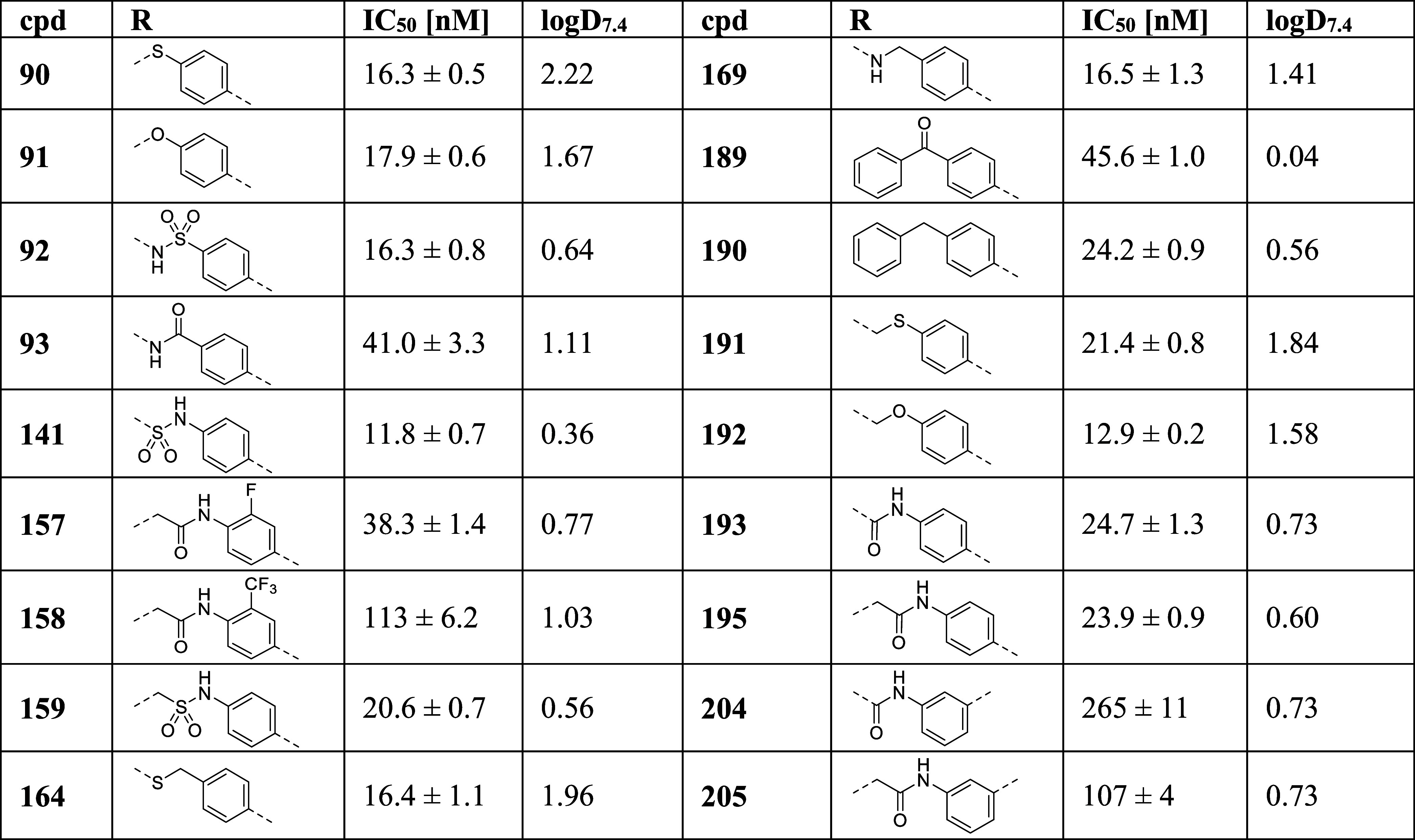
*Pseudomonas aeruginosa* LasB Inhibition from the FRET-Based Assay and Chromatographic logD_7.4_ for Linker-Modified Phosphonates^a^

a Means and SD of at least two independent experiments.

**Table 3:**
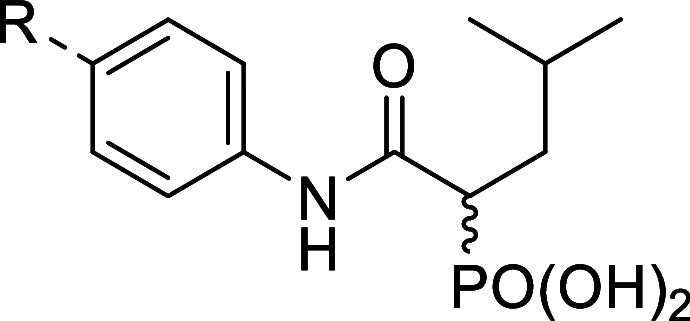

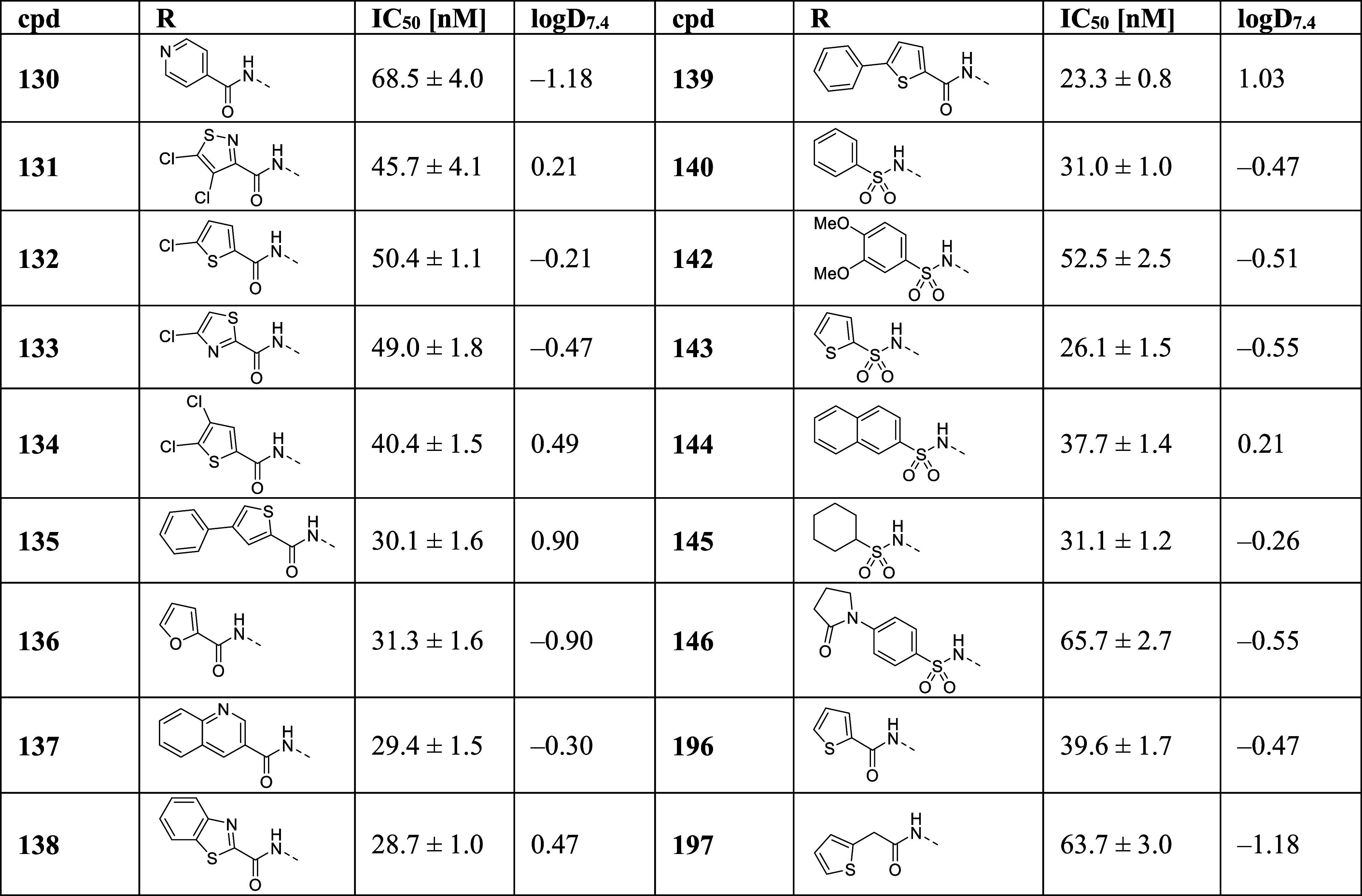
*Pseudomonas aeruginosa* LasB Inhibition from the FRET-Based Assay and Chromatographic logD_7.4_ for Phosphonates with Modified Aryl Unit^a^

a Means and SD of at least two independent experiments.

As mentioned before, we also focused on modification of the additional aryl unit. For our investigations we kept the N–C connection and started with bioisosteric replacement of the phenyl ring with thiophene. In this case, the additional methylene spacer proved to slightly decrease activity (compound **197**), whereas a simple amide linkage (compound **196**) resembled the activity of compound **93**. Furthermore, mono- and dichlorinated derivatives **131** – **134** were synthesized, all with solid double-digit nanomolar activity. Exchanging thiophene for furan gave a similarly potent compound (**136**). Similar potency was displayed by compounds **137**, bearing a quinoline motif, and **138** with a benzothiazole, which inspired us to investigate further growing possibilities. Disconnecting the phenyl moiety from the latter resulted in compounds **135** and **139** with comparable activity, yet slightly in favor of the 2,5-disubstitution pattern regarding thiophene. Next, we focused on further sulfonamide modifications. Omitting the dichloro motif of **141** led to a more than 2-fold drop in activity (compound **140**) and replacement with the corresponding dimethoxy pattern decreased activity even further (compound **142**). Also, for the sulfonamide derivatives, replacing the Western phenyl moiety with a thiophene (compound **143**) led to a similar activity as for the phenyl derivative, while a pyridine derivative showed a higher IC_50_ (**130**). Even replacement of the aromatic Western part with a cyclohexyl substituent gave an active compound (**145**). Further enlargement was possible as well, with naphthyl-substituted compound **144** being in a similar activity range. Incorporation of a pyrrolidone substituent, however, led to a drop in potency (compound **146**). It is worth noting that all of the above-mentioned compounds do not show any antibacterial activity against PA14 in agreement with our pathoblocker approach (Table S1).

### X-ray Crystallography

The extensive optimization of LasB inhibitors was based primarily on the strategy of exploring the growth vector previously identified by X-ray crystallography, resulting in compounds with improved *in vitro* LasB inhibition. To gain more detailed insights into the binding mode and to confirm the design principle, the most potent biaryl compounds, **21** and **35**, and the frontrunner compound with a sulfonamide linker, **141**, were cocrystallized with LasB. The LasB–compound complex structures were determined to high resolution. Our optimized compounds share structural motifs with previously published LasB inhibitors, such as the phosphonate for zinc coordination at the active site, as well as the *iso*-butyl moiety and the amide linker attached to the aryl residue (Figure S1).^19,26^ Thus, key interactions, such as hydrogen bonds with the side chains of His223, Glu141 and Asn112, hydrophobic interactions with Leu197 in the S2’ pocket, and bidentate hydrogen bonds with Arg198 were conserved in all three cocrystal structures (Figure 2, Figure S4). In addition to these common interactions, there are major differences in the binding mode of the biaryl compounds in terms of the substitution pattern of the additionally introduced aromatic ring and the linker region. In the cocrystal structures of the two biaryl compounds without linker (**21** and **35**), an additional CH–π interaction with the conformationally flexible side chain of Met128 was observed (Figure 2, C, Figure S4, B). While only one distinct conformation of the inhibitor was observed for **21**, the electron density observed for **35** could confidently be assigned to an alternative conformation of the compound. As a result, the side chain of Met128 also adopted two distinct conformations, one allowing CH–π interactions with the aromatic moiety, while the other induced a slight rotation of the terminal aromatic ring around the C–C bond (Figure S4). We hypothesize that the CH–π interaction between LasB and **21** was stronger than that between the protein and **35** (no alternative conformation observed). The presence of a pyrimidine substituent within the biphenyl part of **35** enforces a more planar orientation between the two aromatic rings, which constrains conformational flexibility and potentially impairs an effective CH–π interaction. In agreement with this supposition, the *in vitro* inhibition of LasB was almost twice as strong for **21** (IC_50_ of 8.5 ± 0.5 nM) as that of **35** (IC_50_ of 15.3 ± 0.8 nM).

**Figure 2:**
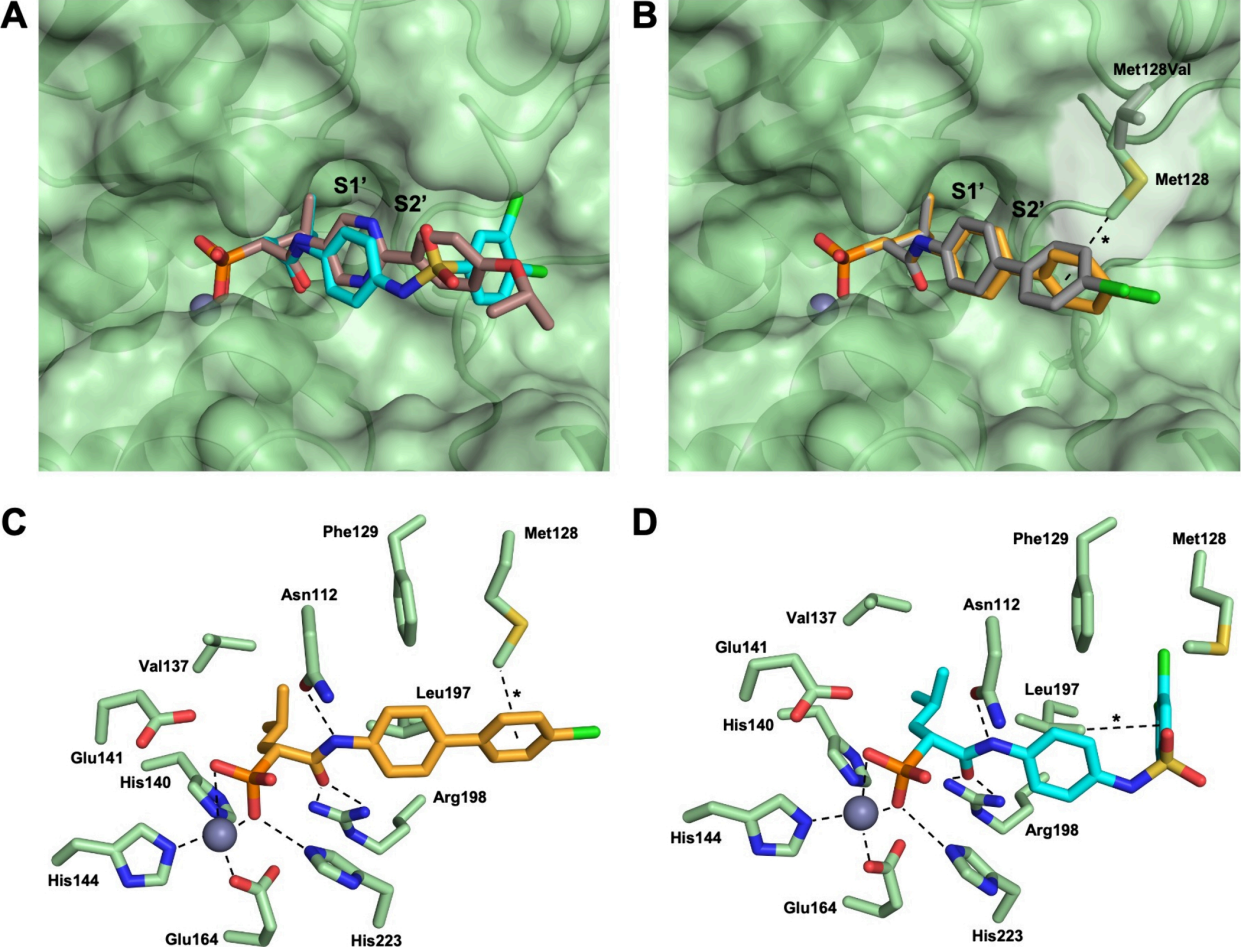
Crystal structure of LasB (green) in complex with **21**, **141**, and **35** (PDB code: 9FRY, 9FRZ and 9FS0). (A) Surface representation and superposition of LasB in complex with **141** (carbon atoms: cyan) and **35** (carbon atoms: dark red). (B) Surface representation and superposition of wild-type LasB and the Met128Val mutant in complex with **21**. The compound bound to the wild-type structure is represented as sticks with carbon atoms colored in dark yellow. The CH–π interaction between the side chain of LasBMet128 and the aromatic ring of **21** is highlighted by an asterisk. The ring conformation is slightly rotated in the cocrystal structure of LasB Met128Val in complex with **21**, for which carbon atoms are colored in gray. (C) Interactions between LasB and **21**. The CH–π interaction is indicated by an asterisk. (D) Interactions between LasB and **141**. The dichlorine-substituted aromatic ring is engaged in a CH–π interaction with the side chain of LasB Leu197 and highlighted by an asterisk.

In a previously reported sequence analysis, mutations in the *lasB* gene were identified in clinically relevant isolates of *P. aeruginosa* , which were characterized biochemically and in terms of activity.^30^ Most mutations had no effect on LasB activity and Met128Val was the only mutant located near the active site (present in 16 out of 2746 analyzed *P. aeruginosa* LasB sequences, 0.5% prevalence). In light of the favorable CH–π interaction of **21** and **35**, we sought to investigate the effects of the Met128Val mutation on the compounds’ activity. The Met128Val mutation has a comparatively minor impact on the *in vitro* activity of compounds, as LasB inhibition remains within a favorable double-digit nanomolar range. Compound **21** shows an almost 7-fold loss in inhibition (IC_50_ of 58.9 ± 11.7 nM), whereas **35** is less affected with a 2.7-fold reduction (IC_50_ of 42.0 ± 10.4 nM), consistent with an alternative binding conformation not reliant on CH–π interactions. The high-resolution crystal structure of LasB Met128Val in complex with **21** revealed merely a slight rotation of the monochlorine-substituted aromatic ring, similar to the alternative conformation observed in the cocrystal structure with **35** (Figure 2, A, Figure S4).

To obtain detailed information on the effects of the introduction of a linker between the two aryl moieties, we obtained the high-resolution crystal structure of LasB in complex with **141**. Similar to the LasB structures in complex with **21** and **35**, the key interactions were retained, while the largest difference in binding was observed for the sulfonamide and the Western part of the compound. Bound to LasB, the sulfonamide linker induces a compound conformation in which the dichloro-substituted ring is oriented at nearly 90° to the aromatic ring engaged in the lipophilic S2’ pocket. This prevents the previously observed interaction with Met128 and enables instead a CH–π interaction of the terminal aromatic ring with the side chain of Leu197 (Figure 2, D).

Taken together, the binding mode of the different biaryls differs subtly depending on the substitution pattern of the aromatic ring and the linker region, as seen in the overlay (Figure 2, B). The shared and respective compound-specific interactions underline the structural versatility of the biaryl class in targeting LasB, which also becomes clear with the overall high activity of most of the designed inhibitors. These findings allow for further rational, structure-based design of LasB inhibitors.

### LasB Inhibition in the Presence of Pulmonary Surfactant

*In vivo*, the LasB inhibitors eventually need to be active in the lungs where pulmonary surfactant is present. It has been shown that surfactant can significantly impair the activity of the antibiotic daptomycin.^31^ Hence, we assessed potential effects of surfactant on the *in vitro* activity of selected LasB inhibitors by adding 1% porcine lung surfactant to the FRET assay. The results show that the activity of several compounds is unaffected by the presence of surfactant (*e.g.*, **81**, **82, 130**), while some LasB inhibitors show a > 3-fold shift in IC_50_ (*e.g.*, **21**, **141**, **195**, Table 4).

**Table 4:** IC_50_ Values in the FRET-Based LasB Inhibition Assay in the Presence and Absence of 1% Porcine Pulmonary Surfactant (equaling ∼0.8 mg/mL) or 0.8 mg/mL DPPC (Dipalmitoylphosphatidylcholine)^a^

cpd	FRET IC_50_ [μM]	FRET IC_50_ in the presence of 1% surfactant [nM]	fold increase in the presence of surfactant	FRET IC_50_ in the presence of 0.8 mg/mL DPPC [nM]	surfactant protein binding [%]
**21**	8.5 ± 0.5	45	5.3	10.3 ± 0.7	37.2 ± 0.8
**23**	9.5 ± 0.4	31 ± 13	3.2	n.d.	n.d.
**35**	15.3 ± 0.8	34 ± 11	2.2	n.d.	n.d.
**81**	174 ± 10.4	221 ± 55	1.3	120 ± 8	n.d.
**82**	110 ± 7.8	159 ± 52	1.4	n.d.	n.d.
**130**	68.5 ± 4.0	68.7 ± 0.71	1.0	46.8 ± 1.9	0
**141**	13.2 ± 0.4	153 ± 43	12	12.5 ± 0.4	44.2 ± 2.5

a Percent (%) surfactant protein binding was determined after 8 h of equilibration using rapid equilibrium dialysis. n.d.: not determined.

Pulmonary surfactant is composed of phospholipids and surfactant proteins. In order to determine the reason for the reduced activity of some LasB inhibitors, we measured binding to surfactant proteins applying rapid equilibrium dialysis. This setup uses a semipermeable membrane between two chambers, allowing distribution of compounds based on their binding to proteins, and is usually applied to determine plasma protein binding (PPB) as done below. We adapted the setup using assay buffer containing 1% surfactant instead of plasma. Indeed, we observed that **21** and **141**, both giving a > 4-fold increase in FRET IC_50_, show significant protein binding of ∼ 40%, while **130** not losing activity also does not bind to surfactant proteins. Hence, the impact on activity could be attributed to the binding to surfactant proteins in contrast to what has been reported for daptomycin, where the loss of activity is due to the interaction with phospholipids.^31^ Since the membrane used in the assay has a molecular weight cutoff of 8 kDa, interactions with phospholipids can be excluded, as these would permeate. Confirming these observations, the *in vitro* activity of compounds **21**, **130**, **141** and **81** with and without shift in the presence of surfactant is not impaired by 0.8 mg/mL diphosphatidylcholine (DPPC), the main component of pulmonary surfactant (Table 4).^32^

### Assessment of Selectivity over Human Off-Targets

Since LasB is a zinc metalloprotease, we continued to explore potential off-target effects on mammalian metalloproteases. Particularly, human matrix-metalloproteases are of interest due to their versatile roles in several physiological and also pathological processes.^33^ We further investigated activity against COX-1 and tumor-necrosis factor α-converting enyzme (TACE). Importantly, all new derivatives both with and without linker could maintain the excellent selectivity profile determined previously for the monoaryl phosphonates (Table S1). Hence, growing the molecule deeper into the pocket does not lead to unwanted off-target activities.

### Determination of *In Vivo* Efficacy in Mice Treated Intranasally with LasB

Based on the previously demonstrated increased lethal effect of WT PAO1 bacteria versus Δ*lasB* PAO1 ^34^ and that of purified LasB instilled intranasally into mouse lungs,^35^ we applied the latter model to test a selection of potent inhibitors from this series before investigating drug metabolism and pharmacokinetic (DMPK) properties. As shown in Figure 3, LasB inhibition led to an increase in survival, with 100% survival observed for the most potent compound tested, **21**.

**Figure 3:**
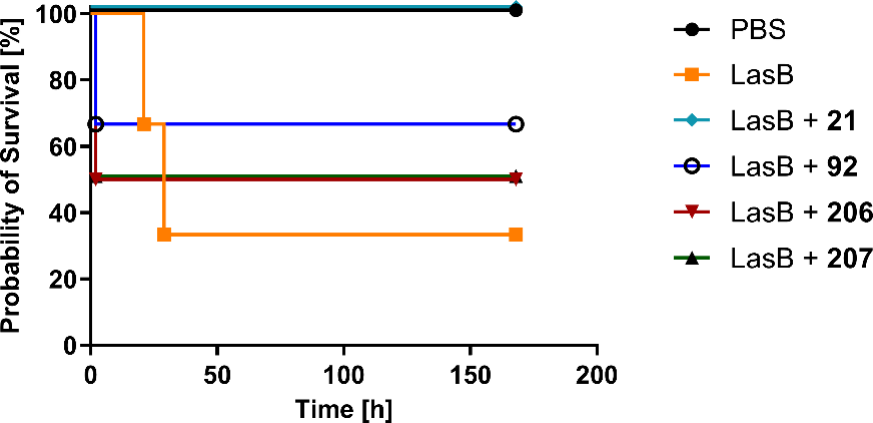
Survival of mice treated with LasB inhibitors. Pure LasB was instilled intranasally into mice together with preincubated LasB inhibitors **21**, **92**, **206** and **207** at a molar ratio of 1/10. Curves represent groups of 3–4 animals.

### DMPK Profiling

In preparation of *in vivo* PK studies, we determined the *in vitro* ADMET profile of the most promising biaryls. The results showed generally high kinetic solubility, low logD_7.4_ mostly in the negative range and high metabolic and plasma stability. Additionally, Calu-3 cell permeability was shown to be low (P_app_ < 2 × 10^–6^ cm/s, Table 5). This profile is highly similar to what we have reported before on monaryls^19^ and dipeptides (Figure 1).^26^ For representative biaryl **35**, we further confirmed these findings across species with high stability in rat and minipig liver fractions and plasma (Table S2). Regarding potential cytotoxicity, the newly designed inhibitors maintained the nontoxic properties on the lung cell line A549. This clean profile was also confirmed against HepG2 and HEK293 cells (Table S1). Furthermore, selected biaryls with linker (**141**) and without (**23**, **35**), were shown to be nontoxic against zebrafish larvae (Table S3), underlining the safety of the scaffold.

**Table 5:** *In Vitro* ADMET (Absorption, Distribution, Metabolism, Excretion and Toxicity) Profiling of Selected Biaryls^a^

cpd	logD_7.4_	S9 Cl_int_ [μL/mg/min] mouse	plasma *t*_1/2_ [min] mouse	Calu-3 P_app_ [10^–6^ cm/s]	A549 viability inh. [%]	PPB [%] mouse
**21**	0.79	<5.8	>240	0.44 ± 0.35	<10	98.3 ± 0.1
**23**	0.32	<5.8	>240	1.25 ± 0.73	<10	97.2 ± 0.9
**35**	0.36	<5.8	>240	0.49 ± 0.13	<10	95.0 ± 0.6
**81**	–1.83	<5.8	>240	0.75 ± 0.28	<10	60.6 ± 8.6
**82**	–1.36	<5.8	>240	0.63 ± 0.18	<10	33 ± 13
**130**	–1.18	<5.8	>240	1.63 ± 1.02	<10	61.7 ± 5.1
**138**	0.32	<5.8	>240	n.d.	<10	96.4 ± 0.76
**141**	0.36	<5.8	>240	0.37 ± 0.20	<10	98.3 ± 0.1
**206**	0.02	<5.8	>240	2.76 ± 1.15	<10	93.8 ± 1.0
**207**	–0.64	<5.8	>240	0.97 ± 0.21	<10	91.8 ± 2.3
**208**	0.06	<5.8	>240	1.27 ± 0.33	<10	98.9 ± 0.70
**209**	–0.84	<5.8	>240	n.d.	<10	79.6 ± 1.2
**210**	0.23	<5.8	>240	n.d.	<10	97.5 ± 0.41
**211**	0.10	<5.8	>240	n.d.	10 ± 4	89.9 ± 0.77
**212**	–0.58	<5.8	>240	0.86 ± 0.39	<10	86.9 ± 2.1
**213**	0.41	<5.8	>240	0.71 ± 0.24	<10	97.6 ± 0.59

a Monoaryls **206–211** reported previously^19^ are shown as a comparison, as well as two dipeptides **212** and **213**.^26^ Growth inhibition of A549 cells was determined at 100 μM.

The primary aim of this study was to assess bioavailability of our LasB inhibitors in the lungs after IV administration. As anticipated, the biaryls show good ELF/plasma ratios indicating a good retention in ELF and lung tissue when administered topically via intratracheal instillation (Table S4). Despite their low cell permeability, we were eager to assess lung permeation after systemic administration for this highly potent compound scaffold. Aiming to obtain first information on their lung permeation from the bloodstream, we subjected a small set of compounds to a murine cassette PK study. We combined four structurally diverse phosphonates: biaryl with linker **141**, biaryl without linker **21**, biaryl without linker **81** that is substituted with a polar piperidine and **207** as one representative from the monoaryl class reported previously,^19^ dosing at 2 mg/kg each.

Looking at the resulting terminal lung levels (Figure 4), it became apparent that only two of the four compounds applied were detected in lung tissue and epithelial lining fluid (ELF) after 5 h post administration, namely monoaryl **207** and biaryl **81**. Looking at the plasma concentration time profiles, three of the four compounds still had considerable levels at 5 h, *i.e.* **21**, **207** and **81**, whereas **141** was cleared from plasma already after 15 min post administration (Figure S8, A). Despite similar terminal plasma levels for the three compounds **21**, **207** and **81**, only two distributed well into ELF and lung tissue suggesting that **21** did not penetrate well despite sufficient plasma concentrations. Considering the *in vitro* ADMET properties of the compounds tested, it became apparent that the only difference lies in PPB, with the two compounds reaching the lung being characterized by PPB below 98% (Table 5). In line with the high unbound fraction of **81**, this compound shows comparably high clearance (Table 6). However, **207** as well as **81** showed similarly high parent compound levels in urine, suggesting additional possibly metabolic clearance of **81** compared to **207** (Figure S8, B). Compounds **207** and **21** are similar in terms of plasma *t*_1/2_, volume of distribution and clearance. Since reduced free drug levels resulting from high binding to plasma proteins might not be sufficient to enable significant lung exposure, these results prompted us to investigate in more detail which PPB window would be beneficial for lung exposure for the phosphonate class.

**Figure 4:**
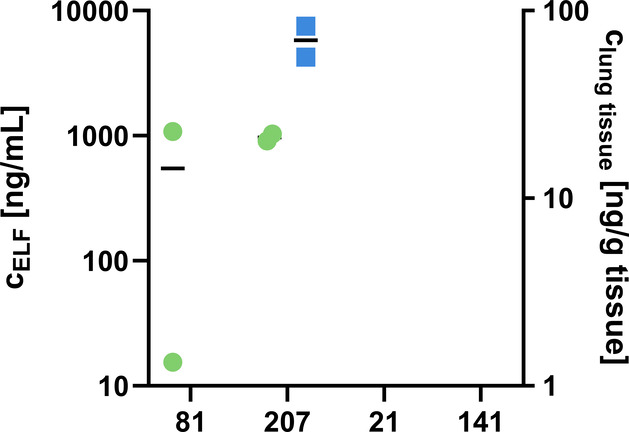
Lung levels after first intravenous cassette showing epithelial lining fluid (ELF) levels in green and lung tissue levels in blue.

**Table 6:** Pharmacokinetic (PK) Parameters after Intraven Cassette Dosing of **207**, **21** and **81** at 2 mg/kg Each^a^

PK parameter	21	81	207
*T*_1/2_ [h]	2.30 ± 0.4	13.3 ± 12.6	1.90 ± 1.2
C_0_ [μg/mL]	8.55 ± 6.96	0.911 ± 0.481	2.64 ± 1.03
AUC_0‑t_ [μg/mL h] ELF	4.68 ± 1.18	0.789 ± 0.278	4.41 ± 1.65
MRT [h]	2.8 ± 0.6	18.4 ± 18.5	2.5 ± 1.4
V_Z_obs_ [L/kg]	1.19 ± 0.4	11.6 ± 1.6	0.99 ± 0.1
Cl_obs_ [mL/min/kg] lung	5.89 ± 1.2	19.66 ± 20.3	7.25 ± 3.9

a For **141**, no PK parameters could be calculated as the compounds were only found in plasma until 15 min.

Hence, we determined PPB for a wider range of potent inhibitors (Table 5). To obtain a better perspective about general trends within the phosphonate class, we included representatives from classes other than the biaryls, namely the initial monoaryls (**206–211** ^19^) and two dipeptides (**212**, **213** ^26^). As we observed PPB to correlate with logD_7.4_ (R^2^ = 0.8628, Figure S9), we based our selection of LasB inhibitors to be tested in protein binding assays on the expected PPB as derived from measured logD_7.4_. Taken together, we identified a broad range of protein binding among the phosphonates from 33–99%. Expansion of the initial small correlation of murine PPB with logD_7.4_ with the newly generated data resulted in a similar trend (R^2^ = 0.8385), confirming applicability of our approach to base PPB testing on experimental logD_7.4_ (Figure S9).

As a next step, we subjected a selection of compounds across a PPB range from 33–95% to cassette PK studies at 2 mg/kg dosed intravenously as above. Since we did not detect compound in the lung when PPB was above 98%, we excluded compounds with very high PPB > 98%. Terminal lung levels determined are shown in Figure 5. Confirming our hypothesis derived from the initial smaller PK study, there was indeed a trend between lung exposure and PPB. We determined favorable lung exposure for the PPB range between 80 and 95% where actually all compounds were detected in lung tissue and ELF. Below 80%, lung levels dropped significantly or were not detectable. It is worth mentioning that corresponding terminal plasma levels of compounds that were not detected in ELF and/or lung tissue were in a similar range as for compounds detected in lung tissue and ELF. Thus, detectability in lung tissue and ELF was not only dependent on the plasma concentration time profile (Figure S10, A). While this might seem to be inconsistent with the proposed general trend, renal excretion supposedly serves as an explanation for this observation as it is known that low PPB leads to faster renal excretion of the free drug.^36^ The significantly higher clearance of **82** and **130** confirms this hypothesis (Table 7). Moreover, both compounds were detected at high concentrations in urine (Figure S10, B). However, also compounds showing good lung retention and moderate clearance appeared at high concentrations in urine, such as **35**. Thus, we assume that additional clearance mechanisms take place for low protein-bound compounds which need to be investigated further. Taken together, the findings suggested a sweet spot of PPB where free drug levels are on the one hand high enough to lead to sufficient permeation into the lung but on the other hand not so low that fast elimination of the free drug impairs lung permeation. Based on the data reported here, we have identified this sweet spot to be between 80 and 95% for the phosphonates under these conditions.

**Figure 5:**
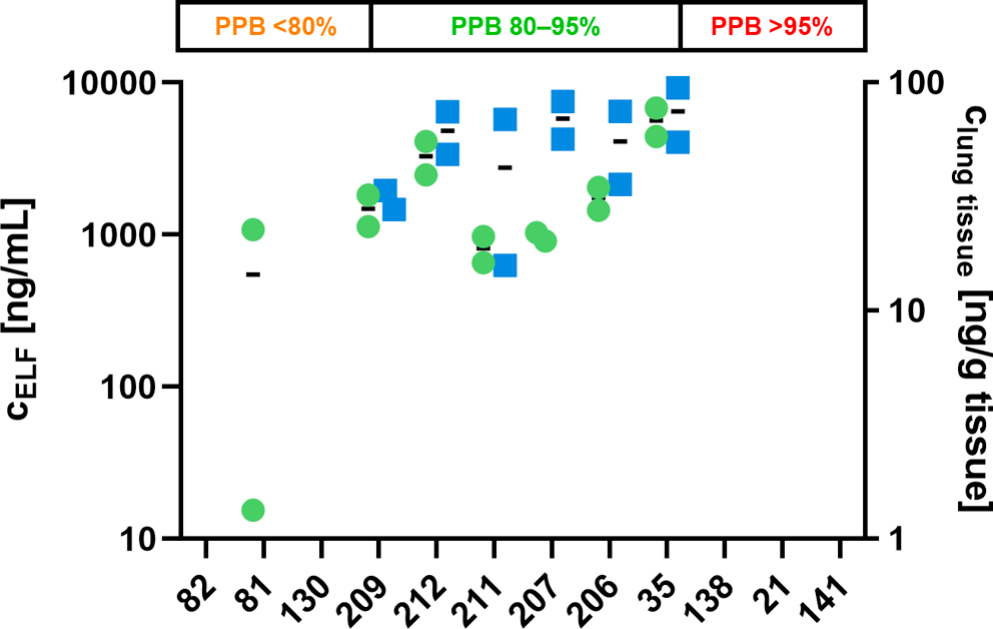
Combined lung levels of additional intravenous cassettes showing epithelial lining fluid (ELF) levels in blue and lung tissue levels in red. Compounds on the *x*-axis are ranked by PPB from low (**82**) to high (**141**).

**Table 7:** Pharmacokinetic Parameters after intravenous Cassette Dosing of **82**, **130**, **209**, **205**, **211**, **204**, **138** and **35** at 2 mg/kg Each^a^

PK parameter	**35**	**82**	**130**	**138**	**206**	**209**	**211**	**212**
*T*_1/2_ [h]	2.20 ± 0.7	1.10 ± 0.2	0.33 ± 0.0	n.d.	1.53 ± 0.2	1.54 ± 0.2	1.12 ± 0.1	3.42 ± 0.1
C_0_ [μg/mL]	0.900 ± 0.131	0.116 ± 0.060	0.194 ± 0.021	1.18 ± 0.043	8.14 ± 0.0458	8.33 ± 4.06	5.19 ± 0.86	18.96 ± 13.95
AUC_0‑t_ [μg/mL h]	2.145 ± 0.293	0.216 ± 0.0348	0.075 ± 0.021	0.309 ± 0.037	17.5 ± 4.59	16.27 ± 1.92	7.40 ± 1.48	13.16 ± 3.02
MRT [h]	3.0 ± 1.0	1.4 ± 0.4	0.42 ± 0.0	n.d.	2.17 ± 0.4	2.1 ± 0.0	1.4 ± 0.3	4.1 ± 0.9
V_Z_obs_ [L/kg]	2.3 ± 0.1	14.38 ± 4.8	11.52 ± 3.0	n.d.	0.23 ± 0.0	0.25 ± 0.0	0.43 ± 0.1	0.54 ± 0.2
Cl_obs_ [mL/min/kg] lung	36.7 ± 3.1	217 ± 84	168 ± 32	n.d.	3.78 ± 0.5	3.85 ± 0.4	5.95 ± 0.1	7.2 ± 0.7

a N.d.: could not be determined as fewer than two data points were available.

Based on lung tissue and ELF levels as well as on FRET IC_50_ in the one-digit nanomolar range, not critically impaired by lung surfactant, we selected **35** as the most promising inhibitor from the biaryl class for a focused PK study determining lung levels and tissue distribution after subcutaneous administration of 30 mg/kg (Figure 6, Table 8). We did observe that tissue concentrations in kidney and lung were mainly following the plasma concentration profile. Moreover, kidney concentrations were much higher compared to lung tissue, which could be a result of active transport into kidneys albeit no accumulation was observed. Also, urine levels showed high parent compound concentrations following the plasma kinetics. Similarly, ELF concentrations were following plasma kinetics suggesting that compound concentrations in lung tissue and ELF can be correlated with plasma kinetics. This is important as plasma can serve as a surrogate for estimating concentrations in relevant compartments. The measured *C*_max_ of 3.2 ± 0.3 μg/mL for **35** in ELF equals ∼ 8 μM, which was >500-fold above IC_50_ as it is assumed that ELF levels represent mainly unbound concentrations. Moreover, a favorable ELF/plasma ratio of 2.38 was determined providing further evidence that **35** reaches good concentrations in target compartments. However, **35** also showed high concentrations in liver, reaching a delayed *C*_max_ compared to plasma concentrations suggesting different transports. Nevertheless, liver concentrations decreased slowly so that dosing schemes would need to be designed to avoid accumulation in liver. Additional studies would be needed to reveal, if transport processes result in higher concentrations of **35** in liver and kidney.

**Figure 6:**
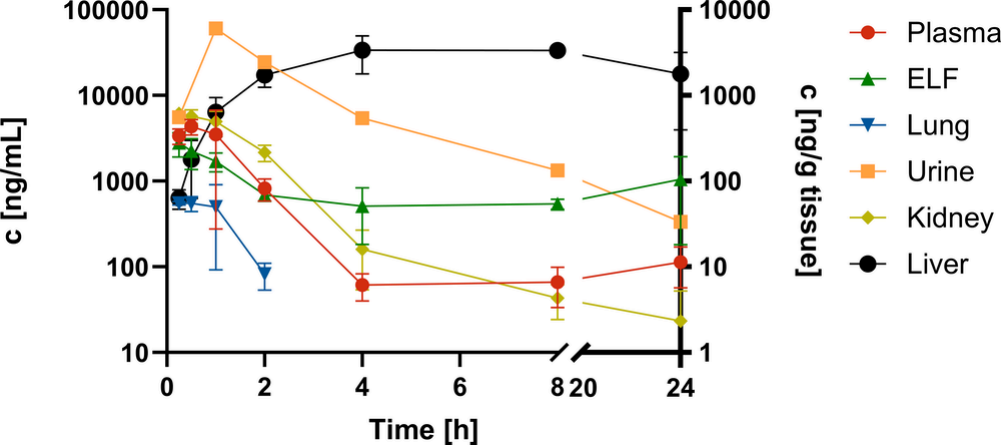
Focused pharmacokinetic (PK) study of **35** at 30 mg/kg SC. Concentrations in plasma, epithelial lining fluid (ELF) and urine are shown as well as tissue levels in lung, kidney and liver.

**Table 8:** Pharmacokinetic (PK) Parameters of **35** in a Focused PK Study Dosing 30 mg/kg SC

PK parameter	**35**
*C*_max_ [μg/mL] ELF	3.2 ± 0.3
*T*_max_ [h] ELF	0.33 ± 0.1
AUC_0‑t_ [μg/mL*h] ELF	19.2 ± 8.5
ELF/plasma ratio	2.38
*C*_max_ [μg/g] lung	0.16 ± 0.05
*T*_max_ [μg/g] lung	0.58 ± 0.4
AUC_0‑t_ [μg/g*h] lung	0.17 ± 0.07

### Advanced *In Vitro* Profiling of Frontrunner **35**

We proceeded to determine the capacity of frontrunner **35** to mitigate LasB-associated cytotoxicity, unveiling intriguing results. Our compound exhibited exceptional potency when applied to cells treated with PAO1 culture supernatant (csn), as illustrated in Figure 7, A. We observed an average cell viability of 81%, when cells encountered the challenge of LasB-deficient ΔlasB PAO1 csn. In contrast, the cell viability dropped to 11% in the presence of PAO1 csn, proving LasB is a significant extracellular virulence factor of *P. aeruginosa* PAO1, as demonstrated previously in NCI-H292, Calu-3, CFBE epithelial cells^35^ and in macrophages.^34^ In the presence of our compound, a notable enhancement in cell viability was detected, exhibiting an effect that falls between the impact of the wild-type and ΔlasB PAO1. Specifically, the utilization of 5 μM **35** resulted in nearly complete inhibition of LasB activity. These findings underscore the pronounced selectivity of our compound for LasB, demonstrating its proficiency in rescuing cells from the toxic effects of this virulence factor. Moreover, a dose-dependent pattern of LasB inhibition was evident within a low micromolar range, further highlighting the potential of **35** as a promising candidate for in-depth investigations.

**Figure 7:**
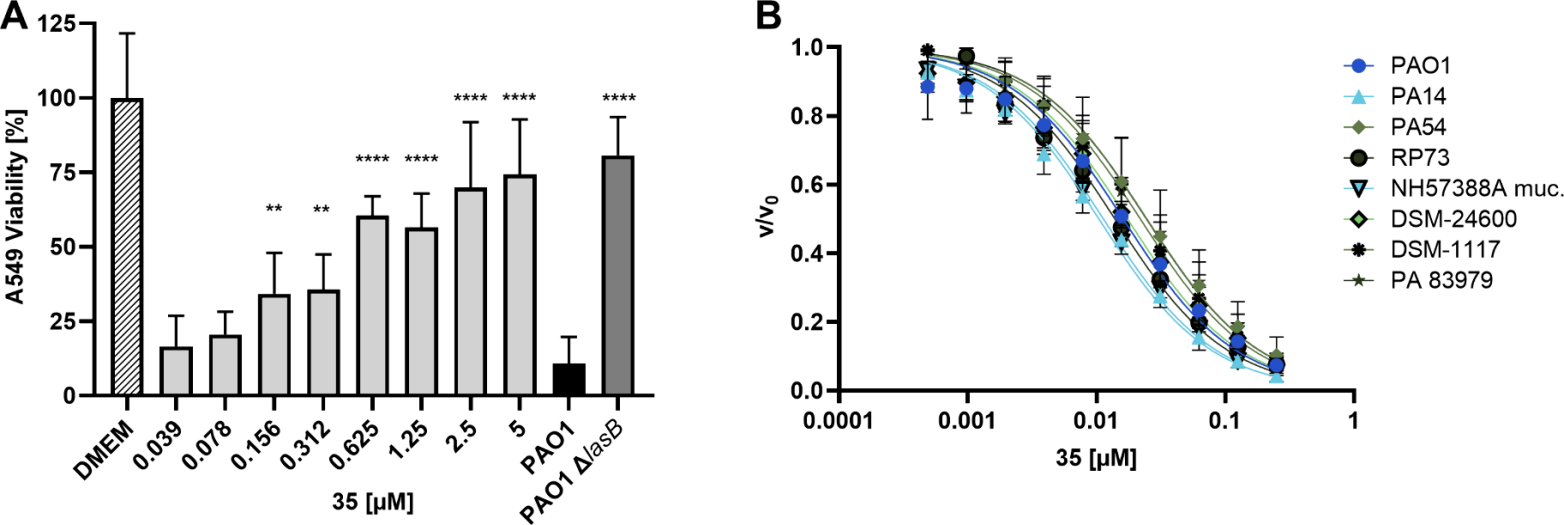
(A) Dose-dependent inhibitory impact of **35** against LasB in 10% (v/v) *Pseudomonas aeruginosa* PAO1 culture supernatant (csn). Each bar reflects the results of three separate experiments, and data are presented with standard deviation (SD). To assess statistical significance, a one-way ANOVA analysis was conducted, followed by Dunnett’s multiple comparisons test, comparing the mean value of each concentration with the mean value of PAO1 without any treatment with the compound. (**** *p* ≤ 0.0001, *** *p* ≤ 0.001, ** *p* ≤ 0.01, * *p* ≤ 0.05). (B) *In vitro* activity of **35** against supernatants generated from *P. aeruginosa* strains PAO1, PA14, PA54, RP73, NH57388A, DSM-24600, DSM-1117, and multiple drug-resistant clinical isolate 83979. IC_50_ values were determined based on a minimum of three independent experiments, each carried out in duplicate.

In a next step, we conducted a comprehensive exploration of **35**’s potential to inhibit the activity of LasB across additional *P. aeruginosa* strains, including clinical isolates by implementing the FRET-based LasB inhibition assay. Therefore, we generated supernatants from these strains and adjusted LasB concentrations by diluting them until a comparable proteolytic activity toward the FRET substrate was achieved. Importantly, the IC_50_ values determined does not vary crucially ranging from 10 to 25 nM (Table S5, Figure 7, B) and matches the *in vitro* IC_50_ values on the isolated enzyme of 15 nM very well. These results highlight that the inhibitor is indeed active against all tested and potentially additional *P. aeruginosa* strains with more clinical relevance than the lab strains PAO1 and PA14. This even includes isolates from chronic infections such as RP73 and NH57883A. Notably, the proteolytic activity in these supernatants was significantly lower (Table S5) than in the other strains, which is likely due to the known down-regulation of LasB during chronicization of infections.

## Discussion and Conclusions

In order to broaden the scope of potential application of inhibitors targeting LasB from *P. aeruginosa* , compound properties need to be tailored to the respective indication and patient population. In this study, we aimed at transferring the good retention of LasB inhibitors in the lung after pulmonary administration into favorable lung exposure after IV dosing. In this context, we performed structure-based optimization of our previously reported LasB inhibitors^19^ and improved activity via growing the LasB inhibitor deeper into the active site of the protease. The binding mode of the selected frontrunner compounds **21**, **35** and **141** was elucidated using X-ray crystallography, and the successful structure-based compound growth strategy was thereby confirmed. While some changes in the structures led to a significant drop in activity (*e.g.*, substitution in *ortho*- or *meta*-position), various changes in the molecule seem to be tolerated well by the LasB binding pocket. Additional beneficial key interactions, typical for the biaryl class – such as CH–π interactions with amino acid side-chain residues - were identified, and the binding mode of **21** to a clinically isolated LasB mutant could be elucidated. Selected inhibitors were further shown to be active against instilled LasB *in vivo*. We based the further optimization of compound pharmacokinetics on their *in vitro* ADMET profile. While metabolic stability often turns out to be a parameter that needs to be optimized in drug development,^37^ this is not applicable to the phosphonate-based LasB inhibitors as they seem to be inert toward liver metabolism. This is likely due to their high polarity as confirmed by the low logD_7.4_, rendering them soluble enough for renal excretion of the nonmetabolized compound *in vivo*. This has been observed before^19,26^ and could be confirmed in this study. Whereas PPB is generally used to assess free drug levels in order to determine doses for PD studies,^38^ we conclude from the data reported here that it is indeed a parameter that needs to be optimized toward a specific range for this distinct compound class to achieve sufficient exposure in target compartments, such as lung or ELF, after systemic dosing. To rationalize compound selection for PPB measurements from our large set of inhibitors, we employed a correlation with logD_7.4_ as a predictive tool. This reduced the need for protein-binding studies and accelerated the identification of compounds within a suitable logD_7.4_ and with this also PPB range. On top of this rather technical advantage, the *in vitro* assay-driven preselection of phosphonates for *in vivo* PK studies assessing lung permeation further reduces the number of animal studies. To what extent this approach is applicable to other compound scaffolds and whether this specific range is transferable to other species or strains of course needs to be determined individually. Here, we could identify a sweet spot of PPB between 80–95% that roughly corresponds to a logD_7.4_ range from –1 to 0.5 or clogP from 1.0 to 1.9. Accordingly, computed logP values can further be used as a parameter guiding straightforward synthetic compound optimization.

In this context, we identified surfactant protein binding to impair the activity of some inhibitors. Notably, **141** and **21**, which do bind to surfactant proteins, also show relatively high PPB > 98%, whereas **130** does not bind to surfactant proteins at all and shows only low PPB (61.7%). Thus, the interaction with surfactant can also be linked to compound polarity and optimizing the physicochemical properties of the scaffold turned out advantageous in both regards, resulting in compounds that are more likely to be available in the lung and, once there, less likely to be impaired by binding to surfactant proteins.

The advanced ADMET and IV PK profiling in this study also included representatives from previously published classes. When looking at both tissue and ELF levels, three compounds showed superior behavior: **206**, a representative from our previous monoaryl class reported by Konstantinovic *et al.* has already been shown to be efficacious in a murine lung-infection model after inhalative administration in combination with the SOC levofloxacin.^19^ Second, **212** turned out to be a frontrunner from our dipeptide class reported by Schütz *et al.* and was shown to be efficacious in combination with meropenem to treat *Pseudomonas* keratitis. The third superior compound is frontrunner **35** from this study. Its PK were investigated in detail, revealing lung exposure >500-fold above the *in vitro* IC_50_ value on LasB. The compound was further found to permeate into lung tissue and ELF with a favorable ELF/plasma ratio demonstrating good compound target exposures.

Furthermore, we could also transfer the *in vitro* activity to other *P. aeruginosa* strains with no to very little changes in IC_50_ value. This finding is not unexpected as the *lasB* gene is known to be highly conserved across strains, rendering the LasB inhibitors applicable to various clinical isolates as we have demonstrated previously^30^ and confirmed in this study via X-ray crystallography.

Taken together, our multiparameter approach combining cocrystallization-guided inhibitor optimization with ADMET-DMPK profiling provides a solid platform to advance LasB inhibitors to treatment options in different indications such as HAP/VAP.

## Experimental Section

### Chemistry

All reagents were used from commercial suppliers without further purification. Procedures were not optimized regarding yield. NMR spectra were recorded on a Bruker AV 500 (500 MHz) spectrometer. Chemical shifts are given in parts per million (ppm) and referenced against the residual proton, ^1^H, or carbon, ^13^C, resonances of the >99% deuterated solvents as internal reference. Coupling constants (*J*) are given in Hertz (Hz). Data are reported as follows: chemical shift, multiplicity, coupling constants and integration. Liquid chromatography–mass spectrometry was performed on an LC-MS system, consisting of a Dionex UltiMate 3000 pump, autosampler, column compartment and detector (Thermo Fisher Scientific, Dreieich, Germany) and ESI quadrupole MS (MSQ Plus or ISQ EC, Thermo Fisher Scientific, Dreieich, Germany). High-resolution mass was determined by LC-MS/MS using Thermo Scientific Q Exactive Focus Orbitrap LC-MS/MS system. Purity of the final compounds was determined by LC-MS using the area percentage method on the UV trace recorded at a wavelength of 254 nm and found to be >95%.

#### General Procedure A-1: Amide Coupling Using EDC·HCl Followed by Boc-Deprotection

**Step 1:** The acid (1.2–2.0 equiv) was dissolved in DCM. EDC·HCl (1.2–2.0 equiv) was added, followed by the corresponding aniline (1.0 equiv). The resultant mixture was stirred at room temperature (r.t.), until the starting aniline was consumed. The solution obtained was washed with 1 M HCl and sat. aq. NaCl solution. The organic layer was dried over anhydrous sodium sulfate, filtered and concentrated under reduced pressure to afford the crude product. The crude product obtained was either used in the next step without further purification or purified using column chromatography.

**Step 2:** Boc-protected aniline (1.0 equiv) obtained in **Step 1** was dissolved in DCM. TFA (7.0 equiv) was added, and the mixture was stirred at r.t. overnight. The mixture was evaporated to dryness. Fresh DCM was added, washed with 2 M NaOH and sat. aq. NaCl solution. Dried over anhydrous sodium sulfate, filtered and concentrated under reduced pressure to afford the crude product, which was used in the next step without purification.

#### General Procedure A-2: Amide Coupling Using EDC·HCl and HOBt

2-(Diethoxyphosphoryl)-4-methylpentanoic acid **214** (1.5 equiv) was dissolved in DCM. EDC·HCl (2.0 equiv), HOBt (2.0 equiv) and DIPEA (2.4 equiv) were added, followed by the corresponding aniline (1.0 equiv). The resultant mixture was stirred at r.t., until the starting aniline was consumed. The solution obtained was washed with 1 M HCl and sat. aq. NaCl solution. The organic layer was dried over anhydrous sodium sulfate, filtered and concentrated under reduced pressure to afford the crude product. The crude product obtained was either used in the next step without further purification or purified using column chromatography.

#### General Procedure A-3: Amide Coupling Using TBTU

2-(Diethoxyphosphoryl)-4-methylpentanoic acid **214** (1.2 equiv) was dissolved in DMF or DCM. TBTU (1.5 equiv) and NMM (2.5 equiv) were added, followed by the corresponding aniline (1.0 equiv). The resultant mixture was stirred at r.t. until the starting aniline was consumed. The solution obtained was washed with 1 M NaOH, 1 M HCl and sat. aq. NaCl solution. The organic layer was dried over anhydrous sodium sulfate, filtered and concentrated under reduced pressure to afford the crude product. The crude product obtained was either used in the next step without further purification or purified using column chromatography.

#### General Procedure B: Synthesis of Sulfonamides Followed by Boc-Deprotection

**Step 1:** *tert*-butyl (4-aminophenyl)­carbamate (1.0 equiv) was dissolved in dry DCM and cooled down to 0 °C. Et_3_N (1.2 equiv) was added, followed by the corresponding sulfonyl chloride (1.1 equiv). The ice bath was removed, and the reaction mixture stirred overnight at r.t. Solvents were evaporated, and the crude product obtained was purified using column chromatography.

**Step 2:** Boc-protected aniline (1.0 equiv) obtained in **Step 1** was dissolved in DCM. TFA (7.0 equiv) was added, and the mixture was stirred at r.t. overnight. The mixture was evaporated to dryness. Fresh DCM was added, washed with 2 M NaOH and sat. aq. NaCl solution. Dried over anhydrous sodium sulfate, filtered and concentrated under reduced pressure to afford the crude product, which was used in the next step without purification.

Alternatively, Boc-protected aniline (1.0 equiv) obtained in **Step 1** was dissolved in DCM/MeOH (1:1). 4 M HCl in dioxane (10.0 equiv) was added, and the mixture was stirred at r.t. overnight. The mixture was evaporated to dryness and used in the next step without purification.

#### General Procedure C: Synthesis of Phosphonic Acid Derivatives

**Step 1:** *N*-Aryl-2-halo-2-alkylacetamide derivative (1.0 equiv) was suspended in triethyl phosphite (10–25 equiv) and heated to 150 °C in a sealed tube for a total of 18 h (or otherwise specified). Most of unreacted triethyl phosphite was evaporated *in vacuo*, and the resultant oil was purified by column chromatography.

**Step 2:** To a solution of diethyl phosphonate (1.0 equiv) in dry DCM, bromotrimethylsilane (5.0–7.0 equiv) was added dropwise over a period of 15 min. The reaction mixture was stirred at r.t. overnight (or otherwise specified). If no full conversion was achieved, the excess of bromotrimethylsilane (5.0 equiv) was added the next day. Then, MeOH was added and stirred at r.t. for 30 min to cleave the previously formed TMS ester. Solvents were concentrated *in vacuo*, and the resultant oil was purified by preparative HPLC.

#### General Procedure D: Synthesis of Derivatives by Suzuki Coupling

To a mixture of bromo aryl (1 equiv), corresponding boronic acid (1.5 equiv) and potassium carbonate 2 M (1 mL) in a 1,4-dioxane/water mixture (4/1) (2 mL), was added [Pd­(dppf)­Cl_2_] (0.05 equiv), and the mixture was heated at 150 °C for 20 min under microwave irradiation. The reaction mixture was concentrated *in vacuo.* The reaction mixtures were diluted with water (5 mL), and the aqueous layer was extracted with DCM (3 × 15 mL). The organic layer was dried over anhydrous sodium sulfate, filtered and evaporated to dryness under reduced pressure. The product was purified by column chromatography.

#### General Procedure E: Synthesis of Nitro Derivatives

To a mixture of 1-fluoro-4-nitrobenzene (1.0 equiv) in dry NMP or DMF (10 mL), were added the corresponding aniline, phenol, or thiophenol (1.2 equiv) and potassium carbonate (1.5 equiv). The resulting suspension was stirred at 150 °C for 2–18 h. The reaction mixture was cooled to RT, poured onto ice and filtered. The product was washed with water and dried to give the title compound. The product was used in the next step without further purification.

#### General Procedure F: Reduction to Afford Amino Derivatives

A mixture of the corresponding nitro derivative (1.0 equiv), Fe (5.0 equiv) and ammonium chloride (0.5 equiv) was dissolved in an ethanol/water (2/1) mixture. The mixture was heated at 100^◦^C for 2 h. Excess ethanol was evaporated under reduced pressure and water (10 mL) was added to the remaining residue, before extraction with ethyl acetate. The organic solvent was then dried over MgSO_4_, filtered and evaporated under reduced pressure. The product was purified by column chromatography.

#### (1-{[4′-Chloro-(1,1′-biphenyl)-2-yl]­amino}-4-methyl-1-oxopentan-2-yl)­phosphonic Acid (**19**)

Compound **19** was synthesized according to general procedure C (step 2), using **13** (83 mg, 0.19 mmol), bromotrimethylsilane (250 μL, 1.9 mmol) and DCM (water (10 mL) was added). The reaction was stirred at r.t. overnight. The crude product was purified using preparative HPLC (CH_3_CN (HCOOH 0.05%)/H_2_O (HCOOH 0.05%) = 1/9 to 10/0). The product was obtained as white solid (59 mg, 81%). ^1^H NMR ((water (10 mL) was added), DMSO) δ 9.27 (s, 1H), 7.60 (d, *J* = 7.6 Hz, 1H), 7.47–7.40 (m, 4H), 7.39–7.34 (m, 1H), 7.30 (dd, *J* = 7.6, 1.8 Hz, 1H), 7.28–7.24 (m, 1H), 2.94–2.78 (m, 1H), 1.84 (ddd, *J* = 11.4, 10.7, 3.7 Hz, 1H), 1.38–1.20 (m, 2H), 0.82 (d, *J* = 6.2 Hz, 3H), 0.78 (d, *J* =water (10 mL) was added, 3H). ^13^C NMR (126 MHz, DMSO) δ 168.7 (d, *J* = 5.1 Hz), 138.0, 135.6, 135.2, 132.4, 131.4, 130.5, 128.7, 128.4, 126.8, 126.1, 45.7 (d, *J* = 127.2 Hz), 36.5 (d, *J* = 3.9 Hz), 26.6 (d, *J* = 14.9 Hz), 23.9, 21.7. ^31^P NMR (202 MHz, DMSO) δ 20.68. HRMS (ESI^–^) calculated for C_18_H_20_ClNO_4_P [M-H]^−^ 380.0824, found 380.0822.

#### (1-{[4′-Chloro-(1,1′-biphenyl)-3-yl]­amino}-4-methyl-1-oxopentan-2-yl)­phosphonic Acid (**20**)

Compound **20** was synthesized according to general procedure C (step 2), using **14** (83 mg, 0.19 mmol), bromotrimethylsilane (250 μL, 1.9 mmol) and DCM (water (10 mL) was added). The reaction was stirred at r.t. overnight. The crude product was purified using preparative HPLC (CH_3_CN (HCOOH 0.05%)/H_2_O (HCOOH 0.05%) = 1/9 to 10/0). The product was obtained as white solid (61 mg, 83%). ^1^H NMR (water (10 mL) was added, DMSO) δ ppm: 10.09 (s, 1H), 8.00 (t, *J* = 1.7 Hz, 1H), 7.66–7.60 (m, 2H), 7.59–7.51 (m, 3H), 7.40 (t, *J* = 7.9 Hz, 1H), 7.33 (d, *J* = 7.8 Hz, 1H), 3.01 (ddd, *J* = 22.4, 11.3, 2.7 Hz, 1H), 2.05–1.91 (m, 1H), 1.58–1.38 (m, 2H), 0.89 (d, *J* = 2.9 Hz, 3H), 0.87 (d, *J* = (water (10 mL) was added, 3H). ^13^C NMR (126 MHz, DMSO) δ ppm: 168.6 (d, *J* = 5.4 Hz), 140.6 (s), 139.7 (s), 139.5 (s), 132.9 (s), 129.9 (s), 129.4 (s), 128.8 (s), 121.8 (s), 118.8 (s), 117.6 (s), 46.6 (d, *J* = 126.7 Hz), 36.2 (d, *J* = 4.1 Hz), 26.9 (d, *J* = 14.8 Hz), 22.8 (d, *J* = 234.2 Hz). ^31^P NMR (water (10 mL) was added, DMSO) δ ppm: 19.81. HRMS (ESI^–^) calculated for C_18_H_20_ClNO_4_P [M–H]^−^ 380.0824, found 380.0822.

#### (1-{[4′-Chloro-(1,1′-biphenyl)-4-yl]­amino}-4-methyl-1-oxopentan-2-yl)­phosphonic Acid (**21**)

Compound **21** was synthesized according to general procedure C (step 2), using **15** (83 mg, 0.19 mmol), bromotrimethylsilane (250 μL, 1.9 mmol) and DCM (15 mL). The reaction was stirred at r.t. overnight. The crude product was purified using preparative HPLC (CH_3_CN (HCOOH 0.05%)/H_2_O (HCOOH 0.05%) = 1/9 to 10/0). The product was obtained as white solid (40 mg, 55%). ^1^H NMR (500 MHz, DMSO) δ ppm: 10.08 (s, 1H), 7.73–7.70 (m, 2H), 7.68–7.65 (m, 2H), 7.64–7.60 (m, 2H), 7.51–7.47 (m, 2H), 3.01 (ddd, *J* = 22.4, 11.3, 2.7 Hz, 1H), 2.05–1.87 (m, 1H), 1.57–1.35 (m, 2H), 0.89 (d, *J* = 2.1 Hz, 3H), 0.87 (d, *J* = 2.1 Hz, 3H). ^13^C NMR (126 MHz, DMSO) δ ppm: 168.5 (d, *J* = 4.8 Hz), 139.7, 139.0, 133.7, 132.2, 129.3, 128.4, 127.3, 119.8, 46.6 (d, *J* = 126.8 Hz), 36.2 (d, *J* = 4.0 Hz), 27.0 (d, *J* = 14.8 Hz), 23.7, 21.9. ^31^P NMR (202 MHz, DMSO) δ ppm: 19.80. HRMS (ESI^–^) calculated for C_18_H_20_ClNO_4_P [MsH]^−^ 380.0824, found 380.0822.

#### 1-(5-(4-Chlorophenyl)­pyridin-2-ylcarbamoyl)-3-methylbutylphosphonic Acid (**22**)

Compound **22** was synthesized according to general procedure C (step 2), using **16** (83 mg, 0.19 mmol), bromotrimethylsilane (250 μL, 1.9 mmol) and DCM (15 mL). The reaction was stirred at r.t. overnight. The crude product was purified using preparative HPLC (CH_3_CN (HCOOH 0.05%)/H_2_O (HCOOH 0.05%) = 1/9 to 10/0). The product was obtained as white solid (40 mg, 55%). ^1^H NMR (500 MHz, MeOD) δ ppm: 8.44 (d, *J* = 2.1 Hz, 1H), 8.07 (d, *J* = 8.6 Hz, 1H), 7.98 (dd, *J* = 8.7, 2.4 Hz, 1H), 7.54 (d, *J* = 8.5 Hz, 2H), 7.38 (d, *J* = 8.5 Hz, 2H), 3.11 (dd, *J* = 24.0, 12.8 Hz, 1H), 2.12–1.95 (m, 1H), 1.56 (dd, *J* = 10.8, 4.9 Hz, 2H), 0.88 (d, *J* = 5.3 Hz, 3H), 0.86 (d, *J* = 4.3 Hz, 3H). ^13^C NMR (126 MHz, MeOD) δ ppm: 170.1, 150.7, 144.4, 136.9, 135.6, 133.7, 131.5, 128.9, 127.8, 114.2, 35.9 (d, *J* = 3.3 Hz), 26.8 (d, *J* = 14.6 Hz), 22.2, 21.3, 20.4. ^31^P NMR (202 MHz, MeOD) δ ppm: 20.28. HRMS (ESI^–^) calculated for C_17_H_19_ClN_2_O_4_P [M-H]^−^ 381.0776, found 381.0776.

#### 1-[6-(4-Chlorophenyl)­pyridin-3-ylcarbamoyl]-3-methylbutylphosphonic Acid (**23**)

Compound **23** was synthesized according to general procedure C (step 2), using **17** (83 mg, 0.19 mmol), bromotrimethylsilane (250 μL, 1.9 mmol) and DCM (15 mL). The reaction was stirred at r.t. overnight. The crude product was purified using preparative HPLC (CH_3_CN (HCOOH 0.05%)/H_2_O (HCOOH 0.05%) = 1/9 to 10/0). The product was obtained as white solid (53 mg, 73%). ^1^H NMR (500 MHz, DMSO) δ ppm: 10.36 (s, 1H), 8.84 (d, *J* = 2.4 Hz, 1H), 8.17 (dd, *J* = 8.7, 2.5 Hz, 1H), 8.09–8.03 (m, 2H), 7.94 (d, *J* = 8.7 Hz, 1H), 7.70–7.40 (m, 2H), 3.04 (ddd, *J* = 22.4, 11.2, 2.4 Hz, 1H), 2.04–1.93 (m, 1H), 1.57–1.39 (m, 2H), 0.89 (s, 3H), 0.88 (s, 3H). ^13^C NMR (126 MHz, DMSO) δ ppm: 169.1 (d, *J* = 4.8 Hz), 149.6, 140.7, 137.7, 135.9, 133.7, 129.2, 128.2, 127.1, 120.6, 46.6 (d, *J* = 126.0 Hz), 36.1 (d, *J* = 3.9 Hz), 27.0 (d, *J* = 14.4 Hz), 23.6, 21.8. ^31^P NMR (202 MHz, DMSO) δ ppm: 19.26. HRMS (ESI^–^) calculated for C_17_H_19_ClN_2_O_4_P [M-H]^−^ 381.0776, found 381.0776.

#### 1-[2-(4-Chlorophenyl)­pyrimidin-5-ylcarbamoyl]-3-methylbutylphosphonic Acid (24)

Compound **24** was synthesized according to general procedure C (step 2), using **18** (83 mg, 0.19 mmol), bromotrimethylsilane (250 μL, 1.9 mmol) and DCM (15 mL). The reaction was stirred at r.t. overnight. The crude product was purified using preparative HPLC (CH_3_CN (HCOOH 0.05%)/H_2_O (HCOOH 0.05%) = 1/9 to 10/0). The product was obtained as white solid (22 mg, 31%). ^1^H NMR (500 MHz, DMSO) δ ppm: 10.59 (s, 1H), 9.12 (s, 2H), 8.48–8.08 (m, 2H), 7.69–7.27 (m, 2H), 3.06 (ddd, *J* = 22.3, 11.0, 2.2 Hz, 1H), 2.01 (ddd, *J* = 15.3, 10.1, 3.7 Hz, 1H), 1.59–1.32 (m, 2H), 0.88 (d, *J* = 6.2 Hz, 6H). ^13^C NMR (126 MHz, DMSO) δ ppm: 169.4 (d, *J* = 4.8 Hz), 157.4, 147.7, 136.3, 135.5, 133.6, 129.4, 129.2, 46.6 (d, *J* = 125.5 Hz), 36.1 (d, *J* = 3.9 Hz), 26.9 (d, *J* = 14.4 Hz), 23.6, 21.8. ^31^P NMR (202 MHz, DMSO) δ ppm: 18.74. HRMS (ESI^–^) calculated for C_16_H_18_ClN_3_O_4_P [M-H]^−^ 382.0729, found 382.0727.

#### 1-[2-(4-Isopropoxyphenyl)­pyrimidin-5-ylcarbamoyl-3-methylbutylphosphonic Acid (**35**)

Compound **35** was synthesized according to general procedure C (step 2), using **33** (88 mg, 0.19 mmol), bromotrimethylsilane (250 μL, 1.9 mmol) and DCM (15 mL). The reaction was stirred at r.t. overnight. The crude product was purified using preparative HPLC (CH_3_CN (HCOOH 0.05%)/H_2_O (HCOOH 0.05%) = 1/9 to 10/0). The product was obtained as white solid (46 mg, 60%). ^1^H NMR (500 MHz, DMSO) δ ppm: 10.46 (s, 1H), 9.05 (s, 2H), 8.24 (d, *J* = 8.9 Hz, 2H), 7.01 (d, *J* = 8.9 Hz, 2H), 4.70 (dt, *J* = 12.1, 6.0 Hz, 1H), 3.04 (ddd, *J* = 22.4, 11.1, 2.3 Hz, 1H), 2.00 (ddd, *J* = 15.4, 10.0, 3.7 Hz, 1H), 1.61–1.35 (m, 2H), 1.30 (d, *J* = 6.0 Hz, 6H), 0.88 (d, *J* = 6.3 Hz, 6H). ^13^C NMR (126 MHz, DMSO) δ ppm: 169.2 (d, *J* = 4.7 Hz), 159.8, 158.5, 147.8, 132.7, 129.7, 129.4, 115.9, 69.8, 46.6 (d, *J* = 125.6 Hz), 36.1 (d, *J* = 3.9 Hz), 26.9 (d, *J* = 14.5 Hz), 23.6, 22.3, 21.8. ^31^P NMR (202 MHz, DMSO) δ ppm: 18.90. HRMS (ESI^–^) calculated for C_19_H_25_N_3_O_5_P [M-H]^−^ 406.1537, found 406.1533.

#### 1-[2-(3-Chloro-4-isopropoxyphenyl)­pyrimidin-5-ylcarbamoyl]-3-methylbutylphosphonic Acid (**36**)

Compound **36** was synthesized according to general procedure C (step 2), using **34** (95 mg, 0.19 mmol), bromotrimethylsilane (250 μL, 1.9 mmol) and DCM (15 mL). The reaction was stirred at r.t. overnight. The crude product was purified using preparative HPLC (CH_3_CN (HCOOH 0.05%)/H_2_O (HCOOH 0.05%) = 1/9 to 10/0). The product was obtained as a white solid (43 mg, 51%). ^1^H NMR (500 MHz, DMSO) δ ppm: 10.54 (s, 1H), 9.07 (s, 2H), 8.29 (d, *J* = 2.1 Hz, 1H), 8.22 (dd, *J* = 8.7, 2.1 Hz, 1H), 7.28 (d, *J* = 8.9 Hz, 1H), 4.78 (dt, *J* = 12.1, 6.0 Hz, 1H), 3.04 (ddd, *J* = 22.4, 11.1, 2.3 Hz, 1H), 2.00 (ddd, *J* = 15.3, 10.2, 3.7 Hz, 1H), 1.56–1.41 (m, 2H), 1.34 (d, *J* = 6.0 Hz, 6H), 0.88 (d, *J* = 6.2 Hz, 6H). ^13^C NMR (126 MHz, DMSO) δ ppm: 169.3 (d, *J* = 5.6 Hz), 157.2, 155.0, 147.7, 133.2, 130.7, 129.1, 127.7, 123.0, 115.6, 71.7, 46.6 (d, *J* = 125.5 Hz), 36.1 (d, *J* = 4.1 Hz), 26.9 (d, *J* = 14.4 Hz), 23.6, 22.2, 21.8. ^31^P NMR (202 MHz, DMSO) δ ppm: 18.79. HRMS (ESI^–^) calculated for C_19_H_24_ClN_3_O_5_P [M–H]^−^ 440.1147, found 440.1144.

#### 1-(4-Morpholinophenylcarbamoyl)-3-methylbutylphosphonic Acid (**80**)

Compound **80** was synthesized according to general procedure C (step 2), using **66** (78 mg, 0.19 mmol), bromotrimethylsilane (250 μL, 1.9 mmol) and DCM (15 mL). The reaction was stirred at r.t. overnight. The crude product was purified using preparative HPLC (CH_3_CN (HCOOH 0.05%)/H_2_O (HCOOH 0.05%) = 0.5/9.5 to 10/0). The product was obtained as a white solid (21 mg, 32%). ^1^H NMR (500 MHz, DMSO) δ ppm: 9.74 (s, 1H), 7.47 (d, *J* = 9.0 Hz, 2H), 6.88 (d, *J* = 9.1 Hz, 2H), 3.79–3.65 (m, 4H), 3.08–2.98 (m, 4H), 2.93 (ddd, *J* = 22.3, 11.3, 2.8 Hz, 1H), 2.01–1.88 (m, 1H), 1.54–1.35 (m, 2H), 0.87 (d, *J* = 1.0 Hz, 3H), 0.86 (d, *J* = 1.0 Hz, 3H). ^13^C NMR (126 MHz, DMSO) δ ppm: 167.6 (d, *J* = 4.7 Hz), 147.5, 132.4, 120.6, 115.9, 66.6, 49.6, 46.3 (d, *J* = 127.5 Hz), 36.3 (d, *J* = 4.1 Hz), 28.0–26.0 (m), 23.7, 21.8. ^31^P NMR (202 MHz, DMSO) δ ppm: 20.49. HRMS (ESI^–^) calculated for C_16_H_24_N_2_O_5_P [M-H]^−^ 355.1428, found 355.1426.

#### 1-[4-(Piperazin-1-yl)­phenylcarbamoyl]-3-methylbutylphosphonic Acid (**81**)

Compound **81** was synthesized according to general procedure C (step 2), using **67** (97 mg, 0.19 mmol), bromotrimethylsilane (250 μL, 1.9 mmol) and DCM (15 mL). The reaction was stirred at r.t. overnight. The crude product was purified using preparative HPLC (CH_3_CN (HCOOH 0.05%)/H_2_O (HCOOH 0.05%) = 0.5/9.5 to 10/0). The product was obtained as a white solid (23 mg, 35%). ^1^H NMR (500 MHz, D_2_O) δ ppm: 7.33 (d, *J* = 8.8 Hz, 2H), 7.01 (d, *J* = 8.5 Hz, 2H), 3.32 (s, 8H), 2.82 (ddd, *J* = 13.5, 12.3, 2.2 Hz, 1H), 1.90 (ddd, *J* = 11.8, 9.3, 6.4 Hz, 1H), 1.47 (dd, *J* = 26.5, 6.9 Hz, 2H), 0.84 (d, *J* = 5.7 Hz, 6H). ^13^C NMR (126 MHz, D_2_O) δ ppm: 146.9 (d, *J* = 5.1 Hz), 131.5, 124.1, 123.4, 118.2, 47.0, 47.4 (d, *J* = 129.9 Hz), 43.0, 36.4 (d, *J* = 4.4 Hz), 26.8 (d, *J* = 14.3 Hz), 22.5, 20.6. ^31^P NMR (202 MHz, D_2_O) δ ppm: 17.60. HRMS (ESI^–^) calculated for C_16_H_25_N_3_O_4_P [M-H]^−^ 354.1588, found 354.1586.

#### 1-(4-Morpholinophenylcarbamoyl)-3-methylbutylphosphonic Acid (**82**)

Compound **82** was synthesized according to general procedure C (step 2), using **68** (80 mg, 0.19 mmol), bromotrimethylsilane (250 μL, 1.9 mmol) and DCM (15 mL). The reaction was stirred at r.t. overnight. The crude product was purified using preparative HPLC (CH_3_CN (HCOOH 0.05%)/H_2_O (HCOOH 0.05%) = 0.5/9.5 to 10/0). The product was obtained as a white solid (28 mg, 41%). ^1^H NMR (500 MHz, D_2_O) δ ppm: 7.41 (d, *J* = 8.9 Hz, 2H), 7.07 (d, *J* = 9.0 Hz, 2H), 3.76 (d, *J* = 11.5 Hz, 2H), 3.62 (d, *J* = 9.6 Hz, 2H), 3.21–3.23 (m, 2H), 3.10 (d, *J* = 11.0 Hz, 2H), 2.94 (s, 3H), 2.93–2.84 (m, 1H), 1.98 (ddd, *J* = 11.9, 9.3, 6.2 Hz, 1H), 1.63–1.47 (m, 2H), 0.92 (d, *J* = 6.0 Hz, 6H). ^13^C NMR (126 MHz, D_2_O) δ ppm: 167.7 (d, J = 5.5 Hz), 120.6, 116.8, 50.3, 46.4 (d, J = 127.1 Hz), 36.3 (d, J = 4.0 Hz), 33.9, 30.5, 26.9 (d, J = 15.0 Hz), 23.7, 22.3, 21.9. ^31^P NMR (202 MHz, D_2_O) δ ppm: 17.67. HRMS (ESI^–^) calculated for C_17_H_27_N_3_O_4_P [M-H]^−^ 368.1745, found 368.1740.

#### 1-[4-(4-Acetylpiperazin-1-yl)­phenylcarbamoyl]-3-methylbutylphosphonic Acid (**83**)

Compound **83** was synthesized according to general procedure C (step 2), using **69** (86 mg, 0.19 mmol), bromotrimethylsilane (250 μL, 1.9 mmol) and DCM (15 mL). The reaction was stirred at r.t. overnight. The crude product was purified using preparative HPLC (CH_3_CN (HCOOH 0.05%)/H_2_O (HCOOH 0.05%) = 0.5/9.5 to 10/0). The product was obtained as white solid (22 mg, 29%). ^1^H NMR (500 MHz, DMSO) δ ppm: 9.73 (s, 1H), 7.47 (d, *J* = 9.0 Hz, 2H), 6.90 (d, *J* = 9.1 Hz, 2H), 3.57 (dd, *J* = 10.2, 5.4 Hz, 4H), 3.12–3.05 (m, 2H), 3.03–2.98 (m, 2H), 2.92 (ddd, *J* = 22.3, 11.3, 2.8 Hz, 1H), 2.04 (s, 3H), 1.95 (tdd, *J* = 11.8, 7.4, 4.3 Hz, 1H), 1.53–1.32 (m, 2H), 0.87 (d, *J* = 6.1 Hz, 6H). ^13^C NMR (126 MHz, DMSO) δ ppm: 168.7, 167.7 (d, *J* = 5.0 Hz), 147.2, 132.7, 120.6, 116.8 (s), 49.7 (d, *J* = 59.2 Hz), 46.8, 46.0, 45.8, 41.1, 36.3 (d, *J* = 4.0 Hz), 26.9 (d, *J* = 15.0 Hz), 23.7, 21.8, 21.7. ^31^P NMR (202 MHz, DMSO) δ ppm: 20.32. HRMS (ESI^–^) calculated for C_18_H_27_N_3_O_5_P [M-H]^−^ 396.1694, found 396.1687.

#### 1-[4-(4-Methylpiperidin-1-yl)­phenylcarbamoyl]-3-methylbutylphosphonic Acid (**84**)

Compound **84** was synthesized according to general procedure C (step 2), using **70** (80 mg, 0.19 mmol), bromotrimethylsilane (250 μL, 1.9 mmol) and DCM (15 mL). The reaction was stirred at r.t. overnight. The crude product was purified using preparative HPLC (CH_3_CN (HCOOH 0.05%)/H_2_O (HCOOH 0.05%) = 0.5/9.5 to 10/0). The product was obtained as white solid (15 mg, 22%). ^1^H NMR (500 MHz, D_2_O) δ ppm: 7.41 (d, *J* = 8.9 Hz, 2H), 7.07 (d, *J* = 9.0 Hz, 2H), 3.76 (d, *J* = 11.5 Hz, 2H), 3.62 (d, *J* = 9.6 Hz, 2H), 3.21–3.23 (m, 2H), 3.10 (d, *J* = 11.0 Hz, 2H), 2.94 (s, 3H), 2.93–2.84 (m, 1H), 1.98 (ddd, *J* = 11.9, 9.3, 6.2 Hz, 1H), 1.63–1.47 (m, 2H), 0.92 (d, *J* = 6.0 Hz, 6H). ^13^C NMR (126 MHz, D_2_O) δ ppm: 172.6 (d, *J* = 4.5 Hz), 146.3, 131.4, 123.4, 118.1, 52.9, 47.4 (d, *J* = 121.2 Hz), 47.2, 42.8, 36.3 (d, *J* = 3.9 Hz), 26.8 (d, *J* = 14.4 Hz), 22.5, 20.6. ^31^P NMR (202 MHz, D_2_O) δ ppm: 17.67. HRMS (ESI^–^) calculated for C_18_H_28_N_2_O_4_P [M-H]^−^ 367.1792, found 367.1791.

#### 1-[4-(1*H*-Imidazol-1-yl)­phenylcarbamoyl]-3-methylbutylphosphonic Acid (**85**)

Compound **85** was synthesized according to general procedure C (step 2), using **71** (75 mg, 0.19 mmol), bromotrimethylsilane (250 μL, 1.9 mmol) and DCM (15 mL). The reaction was stirred at r.t. overnight. The crude product was purified using preparative HPLC (CH_3_CN (HCOOH 0.05%)/H_2_O (HCOOH 0.05%) = 0.5/9.5 to 10/0). The product was obtained as a white solid (18 mg, 28%). ^1^H NMR (500 MHz, D_2_O) δ ppm: 8.85 (s, 1H), 7.66 (s, 1H), 7.52 (d, *J* = 8.7 Hz, 2H), 7.43 (d, *J* = 8.7 Hz, 3H), 2.84 (dd, *J* = 21.3, 10.9 Hz, 1H), 1.87 (dt, *J* = 11.6, 7.6 Hz, 1H), 1.44 (dd, *J* = 11.1, 5.5 Hz, 2H), 0.78 (d, *J* = 5.9 Hz, 6H). ^13^C NMR (126 MHz, D_2_O) δ ppm: 172.8 (d, *J* = 5.0 Hz), 138.6, 133.5, 131.2, 122.9, 122.4, 121.1, 120.7, 47.6 (d, *J* = 120.7 Hz), 36.2 (d, *J* = 4.6 Hz), 26.8 (d, *J* = 14.3 Hz), 22.5, 20.6. ^31^P NMR (202 MHz, D_2_O) δ ppm: 17.41. HRMS (ESI^–^) calculated for C_15_H_19_N_3_O_4_P [M-H]^−^ 336.1119, found 336.1117.

#### 1–4-(1*H*-Pyrazol-1-yl)­phenylcarbamoyl]-3-methylbutylphosphonic Acid (**86**)

Compound **86** was synthesized according to general procedure C (step 2), using **72** (75 mg, 0.19 mmol), bromotrimethylsilane (250 μL, 1.9 mmol) and DCM (15 mL). The reaction was stirred at r.t. overnight. The crude product was purified using preparative HPLC (CH_3_CN (HCOOH 0.05%)/H_2_O (HCOOH 0.05%) = 0.5/9.5 to 10/0). The product was obtained as a white solid (12 mg, 19%). ^1^H NMR (500 MHz, DMSO) δ ppm: 10.10 (s, 1H), 8.40 (d, *J* = 2.4 Hz, 1H), 7.77–7.74 (m, 2H), 7.73–7.71 (m, 2H), 7.70 (d, *J* = 1.6 Hz, 1H), 6.51–6.49 (m, 1H), 3.00 (ddd, *J* = 22.5, 11.3, 2.7 Hz, 1H), 2.03–1.94 (m, 1H), 1.57–1.38 (m, 2H), 0.88 (d, *J* = 1.7 Hz, 3H), 0.87 (d, *J* = 1.8 Hz, 3H). ^13^C NMR (126 MHz, DMSO) δ ppm: 168.3 (d, *J* = 5.0 Hz), 141.0, 138.1, 135.5, 127.8, 120.2, 119.2, 108.0, 46.5 (d, *J* = 127.0 Hz), 36.2 (d, *J* = 3.9 Hz), 26.9 (d, *J* = 14.7 Hz), 23.7, 21.8. ^31^P NMR (202 MHz, DMSO) δ ppm: 20.00. HRMS (ESI^–^) calculated for C_15_H_19_N_3_O_4_P [M-H]^−^ 336.1119, found 336.1116.

#### 1-[4-(2*H*-1,2,3-Triazol-1-yl)­phenylcarbamoyl]-3-methylbutylphosphonic Acid (**87**)

Compound **87** was synthesized according to general procedure C (step 2), using **73** (75 mg, 0.19 mmol), bromotrimethylsilane (250 μL, 1.9 mmol) and DCM (15 mL). The reaction was stirred at r.t. overnight. The crude product was purified using preparative HPLC (CH_3_CN (HCOOH 0.05%)/H_2_O (HCOOH 0.05%) = 0.5/9.5 to 10/0). The product was obtained as a white solid (11 mg, 17%). ^1^H NMR (500 MHz, DMSO) δ ppm: 10.20 (s, 1H), 8.08 (s, 2H), 7.95 (d, *J* = 9.0 Hz, 2H), 7.80 (d, *J* = 9.0 Hz, 2H), 3.02 (ddd, *J* = 22.5, 11.3, 2.6 Hz, 1H), 1.99 (tdd, *J* = 11.7, 7.3, 4.2 Hz, 1H), 1.47 (qdd, *J* = 12.6, 9.7, 4.7 Hz, 2H), 0.88 (d, *J* = 5.9 Hz, 6H). ^13^C NMR (126 MHz, DMSO) δ ppm: 168.6 (d, *J* = 4.8 Hz), 139.4, 136.5, 134.9, 120.2, 119.4, 46.6 (d, *J* = 126.6 Hz), 36.2 (d, *J* = 3.9 Hz), 27.0 (d, *J* = 14.7 Hz), 23.7, 21.8. ^31^P NMR (202 MHz, DMSO) δ ppm: 19.65. HRMS (ESI^–^) calculated for C_14_H_18_N_4_O_4_P [M-H]^−^ 337.1071, found 337.1070.

#### 1-[4-(1*H*-1,2,4-Triazol-1-yl)­phenylcarbamoyl]-3-methylbutylphosphonic Acid (**88**)

Compound **88** was synthesized according to general procedure C (step 2), using **74** (75 mg, 0.19 mmol), bromotrimethylsilane (250 μL, 1.9 mmol) and DCM (15 mL). The reaction was stirred at r.t. overnight. The crude product was purified using preparative HPLC (CH_3_CN (HCOOH 0.05%)/H_2_O (HCOOH 0.05%) = 0.5/9.5 to 10/0). The product was obtained as white solid (22 mg, 34%). ^1^H NMR (500 MHz, DMSO) δ ppm: 10.25 (s, 1H), 9.26 (s, 1H), 8.27 (s, 1H), 7.84 (s, 4H), 3.07 (ddd, *J* = 22.5, 11.2, 2.6 Hz, 1H), 2.05 (tdd, *J* = 11.7, 7.3, 4.1 Hz, 1H), 1.65–1.40 (m, 2H), 0.94 (d, *J* = 5.5 Hz, 6H). ^13^C NMR (126 MHz, DMSO) δ ppm: 168.6 (d, *J* = 4.7 Hz), 152.7, 142.4, 139.4, 132.3, 120.4, 120.2, 46.6 (d, *J* = 126.7 Hz), 36.2 (d, *J* = 4.0 Hz), 27.0 (d, *J* = 14.7 Hz), 23.7, 21.8. ^31^P NMR (202 MHz, DMSO) δ ppm: 19.61. HRMS (ESI^–^) calculated for C_14_H_18_N_4_O_4_P [M-H]^−^ 337.1071, found 337.1070.

#### (1-{[4-(3,4-Dichlorobenzamido)­phenyl]­amino}-4-methyl-1-oxopentan-2-yl)­phosphonic Acid (**193**)

Compound **193** was synthesized over two steps according to the general procedure C using **184** (209 mg, 0.46 mmol) and triethyl phosphite (2 mL). The resultant oil was purified by column chromatography (Hex/EtOAc = 1/1 to 25/75) to give diethyl phosphonate as a white solid (47.6 mg, 20%). The product obtained was then treated with bromotrimethylsilane (80 μL, 0.60 mmol) in DCM (2 mL). TMS ester was cleaved using MeOH (2 mL). Solvents were concentrated *in vacuo*, and the resultant oil was purified by preparative HPLC (CH_3_CN (HCOOH 0.05%)/H_2_O (HCOOH 0.05%) = 1/9 to 10/0). The product was obtained as white solid (25 mg, 63%; 13% over two steps). ^1^H NMR (500 MHz, DMSO) δ 10.36 (s, 1H), 9.99 (s, 1H), 8.21 (d, *J* = 2.0 Hz, 1H), 7.93 (dd, *J* = 8.4, 2.0 Hz, 1H), 7.82 (d, *J* = 8.4 Hz, 1H), 7.66 (d, *J* = 9.0 Hz, 2H), 7.59 (d, *J* = 9.0 Hz, 2H), 2.97 (ddd, *J* = 22.3, 11.2, 2.3 Hz, 1H), 2.02–1.91 (m, 1H), 1.55–1.35 (m, 2H), 0.87 (d, *J* = 6.3 Hz, 6H). ^13^C NMR (126 MHz, DMSO) δ 167.8, 162.9, 135.8, 135.3, 134.3, 133.9, 131.3, 130.8, 129.6, 128.1, 120.9, 119.2, 46.0 (d, *J* = 127.0 Hz), 35.8 (d, *J* = 3.7 Hz), 26.5 (d, *J* = 14.8 Hz), 23.3, 21.4. ^31^P NMR (202 MHz, DMSO) δ 19.94. HRMS (ESI^–^) calculated for C_19_H_20_Cl_2_N_2_O_5_P^–^ [M–H]^−^ 457.0492, found 457.0497.

#### (4-Methyl-1-oxo-1-{[4-(thiophene-2-carboxamido)­phenyl]­amino}­pentan-2-yl)­phosphonic Acid (**196**)

Compound **196** was synthesized over two steps according to the general procedure C, using **187** (147 mg, 0.37 mmol) and triethyl phosphite (2 mL). The resultant oil was purified by column chromatography (Hex/EtOAc = 1/1 to EtOAc) to give diethyl phosphonate as white solid (137.2 mg, 82%). The product obtained (135 mg, 0.30 mmol) was then treated with bromotrimethylsilane (275 μL, 2.10 mmol) in DCM (5 mL). The TMS ester was cleaved using MeOH (5 mL). Solvents were concentrated *in vacuo*, and the resultant oil was purified by preparative HPLC (CH_3_CN (HCOOH 0.05%)/H_2_O (HCOOH 0.05%) = 1/9 to 10/0). The product was obtained as a white solid (36.5 mg, 31%; 25% over two steps). ^1^H NMR (500 MHz, DMSO) δ 10.19 (s, 1H), 9.95 (s, 1H), 8.00 (dd, *J* = 3.8, 0.9 Hz, 1H), 7.84 (dd, *J* = 5.0, 0.9 Hz, 1H), 7.65–7.53 (m, 4H), 7.21 (dd, *J* = 4.9, 3.8 Hz, 1H), 2.97 (ddd, *J* = 22.4, 11.3, 2.5 Hz, 1H), 2.02–1.92 (m, 1H), 1.54–1.33 (m, 2H), 0.87 (d, *J* = 6.1 Hz, 6H). ^13^C NMR (126 MHz, DMSO) δ 167.7 (d, *J* = 4.3 Hz), 159.7, 140.2, 135.5, 133.8, 131.8, 128.9, 128.2, 120.9, 119.3, 46.0 (d, *J* = 127.1 Hz), 35.8, 26.5 (d, *J* = 15.0 Hz), 23.3, 21.4. ^31^P NMR (202 MHz, DMSO) δ 20.02. HRMS (ESI^–^) calculated for C_17_H_20_N_2_O_5_PS^–^ [M-H]^−^ 395.0836, found 395.0840.

#### (1-{[3-(3,4-Dichlorobenzamido)­phenyl]­amino}-4-methyl-1-oxopentan-2-yl)­phosphonic Acid (**204**)

Compound **204** was synthesized according to the general procedure C (Step 2), using **202** (88 mg, 0.17 mmol) and bromotrimethylsilane (112 μL, 0,85 mmol) in DCM (5 mL). The TMS ester was cleaved using MeOH (5 mL). Solvents were concentrated *in vacuo*, and the resultant oil was purified by preparative HPLC (CH_3_CN (HCOOH 0.05%)/H_2_O (HCOOH 0.05%) = 0.5/9.5 to 10/0). The product was obtained as a colorless solid (26 mg, 33%). ^1^H NMR (500 MHz, DMSO) δ10.39 (s, 1H), 10.01 (s, 1H), 8.22 (d, *J* = 2.1 Hz, 1H), 8.12 (t, *J* = 2.1 Hz, 1H), 7.94 (dd, *J* = 8.4, 2.1 Hz, 1H), 7.81 (d, *J* = 8.4 Hz, 1H), 7.39 (ddd, *J* = 14.5, 7.6 Hz, 1.9, 2H), 7.25 (t, *J* = 8.1 Hz, 1H), 3.02 (ddd, *J* = 22.5, 11.3, 2.8 Hz, 1H), 1.97 (tq, *J* = 11.9, 7.0, 5.4 Hz, 1H), 1.46 (dddd, *J* = 32.3, 16.5, 7.7, 4.6 Hz, 2H), 0.87 (dd, *J* = 6.4, 1.8 Hz, 6H). ^13^C NMR (126 MHz, DMSO) δ 167.9 (d, *J* = 6.7 Hz), 163.1, 139.7, 138.9, 135.2, 134.4, 131.3, 130.8, 129.7, 128.7, 128.1, 115.3, 115.0, 111.5, 46.0 (d, *J* = 126.7 Hz), 35.8 (d, *J* = 4.4 Hz), 26.5 (d, *J* = 15.0 Hz), 23.3, 21.4. ^31^P NMR (202 MHz, DMSO) δ19.95. HRMS (ESI^–^) calculated for C_19_H_20_Cl_2_N_2_O_5_P^–^ [M-H]^−^ 457.0492, found 457.0487.

#### (1-{[4-(Isonicotinamido)­phenyl]­amino}-4-methyl-1-oxopentan-2-yl)­phosphonic Acid (**130**)

Compound **130** was synthesized according to the general procedure C (Step 2), using **112** (102 mg, 0.23 mmol) and bromotrimethylsilane (150 μL, 1.14 mmol) in DCM (4 mL). The TMS ester was cleaved using MeOH (4 mL). Solvents were concentrated *in vacuo*, and the resultant oil was purified by preparative HPLC (CH_3_CN (HCOOH 0.05%)/H_2_O (HCOOH 0.05%) = 1/9 to 10/0). The product was obtained as a yellow solid (25.5 mg, 28%). ^1^H NMR (500 MHz, DMSO) δ 10.45 (s, 1H), 9.96 (s, 1H), 8.77 (dd, *J* = 4.5, 1.6 Hz, 2H), 7.85 (dd, *J* = 4.5, 1.6 Hz, 2H), 7.67 (d, *J* = 9.0 Hz, 2H), 7.64–7.54 (m, 2H), 2.97 (ddd, *J* = 22.4, 11.3, 2.8 Hz, 1H), 1.97 (tdd, *J* = 11.7, 7.3, 4.2 Hz, 1H), 1.55–1.37 (m, 2H), 0.91–0.85 (m, 6H). ^13^C NMR (126 MHz, DMSO) δ 167.7 (d, *J* = 4.6 Hz), 163.6, 150.3, 142.0, 135.9, 133.7, 121.6, 120.9, 119.3, 46.0 (d, *J* = 126.9 Hz), 35.8 (d, *J* = 3.7 Hz), 26.5 (d, *J* = 14.7 Hz), 23.3, 21.4. ^31^P NMR (202 MHz, DMSO) δ 19.92. HRMS (ESI^–^) calculated for C_18_H_21_N_3_O_5_P^–^ [M-H]^−^ 390.1224, found 390.1218.

#### (1-{[4-(4,5-Dichloroisothiazole-3-carboxamido)­phenyl]­amino}-4-methyl-1-oxopentan-2-yl)­phosphonic Acid (**131**)

Compound **131** was synthesized according to the general procedure C (Step 2), using **113** (168 mg, 0.32 mmol) and bromotrimethylsilane (211 μL, 0.85 mmol) in DCM (6 mL). The TMS ester was cleaved using MeOH (6 mL). Solvents were concentrated *in vacuo*, and the resultant oil was purified by preparative HPLC (CH_3_CN (HCOOH 0.05%)/H_2_O (HCOOH 0.05%) = 0.5/9.5 to 10/0). The product was obtained as a colorless solid (38 mg, 25%). ^1^H NMR (500 MHz, DMSO) δ 10.71 (s, 1H), 9.99 (s, 1H), 7.69–7.64 (m, 2H), 7.62–7.58 (m, 2H), 2.97 (ddd, *J* = 22.4, 11.3, 2.9 Hz, 1H), 2.02–1.91 (m, 1H), 1.55–1.37 (m, 2H), 0.87 (d, *J* = 6.3 Hz, 6H). ^13^C NMR (126 MHz, DMSO) δ 167.8 (d, *J* = 6.6 Hz), 158.6, 157.8, 149.6, 136.1, 133.1, 122.8, 120.6, 119.4, 46.1 (d, *J* = 127.0 Hz), 35.8 (d, *J* = 2.5 Hz), 26.5 (d, *J* = 14.7 Hz), 23.3, 21.4. ^31^P NMR (202 MHz, DMSO) δ 19.85. HRMS (ESI^–^) calculated for C_16_H_17_Cl_2_N_3_O_5_PS^–^ [M-H]^−^ 464.0009, found 464.0000.

#### (1-{[4-(5-Chlorothiophene-2-carboxamido)­phenyl]­amino}-4-methyl-1-oxopentan-2-yl)­phosphonic Acid (**132**)

Compound **132** was synthesized according to the general procedure C (Step 2), using **114** (107 mg, 0.22 mmol) and bromotrimethylsilane (145 μL, 1.1 mmol) in DCM (5 mL). The TMS ester was cleaved using MeOH (5 mL). Solvents were concentrated *in vacuo*, and the resultant oil was purified by preparative HPLC (CH_3_CN (HCOOH 0.05%)/H_2_O (HCOOH 0.05%) = 0.5/9.5 to 10/0). The product was obtained as a colorless solid (66 mg, 68%). ^1^H NMR (500 MHz, DMSO) δ 10.25 (s, 1H), 9.96 (s, 1H), 7.89 (d, *J* = 4.1 Hz, 1H), 7.59 (s, 4H), 7.26 (d, *J* = 4.1 Hz, 1H), 2.97 (ddd, *J* = 22.3, 11.3, 2.9 Hz, 1H), 2.02–1.89 (m, 1H), 1.46 (dddd, *J* = 32.9, 16.5, 7.3, 4.2 Hz, 2H), 0.87 (d, *J* = 6.4 Hz, 6H). ^13^C NMR (126 MHz, DMSO) δ 167.7 (d, *J* = 4.6 Hz), 158.5, 139.3, 135.7, 133.7, 133.4, 128.9, 128.3, 120.9, 119.3, 46.0 (d, *J* = 127.0 Hz), 35.8 (d, *J* = 4.0 Hz), 26.5 (d, *J* = 14.9 Hz), 23.3, 21.4. ^31^P NMR (202 MHz, DMSO) δ19.91. HRMS (ESI^–^) calculated for C_17_H_19_ClN_2_O_5_PS^–^ [M-H]^−^ 429.0446, found 429.0443.

#### (1-{[4-(5-Chlorothiophene-2-carboxamido)­phenyl]­amino}-4-methyl-1-oxopentan-2-yl)­phosphonic Acid (**133**)

Compound **133** was synthesized according to the general procedure C (Step 2), using **115** (106 mg, 0.25 mmol) and bromotrimethylsilane (165 μL, 1.25 mmol) in DCM (5 mL). The TMS ester was cleaved using MeOH (5 mL). Solvents were concentrated *in vacuo*, and the resultant oil was purified by preparative HPLC (CH_3_CN (HCOOH 0.05%)/H_2_O (HCOOH 0.05%) = 0.5/9.5 to 10/0). The product was obtained as a colorless solid (52 mg, 48%). ^1^H NMR (500 MHz, DMSO) δ 10.24 (d, *J* = 3.2 Hz, 1H), 9.97 (s, 1H), 8.41 (d, *J* = 17.4 Hz, 1H), 7.71 (d, *J* = 8.7 Hz, 2H), 7.57 (d, *J* = 8.6 Hz, 2H), 2.97 (dd, *J* = 22.3, 11.0 Hz, 1H), 1.97 (s, 1H), 1.57–1.39 (m, 2H), 0.87 (d, *J* = 6.2 Hz, 6H). ^13^C NMR (126 MHz, DMSO) δ 167.8 (d, *J* = 2.7 Hz), 157.8, 157.7, 150.9, 149.7, 148.1, 136.5, 135.8, 133.4, 129.6, 128.1, 121.0, 119.1, 46.1 (d, *J* = 126.7 Hz), 35.8, 26.5 (d, *J* = 14.3 Hz), 23.2, 21.4 (rotamers detected). ^31^P NMR (202 MHz, DMSO) δδ 19.95. HRMS (ESI^–^) calculated for C_16_H_18_ClN_3_O_5_PS^–^ [M-H]^−^ 430.0399, found 430.0391.

#### (1-{[4-(4,5-Dichlorothiophene-2-carboxamido)­phenyl]­amino}-4-methyl-1-oxopentan-2-yl)­phosphonic Acid (**134**)

Compound **134** was synthesized according to the general procedure C (Step 2), using **116** (105 mg, 0.20 mmol) and bromotrimethylsilane (132 μL, 1.00 mmol) in DCM (5 mL). The TMS ester was cleaved using MeOH (5 mL). Solvents were concentrated *in vacuo*, and the resultant oil was purified by preparative HPLC (CH_3_CN (HCOOH 0.05%)/H_2_O (HCOOH 0.05%) = 0.5/9.5 to 10/0). The product was obtained as a colorless solid (19 mg, 20%). ^1^H NMR (500 MHz, DMSO) δδ 10.33 (s, 1H), 10.00 (s, 1H), 8.07 (s, 1H), 7.59 (s, 4H), 2.98 (dt, *J* = 20.5, 6.9, 1H), 1.96 (s, 1H), 1.59–1.38 (m, 2H), 0.87 (d, *J* = 6.3, 6H). ^13^C NMR (126 MHz, DMSO) δ 167.8 (d, *J* = 6.6 Hz), 157.6, 137.5, 136.0, 133.1, 128.9, 128.0, 123.4, 120.7, 119.3, 46.1 (d, *J* = 126.8 Hz), 35.8, 26.5 (d, *J* = 14.5 Hz), 23.3, 21.4. ^31^P NMR (202 MHz, DMSO) δδ 19.86. HRMS (ESI^–^) calculated for C_17_H_18_Cl_2_N_2_O_5_PS^–^ [M-H]^−^ 463.0057, found 463.0042.

#### (4-Methyl-1-oxo-1-{[4-(4-phenylthiophene-2-carboxamido)­phenyl]­amino}­pentan-2-yl)­phosphonic Acid (**135**)

Compound **135** was synthesized according to the general procedure C (Step 2), using **117** (99 mg, 0.19 mmol) and bromotrimethylsilane (125 μL, 0.95 mmol) in DCM (5 mL). The TMS ester was cleaved using MeOH (5 mL). Solvents were concentrated *in vacuo* and the resultant oil was purified by preparative HPLC (CH_3_CN (HCOOH 0.05%)/H_2_O (HCOOH 0.05%) = 0.5/9.5 to 10/0). The product was obtained as a colorless solid (21 mg, 23%). ^1^H NMR (500 MHz, DMSO) δδ 10.25 (s, 1H), 10.03 (s, 1H), 8.47 (d, *J* = 1.5 Hz, 1H), 8.17 (d, *J* = 1.4 Hz, 1H), 7.77–7.72 (m, 2H), 7.63 (q, *J* = 8.9 Hz, 4H), 7.47 (t, *J* = 7.7 Hz, 2H), 7.35 (t, *J* = 7.3 Hz, 1H), 2.98 (dd, *J* = 22.6, 11.2 Hz, 1H), 1.97 (s, 1H), 1.56–1.36 (m, 2H), 0.87 (d, *J* = 6.3 Hz, 6H). ^13^C NMR (126 MHz, DMSO) δ 167.9, 159.5, 141.9, 140.9, 135.7, 134.7, 133.8, 129.2, 127.7, 127.5, 126.4, 126.0, 120.7, 119.3, 46.1 (d, *J* = 127.2 Hz), 35.8 (d, *J* = 2.2 Hz), 26.5 (d, *J* = 14.5 Hz), 23.4, 21.5. ^31^P NMR (202 MHz, DMSO) δδ 19.91. HRMS (ESI^–^) calculated for C_23_H_24_N_2_O_5_PS^–^ [M-H]^−^ 471.1149, found 471.1154.

#### (1-{[4-(Furan-2-carboxamido)­phenyl]­amino}-4-methyl-1-oxopentan-2-yl)­phosphonic Acid (**136**)

Compound **136** was synthesized according to the general procedure C (Step 2), using **118** (109 mg, 0.24 mmol) and bromotrimethylsilane (158 μL, 1.20 mmol) in DCM (5 mL). The TMS ester was cleaved using MeOH (5 mL). Solvents were concentrated *in vacuo*, and the resultant oil was purified by preparative HPLC (CH_3_CN (HCOOH 0.05%)/H_2_O (HCOOH 0.05%) = 0.5/9.5 to 10/0). The product was obtained as a pale yellow solid (46 mg, 50%). ^1^H NMR (500 MHz, DMSO) δ 10.13 (s, 1H), 9.99 (s, 1H), 7.92 (d, *J* = 1.6 Hz, 1H), 7.64 (d, *J* = 9.0 Hz, 2H), 7.56 (d, *J* = 9.1 Hz, 2H), 7.30 (d, *J* = 3.4 Hz, 1H), 6.69 (dd, *J* = 3.5, 1.7 Hz, 1H), 2.97 (ddd, *J* = 22.3, 11.4, 2.8 Hz, 1H), 1.95 (dq, *J* = 11.7, 6.9, 4.7 Hz, 1H), 1.55–1.35 (m, 2H), 0.86 (d, *J* = 6.4 Hz, 6H). ^13^C NMR (126 MHz, DMSO) δ 167.8 (d, *J* = 5.9 Hz), 156.0, 147.7, 145.7, 135.6, 133.6, 120.8, 119.2, 114.5, 112.2, 46.1 (d, *J* = 127.4 Hz), 35.9, 26.5 (d, *J* = 14.7 Hz), 23.3, 21.5. ^31^P NMR (202 MHz, DMSO) δ 19.93. HRMS (ESI^–^) calculated for C_17_H_20_N_2_O_6_P^–^ [M-H]^−^ 379.1064, found 390.1071.

#### (4-Methyl-1-oxo-1-{[4-(quinoline-3-carboxamido)­phenyl]­amino}­pentan-2-yl)­phosphonic Acid (**137**)

Compound **137** was synthesized according to the general procedure C (Step 2), using **119** (103 mg, 0.23 mmol) and bromotrimethylsilane (152 μL, 1.15 mmol) in DCM (5 mL). The TMS ester was cleaved using MeOH (5 mL). Solvents were concentrated *in vacuo*, and the resultant oil was purified by preparative HPLC (CH_3_CN (HCOOH 0.05%)/H_2_O (HCOOH 0.05%) = 0.5/9.5 to 10/0). The product was obtained as a pale yellow solid (38 mg, 37%). ^1^H NMR (500 MHz, DMSO) δ 10.56 (s, 1H), 9.98 (s, 1H), 9.35 (d, *J* = 2.3 Hz, 1H), 8.95 (d, *J* = 2.2 Hz, 1H), 8.21–8.07 (m, 2H), 7.90 (ddd, *J* = 8.4, 6.7, 1.5 Hz, 1H), 7.78–7.70 (m, 3H), 7.66–7.58 (m, 2H), 2.99 (ddd, *J* = 22.4, 11.4, 2.9 Hz, 1H), 1.98 (tdd, *J* = 12.0, 7.1, 4.2 Hz, 1H), 1.47 (dddd, *J* = 32.5, 16.5, 7.3, 4.2 Hz, 2H), 0.88 (dd, *J* = 6.5, 1.6 Hz, 6H). ^13^C NMR (126 MHz, DMSO) δ 167.7, 163.8, 149.1, 148.5, 135.9, 135.7, 134.1, 131.4, 129.2, 128.8, 127.7, 127.5, 126.5, 120.7, 119.3, 46.0 (d, *J* = 126.9 Hz), 35.8 (d, *J* = 3.8 Hz), 26.5 (d, *J* = 14.5 Hz), 23.3, 21.4. ^31^P NMR (202 MHz, DMSO) δ 19.99. HRMS (ESI^–^) calculated for C_22_H_23_N_3_O_5_P^–^ [M-H]^−^ 440.1381, found 440.1381.

#### (1-{[4-(Benzo­[d]­thiazole-2-carboxamido)­phenyl]­amino}-4-methyl-1-oxopentan-2-yl)­phosphonic Acid (**138**)

Compound **138** was synthesized according to the general procedure C (Step 2), using **120** (102 mg, 0.23 mmol) and bromotrimethylsilane (152 μL, 1.15 mmol) in DCM (5 mL). The TMS ester was cleaved using MeOH (5 mL). Solvents were concentrated *in vacuo*, and the resultant oil was purified by preparative HPLC (CH_3_CN (HCOOH 0.05%)/H_2_O (HCOOH 0.05%) = 0.5/9.5 to 10/0). The product was obtained as a pale yellow solid (36 mg, 36%). ^1^H NMR (500 MHz, DMSO) δ 11.04 (s, 1H), 10.00 (s, 1H), 8.28–8.25 (m, 1H), 8.21 (d, *J* = 8.1 Hz, 1H), 7.84–7.79 (m, 2H), 7.69–7.64 (m, 1H), 7.64–7.59 (m, 3H), 2.98 (ddd, *J* = 22.6, 11.3, 2.9 Hz, 1H), 1.97 (tdd, *J* = 11.8, 7.1, 4.2 Hz, 1H), 1.46 (ddtd, *J* = 35.5, 12.7, 9.6, 4.7 Hz, 2H), 0.87 (d, *J* = 6.3 Hz, 6H). ^13^C NMR (126 MHz, DMSO) δ 167.8 (d, *J* = 5.0 Hz), 164.9, 157.8, 152.7, 136.4, 136.2, 132.9, 127.3, 127.1, 124.1, 123.1, 121.1, 119.2, 46.1 (d, *J* = 126.7 Hz), 35.8 (d, *J* = 4.2 Hz), 26.5 (d, *J* = 14.6 Hz), 23.2, 21.4. ^31^P NMR (202 MHz, DMSO) δ 19.90. HRMS (ESI^–^) calculated for C_20_H_21_N_3_O_5_PS^–^ [M-H]^−^ 446.0945, found 446.0947.

#### (4-Methyl-1-oxo-1-{[4-(5-phenylthiophene-2-carboxamido)­phenyl]­amino}­pentan-2-yl)­phosphonic Acid (**139**)

Compound **139** was synthesized according to the general procedure C (Step 2), using **121** (98 mg, 0.21 mmol) and bromotrimethylsilane (139 μL, 1.05 mmol) in DCM (5 mL). The TMS ester was cleaved using MeOH (5 mL). Solvents were concentrated *in vacuo*, and the resultant oil was purified by preparative HPLC (CH_3_CN (HCOOH 0.05%)/H_2_O (HCOOH 0.05%) = 0.5/9.5 to 10/0). The product was obtained as a pale yellow solid (48 mg, 48%). ^1^H NMR (500 MHz, DMSO) δ 10.21 (s, 1H), 9.94 (s, 1H), 8.01 (d, *J* = 4.0 Hz, 1H), 7.75 (dd, *J* = 7.3, 1.7 Hz, 2H), 7.66–7.58 (m, 5H), 7.49–7.44 (m, 2H), 7.41–7.36 (m, 1H), 2.98 (ddd, *J* = 22.5, 11.3, 2.9 Hz, 1H), 1.98 (tq, *J* = 12.1, 5.7, 4.8 Hz, 1H), 1.55–1.37 (m, 2H), 0.87 (dd, *J* = 6.4, 1.3 Hz, 6H). ^13^C NMR (126 MHz, DMSO) δ 167.6 (d, *J* = 4.8 Hz), 159.4, 148.2, 139.1, 135.5, 133.8, 133.0, 130.0, 129.3, 128.7, 125.7, 124.5, 120.8, 119.3, 46.0 (d, *J* = 127.0 Hz), 35.8 (d, *J* = 4.1 Hz), 26.5 (d, *J* = 14.6 Hz), 23.3, 21.4. ^31^P NMR (202 MHz, DMSO) δ 20.01. HRMS (ESI^–^) calculated for C_23_H_24_N_2_O_5_PS^–^ [M-H]^−^ 471.1149, found 471.1134.

#### (4-Methyl-1-oxo-1-{[4-(phenylsulfonamido)­phenyl]­amino}­pentan-2-yl)­phosphonic Acid (**140**)

Compound **140** was synthesized according to the general procedure C (Step 2), using **122** (156 mg, 0.32 mmol) and bromotrimethylsilane (215 μL, 1.62 mmol) in DCM (4 mL). The TMS ester was cleaved using MeOH (4 mL). Solvents were concentrated *in vacuo*, and the resultant oil was purified by preparative HPLC (CH_3_CN (HCOOH 0.05%)/H_2_O (HCOOH 0.05%) = 1/9 to 10/0). The product was obtained as white solid (83 mg, 61%). ^1^H NMR (500 MHz, DMSO) δ 10.07 (s, 1H), 9.90 (s, 1H), 7.70 (dd, *J* = 5.3, 3.4 Hz, 2H), 7.58 (ddd, *J* = 6.4, 3.7, 1.2 Hz, 1H), 7.56–7.49 (m, 2H), 7.44 (d, *J* = 8.8 Hz, 2H), 6.97 (d, *J* = 8.8 Hz, 2H), 2.98–2.84 (m, 1H), 1.92 (s, 1H), 1.48–1.27 (m, 2H), 0.83 (dd, *J* = 6.2, 2.2 Hz, 6H). ^13^C NMR (126 MHz, DMSO) δ 167.7, 139.4, 136.3, 132.9, 132.4, 129.3, 126.7, 121.7, 119.7, 46.0 (d, *J* = 126.6 Hz), 35.7, 26.4 (d, *J* = 14.0 Hz), 23.3, 21.4. ^31^P NMR (202 MHz, DMSO) δ 19.87. HRMS (ESI^–^) calculated for C_18_H_22_N_2_O_6_PS^–^ [M-H]^−^ 425.0942, found 425.0931.

#### [1-({4-[(3,4-Dichlorophenyl)­sulfonamido]­phenyl}­amino)-4-methyl-1-oxopentan-2-yl]­phosphonic Acid (**141**)

Compound **141** was synthesized according to the general procedure C (Step 2), using **123** (80 mg, 0.14 mmol) and bromotrimethylsilane (100 μL, 0.72 mmol) in DCM (4 mL). The TMS ester was cleaved using MeOH (4 mL). Solvents were concentrated *in vacuo*, and the resultant oil was purified by preparative HPLC (CH_3_CN (HCOOH 0.05%)/H_2_O (HCOOH 0.05%) = 1/9 to 10/0). The product was obtained as a white solid (48.4 mg, 70%). ^1^H NMR (500 MHz, DMSO) δ 10.22 (s, 1H), 9.92 (s, 1H), 7.89 (d, *J* = 2.1 Hz, 1H), 7.82 (d, *J* = 8.4 Hz, 1H), 7.60 (dd, *J* = 8.4, 2.1 Hz, 1H), 7.53–7.45 (m, 2H), 7.04–6.94 (m, 2H), 2.92 (ddd, *J* = 22.4, 11.3, 2.6 Hz, 1H), 1.99–1.86 (m, 1H), 1.52–1.33 (m, 2H), 0.83 (d, *J* = 6.3 Hz, 6H). ^13^C NMR (126 MHz, DMSO) δ 167.8 (d, *J* = 4.9 Hz), 139.7, 136.9, 136.0, 132.2, 131.8, 131.5, 128.4, 126.9, 122.3, 119.8, 46.0 (d, *J* = 127.0 Hz), 35.7 (d, *J* = 3.7 Hz), 26.4 (d, *J* = 14.7 Hz), 23.3, 21.4. ^31^P NMR (202 MHz, DMSO) δ 19.76. HRMS (ESI^–^) calculated for C_18_H_20_Cl_2_N_2_O_6_PS^–^ [M-H]^−^ 493.0162, found 493.0156.

#### [1-({4-[(3,4-Dimethoxyphenyl)­sulfonamido]­phenyl}­amino)-4-methyl-1-oxopentan-2-yl]­phosphonic Acid (**142**)

Compound **142** was synthesized according to the general procedure C (Step 2), using **124** (195 mg, 0.36 mmol) and bromotrimethylsilane (240 μL, 1.80 mmol) in DCM (6 mL). The TMS ester was cleaved using MeOH (6 mL). Solvents were concentrated *in vacuo*, and the resultant oil was purified by preparative HPLC (CH_3_CN (HCOOH 0.05%)/H_2_O (HCOOH 0.05%) = 1/9 to 10/0). The product was obtained as white solid (109 mg, 62%). ^1^H NMR (500 MHz, DMSO) δ 9.86 (s, 1H), 9.85 (s, 1H), 7.44 (d, *J* = 8.6 Hz, 2H), 7.26–7.20 (m, 2H), 7.03 (d, *J* = 8.4 Hz, 1H), 6.99 (d, *J* = 8.6 Hz, 2H), 3.78 (s, 3H), 3.73 (s, 3H), 2.98–2.83 (m, 1H), 1.92 (s, 1H), 1.50–1.30 (m, 2H), 0.83 (d, *J* = 6.0 Hz, 6H). ^13^C NMR (126 MHz, DMSO) δ 167.7 (d, *J* = 5.0 Hz), 152.1, 148.5, 136.2, 132.7, 130.8, 121.7, 120.5, 119.6, 111.0, 109.4, 55.7, 55.7, 46.7–45.3 (m), 35.7, 26.4 (d, *J* = 12.3 Hz), 23.2, 21.3. ^31^P NMR (202 MHz, DMSO) δ 19.89. HRMS (ESI^–^) calculated for C_20_H_26_N_2_O_8_PS^–^ [M-H]^−^ 485.1153, found 485.1139.

#### (4-Methyl-1-oxo-1-{[4-(thiophene-2-sulfonamido)­phenyl]­amino}­pentan-2-yl)­phosphonic Acid (**143**)

Compound **143** was synthesized according to the general procedure C (Step 2), using **125** (175 mg, 0.36 mmol) and bromotrimethylsilane (470 μL, 3.58 mmol) in DCM (5 mL). The TMS ester was cleaved using MeOH (5 mL). Solvents were concentrated *in vacuo*, and the resultant oil was purified by preparative HPLC (CH_3_CN (HCOOH 0.05%)/H_2_O (HCOOH 0.05%) = 1/9 to 10/0). The product was obtained as white solid (68.7 mg, 44%). ^1^H NMR (500 MHz, DMSO) δ 10.19 (s, 1H), 9.95 (s, 1H), 7.86 (dd, *J* = 5.0, 1.3 Hz, 1H), 7.49 (d, *J* = 8.9 Hz, 2H), 7.46 (dd, *J* = 3.7, 1.3 Hz, 1H), 7.10 (dd, *J* = 4.9, 3.8 Hz, 1H), 7.02 (d, *J* = 8.9 Hz, 2H), 2.93 (dd, *J* = 21.7, 10.6 Hz, 1H), 1.97–1.88 (m, *J* = 15.0, 7.5 Hz, 1H), 1.51–1.30 (m, 2H), 0.93–0.76 (m, 6H). ^13^C NMR (126 MHz, DMSO) δ 167.8, 139.8, 136.7, 133.3, 132.4, 132.1, 127.7, 122.0, 119.7, 46.0 (d, *J* = 126.7 Hz), 35.8, 26.5 (d, *J* = 14.7 Hz), 23.4, 21.4. ^31^P NMR (202 MHz, DMSO) δ 19.82. HRMS (ESI^–^) calculated for C_16_H_20_N_2_O_6_PS_2_^–^ [M-H]^−^ 431.0506, found 431.0506.

#### (4-Methyl-1-{[4-(naphthalene-2-sulfonamido)­phenyl]­amino}-1-oxopentan-2-yl)­phosphonic Acid (**144**)

Compound **144** was synthesized according to the general procedure C (Step 2), using **126** (110.6 mg, 0.21 mmol) and bromotrimethylsilane (275 μL, 2.08 mmol) in DCM (4 mL). The TMS ester was cleaved using MeOH (4 mL). Solvents were concentrated *in vacuo*, and the resultant oil was purified by preparative HPLC (CH_3_CN (HCOOH 0.05%)/H_2_O (HCOOH 0.05%) = 1/9 to 10/0). The product was obtained as a white solid (43.2 mg, 43%). ^1^H NMR (500 MHz, DMSO) δ 10.18 (s, 1H), 9.87 (s, 1H), 8.37 (d, *J* = 1.3 Hz, 1H), 8.09 (dd, *J* = 19.3, 8.4 Hz, 2H), 7.99 (d, *J* = 8.1 Hz, 1H), 7.72 (dd, *J* = 8.7, 1.8 Hz, 1H), 7.70–7.65 (m, 1H), 7.65–7.60 (m, 1H), 7.41 (d, *J* = 8.8 Hz, 2H), 7.00 (d, *J* = 8.8 Hz, 2H), 2.88 (dd, *J* = 21.9, 10.8 Hz, 1H), 1.90 (s, 1H), 1.47–1.30 (m, 2H), 0.80 (dd, *J* = 6.1, 3.0 Hz, 6H). ^13^C NMR (126 MHz, DMSO) δ 167.7 (d, *J* = 2.9 Hz), 136.5, 136.3, 134.3, 132.3, 131.6, 129.4, 129.3, 129.0, 128.0, 127.9, 127.7, 122.2, 121.7, 119.7, 46.0 (d, *J* = 125.0 Hz), 35.7, 26.4 (d, *J* = 14.6 Hz), 23.3, 21.3. ^31^P NMR (202 MHz, DMSO) δ 19.81. HRMS (ESI^–^) calculated for C_22_H_24_N_2_O_6_PS^–^ [M-H]^−^ 475.1098, found 475.1082.

#### (1-{[4-(Cyclohexanesulfonamido)­phenyl]­amino}-4-methyl-1-oxopentan-2-yl)­phosphonic Acid (**145**)

Compound **145** was synthesized according to the general procedure C (Step 2), using **127** (123.3 mg, 0.25 mmol) and bromotrimethylsilane (230 μL, 1.77 mmol) in DCM (4 mL). TMS ester was cleaved using MeOH (4 mL). Solvents were concentrated *in vacuo*, and the resultant oil was purified by preparative HPLC (CH_3_CN (HCOOH 0.05%)/H_2_O (HCOOH 0.05%) = 1/9 to 10/0). The product was obtained as a white solid (57.3 mg, 53%). ^1^H NMR (500 MHz, DMSO) δ 9.95 (s, 1H), 9.61 (s, 1H), 7.53 (d, *J* = 8.9 Hz, 2H), 7.13 (d, *J* = 8.9 Hz, 2H), 3.00–2.83 (m, 2H), 2.03–1.90 (m, 3H), 1.73 (d, *J* = 13.0 Hz, 2H), 1.60–1.30 (m, 5H), 1.24–1.03 (m, 3H), 0.85 (d, *J* = 6.4 Hz, 6H). ^13^C NMR (126 MHz, DMSO) δ 168.1, 136.2, 133.8, 120.9, 120.3, 58.9, 46.4 (d, *J* = 127.4 Hz), 36.2, 26.9 (d, *J* = 14.7 Hz), 26.4, 25.2, 24.8, 23.7, 21.8. ^31^P NMR (202 MHz, DMSO) δ 19.96. HRMS (ESI^+^) calculated for C_18_H_30_N_2_O_6_PS^+^ [M + H]^+^ 433.1557, found 433.1542.

#### {4-Methyl-1-oxo-1-[(4-{[4-(2-oxopyrrolidin-1-yl)­phenyl]­sulfonamido}­phenyl)­amino]­pentan-2-yl}­phosphonic Acid (**146**)

Compound **146** was synthesized according to the general procedure C (Step 2), using **128** (120 mg, 0.21 mmol) and bromotrimethylsilane (200 μL, 1.48 mmol) in DCM (4 mL). The TMS ester was cleaved using MeOH (4 mL). Solvents were concentrated *in vacuo*, and the resultant oil was purified by preparative HPLC (CH_3_CN (HCOOH 0.05%)/H_2_O (HCOOH 0.05%) = 1/9 to 10/0). The product was obtained as a white solid (68.3 mg, 64%). ^1^H NMR (500 MHz, DMSO) δ 9.98 (s, 1H), 9.86 (s, 1H), 7.84–7.75 (m, 2H), 7.71–7.64 (m, 2H), 7.43 (d, *J* = 8.9 Hz, 2H), 6.98 (d, *J* = 8.9 Hz, 2H), 3.81 (t, *J* = 7.0 Hz, 2H), 2.91 (ddd, *J* = 22.4, 11.3, 2.6 Hz, 1H), 2.48–2.52 (m, 2H), 2.09–1.99 (m, 2H), 1.91 (dtd, *J* = 11.6, 7.7, 3.7 Hz, 1H), 1.49–1.32 (m, 2H), 0.83 (dd, *J* = 6.4, 1.8 Hz, 6H). ^13^C NMR (126 MHz, DMSO) δ 174.7, 167.6 (d, *J* = 4.8 Hz), 143.0, 136.2, 133.5, 132.5, 127.7, 121.4, 119.6, 118.6, 47.9, 45.9 (d, *J* = 126.7 Hz), 35.7, 32.4, 26.4 (d, *J* = 14.7 Hz), 23.2, 21.3, 17.3. ^31^P NMR (202 MHz, DMSO) δ 19.82. HRMS (ESI^–^) calculated for C_22_H_27_N_3_O_7_PS^–^ [M-H]^−^ 508.1313, found 508.1316.

#### [1-({4-[2-(3,4-Dichlorophenyl)­acetamido]­phenyl}­amino)-4-methyl-1-oxopentan-2-yl]­phosphonic Acid (**195**)

Compound **195** was synthesized over two steps according to the general procedure C using **186** (190 mg, 0.45 mmol) and triethyl phosphite (3 mL). The resultant oil was purified by column chromatography (Hex/EtOAc = 1/1 to EtOAc/MeOH = 1/1) to give diethyl phosphonate as a white solid (70.6 mg, 30%). The product obtained (67.2 mg, 0.13 mmol) was then treated with bromotrimethylsilane (120 μL, 0.89 mmol) in DCM (5 mL). TMS ester was cleaved using MeOH (5 mL). Solvents were concentrated *in vacuo*, and the resultant oil was purified by preparative HPLC (CH_3_CN (HCOOH 0.05%)/H_2_O (HCOOH 0.05%) = 1/9 to 10/0). The product was obtained as a white solid (15.6 mg, 25%; 7.5% over two steps). ^1^H NMR (500 MHz, DMSO) δ 10.14 (s, 1H), 9.91 (s, 1H), 7.62–7.56 (m, 2H), 7.50 (dd, *J* = 21.8, 9.1 Hz, 4H), 7.31 (dd, *J* = 8.3, 2.0 Hz, 1H), 3.66 (s, 2H), 2.94 (ddd, *J* = 22.3, 11.3, 2.5 Hz, 1H), 2.00–1.89 (m, 1H), 1.44 (ddt, *J* = 29.9, 19.8, 8.3 Hz, 2H), 0.85 (d, *J* = 6.4 Hz, 6H). ^13^C NMR (126 MHz, DMSO) δ 167.9, 167.6 (d, *J* = 3.9 Hz), 137.2, 135.2, 134.3, 131.4, 130.7, 130.4, 129.8, 129.3, 119.5, 119.4, 46.0 (d, *J* = 126.9 Hz), 41.9, 35.8 (d, *J* = 3.6 Hz), 26.5 (d, *J* = 14.9 Hz), 23.3, 21.4. ^31^P NMR (202 MHz, DMSO) δ 20.01. HRMS (ESI^–^) calculated for C_20_H_22_Cl_2_N_2_O_5_P^–^ [M-H]^−^ 471.0649, found 471.0653.

#### [4-Methyl-1-oxo-1-({4-[2-(thiophen-2-yl)­acetamido]­phenyl}­amino)­pentan-2-yl]­phosphonic Acid (**197**)

Compound **197** was synthesized over two steps according to the general procedure C using **188** (166 mg, 0.46 mmol) and triethyl phosphite (2 mL). The resultant oil was purified by column chromatography (Hex/EtOAc = 1/1 to EtOAc) to give diethyl phosphonate as a white solid (80.7 mg, 38%). The product obtained (77.5 mg, 0.17 mmol) was then treated with bromotrimethylsilane (150 μL, 1.16 mmol) in DCM (3 mL). The TMS ester was cleaved using MeOH (3 mL). Solvents were concentrated *in vacuo*, and the resultant oil was purified by preparative HPLC (CH_3_CN (HCOOH 0.05%)/H_2_O (HCOOH 0.05%) = 1/9 to 10/0). The product was obtained as a white solid (25.4 mg, 36%; 14% over two steps). ^1^H NMR (500 MHz, DMSO) δ 10.15 (s, 1H), 9.91 (s, 1H), 7.51 (q, *J* = 9.1 Hz, 4H), 7.43–7.33 (m, 1H), 6.97 (d, *J* = 3.6 Hz, 2H), 3.84 (s, 2H), 2.94 (dd, *J* = 21.4, 10.4 Hz, 1H), 1.99–1.89 (m, 1H), 1.52–1.34 (m, 2H), 0.85 (d, *J* = 6.4 Hz, 6H). ^13^C NMR (126 MHz, DMSO) δ 167.7, 162.0 (d, *J* = 29.5 Hz), 137.3, 135.2, 134.3, 126.7, 126.4, 125.1, 119.5, 119.4, 46.0 (d, *J* = 127.1 Hz), 37.5, 35.8 (d, *J* = 3.0 Hz), 26.5 (d, *J* = 14.8 Hz), 23.3, 21.4. ^31^P NMR (202 MHz, DMSO) δ 20.03. HRMS (ESI^–^) calculated for C_18_H_22_N_2_O_5_PS^–^ [M-H]^−^ 409.0993, found 409.0993.

#### [1-({3-[2-(3,4-Dichlorophenyl)­acetamido]­phenyl}­amino)-4-methyl-1-oxopentan-2-yl]­phosphonic Acid (**205**)

Compound **205** was synthesized over two steps according to the general procedure C using **203** (190 mg, 0.45 mmol) and triethyl phosphite (2 mL). The resultant oil was purified by column chromatography (Hex/EtOAc = 7/3 to 3/7) to give diethyl phosphonate as transparent oil (157.3 mg, 74%). The product obtained (155 mg, 0.29 mmol) was then treated with bromotrimethylsilane (200 μL, 1.46 mmol) in DCM (6 mL). The TMS ester was cleaved using MeOH (6 mL). Solvents were concentrated *in vacuo*, and the resultant oil was purified by preparative HPLC (CH_3_CN (HCOOH 0.05%)/H_2_O (HCOOH 0.05%) = 1/9 to 10/0). The product was obtained as a white solid (81 mg, 58%; 43% over two steps). ^1^H NMR (500 MHz, DMSO) δ 10.18 (s, 1H), 9.93 (s, 1H), 7.94 (s, 1H), 7.59 (t, *J* = 5.4 Hz, 2H), 7.36–7.12 (m, 4H), 3.67 (s, 2H), 2.98 (dd, *J* = 22.2, 10.8 Hz, 1H), 2.02–1.89 (m, *J* = 6.2 Hz, 1H), 1.54–1.33 (m, 2H), 0.86 (d, *J* = 6.4 Hz, 6H). ^13^C NMR (126 MHz, DMSO) δ 168.2, 167.9 (d, *J* = 3.7 Hz), 139.7, 139.2, 137.1, 131.3, 130.7, 130.4, 129.7, 129.3, 128.8, 114.3, 114.0, 110.2, 46.0 (d, *J* = 127.4 Hz), 42.0, 35.8, 26.5 (d, *J* = 14.5 Hz), 23.2, 21.4. ^31^P NMR (202 MHz, DMSO) δ 19.97. HRMS (ESI^–^) calculated for C_20_H_22_Cl_2_N_2_O_5_P^–^ [M-H]^−^ 471.0649, found 471.0649.

#### [1-({4-[2-(3,4-Dichlorophenyl)­acetamido]-3-fluorophenyl}­amino)-4-methyl-1-oxopentan-2-yl]­phosphonic Acid (**157**)

Compound **157** was synthesized over two steps according to the general procedure C (Step 2), using **154** (200 mg, 0.36 mmol) and bromotrimethylsilane (480 μL, 3.65 mmol) in DCM (6 mL). The TMS ester was cleaved using MeOH (6 mL). Solvents were concentrated *in vacuo*, and the resultant oil was purified by preparative HPLC (CH_3_CN (HCOOH 0.05%)/H_2_O (HCOOH 0.05%) = 1/9 to 10/0). The product was obtained as a white solid (89.5 mg, 50%). ^1^H NMR (500 MHz, DMSO) δ 10.17 (s, 1H), 9.92 (s, 1H), 7.73–7.64 (m, 2H), 7.63–7.55 (m, 2H), 7.32 (dd, *J* = 8.3, 1.9 Hz, 1H), 7.19 (dd, *J* = 8.8, 1.5 Hz, 1H), 3.72 (s, 2H), 2.95 (ddd, *J* = 22.3, 11.2, 2.3 Hz, 1H), 1.95 (ddd, *J* = 15.7, 9.9, 3.7 Hz, 1H), 1.52–1.35 (m, 2H), 0.85 (d, *J* = 6.3 Hz, 6H). ^13^C NMR (126 MHz, DMSO) δ 168.6, 168.2 (d, *J* = 4.9 Hz), 153.7 (d, *J* = 243.0 Hz), 137.1, 137.0, 131.4, 130.8, 130.5, 129.8, 129.3, 124.8, 120.6 (d, *J* = 12.4 Hz), 114.5, 106.2 (d, *J* = 24.8 Hz), 46.2 (d, *J* = 126.6 Hz), 41.2, 35.7, 26.5 (d, *J* = 14.7 Hz), 23.3, 21.4. ^31^P NMR (202 MHz, DMSO) δ 19.49. ^19^F NMR (470 MHz, DMSO) δ – 22.79. HRMS (ESI^–^) calculated for C_20_H_21_Cl_2_FN_2_O_5_P^–^ [M-H]^−^ 489.0555, found 489.0554.

#### [1-({4-[2-(3,4-Dichlorophenyl)­acetamido]-3-(trifluoromethyl)­phenyl}­amino)-4-methyl-1-oxopentan-2-yl]­phosphonic Acid (**158**)

Compound **158** was synthesized over two steps according to the general procedure C (Step 2), using **155** (129 mg, 0.22 mmol) and bromotrimethylsilane (285 μL, 2.20 mmol) in DCM (4 mL). The TMS ester was cleaved using MeOH (4 mL). Solvents were concentrated *in vacuo*, and the resultant oil was purified by preparative HPLC (CH_3_CN (HCOOH 0.05%)/H_2_O (HCOOH 0.05%) = 1/9 to 10/0). The product was obtained as a white solid (61 mg, 51%). ^1^H NMR (500 MHz, DMSO) δ 10.33 (s, 1H), 9.77 (s, 1H), 8.14 (d, *J* = 1.7 Hz, 1H), 7.72 (d, *J* = 8.5 Hz, 1H), 7.64–7.55 (m, 2H), 7.40–7.25 (m, 2H), 3.69 (s, 2H), 3.01–2.91 (m, 1H), 2.04–1.91 (m, 1H), 1.53–1.38 (m, 2H), 0.86 (d, *J* = 6.3 Hz, 6H). ^13^C NMR (126 MHz, DMSO) δ 169.4, 168.5 (d, *J* = 4.4 Hz), 138.0, 137.0, 131.2, 131.2, 130.8, 130.4, 129.7, 129.6, 129.3, 125.3 (q, *J* = 29.0 Hz), 123.4 (q, *J* = 273.4 Hz), 122.8, 116.1 (q, *J* = 5.0 Hz), 46.3 (d, *J* = 125.9 Hz), 41.0, 35.6, 26.5 (d, *J* = 14.5 Hz), 23.2, 21.4. ^31^P NMR (202 MHz, DMSO) δ 19.22. ^19^F NMR (470 MHz, DMSO) δ −59.73. HRMS (ESI^–^) calculated for C_21_H_21_Cl_2_F_3_N_2_O_5_P^–^ [M-H]^−^ 539.0523, found 539.0512.

#### {1-[(4-{[(3,4-Dichlorophenyl)­methyl]­sulfonamido}­phenyl)­amino]-4-methyl-1-oxopentan-2-yl}­phosphonic Acid (**159**)

Compound **159** was synthesized according to the general procedure C (Step 2), using **156** (60 mg, 0.11 mmol) and bromotrimethylsilane (70 μL, 0.53 mmol) in DCM (2 mL). The TMS ester was cleaved using MeOH (2 mL). Solvents were concentrated *in vacuo*, and the resultant oil was purified by preparative HPLC (CH_3_CN (HCOOH 0.05%)/H_2_O (HCOOH 0.05%) = 1/9 to 10/0). The product was obtained as a white solid (18.5 mg, 34%). ^1^H NMR (500 MHz, DMSO) δ 9.96 (s, 1H), 9.72 (s, 1H), 7.64 (d, *J* = 8.3 Hz, 1H), 7.58 (d, *J* = 8.7 Hz, 2H), 7.50 (d, *J* = 1.9 Hz, 1H), 7.24 (dd, *J* = 8.3, 2.0 Hz, 1H), 7.12 (d, *J* = 8.8 Hz, 2H), 4.48 (s, 2H), 2.96 (dd, *J* = 22.1, 11.2 Hz, 1H), 1.96 (s, 1H), 1.44 (dd, *J* = 26.4, 14.6 Hz, 2H), 0.87 (d, *J* = 6.3 Hz, 6H). ^13^C NMR (126 MHz, DMSO) δ 167.7, 135.9, 132.8, 132.8, 131.3, 131.1, 130.9, 130.9, 130.5, 120.4, 119.8, 55.2, 46.0 (d, *J* = 132.8 Hz), 35.8, 26.5 (d, *J* = 14.3 Hz), 23.3, 21.4. ^31^P NMR (202 MHz, DMSO) δ 19.93. HRMS (ESI^–^) calculated for C_19_H_22_Cl_2_N_2_O_6_PS^–^ [M-H]^−^ 507.0319, found 507.0320.

#### {1-[(4-Benzoylphenyl)­amino]-4-methyl-1-oxopentan-2-yl}­phosphonic Acid (**189**)

Compound **189** was synthesized over two steps according to the general procedure C using **180** (195 mg, 0.52 mmol) and triethyl phosphite (2 mL). The resultant oil was purified by column chromatography (Hex/EtOAc = 7/3 to 3/7) to give diethyl phosphonate as transparent oil (132 mg, 59%). The product obtained was then treated with bromotrimethylsilane (275 μL, 2.08 mmol) in DCM (6 mL). TMS ester was cleaved using MeOH (6 mL). Solvents were concentrated *in vacuo* and the resultant oil was purified by preparative HPLC (CH_3_CN (HCOOH 0.05%)/H_2_O (HCOOH 0.05%) = 1/9 to 10/0). The product was obtained as a white solid (90 mg, 80%; 47% over two steps). ^1^H NMR (500 MHz, DMSO) δ 10.37 (s, 1H), 7.81–7.77 (m, 2H), 7.75–7.69 (m, 4H), 7.66 (ddd, *J* = 8.7, 2.5, 1.2 Hz, 1H), 7.55 (dd, *J* = 10.6, 4.6 Hz, 2H), 3.06 (ddd, *J* = 22.5, 11.2, 2.5 Hz, 1H), 2.03–1.93 (m, 1H), 1.55–1.39 (m, 2H), 0.87 (d, *J* = 5.8 Hz, 6H). ^13^C NMR (126 MHz, DMSO) δ 194.6, 168.7 (d, *J* = 4.9 Hz), 143.6, 137.7, 132.3, 131.2, 129.4, 128.5, 118.3, 46.3 (d, *J* = 126.0 Hz), 35.7, 26.6 (d, *J* = 14.4 Hz), 23.2, 21.4. ^31^P NMR (202 MHz, DMSO) δ 19.32. HRMS (ESI^–^) calculated for C_19_H_21_NO_5_P^–^ [M-H]^−^ 374.1163, found 374.1163.

#### {1-[(4-Benzylphenyl)­amino]-4-methyl-1-oxopentan-2-yl}­phosphonic Acid (**190**)

Compound **190** was synthesized over two steps according to the general procedure C using **181** (158 mg, 0.44 mmol) and triethyl phosphite (1 mL). The resultant oil was purified by column chromatography (Hex/EtOAc = 7/3 to 3/7) to give diethyl phosphonate as transparent oil (80 mg, 44%). The product obtained was then treated with bromotrimethylsilane (125 μL, 0.95 mmol) in DCM (4 mL). TMS ester was cleaved using MeOH (4 mL). Solvents were concentrated *in vacuo*, and the resultant oil was purified by preparative HPLC (CH_3_CN (HCOOH 0.05%)/H_2_O (HCOOH 0.05%) = 1/9 to 10/0). The product was obtained as a white solid (35.8 mg, 52%; 23% over two steps). ^1^H NMR (500 MHz, DMSO) δ 9.86 (s, 1H), 7.50 (d, *J* = 8.4 Hz, 2H), 7.29–7.24 (m, 2H), 7.21–7.11 (m, 5H), 3.87 (s, 2H), 2.95 (dd, *J* = 21.6, 10.5 Hz, 1H), 2.00–1.89 (m, 1H), 1.52–1.34 (m, 2H), 0.85 (d, *J* = 6.1 Hz, 6H). ^13^C NMR (126 MHz, DMSO) δ 167.7, 141.6, 137.5, 135.8, 128.9, 128.6, 128.4, 125.9, 119.2, 46.0 (d, *J* = 127.0 Hz), 40.5, 35.8, 26.5 (d, *J* = 14.5 Hz), 23.2, 21.4. ^31^P NMR (202 MHz, DMSO) δ 20.03. HRMS (ESI^–^) calculated for C_19_H_23_NO_4_P^–^ [M-H]^−^ 360.1370, found 360.1375.

#### [1-({4-[(3,4-Dichlorobenzyl)­thio]­phenyl}­amino)-4-methyl-1-oxopentan-2-yl]­phosphonic Acid (**191**)

Compound **191** was synthesized over two steps according to the general procedure C using **182** (142 mg, 0.31 mmol) and triethyl phosphite (1.5 mL). The resultant oil was purified by column chromatography (Hex/EtOAc = 8/2 to 3/7) to give diethyl phosphonate as transparent oil (84 mg, 53%). The product obtained was then treated with bromotrimethylsilane (106 μL, 0.80 mmol) in DCM (3 mL). The TMS ester was cleaved using MeOH (3 mL). Solvents were concentrated *in vacuo*, and the resultant oil was purified by preparative HPLC (CH_3_CN (HCOOH 0.05%)/H_2_O (HCOOH 0.05%) = 1/9 to 10/0). The product was obtained as a white solid (65 mg, 87%; 46% over two steps). ^1^H NMR (500 MHz, DMSO) δ 9.99 (s, 1H), 7.53 (dd, *J* = 9.3, 7.5 Hz, 4H), 7.32–7.17 (m, 3H), 4.15 (s, 2H), 2.95 (ddd, *J* = 22.4, 11.3, 2.6 Hz, 1H), 2.01–1.88 (m, 1H), 1.52–1.35 (m, 2H), 0.85 (d, *J* = 6.4 Hz, 6H). ^13^C NMR (126 MHz, DMSO) δ 167.9 (d, *J* = 4.6 Hz), 139.6, 138.5, 130.9, 130.8, 130.7, 130.4, 129.4, 129.1, 127.7, 119.5, 46.1 (d, *J* = 126.3 Hz), 36.6, 35.7, 26.4 (d, *J* = 14.7 Hz), 23.2, 21.4. ^31^P NMR (202 MHz, DMSO) δ 19.68. HRMS (ESI^–^) calculated for C_19_H_21_Cl_2_NO_4_PS^–^ [M-H]^−^ 460.0311, found 460.0317.

#### [1-({4-[(3,4-Dichlorobenzyl)­oxy]­phenyl}­amino)-4-methyl-1-oxopentan-2-yl]­phosphonic Acid (**192**)

Compound **192** was synthesized over two steps according to the general procedure C using **183** (102 mg, 0.23 mmol) and triethyl phosphite (1 mL). The resultant oil was purified by column chromatography (Hex/EtOAc = 8/2 to 2/8) to give diethyl phosphonate as transparent oil (47 mg, 41%). The product obtained was then treated with bromotrimethylsilane (60 μL, 0.44 mmol) in DCM (2 mL). The TMS ester was cleaved using MeOH (2 mL). Solvents were concentrated *in vacuo*, and the resultant oil was purified by preparative HPLC (CH_3_CN (HCOOH 0.05%)/H_2_O (HCOOH 0.05%) = 1/9 to 10/0). The product was obtained as a white solid (20.5 mg, 53%; 22% over two steps). ^1^H NMR (500 MHz, DMSO) δ 9.80 (s, 1H), 7.70 (d, *J* = 1.9 Hz, 1H), 7.65 (d, *J* = 8.3 Hz, 1H), 7.51 (d, *J* = 9.0 Hz, 2H), 7.42 (dd, *J* = 8.3, 1.9 Hz, 1H), 6.94 (d, *J* = 9.0 Hz, 2H), 5.08 (s, 2H), 2.92 (dd, *J* = 21.5, 10.5 Hz, 1H), 2.00–1.88 (m, *J* = 10.9, 4.5 Hz, 1H), 1.53–1.34 (m, 2H), 0.86 (d, *J* = 6.4 Hz, 6H). ^13^C NMR (126 MHz, DMSO) δ 167.4 (d, *J* = 1.4 Hz), 153.6, 138.6, 133.2, 131.1, 130.7, 130.3, 129.4, 127.8, 120.5, 114.8, 67.8, 45.9 (d, *J* = 127.3 Hz), 35.8, 26.4 (d, *J* = 14.9 Hz), 23.2, 21.4. ^31^P NMR (202 MHz, DMSO) δ 20.11. HRMS (ESI^–^) calculated for C_19_H_21_Cl_2_NO_5_P^–^ [M-H]^−^ 444.0540, found 444.0545.

#### 1-[4-(3,4-Dichlorophenylthio)­phenylcarbamoyl]-3-methylbutylphosphonic Acid (**90**)

Compound **90** was synthesized according to general procedure C (step 2), using **76** (96 mg, 0.19 mmol), bromotrimethylsilane (250 μL, 1.9 mmol) and DCM (15 mL). The reaction was stirred at r.t. overnight. The crude product was purified using preparative HPLC (CH_3_CN (HCOOH 0.05%)/H_2_O (HCOOH 0.05%) = 1/9 to 10/0). The product was obtained as a white solid (35 mg, 41%). ^1^H NMR (500 MHz, DMSO) δ ppm: 10.22 (s, 1H), 7.71 (d, *J* = 8.7 Hz, 2H), 7.53 (d, *J* = 8.5 Hz, 1H), 7.45 (d, *J* = 8.7 Hz, 2H), 7.32 (d, *J* = 2.2 Hz, 1H), 7.03 (dd, *J* = 8.5, 2.2 Hz, 1H), 3.00 (ddd, *J* = 22.5, 11.3, 2.6 Hz, 1H), 2.05–1.82 (m, 1H), 1.58–1.22 (m, 2H), 0.85 (d, *J* = 6.4 Hz, 6H). ^13^C NMR (126 MHz, DMSO) δ ppm: 168.7 (d, *J* = 4.9 Hz), 141.0, 139.8, 135.4, 132.3, 131.6, 128.8, 128.8, 127.8, 124.3, 120.7, 46.6 (d, *J* = 126.3 Hz), 36.1 (d, *J* = 4.0 Hz), 26.9 (d, *J* = 14.7 Hz), 23.7, 21.8. ^31^P NMR (202 MHz, DMSO) δ ppm: 19.52. HRMS (ESI^–^) calculated for C_18_H_19_Cl_2_NO_4_PS [M-H]^−^ 446.0155, found 446.0157.

#### 1-[4-(3,4-Dichlorophenoxy)­phenylcarbamoyl]-3-methylbutylphosphonic Acid (**91**)

Compound **91** was synthesized according to general procedure C (step 2), using **77** (93 mg, 0.19 mmol), bromotrimethylsilane (250 μL, 1.9 mmol) and DCM (15 mL). The reaction was stirred at r.t. overnight. The crude product was purified using preparative HPLC (CH_3_CN (HCOOH 0.05%)/H_2_O (HCOOH 0.05%) = 1/9 to 10/0). The product was obtained as a white solid (25 mg, 30%). ^1^H NMR (500 MHz, DMSO) δ ppm: 10.05 (s, 1H), 7.67 (d, *J* = 8.9 Hz, 2H), 7.59 (d, *J* = 8.9 Hz, 1H), 7.22 (d, *J* = 2.8 Hz, 1H), 7.06 (d, *J* = 8.9 Hz, 2H), 6.95 (dd, *J* = 8.9, 2.8 Hz, 1H), 2.98 (ddd, *J* = 22.4, 11.3, 2.6 Hz, 1H), 1.98 (tdd, *J* = 11.8, 7.3, 4.2 Hz, 1H), 1.55–1.37 (m, 2H), 0.88 (d, *J* = 6.3 Hz, 6H). ^13^C NMR (126 MHz, DMSO) δ ppm: 168.2 (d, *J* = 4.7 Hz), 157.8, 150.7, 136.8, 132.4, 132.0, 125.1, 121.2, 120.6, 119.7, 118.2, 46.5 (d, *J* = 126.9 Hz), 36.2 (d, *J* = 4.0 Hz), 26.9 (d, *J* = 14.8 Hz), 23.7, 21.8.

^31^P NMR (202 MHz, DMSO) δ ppm: 19.95. HRMS (ESI^–^) calculated for C_18_H_19_Cl_2_NO_5_P [M-H]^−^ 430.0383, found 430.0382.

#### 1-[4-(3,4-Dichlorophenylcarbamoyl)­phenylcarbamoyl]-3-methylbutylphosphonic Acid (**93**)

Compound **93** was synthesized according to general procedure C (step 2), using **79** (98 mg, 0.19 mmol), bromotrimethylsilane (250 μL, 1.9 mmol) and DCM (15 mL). The reaction was stirred at r.t. overnight. The crude product was purified using preparative HPLC (CH_3_CN (HCOOH 0.05%)/H_2_O (HCOOH 0.05%) = 1/9 to 10/0). The product was obtained as white solid (22 mg, 25%). ^1^H NMR (500 MHz, DMSO) δ ppm: 10.34 (s, 2H), 8.13 (d, *J* = 2.4 Hz, 1H), 7.90 (d, *J* = 8.8 Hz, 2H), 7.81–7.64 (m, 3H), 7.58 (d, *J* = 8.8 Hz, 1H), 3.02 (dd, *J* = 22.2, 10.6 Hz, 1H), 1.95 (ddd, *J* = 15.4, 10.0, 3.4 Hz, 1H), 1.52–1.34 (m, 2H), 0.85 (s, 3H), 0.83 (s, 3H). ^13^C NMR (126 MHz, DMSO) δ ppm: 169.0 (d, *J* = 6.0 Hz), 165.6, 143.3, 139.9, 131.3, 131.0, 129.2, 128.5, 125.3, 121.8, 120.6, 118.6, 46.7 (d, *J* = 127.3 Hz), 36.2 (d, *J* = 4.1 Hz), 27.0 (d, *J* = 14.3 Hz), 23.7, 21.8. ^31^P NMR (202 MHz, DMSO) δ ppm: 19.39. HRMS (ESI^–^) calculated for C_19_H_20_Cl_2_N_2_O_5_P [M-H]^−^ 457.0492, found 457.0493.

#### 1-[4-(3,4-Dichlorophenylsulfamoyl)­phenylcarbamoyl]-3-methylbutylphosphonic Acid (**92**)

Compound **92** was synthesized according to general procedure C (step 2), using **78** (105 mg, 0.19 mmol), bromotrimethylsilane (250 μL, 1.9 mmol) and DCM (15 mL). The reaction was stirred at r.t. overnight. The crude product was purified using preparative HPLC (CH_3_CN (HCOOH 0.05%)/H_2_O (HCOOH 0.05%) = 1/9 to 10/0). The product was obtained as a white solid (42 mg, 45%). ^1^H NMR (500 MHz, Acetone) δ ppm: 9.88 (s, 1H), 9.33 (s, 1H), 7.85 (d, *J* = 8.7 Hz, 2H), 7.78 (d, *J* = 8.7 Hz, 2H), 7.45 (t, *J* = 6.0 Hz, 2H), 7.25–7.19 (m, 1H), 3.25–3.08 (m, 1H), 2.22–2.10 (m, 1H), 1.65–1.54 (m, 2H), 0.87 (d, *J* = 14.3 Hz, 6H). ^13^C NMR (126 MHz, Acetone) δ ppm: 168.0 (d, *J* = 5.0 Hz), 143.4, 138.2, 133.6, 132.2, 131.1, 128.3, 127.0, 121.6, 120.0, 119.2, 46.4 (d, *J* = 128.8 Hz), 35.5 (d, *J* = 5.0 Hz), 26.7 (d, *J* = 14.5 Hz), 22.5, 20.8. ^31^P NMR (202 MHz, Acetone) δ ppm: 23.91. HRMS (ESI^–^) calculated for C_18_H_20_Cl_2_N_2_O_6_PS [M-H]^−^ 493.0162, found 493.0161.

#### 1-{4-[(3,4-Dichlorophenylthio)­methyl]­phenylcarbamoyl}-3-methylbutylphosphonic Acid (**164**)

Compound **164** was synthesized according to general procedure C (step 2), using **163** (98 mg, 0.19 mmol), bromotrimethylsilane (250 μL, 1.9 mmol) and DCM (15 mL). The reaction was stirred at r.t. overnight. The crude product was purified using preparative HPLC (CH_3_CN (HCOOH 0.05%)/H_2_O (HCOOH 0.05%) = 1/9 to 10/0). The product was obtained as white solid (16 mg, 18%). ^1^H NMR (500 MHz, DMSO) δ ppm: 9.98 (s, 1H), 7.60 (d, *J* = 2.2 Hz, 1H), 7.54 (dd, *J* = 8.5, 4.6 Hz, 3H), 7.32–7.24 (m, 3H), 4.27 (s, 2H), 2.97 (ddd, *J* = 22.4, 11.3, 2.7 Hz, 1H), 1.96 (tdd, *J* = 11.6, 7.2, 4.1 Hz, 1H), 1.51–1.32 (m, 2H), 0.86 (d, *J* = 6.4 Hz, 6H). ^13^C NMR (126 MHz, DMSO) δ ppm: 168.3 (d, *J* = 5.6 Hz), 139.1, 138.2, 132.0, 131.4, 131.2, 129.6, 129.5, 128.5, 128.4, 119.4, 46.5 (d, *J* = 126.7 Hz), 40.5, 36.2 (d, *J* = 16.6 Hz), 26.9 (d, *J* = 15.0 Hz), 23.7, 21.8. ^31^P NMR (202 MHz, DMSO) δ ppm: 19.89. HRMS (ESI^–^) calculated for C_19_H_21_Cl_2_NO_4_PS [M-H]^−^ 460.0311, found 460.0311.

#### 1-{4-[(3,4-Dichlorophenylamino)­methyl]­phenylcarbamoyl}-3-methylbutylphosphonic Acid (**169**)

Compound **169** was synthesized according to general procedure C (step 2), using **168** (115 mg, 0.19 mmol), bromotrimethylsilane (250 μL, 1.9 mmol) and DCM (15 mL). The reaction was stirred at r.t. overnight. The crude product was purified using preparative HPLC (CH_3_CN (HCOOH 0.05%)/H_2_O (HCOOH 0.05%) = 1/9 to 10/0). The product was obtained as white solid (31 mg, 37%). ^1^H NMR (500 MHz, Acetone) δ ppm: 9.56 (s, 1H), 7.50 (dd, *J* = 8.4, 1.6 Hz, 2H), 7.16 (d, *J* = 8.4 Hz, 2H), 7.06 (d, *J* = 8.8 Hz, 1H), 6.67 (d, *J* = 2.7 Hz, 1H), 6.49 (dd, *J* = 8.8, 2.7 Hz, 1H), 4.18 (s, 2H), 3.16–3.00 (m, 1H), 2.08–1.94 (m, 1H), 1.46 (dd, *J* = 11.9, 5.9 Hz, 2H), 0.74 (d, *J* = 6.1 Hz, 3H), 0.73 (d, *J* = 6.1 Hz, 3H). ^13^C NMR (126 MHz, Acetone) δ ppm: 167.8 (d, *J* = 4.7 Hz), 148.9–147.7 (m), 138.9–136.7 (m), 135.1–133.3 (m), 131.9, 130.5, 127.7, 119.7, 118.0, 113.6, 113.0, 46.6, 46.2 (d, *J* = 128.8 Hz), 35.8 (d, *J* = 3.9 Hz), 26.8 (d, *J* = 14.7 Hz), 22.6, 21.0. ^31^P NMR (202 MHz, Acetone) δ ppm: 24.39. HRMS (ESI^–^) calculated for C_19_H_22_Cl_2_N_2_O_4_P [M-H]^−^ 443.0670, found 443.0697.

### *In Vitro* Inhibition Assays (LasB, MMPs, TACE, anad COX-1)

All *in vitro* inhibition assays were performed as described previously.^12^ The TACE inhibitor screening kit were purchased from Sigma-Aldrich (Saint Louis, MO), the COX-1 kit from Abcam (Cambridge, UK). MMPs along with the SensoLyte 520 Generic MMP Activity Kit Fluorimetric were purchased from AnaSpec (Fremont, CA, USA), and the fluorometric cyclooxygenase 1 (COX1) inhibitor assay kit was purchased from Abcam (Cambridge, UK). The assays were performed according to the guidelines of the respective manufacturer. Fluorescence signals were measured using a CLARIOstar plate reader (BMG Labtech, Ortenberg, Germany). Pulmonary surfactant (poractant alfa), which is an extract of natural porcine lung surfactant, was purchased from Creative BioMart (Shirley, NY, USA). DPPC was purchased from Lipoid (Ludgwigshafen, Germany). LasB was expressed as described previously.^12,30^

### Murine Survival Model with Instilled LasB

Procedures involving mice were approved by the local ethical committee (Paris-Nord/No 121) and by the French Ministry of Education and Research (agreement number 28050–2020102015484235). Eight-week-old male C57BL/6 mice were purchased from Janvier (Le Genest-Saint-Isle, France. Mice were anaesthetised using intramuscular injection of ketamine 500 and xylazine 2% in 0.9% NaCl (20/10/70). 40 μg of purified LasB were instilled intranasally in a final volume of 40 μL. In groups with LasB inhibitor treatment, LasB was preincubated with inhibitors at a 1/10 molar ratio at 37 °C for 1 h before intranasal instillation (300 μM of compound vs 30 μM of enzyme). Finally, the survival of the mice was monitored over 7 d.

### Lipophilicity Determination (logD_7.4_)

LogD_7.4_ was analyzed using an HPLC-based method. The UV retention time of reference compounds with known logD_7.4_ was determined and plotted toward their logD_7.4_. Linear regression was used to determine LogD_7.4_ of unknown compounds. Analysis was performed using a Vanquish Flex HPLC system with variable wavelength detector (Thermo Fisher, Dreieich, Germany) with the following conditions: EC150/2 NUCLEODUR C18 Pyramid column, 5 μM (Macherey Nagel, Düren, Germany); eluent A: 50 mM NH_4_OAc pH 7.4, eluent B: acetonitrile, and flow: 0.6 mL/min. The gradient was set to 0–100% B from 0 to 2.5 min, 100% B from 2.5 to 3.0 min, 100–0% B from 3.0 to 3.2 min, and 0% B from 3.2–5.0.

### X-ray Crystallography

For LasB cocrystallization trials, the protein was purified from culture supernatant of *P. aeruginosa* PA14 (for **35** and **141**), or recombinantly expressed in *Eschierichia coli* (for **21**), as described previously.^12,30^ The amino acid sequence of the recombinantly expressed LasB differs from PA14 in four positions and corresponds to the LasB sequence of *P. aeruginosa* PAO1. There is no difference between both protein variants in terms of protein activity and susceptibility to inhibitors, and the four mutations are not located in the vicinity of the LasB active (compound binding) site.^30^ The Met128Val variant of LasB was constructed via Gibson assembly using the primers listed in Table S7, and the protein was recombinantly expressed in *E. coli* and purified under the same reported conditions as the wild type. LasB was concentrated to ∼ 5 mg/mL after size-exclusion chromatography using a HiLoad Superdex S200 16/600 column and incubated with a final concentration of 1 mM (600 μM for **35**) compound in 10 mM Tris pH 8.0 and 2 mM CaCl_2_ for 1 h on ice prior to crystallization. Co-crystallization trials for all compounds were carried out using commercially available screens (NeXtal) and prepared in sitting-drop SwissSCI plates by a Gryphon crystallization robot. Crystals were observed in a variety of conditions after approximately one moth incubation at 293 K. Crystals of wild-type LasB in complex with **21** appeared in a well solution of 0.1 M sodium citrate pH 5.5 and 35% (v/v) 2-ethoxyethanol and cocrystals of the LasB Met128Val variant were obtained in the condition 0.1 M sodium chloride, 0.1 M HEPES pH 7.5, 1.6 M ammonium sulfate. Diffraction quality cocrystals for **141** were observed in the condition 0.01 M trisodium citrate and 33% (w/v) PEG 6000 and crystals for **35** in complex with LasB appeared in a well solution of 0.1 M HEPES pH 6.5 and 2.4 M ammonium sulfate. Protein crystals were cryo-protected with 32% glycerol and flash-frozen in liquid nitrogen. Diffraction data were collected at 100 K using ESRF beamline ID23–1 for LasB in complex with **35** and at beamline P11 at Petra III (DESY) for the other tree compounds.^39^ Data were processed using Aimless and Pointless, both implemented in CCP4 and the structures were determined by molecular replacement using PHASER.^40–42^ Structures 8CR3, 1EZM and 6FZX were used as search models.^30,43,44^ Further processing of the data was performed using AutoBuild, the structure was manually rebuilt in COOT and the refinement was carried out using Phenix.refine.^45,46^ The refined structures of LasB in complex with **21**, **141** and **35**, as well as the LasB Met128Val structure in complex with **21** were deposited in the Protein Data Bank (PDB) as entries 9FRY, 9GMV, 9FRZ and 9FS0, respectively. Figures were generated in PyMOL by Schrödinger (version 3.1.6.1).

### Culturing of HepG2, A549, and HEK293 Cells

The human hepatocellular carcinoma cell line HepG2, the lung adenocarcinoma cell line A549 and Human Embryonic Kidney (HEK) 293 cells were cultured in Dulbecco’s Modified Eagle Medium (DMEM), containing 10% Fetal Bovine Serum (FBS) and 1% penicillin-streptomycin mixture. Cells were maintained according to standard cell-culture procedures.

### Cytotoxicity Assay

An MTT-based assay was employed to evaluate the viability of HepG2, HEK293 and A549 cells after challenge with selected LasB inhibitors and performed as described previously.^47^

### Metabolic and Plasma Stability

Murine liver S9 and plasma stability as well as profiling of stability in mouse, rat and minipig liver microsomes and plasma was performed as described previously.^19^

### Calu-3 Permeability

Compound permeability was assessed *in vitro* with Calu-3 HTB-55 cell line (ATCC) as described previously.^19^

### Protein Binding Assays

PPB was determined using the Rapid Equilibrium Dialysis (RED) system (Thermo Fisher Scientific, Waltham MA, USA). Compounds were diluted to 10 μM in murine (CD-1) plasma (Neo Biotech, Nanterre, France) and added to the respective chamber according to the manufacturer’s protocol, followed by addition of PBS pH 7.4 to the opposite chamber. Samples were taken immediately after addition to the plate as well as after 2, 4, and 5 h by mixing 10 μL with 80 μL ice-cold acetonitrile containing 12.5 nM diphenhydramine as internal standard, followed by addition of 10 μL plasma to samples taken from PBS and *vice versa*. Samples were stored on ice until the end of the incubation, and precipitated protein was removed by centrifugation (15 min, 4 °C, 4,000 g, 2 centrifugation steps). Concentration of the remaining test compound at the different time points was analyzed by HPLC-MS/MS (TSQ Quantum Access MAX, Thermo Fisher, Dreieich, Germany). The amount of compound bound to protein was calculated using the equation PPB [%] = 100–100­(amount in buffer chamber/amount in plasma chamber).

Surfactant protein binding was determined similarly using 1% porcine lung surfactant in FRET assay buffer instead of plasma. The mixture was incubated for 8 h.

### Bacterial Growth Inhibition Assay

Assays regarding the determination of the MIC were performed as described recently for *P. aeruginosa* PA14.^48^

### Production of Culture Supernatants

The culture supernatants of wild type PAO1 and PAO1 Δ*lasB* as well as the strains used for the screening across isolates were produced as previously reported.

### Biological Evaluation of 35 via an A549 Cell-Based Assay

The A549 human lung adenocarcinoma cell line was cultivated and maintained in Dulbecco’s Modified Eagle Medium (DMEM) supplemented with 10% Fetal bovine serum (FBS) and 1% penicillin-streptomycin mixture, following standard cell-culture protocols.

2.5 × 10^3^ cells per well were seeded into a 96-well flat-bottom plate (Corning Costar) and incubated at 37 °C with 5% CO_2_ for 24 h. The next day, various concentrations of the compound **35** were tested against 10% (v/v) *P. aeruginosa* PAO1 (DSM 22644, ATCC 15692) culture supernatant, which was diluted in DMEM, starting from 5 μM. Initially, the compound was dissolved in 99.9% dimethyl sulfoxide (DMSO), and a final assay concentration of 0.5% (v/v) DMSO was applied. Furthermore, 10% (v/v) Δ*lasB* PAO1 was introduced to the cells to verify the toxic effect attributed to LasB. A control group was included, consisting of cells treated solely with DMEM, without any additional agents. Subsequently, the plates were incubated at 37 °C with 5% CO_2_ for 24 h before conducting the MTT assay.

### MTT Assay

To assess cell viability, the content of the wells was aspirated, followed by a single wash with 100 μL of phosphate buffered saline (PBS) at pH 7.4. A solution of 5 mg of MTT (3-(4,5-dimethylthiazol-2-yl)-2,5-diphenyltetrazolium bromide) per milliliter of PBS was prepared and then diluted to a 10% (v/v) concentration in DMEM. Subsequently, 100 μL of the MTT solution was added to each well, and the plates were incubated at 37 °C for 2 h with 5% CO_2_. Following the incubation period, the MTT solution was carefully aspirated, and the formed crystals were dissolved by the addition of 150 μL per well of MTT lysis buffer (composed of 250 mL DMSO, 25 g SDS, and 1.25 mL acetic acid). The plates were once again incubated for 30 min at 37 °C with 5% CO_2_ before the measurement was performed using a PHERAstar microplate reader. The optical density was measured at 550 nm for the test samples and at 620 nm for the blank. Finally, the obtained data were statistically analyzed and graphically presented using GraphPad Prism 9.

### Assessment of 35 Inhibitory Effects Against Clinical Isolates of *P. aeruginosa* Using a FRET-Based Inhibition Assay

Bacterial culture supernatants were prepared from eight clinical isolates of *P. aeruginosa* following overnight growth in lysogeny broth medium (LB). After approximately 18 h of growth, the cultures were centrifuged for 10 min at 5,000 rpm and filtered through 0.2 μm nonpyrogenic sterile filters. The fluorogenic substrate 2-Aminobenzoyl-Ala-Gly-Leu-Ala-4-nitrobenzylamide, sourced from Peptides International (Louisville, KY, USA), was employed in this study.

Fluorescence intensity measurements were conducted at 37 °C for a duration of 60 min using a CLARIOstar microplate reader (BMG Labtech, Ortenberg, Germany). The excitation and emission wavelengths utilized were 340 ± 15 nm and 415 ± 20 nm, respectively. The measurements were performed in black 384-well microtiter plates from Greiner Bio-One (Kremsmünster, Austria). The experimental setup involved a final volume of 50 μL, which included varying concentrations of **35** dissolved in DMSO and mixed with assay buffer (comprising 50 mM Tris, pH 7.2, 2.5 mM CaCl_2_, 0.075% Pluronic F-127, and 5% DMSO), along with bacterial culture supernatant at a concentration where linear LasB activity was observed, and the substrate at a final concentration of 150 μM.

Each sample was duplicated in the multiwell plate, and controls without supernatant/compound were included to facilitate blank correction. After subtracting the blank values, the FRET signal of the samples was plotted at various time points, with measurements taken every 2 min, using GraphPad Prism 9.

### Zebrafish Embryo Toxicity

The experiment was performed according to a procedure described in the literature^49^ with minor modifications using zebrafish embryos of the AB wild-type line at 2 days post fertilization (dpf). A detailed protocol has been given in our recent publication.^50^

### *In Vivo* Pharmacokinetic Studies

For pharmacokinetic experiments, outbred male CD-1 mice (Charles River, Netherlands), 4 weeks old, were used. The animal studies were conducted in accordance with the recommendations of the European Community (Directive 2010/63/EU, first January 2013). All animal procedures were performed in strict accordance with the German regulations of the Society for Laboratory Animal Science (GV- SOLAS) and the European Health Law of the Federation of Laboratory Animal Science Associations (FELASA). Animals were excluded from further analysis if sacrifice was necessary according to the human end points established by the ethical board. All experiments were approved by the ethical board of the Niedersächsisches Landesamt für Verbraucherschutz and Lebensmittelsicherheit, Oldenburg, Germany.

Compounds **23**, **141**, **21**, **130**, **81**, **82** and **195** were dissolved in 2% DMSO, 1% Tyloxapol and 97% PBS. The compounds were administered intratracheally (IT) at 0.25 mg/kg per compound as cassette. Up to 5 compounds were dosed per cassette. Before administration, mice were anesthetized using ketamine 100 mg/kg and xylazine 10 mg/kg intraperitoneally. Mice (*n* = 3 per time point) were euthanatized at t= 0.5, 2, and 5 h post administration. Blood was collected from the heart. **207**, **21**, **141**, **81**, **82**, **130**, **209**, **212**, **211**, **206**, **35** and **138** were administered at 2 mg/kg intravenously per compound as cassette. Up to 5 compounds were dosed per cassette intravenously, n = 2 mice were used per cassette. Blood was collected from the lateral tail vein at time points t= 0.25, 0.5, 1, and 3 h post administration. At 5 h post administration, mice were euthanatized, and blood was collected from the heart. **35** was subjected to a focused PK study and was administered 30 mg/kg SC. At time points t= 0.25, 0.5, 1, 2, 4, 8, and 24 h post administration, mice (t= 3 mice per time point) were euthanatized and blood was collected from the heart. For all PK studies, whole blood was collected into Eppendorf tubes coated with 0.5 M EDTA and immediately spun down at 13,000 rpm at 4 °C for 10 min. The plasma was transferred into a new Eppendorf tube and then stored at −80 °C until analysis. Then, a broncheoalveolar lavage was conducted using isotonic sodium chloride solution for all PK studies. For all PK studies, lung, kidney and liver were aseptically removed and homogenized using a Polytron (Kinematica) in isotonic sodium chloride solution. Organ samples were aliquoted into Eppendorf tubes and stored at −80 °C until analysis. Moreover, spontaneous urine was also collected.

All PK plasma samples were analyzed via HPLC-MS/MS using an Agilent 1290 Infinity II HPLC system and coupled to an AB Sciex QTrap6500+ mass spectrometer as described previously.^19^ Mass spectrometric conditions can be found in Table S8. Urea was used to enable calculation of epithelial lining fluid (ELF) concentrations. Peak areas of each sample and of the corresponding internal standard were analyzed using MultiQuant 3.0 software (AB Sciex). Peak areas of the respective sample were normalized to the internal standard peak area. Peaks of PK samples were quantified using the calibration curve. The accuracy of the calibration curve was determined using QCs independently prepared on different days. PK parameters were determined using a noncompartmental analysis with PKSolver.^51^ ELF concentrations were calculated using the following formula:^2,52^

$$V_{ELF}=V_{BALF}\times \frac{Urea_{BALF}}{Urea_{Plasma}}$$ 1

$$c_{ELF}=c_{BALF}\times \frac{V_{BALF}}{V_{ELF}}$$ 2

## Supplementary Material





## Notes

Supplementary Tables: Off-target, MIC and further cytotoxicity assays for frontrunner compounds, *in vitro* metabolism species profiling of **35**, zebrafish toxicity assays for **23**, **35**, **141**, PK parameters after IT dosing, inhibition assays with culture supernatants, data collection and refinement statistics for X-ray crystallography, primers used for expression of LasB mutants, MS conditions for *in vivo* bioanalysis; Supplementary Figures: additional X-ray cocrystal structures, LigPlot^+^ diagrams and polder maps, IV cassette PK urine and plasma levels, correlation of logD_7.4_ with PPB; additional experimental data and HPLC traces of all final compounds (PDF)
Molecular formula strings for all final compounds (CSV)

## #

College of Pharmacy, Al-Farahidi University, Baghdad, 10021, Iraq

## +

Innovation Campus Berlin (Nuvisan ICB GmbH), Berlin 13353, Germany

## §

Environmental Health Research Division, National Institute of Environmental Research­(NIER), Incheon, 22689, Republic of Korea

## Abbreviations

CF: cystic fibrosis
csn: culture supernatant
DPPC: dipalmitoylphosphatidylcholine
ELF: epithelial lining fluid
ESKAPE: *Enterococcus faecium* , *Escherichia coli, Staphylococcus aureus*, *Klebsiella pneumoniae* , *Acinetobacter baumannii*, *Pseudomonas aeruginosa* , Enterobacter
HAP: hospital-acquired pneumonia
ICU: intensive-care unit
LasB: elastase
NCFB: noncystic fibrosis bronchiectasis
SOC: standard-of-care
TACE: tumor-necrosis factor α-converting enzyme
TLP: target-lead profile
TPP: target-product profile
VAP: ventilator-associated pneumonia
