# Supplementary material for: Multiparameter Optimization of Pseudomonas aeruginosa Elastase Inhibitors for Systemic Administration
Source: J Med Chem. 2026 Feb 5;69(4):4160–86. doi: 10.1021/acs.jmedchem.5c02788 (PMC12951441; doi:10.1021/acs.jmedchem.5c02788)
Supplement: Supplementary file 1 [file jm5c02788_si_001.pdf]

Supporting Information

Multiparameter Optimization of *Pseudomonas aeruginosa* Elastase (LasB) Inhibitors for Systemic Administration

Ahmed S. Abdelsamie[1,2, #], Jelena Konstantinović[1,2, +], Andreas M. Kany[1,2,3], Christian Schütz[1,2], Dominik Kolling[1,2,4,5], Samira Speicher[1,2], Andreas Klein[1,2], Roya Shafiei[1,2], Mélodie Bouté[6], Katharina Mundry[4], Yu Mi Park[1,2, ~], Brigitta Loretz[1,2], Rolf Müller[1,2,3,5,7], Jean-Michel Sallenave[6, 8], Claus-Michael-Lehr[1,2,5], Jesko Koehnke[4], Katharina Rox[3,9], Jörg Haupenthal[1,2,3] and Anna K. H. Hirsch[1,2,3,5,7]*

[1] Helmholtz Institute for Pharmaceutical Research Saarland (HIPS) – Helmholtz Centre for Infection Research (HZI), Saarbrücken 66123, Germany

[2] PharmaScienceHub, Saarbrücken 66123, Germany

[3] Deutsches Zentrum für Infektionsforschung (DZIF) e.V., Partner site Braunschweig-Hannover, Braunschweig 38124, Germany

[4] Institute for Food Chemistry, Hannover 30167, Germany

[5] Department of Pharmacy, Saarbrücken 66123, Germany

[6] Laboratoire d’Excellence Inflamex, Institut National de la Santé et de la Recherche Medicale U1152, Physiopathologie et Épidémiologie des Maladies Respiratoires, Université Paris-Cité, 75006 Paris, France

[7] Helmholtz International Lab for Anti-infectives, Saarbrücken 66123, Germany

[8] INSERM U1149, Centre de Recherche sur l'Inflammation, Hôpital Bichat, Université Paris-Cité, 16 rue Henri Huchard, 75018 Paris, France

[9] Department of Chemical Biology (CBIO), Helmholtz Centre for Infection Research (HZI), Braunschweig 38124, Germany

[#] present address: College of Pharmacy, Al-Farahidi University, Baghdad, 10021, Iraq

[+] present address: Innovation Campus Berlin (Nuvisan ICB GmbH), Berlin 13353, Germany

[~] present address: Environmental Health Research Division, National Institute of Environmental Research(NIER), Incheon, 22689, Republic of Korea

*anna.hirsch@helmholtz-hips.de

Contents

Supplementary Tables and Figures S3

Experimental Section S16

Supplementary Tables and Figures

Table S1. Off-target activities of selected compounds against human MMPs1-3, COX-1 and TACE (ADAM17), antibacterial activity against *Pseudomonas aeruginosa* PA14 and cytotoxicity results tested with HepG2 and HEK293.

**Cpd**	**IC_{50} [µM]**					**MIC [µM]**	**Viability inh. @100 µM [%]**	
	**MMP-1**	**MMP-2**	**MMP-3**	**COX-1**	**TACE**	**PA14**	**HepG2**	**HEK293**
**23**	>100	>100	>100	>100	>100	>100	<10	<10
**21**	>100	>100	>100	>100	>100	>100	<10	11±8
**195**	>100	>100	>100	>100	>100	>100	<10	24±7
**90**	>100	>100	>100	>100	>100	>100	<10	48±11
**130**	>100	>100	>100	>100	>100	>100	<10	<10
**141**	>100	>100	>100	>100	>100	>100	<10	<10
**81**	>100	>100	>100	>100	>100	>100	<10	<10
**82**	>100	>100	>100	>100	>100	>100	<10	<10
**138**	>100	>100	>100	>100	>100	>100	<10	<10
**35**	>100	>100	>100	>100	>100	>100	<10	<10
**89**	>100	>100	>100	>100	>100	>100	<10	<10

Table S2. *In vitro* species profiling of compound **35**. Species used were C57BL6 mice, Wistar rat and Göttingen minipig

**Compound**	**Liver Microsome Cl_{int} [µL/mg/min] Mouse / Rat / Minipig**	**Plasma $t_{1/2}$ [min] Mouse / Rat / Minipig**
**35**	<11.6 / <11.6 / <11.6	>240 / >240 / >240

Table S3. Toxicity of selected compounds in zebrafish larvae.

**Compound**	**MTC in zebrafish larvae [µM]**
**23**	>100
**141**	>30
**35**	>100

Table S4. PK Parameters of selected compounds after intratracheal cassette dosing at 0.25 mg/kg per compound.

**PK Parameter**	**23**	**141**	**21**	**130**	**81**	**82**	**195**
C_{max} [ng/mL] Plasma	174.45	-	176.57	33.84	46.38	30.33	49.73
AUC_{0-t} [ng/mL*h] Plasma	470.01	-	798.26	73.38	97.24	64.33	142.33
AUC_{0-t} [ng/mL*h] BALF	4,745.62	12,298.65	3937.04	2164.71	794.15	879.33	29,897.76
AUC_{0-t} [µg/mL*h] ELF	93.34	283.5	32.73	17.67	6.46	7.16	238.48
Ratio AUC ELF/Plasma	199	N/A	73	241	66	111	1676

Table S5. LasB activity in different culture supernatants and IC_{50} of **35** against the respective culture supernatant. Dilution factor shows fold-dilution needed to reach linear kinetics of FRET substrate conversion with a slope of the uninhibited control between 10-25. The exact slope is given per culture supernatant.

***P. aeruginosa* strain**	**Dilution factor**	**FRET assay slope DMSO**	**IC_{50} 35 [nM]**
PAO1	1:3125	23.8	16.1 ± 0.95
PA14	1:3125	17.4	10.4 ± 0.45
PA54	1:4096	21	24.6 ± 2.3
RP73	1:16	10	13.9 ± 0.52
NH57388A	1:25	11.7	11.3 ± 0.40
DSM-24600	1:25	26.7	17.4 ± 1.2
DSM-1117	1:125	23.4	21.9 ± 1.4
PA 83979	1:128	26	24.4 ± 1.2

Table S6. Data collection and refinement statistics.

	LasB_**21**	LasB$_{M128V}$_**21**	LasB_**141**	LasB_**35**
PDB code	9FRY	9GMV	9FRZ	9FS0
Data collection				
Space group	P 2_1 2_1 2_1	P 2_1 2_1 2_1	P 1 2_1 1	P 2_1 2_1 2_1
Cell dimensions				
a, b, c [Å]	44.0, 50.4, 125.8	44.2, 50.0, 126.6	44.3, 67.4, 47.5	43.8, 50.0, 125.8
α, β, γ [°]	90.0, 90.0, 90.0	90.0, 90.0, 90.0	90.0, 102.0, 90.0	90.0, 90.0, 90.0
Wavelength [Å]	1.0332	1.0332	1.0332	0.8856
Resolution [Å]	46.8 – 1.35 (1.37 – 1.35)	46.5 – 1.35 (1.37 – 1.35)	38.2 – 1.70 (1.76 – 1.70)	32.9 – 1.30 (1.32 – 1.30)
$CC_{1/2}$	0.999 (0.984)	0.998 (0.715)	0.997 (0.814)	1.000 (0.326)
I / σI	27.4 (10.0)	13.4 (2.3)	11.0 (2.9)	25.7 (6.7)
Completeness [%]	99.9 (99.9)	99.7 (99.8)	99.1 (99.5)	99.9 (100.0)
Redundancy	9.2 (9.1)	7.4 (7.3)	2.0 (2.0)	6.5 (1.2)
Refinement				
Resolution [Å)	46.8 – 1.35	46.5 – 1.35	38.2 – 1.70	43.8 – 1.30
No. reflections	62297 (2084)	62343 (2036)	29866 (2624)	68882 (2826)
R_{work} / R_{free}	0.130 / 0.143	0.146 / 0.155	0.155 / 0.183	0.162 / 0.178
No. atoms	2737	2623	2564	2820
Protein	2328	2316	2309	2325
Ligand/ion	39	38	51	75
Water	370	269	223	420
B-factors	11.52	16.45	19.46	13.72
Protein	9.44	15.14	18.08	11.50
Ligand/ion	11.64	15.81	38.26	14.25
Water	24.59	27.81	31.08	25.90
R.m.s. deviations				
Bond lengths [Å]	0.007	0.005	0.010	0.006
Bond angles [°]	0.99	0.87	1.11	0.91
MolProbity clash score	3.51	1.10	1.34	3.90
Ramachandran [%]				
Favoured	96.62	97.30	96.62	95.95
Allowed	3.38	2.70	3.04	4.05
Outliers	0.00	0.00	0.34	0.00

Statistics for the highest resolution shell are shown in parentheses.

Table S7. Primers used for construction of the pET-26b(+)-LasB-PAO1-Met128Val expression plasmid.

**Primer name**	**Primer sequence**
LasB-PAO1-Met128Val insert Primer forward	AGCCGGCGATGGCCATGGGAGCAGATCTGATT
LasB-PAO1-Met128Val insert Primer reverse	AGGCTAACCAGCGGATAAAA**AAC**GGTGGCACCATC
LasB-PAO1-Met128Val vector Primer forward	TTGGCGATGGTGCCACC**GTT**TTTATCCGCTGGTT
LasB-PAO1-Met128Val vector Primer reverse	CTAACATCAATCAGATCTGCTCCCATGGCCAT

Table S8. Mass spectrometric conditions for *in vivo* bioanalysis.

ID	Q1 Mass [Da]	Q3 Mass [Da]	DP [V]	CE [V]	CXP [V]
**Urea**	60.915	43.8	56.0	17.0	16.0
		43.1	56.0	53.0	12.0
		29.1	56.0	111.0	6.0
**Caffeine**	195.024	138.0	130.0	25.0	14.0
		110.0	130.0	31.0	18.0
**23**	380.926	78.9	-10	-68	-35
		282.9	-10	-20	-15
		362.9	-10	-28	-29
**141**	492.880	208.8	-105	-34	-23
		78.9	-105	-86	-35
		376.8	-105	-32	-17
**21**	379.944	78.9	-75	-22	-9
		362.0	-75	-26	-17
		281.9	-75	-20	-15
**130**	389.965	78.9	-90	-58	-9
		292.0	-90	-20	-15
		372.0	-90	-26	-19
**81**	353.998	78.9	-85	-24	-9
		336.0	-85	-28	-17
		132.9	-85	-42	-13
**82**	368.026	78.9	-70	-24	-21
		350.0	-70	-28	-17
		132.9	-70	-42	-13
**195**	470.915	78.9	-100	-82	-19
		372.8	-100	-24	-19
		452.9	-100	-30	-21
**207**	263.920	78.8	-75	-22	-19
		63.0	-75	-130	-27
		132.9	-75	-36	-13
		185.9	-75	-16	-9
**141**	492.880	208.8	-105	-34	-23
		78.9	-105	-86	-35
		376.8	-105	-32	-17
**209**	294.966	78.9	-50	-22	-9
		197.0	-50	-18	-17
		277.0	-50	-22	-13
**212**	411.125	78.8	-95	-82	-35
		294.9	-95	-40	-13
		392.9	-95	-30	-17
**211**	371.935	78.9	-40	-68	-35
		239.8	-40	-20	-13
		353.8	-40	-26	-17
**206**	337.906	78.9	-125	-22	-9
		239.9	-125	-20	-11
		319.9	-125	-26	-15
**35**	406.111	79.0	-75	-62	-9
		388.0	-75	-36	-21
		307.9	-75	-22	-17
**138**	445.987	78.9	-70	-76	-35
		427.8	-70	-28	-21
		325.8	-70	-22	-17

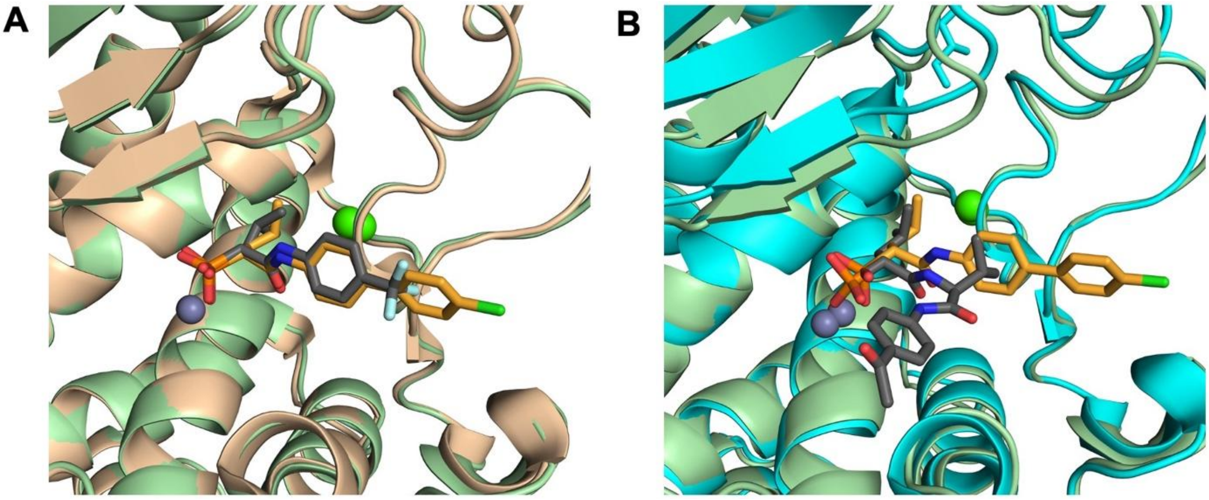

Figure S1. (**A**) Superposition of the crystal structures of LasB (beige) in complex with a phosphonate-based inhibitor (grey) (PDB code: 8CCA) and of LasB (green) in complex with **21** (orange). The active-site Zn^{2+} is shown as a grey sphere. The aromatic ring of both compounds aligns exceptionally well and is engaged in hydrophic interactions in the lipophilic S2' pocket. (**B**) Superposition of the two c-crystal structure of LasB (cyan) in complex with a recently published dipeptide phosphonate inhibitor (gray) and LasB (green) in complex with **21**. The binding mode of the two derivatives differs significantly.

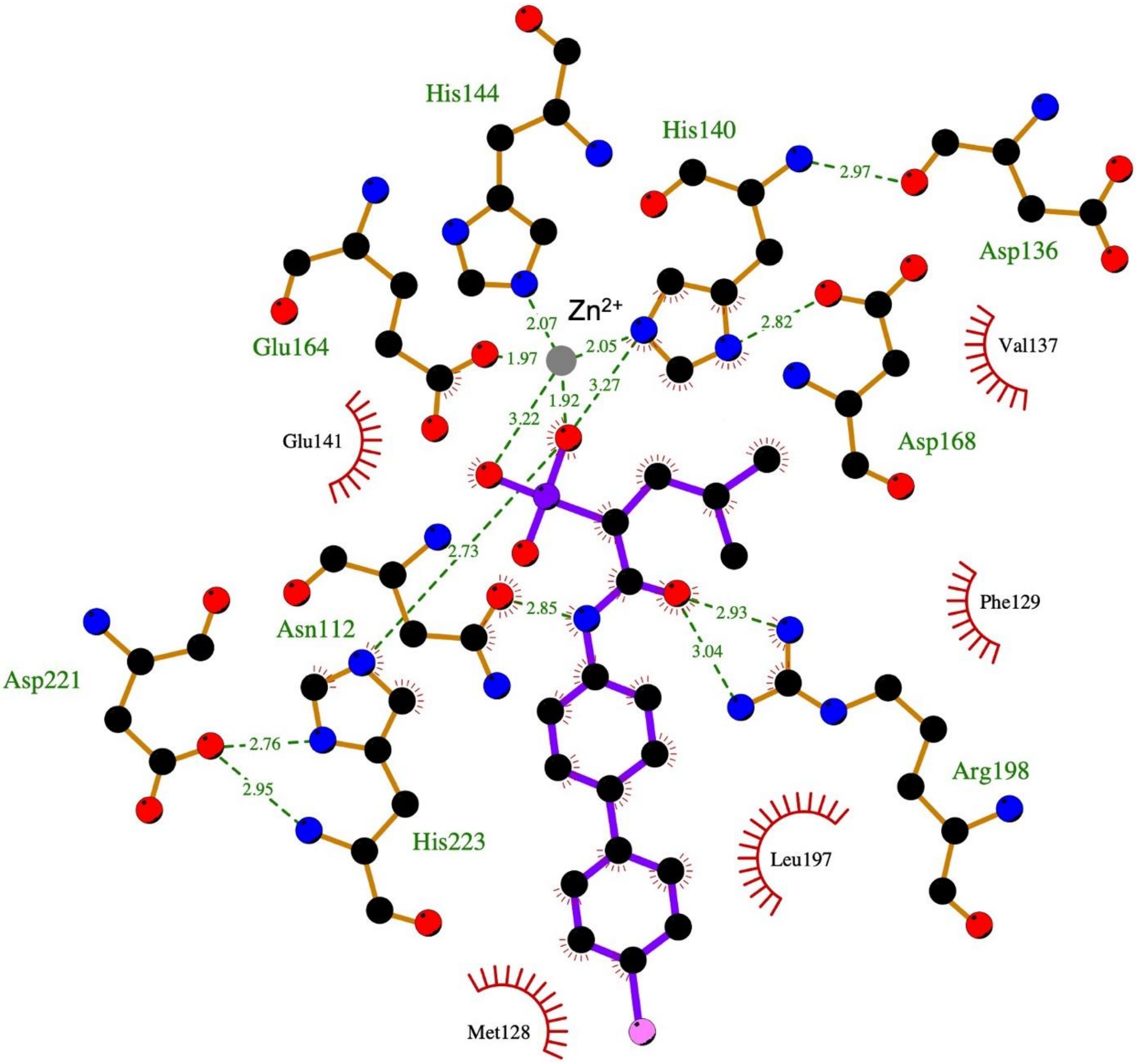

Figure S2. LigPlot+ diagram for the interactions between LasB and **21**. Ligand bonds are represented in purple and protein bonds are colored in dark brown. The distances for hydrogen bonds are shown in Å and the interactions are represented as green, dashed lines. Hydrophic interactions between **21** and LasB are shown as red spoked arcs. The Zn^{2+} cation is represented as a grey sphere.

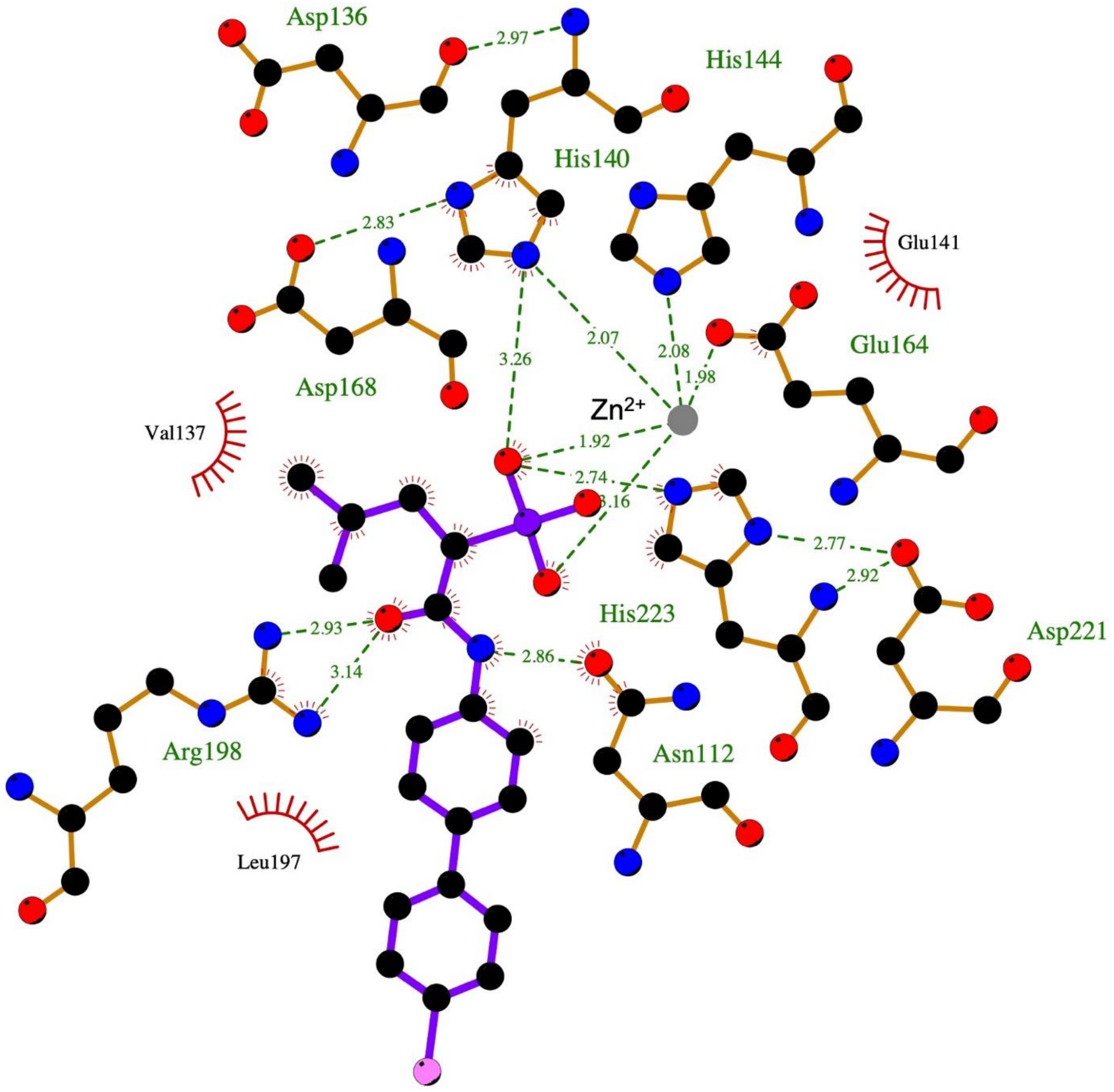

Figure S3. LigPlot+ diagram for the interactions between LasB mutant Met128Val and **21**. Ligand bonds are represented in purple and protein bonds are colored in dark brown. The distances for hydrogen bonds are shown in Å and the interactions are represented as green, dashed lines. Hydrophic interactions between **21** and LasB are shown as red spoked arcs. The Zn^{2+} cation is represented as a grey sphere.

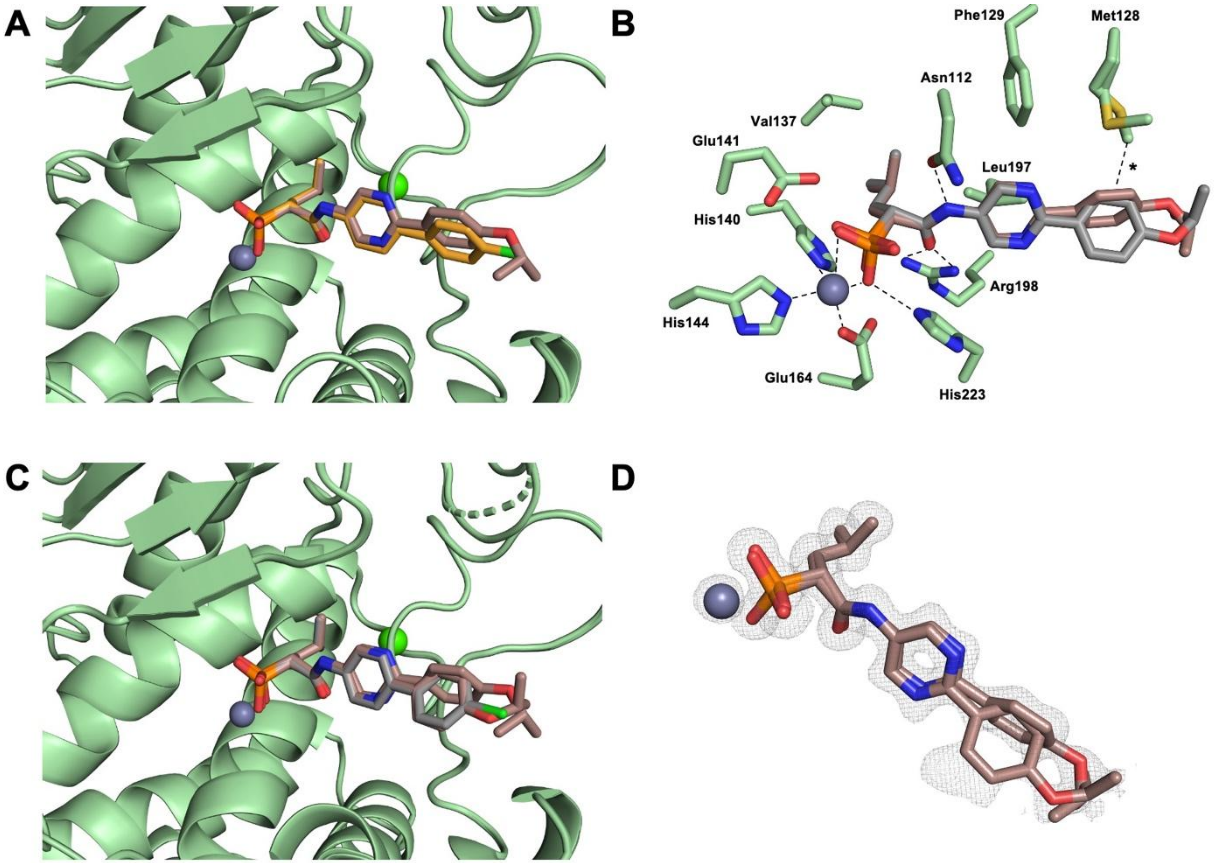

Figure S4. (**A**) Cartoon representation and superposition of LasB (green) in complex with **21** (orange, PDB code 9FRY) and **35** (pale red, PDB code 9FS0). The core of the compounds aligns very well and the Western part slightly differs in the ring conformation, but show CH-π interactions with the side chain of LasB Met128, which is highlighted by an asterisk. (**B**) Interactions between LasB and **35**. The conformation of the compound stabilized by the CH-π interaction with LasB Met128 is colored pale red and the alternative conformation is shown in grey. Both conformations of LasB Met128 are shown, indicating the conformational flexibility of the amino acid side chain. (**C**) Cartoon representation of both conformations of **35** bound to LasB. (**D**) Polder maps (grey isomesh) of **35** (pale red) contoured at a level of 3 σ.

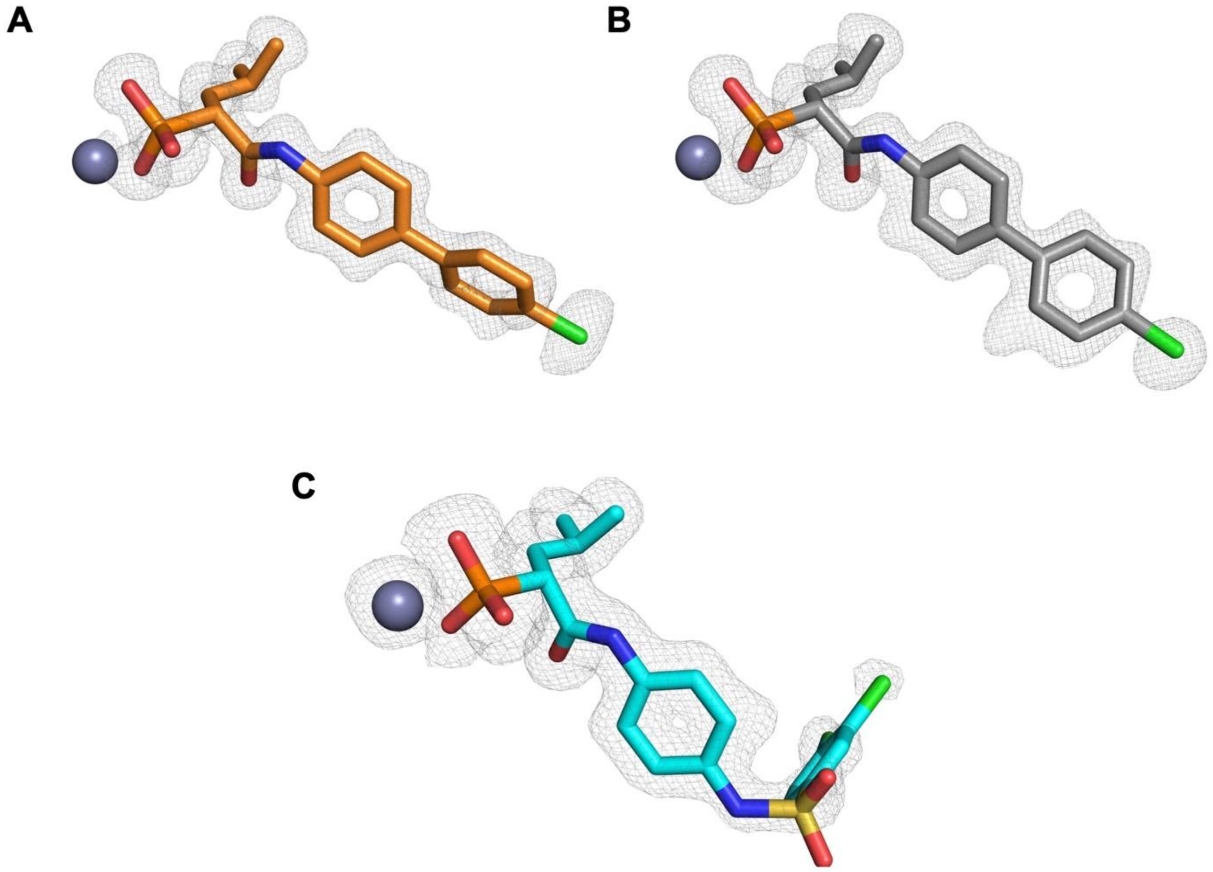

Figure S5. Polder map (grey isomesh) of the compounds (**A**) **21** (orange) bound to wild-type LasB, (**B**) **21** (grey) in complex with M128Val LasB mutant and (**C**) **141** (cyan) contoured at a level of 3 σ.

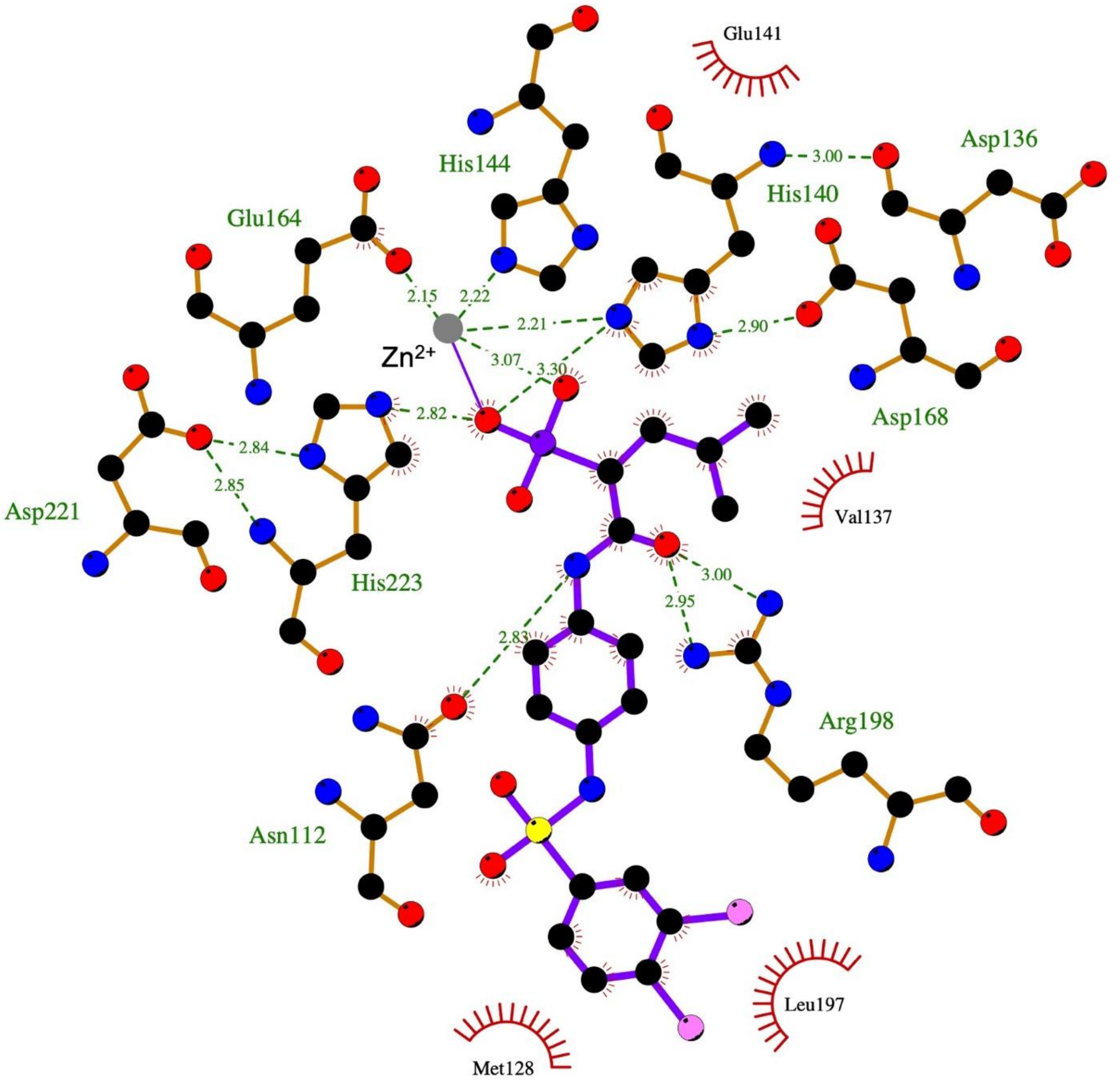

Figure S6. LigPlot+ diagram for the interactions between LasB and **141**. Ligand bonds are represented in purple and protein bonds are colored in dark brown. The distances for hydrogen bonds are shown in Å and the interactions are represented as green, dashed lines. Hydrophic interactions between **141** and LasB are shown as red spoked arcs. The Zn^{2+} cation is represented as a grey sphere.

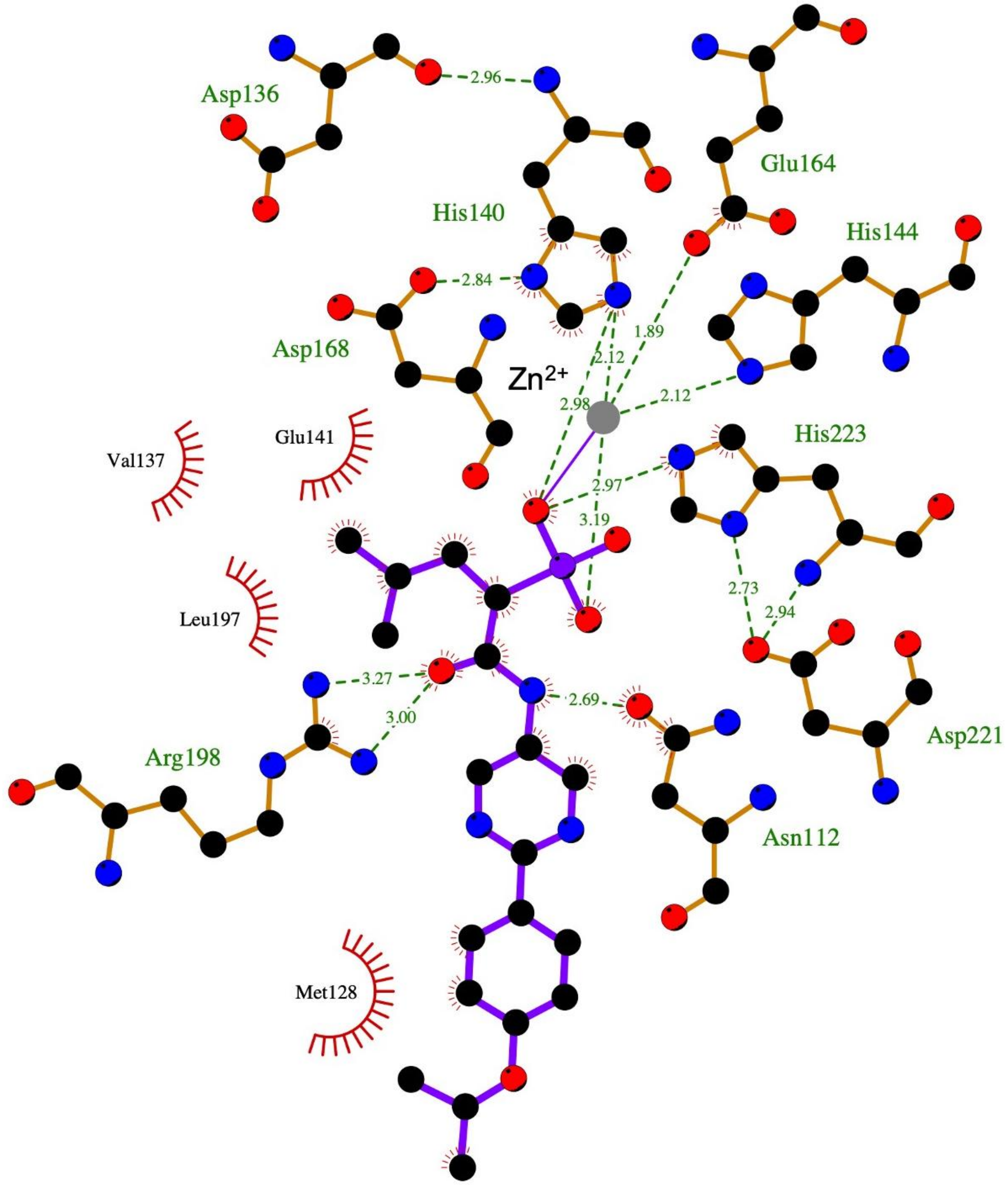

Figure S7. LigPlot+ diagram for the interactions between LasB and **35**. Ligand bonds are represented in purple and protein bonds are colored in dark brown. The distances for hydrogen bonds are shown in Å and the interactions are represented as green, dashed lines. Hydrophic interactions between **35** and LasB are shown as red spoked arcs. The Zn^{2+} cation is represented as a grey sphere.

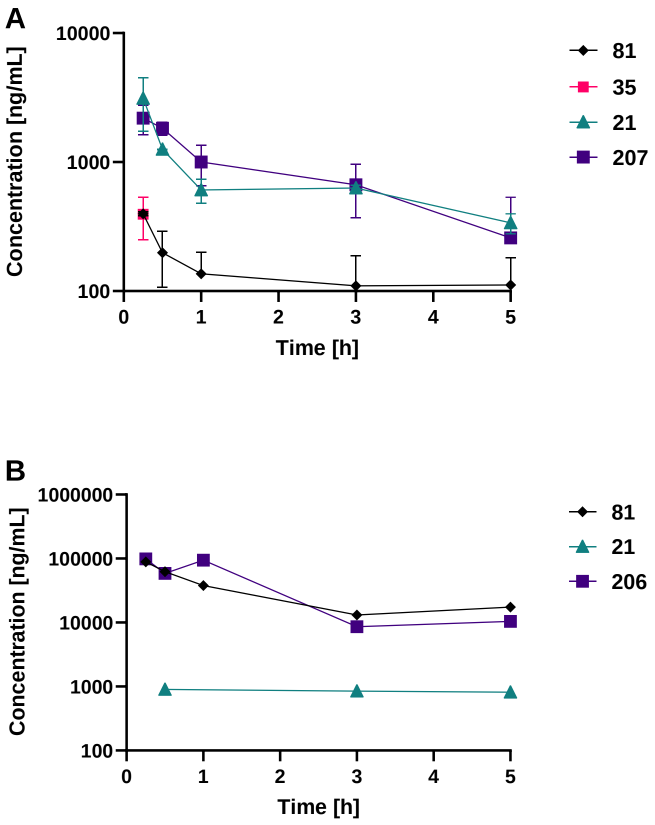

Figure S8. Concentrations of LasB inhibitors in plasma (**A**) and urine (**B**) after IV administration at 2 mg/kg (cassette dosing).

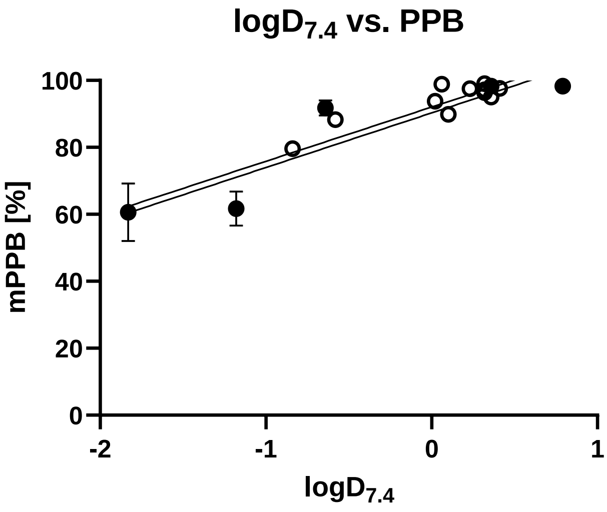

Figure S9. Correlation of chromatographic $logD_{7.4}$ with murine plasma protein binding. The initial set of 5 compounds (●, R^2 = 0.8274) is shown vs. the full dataset excluding **82** with PPB of 33% (○, R^2 = 0.8385).

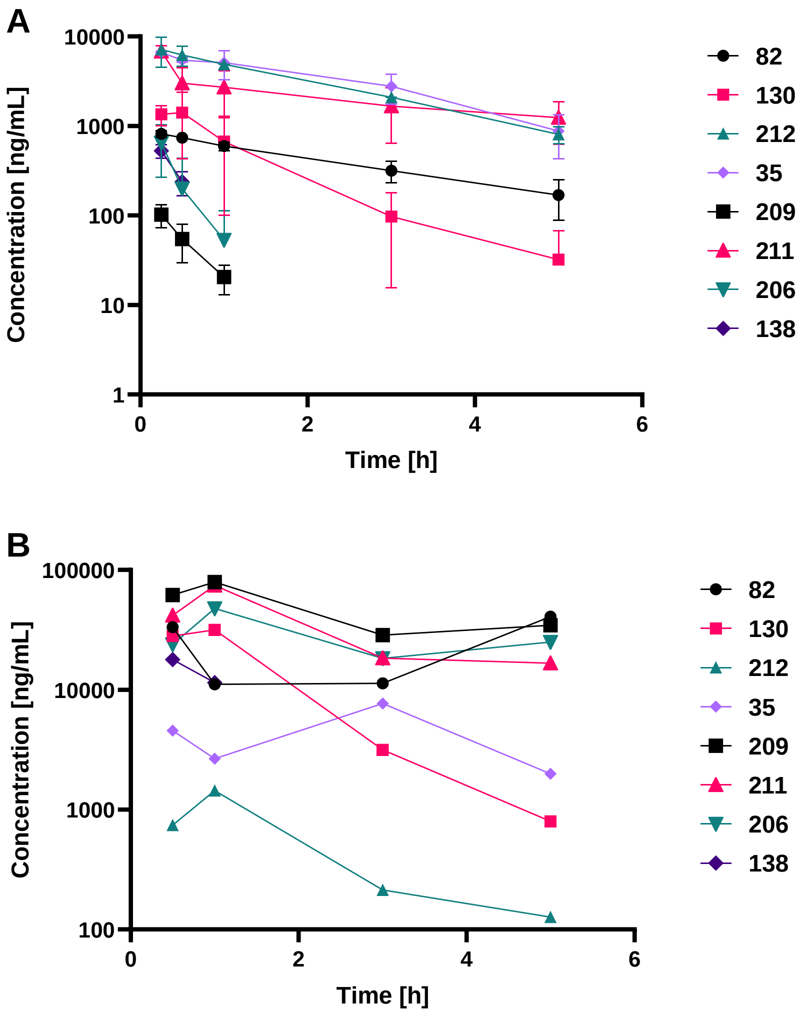

Figure S10. Concentrations of LasB inhibitors in plasma (**A**) and urine (**B**) after IV administration at 2 mg/kg (cassette dosing).

Experimental Section

Chemistry. All reagents were used from commercial suppliers without further purification. Procedures were not optimized regarding yield. NMR spectra were recorded on a Bruker AV 500 (500 MHz) spectrometer. Chemical shifts are given in parts per million (ppm) and referenced against the residual proton, ^{1}H, or carbon, ^{13}C, resonances of the >99% deuterated solvents as internal reference. Coupling constants (J) are given in Hertz. Data are reported as follows: chemical shift, multiplicity, coupling constants, and integration. Liquid chromatography-mass spectrometry was performed on a LC-MS system, consisting of a Dionex UltiMate 3000 pump, autosampler, column compartment and detector (Thermo Fisher Scientific, Dreieich, Germany) and ESI quadrupole MS (MSQ Plus or ISQ EC, Thermo Fisher Scientific, Dreieich, Germany). High resolution mass was determined by LC-MS/MS using Thermo Scientific Q Exactive Focus Orbitrap LC-MS/MS system. Purity of the final compounds was determined by LC-MS using the area percentage method on the UV trace recorded at a wavelength of 254 nm and found to be >95%.

General procedure A-1: Amide coupling using EDC·HCl followed by Boc-deprotection

Step 1: The acid (1.2–2.0 eq) was dissolved in DCM. EDC·HCl (1.2–2.0 eq) was added, followed by the corresponding aniline (1.0 eq). The resultant mixture was stirred at room temperature, until the starting aniline was consumed. The obtained solution was washed with 1 M HCl and brine. The organic layer was dried over anhydrous sodium sulfate, filtered and concentrated under reduced pressure to afford the crude product. The obtained crude product was either used in the next step without further purification or purified using column chromatography.

Step 2: Boc-protected aniline (1.0 eq) obtained in **Step 1** was dissolved in DCM. TFA (7.0 eq) were added and the mixture was stirred at r.t. overnight. The mixture was evaporated to dryness. Fresh DCM was added, washed with 2 M NaOH and brine. Dried over anhydrous sodium sulfate, filtered and concentrated under reduced pressure to afford the crude product which was used in the next step without purification.

General procedure A-2: Amide coupling using EDC·HCl and HOBt

2-(Diethoxyphosphoryl)-4-methylpentanoic acid (1.5 eq) was dissolved in DCM. EDC·HCl (2.0 eq), HOBt (2.0 eq) and DIPEA (2.4 eq) were added, followed by the corresponding aniline (1.0 eq). The resultant mixture was stirred at r.t., until the starting aniline was consumed. The obtained solution was washed with 1 M HCl and brine. The organic layer was dried over anhydrous sodium sulfate, filtered and concentrated under reduced pressure to afford the crude product. The obtained crude product was either used in the next step without further purification or purified using column chromatography.

General procedure A-3: Amide coupling using TBTU

2-(Diethoxyphosphoryl)-4-methylpentanoic acid (1.2 eq) was dissolved in DMF or DCM. TBTU (1.5 eq) and NMM (2.5 eq) were added, followed by the corresponding aniline (1.0 eq). The resultant mixture

was stirred at r.t., until the starting aniline was consumed. The obtained solution was washed with 1 M NaOH, 1 M HCl and brine. The organic layer was dried over anhydrous sodium sulfate, filtered and concentrated under reduced pressure to afford the crude product. The obtained crude product was either used in the next step without further purification or purified using column chromatography.

General procedure B: Synthesis of sulfonamides followed by Boc-deprotection

Step 1: *tert*-butyl (4-aminophenyl)carbamate (1.0 eq) was dissolved in dry DCM and cooled down to 0 °C. Et_3N (1.2 eq) was added, followed by the corresponding sulfonyl chloride (1.1 eq). Ice-bath was removed and the reaction mixture stirred overnight at r.t. Solvents were evaporated and the obtained crude product was purified using column chromatography.

Step 2: Boc-protected aniline (1.0 eq) obtained in **Step 1** was dissolved in DCM. TFA (7.0 eq) was added and the mixture was stirred at r.t. overnight. The mixture was evaporated to dryness. Fresh DCM was added, washed with 2 M NaOH and brine. Dried over anhydrous sodium sulfate, filtered and concentrated under reduced pressure to afford the crude product which was used in the next step without purification.

Alternatively, Boc-protected aniline (1.0 eq) obtained in **Step 1** was dissolved in DCM/MeOH (1/1). 4 M HCl in dioxane (10.0 eq) was added and the mixture was stirred at r.t. overnight. The mixture was evaporated to dryness and used in the next step without purification.

General procedure C: Synthesis of phosphonic acid derivatives

Step 1: *N*-Aryl-2-halo-2-alkylacetamide derivative (1.0 eq) was suspended in triethyl phosphite (10-25 eq) and heated to 150 °C in a sealed tube for a total of 18 h (or otherwise specified). Most of unreacted triethyl phosphite was evaporated in vacuo and the resultant oil was purified by column chromatography.

Step 2: To a solution of diethyl phosphonate (1.0 eq) in dry DCM, bromotrimethylsilane (5.0–7.0 eq) was added dropwise over a period of 15 min. The reaction mixture was stirred at r.t. overnight (or otherwise specified). If no full conversion was achieved, the excess of bromotrimethylsilane (5.0 eq) was added next day. Then MeOH was added and stirred for 30 min at room temperature to cleave the previously formed TMS ester. Solvents were concentrated in vacuo and the resultant oil was purified by preparative HPLC.

General procedure D: Synthesis of Suzuki coupling derivatives

To a mixture of bromo aryl (1 eq), corresponding boronic acid (1.5 eq) and potasium carbonate 2M (1 mL) in 1,4-dioxane:water mixture (4/1) (2 mL) was added Pd(dppf)Cl_2 (0.05 eq) the mixture was heated at 150°C for 20 min under microwave irradiation. The reaction mixture was concentrated *in vacuo.* The reaction mixtures were diluted with water (5 mL) and the aqueous layer was extracted with DCM (3 X 15 mL). The organic layer was dried over anhydrous sodium sulfate, filtered and evaporated to dryness under reduced pressure. The product was purified by column chromatography.

General procedure E: Synthesis of nitro derivatives

To a mixture of 1-fluoro-4-nitrobenzene (1.0 eq) in dry NMP or DMF (10 mL) were added corresponding aniline, phenol, or thiophenol (1.2 eq) and potassium carbonate (1.5 eq). The resulting

suspension was stirred at 150 °C for 2–18 h. The reaction mixture was cooled to RT, poured over ice, and filtered. The product was washed with water and dried to give the titled compound. The product was used in the next step without further purification.

General procedure F: Reduction to afford amino derivatives

A mixture of corresponding nitro derivative (1.0 eq), Fe (5.0 eq) and ammonium chloride (0.5 eq) was dissolved in an ethanol/water (2/1) mixture. The mixture was heated at 100 °C for 2 h. Excess ethanol was evaporated under reduced pressure and to the remaining residue, water (10 mL) was added and then extraction with ethyl acetate. The organic solvent was then dried over $MgSO_4$ and evaporated under reduced pressure. The product was purified by column chromatography to afford the desired compound.

2-Bromo-4-methylpentanoic acid (26)

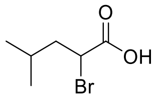

Racemic leucine (10.5 g 80.0 mmol) was dissolved in 48% HBr (80 mL) and 72 mL dist. water. The mixture was cooled to 0 °C and a solution of $NaNO_2$ (8.82 g, 128.0 mmol, 1.6 eq) in 20 mL dist. water was added dropwise over 2 h. The mixture was warmed up to rt and was stirred overnight. After that, the mixture was transferred into a separatory funnel and extracted with acetone (4 x 100 mL). The combined organic layers were washed with dist. water (400 mL) and saturated aqueous NaCl solution (400 mL), dried over $MgSO_4$, filtered and concentrated under reduced pressure. The compound **26** (15.06 g, 80.0 mmol, quant.) was obtained as a pale yellow liquid and was used in the next step without further purification. ^1H NMR (500 MHz, $CDCl_3$) δ 4.30 (t, *J*=7.7 Hz, 1H), 1.93 (t, *J*=7.3 Hz, 2H), 1.81 (dt, *J*=13.4, 6.7 Hz, 1H), 0.98 (d, *J*=6.6 Hz, 3H), 0.93 (d, *J*=6.6 Hz, 3H). ^{13}C NMR (126 MHz, $CDCl_3$) δ 176.16, 44.05, 43.34, 26.45, 22.46, 21.69.

Ethyl 2-bromo-4-methylpentanoate (27)

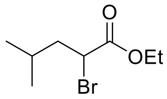

To α-bromo acid **26** (15.06 g, 80.0 mmol) a solution of concentrated sulphuric acid (30 μL/mmol) in ethanol (2 mL/mmol) was added and the mixture was refluxed for 2 h. After that, the solution was cooled to rt and concentrated under reduced pressure. Et_2O (150 mL) was added and the organic layer was washed with aqueous saturated $NaHCO_3$ solution (150 mL), followed by saturated aqueous NaCl solution (150 mL). The organic layer was dried over $MgSO_4$, filtered and concentrated under reduced pressure. The compound **27** (14.45 g, 64.7 mmol, 81% yield) as a pale yellow liquid and was used in the next step without further purification. ^1H NMR (500 MHz, $CDCl_3$) δ 4.29 – 4.20 (m, 2H), 1.90 (td, *J*=7.2, 2.4 Hz, 2H), 1.76 (dp, *J*=13.4, 6.7 Hz, 1H), 1.30 (t, *J*=7.1 Hz, 3H), 0.96 (d, *J*=6.6 Hz, 3H), 0.91 (d, J=6.6 Hz, 3H). ^{13}C NMR (126 MHz, $CDCl_3$) δ 170.3, 62.1, 44.9, 43.6, 26.5, 22.5, 21.7, 14.1.

Ethyl 2-(diethoxyphosphoryl)-4-methylpentanoate (28)

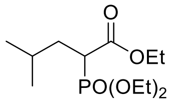

The α-bromo ester **27** (14.45 g, 64.7 mmol) and $P(OEt)_3$ (22.41 mL, 129.4 mmol) were mixed and heated to 150 °C for 48 h. After that, the mixture was cooled down to rt, and Et_2O (350 mL) was added. The mixture was transferred into a separatory funnel and washed with saturated aqueous NaCl solution (2 x 350 mL), dried over $MgSO_4$, filtered and concentrated under reduced pressure. The product was purified using flash chromatography (SiO_2, hexanes/EtOAc

1/1), and compound **28** (7.93 g, 28.3 mmol, 44%) was obtained as pale yellow oil. ^{1}H NMR (500 MHz, CDCl3) δ 4.24 – 4.06 (m, 6H), 3.02 (ddd, *J*=23.2, 11.5, 2.9 Hz, 1H), 2.06 – 1.94 (m, 1H), 1.65 – 1.53 (m, 2H), 1.32 (td, *J*=7.1, 2.4 Hz, 6H), 1.27 (t, *J*=7.2 Hz, 3H), 0.90 (dd, *J*=12.5, 6.2 Hz, 6H). ^{13}C NMR (126 MHz, $CDCl_3$) δ 169.6 (d, *J* = 5.0 Hz), 62.9 (d, *J* = 6.5 Hz), 62.8 (d, *J* = 6.9 Hz), 61.4, 44.1 (d, *J* = 131.0 Hz), 35.7 (d, *J* = 5.1 Hz), 27.1 (d, *J* = 14.7 Hz), 23.1, 21.4, 16.5 (d, *J* = 3.6 Hz), 16.5 (d, *J* = 3.7 Hz), 14.3. ^{31}P NMR (202 MHz, CDCl3) δ 23.39. HRMS (ESI^+) calculated for $C_{12}H_{24}O_5P^+$ $[M+H]^+$ 281.1503, found 281.1503.

2-(Diethoxyphosphoryl)-4-methylpentanoic acid (29)

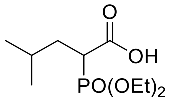

The compound **28** (7.93 g, 28.3 mmol) was dissolved in EtOH (270 mL), and NaOH (2.15 g, 53.9 mmol) in dist. H_2O (100 mL) was added. The mixture was stirred at rt overnight. The progress was monitored using LC-MS. After completion, the mixture was transferred into a separatory funnel, dist. water (300 mL) and Et_2O (400 mL) were added, and the layers were separated. The aqueous layer was acidified to pH = 1 using HCl (6 M), and extracted with EtOAc (3 x 300 mL). The combined organic were washed with saturated aqueous NaCl solution (2 x 500 mL), dried over $MgSO_4$, filtered and concentrated under reduced pressure. The compound **29** (6.63 g, 26.3 mmol, 98%) was obtained as a pale-yellow oil, which was used without further purification. ^{1}H NMR (500 MHz, $CDCl_3$) δ 4.27 – 4.09 (m, 4H), 3.06 (ddd, *J*=23.3, 11.4, 3.3 Hz, 1H), 1.99 (dddd, *J*=13.5, 11.4, 8.5, 4.8 Hz, 1H), 1.65 (dddd, *J*=13.0, 11.3, 9.1, 5.4 Hz, 1H), 1.53 (dddd, *J*=13.9, 10.8, 9.3, 3.4 Hz, 1H), 1.37 – 1.30 (m, 6H), 0.91 (dd, *J*=15.7, 6.6 Hz, 6H). ^{13}C NMR (126 MHz, $CDCl_3$) δ 172.0 (d, *J* = 4.2 Hz), 63.8 (d, *J* = 6.5 Hz), 63.1 (d, *J* = 6.8 Hz), 44.0 (d, *J* = 130.1 Hz), 35.7 (d, *J* = 5.6 Hz), 26.9 (d, *J* = 14.5 Hz), 23.1, 21.3, 16.5 (d, *J* = 2.7 Hz), 16.4 (d, *J* = 2.6 Hz). ^{31}P NMR (202 MHz, $CDCl_3$) δ 24.26. HRMS (ESI^+) calculated for $C_{10}H_{22}O_5P^+$ $[M+H]^+$ 253.1199, found 253.1191.

4'-Chloro-biphenyl-2-ylamine (1).

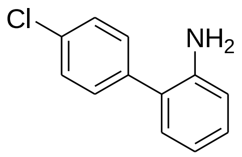

Compound **1** was synthesized according to general procedure D, using 2-bromobenzenamine (172 mg, 1 mmol), 4-chlorophenylboronic acid (234 mg, 1.5 mmol), Pd(dpff)Cl_2 (36.5 mg, 0.05 mmol) and Na_2CO_3 (2M, 1 mL) in DCM (20 mL). The crude product was purified using column chromatography (DCM). The product was obtained as brown oil (162 mg, 80%). MS (ESI^+) m/z 204.03 $[M+H]^+$.

2-Chloro-*N*-[4'-chloro-(1,1'-biphenyl)-2-yl]-4-methylpentanamide (7).

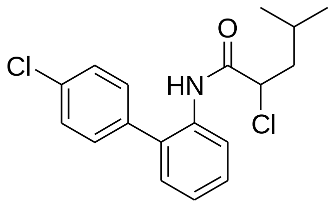

Compound 7 was synthesized according to general procedure A-1 (step 1), using **1** (100 mg, 0.50 mmol), 2-chloro-4-methylpentanoic acid (150 mg, 1 mmol) and EDC·HCl (191 mg, 1 mmol) in DCM (20 mL). The reaction was stirred at room temperature for 24 h. The crude product was purified by automated column chromatography (DCM). The product was obtained as yellow solid (122 mg, 74%). ^{1}H NMR (500 MHz, $CDCl_3$) δ ppm: 8.11 (d, *J* = 8.2 Hz, 1H), 7.92 (s, 1H), 7.38 (d, *J* = 8.4 Hz, 2H), 7.34 – 7.29 (m, 1H), 7.25 (d, *J* = 8.4 Hz, 2H), 7.19 – 7.09 (m,

2H), 4.28 – 4.19 (m, 1H), 1.91 – 1.81 (m, 1H), 1.80 – 1.66 (m, 2H), 0.87 (d, *J* = 6.3 Hz, 3H), 0.82 (d, *J* = 6.4 Hz, 3H). ^{13}C NMR (126 MHz, $CDCl_3$) δ ppm: 167.1, 136.1, 134.3, 134.1, 132.0, 130.9, 130.1, 129.3, 128.8, 125.2, 121.8, 50.6, 44.6, 26.4, 22.6, 21.1. MS (ESI^+) m/z 336.03 $[M+H]^+$.

[1-(4'-Chloro-biphenyl-2-ylcarbamoyl)-3-methyl-butyl]-diethyl phosphonate (13).

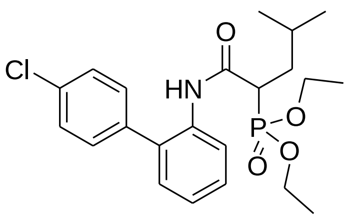

Compound **13** was synthesized according to general procedure C (step 1), using **7** (110 mg, 0.29 mmol) and triethyl phosphite (0.53 mL, 2.9 mmol). The residue was purified by automated column chromatography (petroleum ether/EtOAc=9/1 to EtOAc) to give the desired product as white solid (90 mg, 63%). MS (ESI^+) m/z 438.07 $[M+H]^+$.

4'-Chloro-biphenyl-3-ylamine (2).

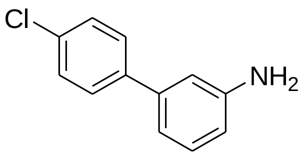

Compound **2** was synthesized according to general procedure D, using 3-bromobenzenamine (172 mg, 1 mmol), 4-chlorophenylboronic acid (234 mg, 1.5 mmol), Pd(dpff)Cl_2 (36.5 mg, 0.05 mmol) and Na_2CO_3 (2 M, 1 mL) in DCM (20 mL). The crude product was purified using column chromatography (DCM). The product was obtained as yellow solid (186 mg, 92%). ^{1}H NMR (500 MHz, DMSO) δ ppm: 7.60 – 7.55 (m, 2H), 7.50 – 7.46 (m, 2H), 7.11 (t, *J* = 7.8 Hz, 1H), 6.83 (t, *J* = 1.9 Hz, 1H), 6.79 – 6.71 (m, 1H), 6.61 – 6.55 (m, 1H), 5.19 (s, 2H). ^{13}C NMR (126 MHz, DMSO) δ ppm: 149.7, 140.4, 140.0, 132.3, 130.0, 129.2, 128.6, 114.7, 113.9, 112.4. MS (ESI^+) m/z 204.09 $[M+H]^+$.

2-Bromo-4-methyl-pentanoic acid (4'-chloro-biphenyl-3-yl)-amide (8).

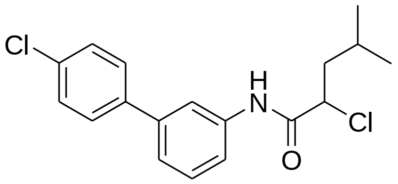

Compound **8** was synthesized according to general procedure A (step 1), using **2** (100 mg, 0.50 mmol), 2-chloro-4-methylpentanoic acid (150 mg, 1 mmol) and EDC·HCl (191 mg, 1 mmol) in DCM (20 mL). The reaction was stirred at room temperature for 24 h. The crude product was purified by automated column chromatography (DCM). The product was obtained as white solid (130 mg, 79%). ^{1}H NMR (500 MHz, $CDCl_3$) δ ppm: 8.03 (s, 1H), 7.73 (d, *J* = 7.6 Hz, 1H), 7.46 – 7.40 (m, 3H), 7.37 – 7.31 (m, 3H), 7.27 (d, *J* = 7.6 Hz, 1H), 4.45 – 4.35 (m, 1H), 2.05 – 1.89 (m, 2H), 1.89 – 1.79 (m, 1H), 0.94 (d, *J* = 6.5 Hz, 3H), 0.89 (d, *J* = 6.4 Hz, 3H). ^{13}C NMR (126 MHz, $CDCl_3$) δ ppm: 167.3, 141.1, 138.9, 137.8, 133.8, 129.6, 129.0, 128.5, 123.6, 119.0, 118.6, 50.6, 44.6, 26.5, 22.7, 21.1. MS (ESI^+) m/z 336.17 $[M+H]^+$.

[1-(4'-Chloro-biphenyl-3-ylcarbamoyl)-3-methyl-butyl]-diethyl phosphonate (14).

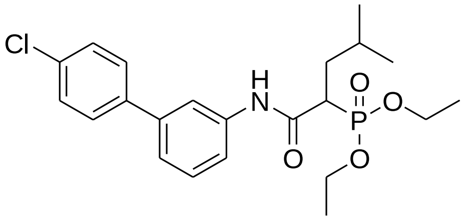

Compound **14** was synthesized according to general procedure C (step 1), using **8** (110 mg, 0.29 mmol) and triethyl phosphite (0.53 mL, 2.9 mmol). The residue was purified by automated column chromatography (petroleum ether/EtOAc=9/1 to EtOAc) to give the desired product as white solid (93 mg, 65%). MS (ESI^+) m/z 438.41 $[M+H]^+$.

4'-Chloro-biphenyl-4-ylamine (3).

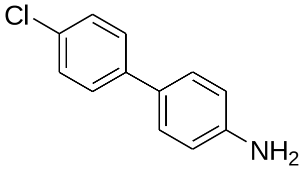

Compound **3** was synthesized according to general procedure D, using 4-bromobenzenamine (172 mg, 1 mmol), 4-chlorophenylboronic acid (234 mg, 1.5 mmol), Pd(dpff)Cl_2 (36.5 mg, 0.05 mmol) and Na_2CO_3 (2 M, 1 mL) in DCM (20 mL). The crude product was purified using column chromatography (DCM). The product was obtained as yellow solid (150 mg, 74%). ^{1}H NMR (500 MHz, DMSO) δ ppm: 7.59 (d, *J* = 8.6 Hz, 2H), 7.43 (d, *J* = 8.6 Hz, 2H), 7.39 (d, *J* = 8.5 Hz, 2H), 6.68 (d, *J* = 8.5 Hz, 2H), 5.33 (s, 2H). ^{13}C NMR (126 MHz, DMSO) δ ppm: 140.0, 130.7, 129.1, 127.6, 127.4, 126.3, 117.4, 114.7. MS (ESI$^+$) m/z 204.01 [M+H]$^+$.

2-Chloro-*N*-[4'-chloro-(1,1'-biphenyl)-4-yl]-4-methylpentanamide (9).

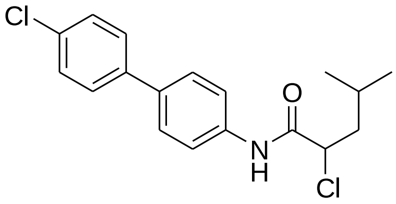

Compound **9** was synthesized according to general procedure A-1 (step 1), using **3** (100 mg, 0.50 mmol), 2-chloro-4-methylpentanoic acid (150 mg, 1 mmol) and EDC·HCl (191 mg, 1 mmol) in DCM (20 mL). The reaction was stirred at room temperature for 24 h. The crude product was purified by automated column chromatography (DCM). The product was obtained as pale yellow solid (110 mg, 67%). ^{1}H NMR (500 MHz, $CDCl_3$) δ ppm: 8.26 (s, 1H), 7.55 (d, *J* = 8.5 Hz, 2H), 7.46 (d, *J* = 8.4 Hz, 2H), 7.41 (d, *J* = 8.4 Hz, 2H), 7.32 (d, *J* = 8.3 Hz, 2H), 4.42 (dd, *J* = 10.1, 4.1 Hz, 1H), 2.03 – 1.95 (m, 1H), 1.94 – 1.79 (m, 2H), 0.93 (d, *J* = 6.5 Hz, 3H), 0.91 (d, *J* = 6.3 Hz, 3H). ^{13}C NMR (126 MHz, $CDCl_3$) δ ppm: 167.5, 138.8, 136.6, 136.6, 133.4, 129.0, 128.1, 127.6, 120.4, 60.2, 44.4, 25.3, 22.9, 20.9. MS (ESI$^+$) m/z 336.03 [M+H]$^+$.

[1-(4'-Chloro-biphenyl-4-ylcarbamoyl)-3-methyl-butyl]-diethyl phosphonate (15).

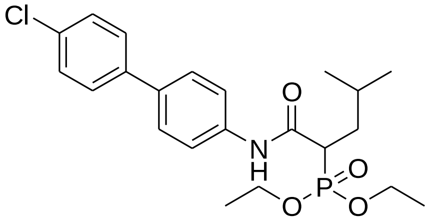

Compound **15** was synthesized according to general procedure C (step 1), using **9** (98 mg, 0.29 mmol) and triethyl phosphite (0.53 mL, 2.9 mmol). The residue was purified by automated column chromatography (petroleum ether/EtOAc=9/1 to EtOAc) to give the desired product as white solid (115 mg, 80%). ^{1}H NMR (500 MHz, $CDCl_3$) δ ppm: 9.13 (s, 1H), 7.49 (d, *J* = 8.3 Hz, 2H), 7.31 – 7.28 (m, 3H), 7.28 – 7.19 (m, 3H), 4.25 – 3.98 (m, 4H), 3.10 (ddd, *J* = 22.6, 11.1, 1.3 Hz, 1H), 2.06 (ddd, *J* = 18.7, 10.5, 4.4 Hz, 1H), 1.69 – 1.60 (m, 1H), 1.50 (ddd, *J* = 23.6, 10.1, 3.0 Hz, 1H), 1.35 – 1.25 (m, 6H), 0.88 (t, *J* = 6.7 Hz, 6H). ^{13}C NMR (126 MHz, $CDCl_3$) δ ppm: 166.0 (d, *J* = 2.6 Hz), 139.00 (s), 137.8 (s), 135.4 (s), 133.0 (s), 128.8 (s), 127.9 (s), 127.1 (s), 119.9 (s), 63.5 (d, *J* = 6.6 Hz), 62.5 (d, *J* = 7.0 Hz), 45.3 (d, *J* = 129.2 Hz), 35.9 (d, *J* = 5.2 Hz), 26.6 (d, *J* = 14.1 Hz), 23.3 (s), 21.3 (s), 16.5 (d, *J* = 6.2 Hz), 16.4 (d, *J* = 6.5 Hz). ^{31}P NMR (202 MHz, $CDCl_3$) δ ppm: 26.22. MS (ESI$^+$) m/z 438.00 [M+H]$^+$.

5-(4-Chlorophenyl)pyridin-2-amine (4).

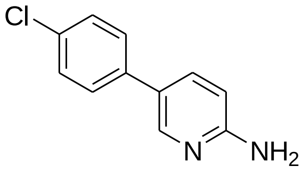

Compound **4** was synthesized according to general procedure D, using 5-bromopyridin-2-amine (173 mg, 1 mmol), 4-chlorophenylboronic acid (234 mg, 1.5 mmol), Pd(dpff)Cl_2 (36.5 mg, 0.05 mmol) and Na_2CO_3 (2 M, 1 mL) in DCM (20 mL). The crude product was purified using column chromatography (DCM to DCM/MeOH 1%). The product was obtained as pale yellow solid (170 mg, 83%). ^{1}H NMR (500 MHz, Acetone) δ ppm: 7.57 (dd, *J* = 8.6, 2.4 Hz, 1H), 7.43 (d, *J* = 8.6 Hz, 2H), 7.28 (d, *J* = 8.6 Hz, 2H), 6.52 (d, *J* = 8.6 Hz, 1H), 5.59 (s, 2H). ^{13}C NMR (126 MHz, Acetone) δ ppm: 159.3, 145.7, 137.5, 135.7, 131.9, 128.9, 127.3, 124.1, 108.3. MS (ESI$^+$) m/z 205.11 [M+H]$^+$.

2-Chloro-*N*-[5-(4-chlorophenyl)pyridin-2-yl]-4-methylpentanamide (10).

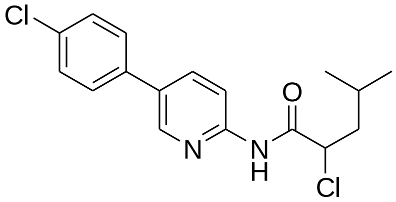

Compound **10** was synthesized according to general procedure A-1 (step 1), using **4** (102 mg, 0.50 mmol), 2-chloro-4-methylpentanoic acid (150 mg, 1 mmol) and EDC·HCl (191 mg, 1 mmol) in DCM (20 mL). The reaction was stirred at room temperature for 24 h. The crude product was purified by automated column chromatography (DCM). The product was obtained as yellow solid (106 mg, 63%). ^{1}H NMR (500 MHz, $CDCl_3$) δ ppm: 8.89 (s, 1H), 8.43 (dd, *J* = 2.4, 0.6 Hz, 1H), 8.20 (d, *J* = 8.6 Hz, 1H), 7.82 (dd, *J* = 8.6, 2.5 Hz, 1H), 7.43 – 7.38 (m, 2H), 7.38 – 7.32 (m, 2H), 4.40 (dd, *J* = 9.8, 4.5 Hz, 1H), 2.01 – 1.78 (m, 3H), 0.93 (d, *J* = 6.3 Hz, 3H), 0.89 (d, *J* = 6.4 Hz, 3H). ^{13}C NMR (126 MHz, $CDCl_3$) δ ppm: 168.0, 150.1, 146.1, 136.7, 135.7, 134.2, 132.4, 129.3, 128.1, 113.8, 59.5, 44.3, 25.3, 22.8, 21.0. MS (ESI$^+$) m/z 337.01 [M+H]$^+$.

Diethyl 1-[5-(4-chlorophenyl)pyridin-2-ylcarbamoyl]-3-methylbutylphosphonate (16).

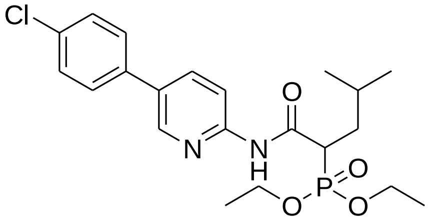

Compound **16** was synthesized according to general procedure C (step 1), using **10** (196 mg, 0.58 mmol) and triethyl phosphite (0.106 mL, 5.8 mmol). The residue was purified by automated column chromatography (DCM to DCM/MeOH 1%) to give the desired product as white solid (112 mg, 44%). MS (ESI$^+$) m/z 439.17 [M+H]$^+$.

6-(4-Chlorophenyl)pyridin-3-amine (5).

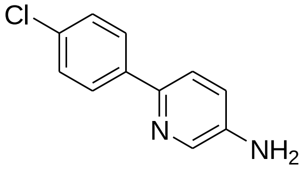

Compound **5** was synthesized according to general procedure D, using 5-bromopyridin-3-amine (173 mg, 1 mmol), 4-chlorophenylboronic acid (234 mg, 1.5 mmol), Pd(dpff)Cl_2 (36.5 mg, 0.05 mmol) and Na_2CO_3 (2M, 1 mL) in DCM (20 mL). The crude product was purified using column chromatography (DCM to DCM/MeOH 1%). The product was obtained as yellow solid (165 mg, 80%). ^{1}H NMR (500 MHz, Acetone) δ ppm: 8.21 (d, *J* = 2.8 Hz, 1H), 8.02 – 7.98 (m, 2H), 7.63 (d, *J* = 8.6 Hz, 1H), 7.43 – 7.39 (m, 2H), 7.14 (dd, *J* = 8.5, 2.8 Hz, 1H). ^{13}C NMR (126 MHz, Acetone) δ ppm: 141.2, 138.5, 136.5, 132.5, 128.5, 127.8, 127.0, 121.2, 120.3. MS (ESI$^+$) m/z 205.15 [M+H]$^+$.

2-Chloro-*N*-[6-(4-chlorophenyl)pyridin-3-yl]-4-methylpentanamide (11).

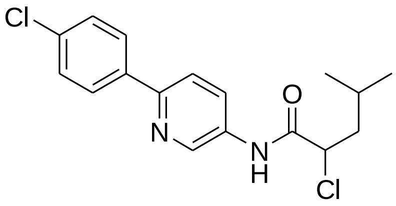

Compound **11** was synthesized according to general procedure A-1 (step 1), using **5** (102 mg, 0.50 mmol), 2-chloro-4-methylpentanoic acid (150 mg, 1 mmol) and EDC·HCl (191 mg, 1 mmol) in DCM (20 mL). The reaction was stirred at room temperature for 24 h. The crude product was purified by automated column chromatography (DCM). The product was obtained as brown solid (109 mg, 65%). ^{1}H NMR (500 MHz, $CDCl_3$) δ ppm: 8.62 (d, *J* = 2.3 Hz, 1H), 8.44 (s, 1H), 8.19 (dd, *J* = 8.7, 2.6 Hz, 1H), 7.82 – 7.73 (m, 2H), 7.61 (d, *J* = 8.7 Hz, 1H), 7.38 – 7.32 (m, 2H), 4.43 (dd, *J* = 10.0, 4.2 Hz, 1H), 1.97 (ddd, *J* = 13.1, 7.7, 3.7 Hz, 1H), 1.93 – 1.77 (m, 2H), 0.93 (d, *J* = 6.4 Hz, 3H), 0.90 (d, *J* = 6.4 Hz, 3H). ^{13}C NMR (126 MHz, $CDCl_3$) δ ppm: 168.1, 152.5, 141.0, 136.9, 135.1, 133.0, 129.0, 128.4, 128.0, 120.7, 59.8, 44.2, 25.3, 22.9, 20.8. MS (ESI^{+}) m/z 337.08 [M+H]$^{+}$.

Diethyl 1-[6-(4-chlorophenyl)pyridin-3-ylcarbamoyl]-3-methylbutylphosphonate (17).

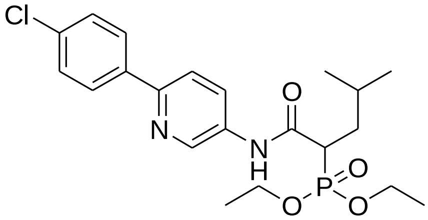

Compound **17** was synthesized according to general procedure C (step 1), using **11** (196 mg, 0.58 mmol) and triethyl phosphite (0.106 mL, 5.8 mmol). The residue was purified by automated column chromatography (DCM to DCM/MeOH 1%) to give the desired product as white solid (124 mg, 49%). ^{1}H NMR (500 MHz, DMSO) δ ppm: 10.54 (s, 1H), 8.79 (d, *J* = 2.5 Hz, 1H), 8.17 (dd, *J* = 8.7, 2.6 Hz, 1H), 8.11 – 8.03 (m, 2H), 7.98 (d, *J* = 8.7 Hz, 1H), 7.57 – 7.49 (m, 2H), 4.14 – 3.90 (m, 4H), 3.25 (ddd, *J* = 22.6, 11.2, 2.9 Hz, 1H), 2.06 – 1.92 (m, 1H), 1.58 – 1.40 (m, 2H), 1.23 (dt, *J* = 9.1, 7.1 Hz, 6H), 0.90 (d, *J* = 2.5 Hz, 3H), 0.89 (d, *J* = 2.5 Hz, 3H). ^{13}C NMR (126 MHz, DMSO) δ ppm: 167.6 (d, *J* = 4.8 Hz), 150.1, 140.7, 137.5, 135.4, 133.9, 129.2, 128.3, 127.4, 120.8, 62.5 (dd, *J* = 24.1, 6.5 Hz), 45.0 (d, *J* = 129.9 Hz), 35.8 (d, *J* = 4.8 Hz), 26.8 (d, *J* = 14.9 Hz), 23.4, 21.7, 16.8 (d, *J* = 5.6 Hz). ^{31}P NMR (202 MHz, DMSO) δ ppm: 24.31. MS (ESI^{+}) m/z 439.20 [M+H]$^{+}$.

2-(4-Chlorophenyl)pyrimidin-5-amine (6).

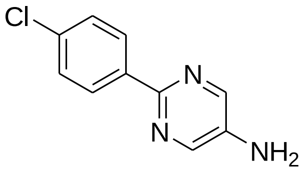

Compound **6** was synthesized according to general procedure D, using 2-bromo-pyrimidin-5-ylamine (174 mg, 1 mmol), 4-chlorophenylboronic acid (234 mg, 1.5 mmol), Pd(dpff)Cl_2 (36.5 mg, 0.05 mmol) and Na_2CO_3 (2 M, 1 mL) in DCM (20 mL). The crude product was purified using column chromatography (DCM to DCM/MeOH 2%). The product was obtained as yellow solid (247 mg, 81%). MS (ESI^{+}) m/z 306.03 [M+H]$^{+}$.

2-Chloro-*N*-[2-(4-chlorophenyl)pyrimidin-5-yl]-4-methylpentanamide (12).

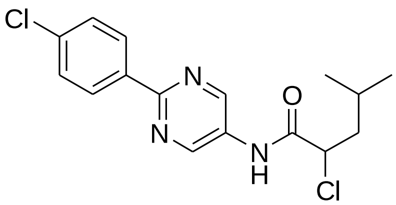

Compound **12** was synthesized according to general procedure A-1 (step 1), using **6** (103 mg, 0.50 mmol), 2-chloro-4-methylpentanoic acid (150 mg, 1 mmol) and EDC·HCl (191 mg, 1 mmol) in DCM (20 mL). The reaction was stirred at room temperature for 24 h. The

crude product was purified by automated column chromatography (DCM to DCM/MeOH 1%). The product was obtained as brown solid (102 mg, 88%). ^{1}H NMR (500 MHz, $CDCl_3$) δ ppm: 10.27 (s, 1H), 8.79 (s, 2H), 7.90 (d, *J* = 8.8 Hz, 2H), 6.69 (d, *J* = 8.8 Hz, 2H), 4.50 (hept, *J* = 6.0 Hz, 1H), 4.14 – 4.05 (m, 1H), 2.17 – 2.06 (m, 1H), 1.60 – 1.52 (m, 1H), 0.87 (d, *J* = 6.6 Hz, 3H), 0.85 (d, *J* = 6.5 Hz, 3H). MS (ESI^+) m/z 338.13 $[M+H]^+$.

Diethyl 1-[2-(4-chlorophenyl)pyrimidin-5-ylcarbamoyl]-3-methylbutylphosphonate (18).

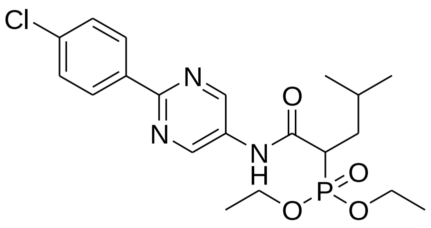

Compound **18** was synthesized according to general procedure C (step 1), using **12** (98 mg, 0.29 mmol) and triethyl phosphite (0.53 mL, 2.9 mmol). The residue was purified by automated column chromatography (DCM to DCM/MeOH 1%) to give the desired product as beige solid (91 mg, 71%). MS (ESI^+) m/z 440.17 $[M+H]^+$.

2-(4-Isopropoxyphenyl)pyrimidin-5-amine (31).

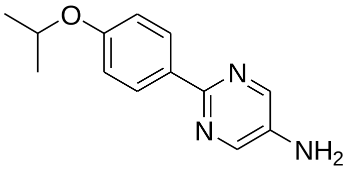

Compound **31** was synthesized according to general procedure D, using 2-bromo-pyrimidin-5-ylamine (174 mg, 1 mmol), 4-isopropoxyphenylboronic acid (270 mg, 1.5 mmol), Pd(dpff)Cl_2 (36.5 mg, 0.05 mmol) and Na_2CO_3 (2 M, 1 mL) in DCM (20 mL). The crude product was purified using column chromatography (DCM to DCM/MeOH 1%). The product was obtained as yellow solid (260 mg, 85%). MS (ESI^+) m/z 230.04 $[M+H]^+$.

Diethyl 1-[2-(4-isopropoxyphenyl)pyrimidin-5-ylcarbamoyl]-3-methylbutylphosphonate (33).

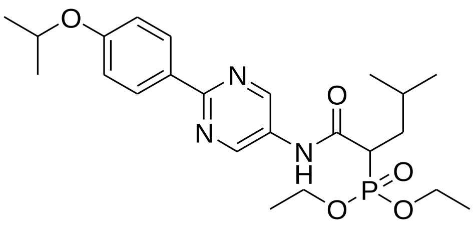

Compound **33** was synthesized according to general procedure A-2, using **31** (115 mg, 0.50 mmol), 2-(diethoxy-phosphoryl)-4-methyl-pentanoic acid **29** (252 mg, 1 mmol) and EDC·HCl (191 mg, 1 mmol), HOBt (135 mg, 1 mmol) and DIPEA (205 µL, 1.2 mmol) in DCM (20 mL). The reaction was stirred at room temperature for 24 h. The crude product was purified by automated column chromatography (DCM to DCM/MeOH 1%). The product was obtained as white solid (120 mg, 52%). ^{1}H NMR (500 MHz, $CDCl_3$) δ ppm: 10.27 (s, 1H), 8.79 (s, 2H), 7.90 (d, *J* = 8.8 Hz, 2H), 6.69 (d, *J* = 8.8 Hz, 2H), 4.50 (hept, *J* = 6.0 Hz, 1H), 4.32 – 4.17 (m, 2H), 4.14 – 4.05 (m, 2H), 3.34 (ddd, *J* = 22.8, 11.3, 2.8 Hz, 1H), 2.17 – 2.06 (m, 1H), 1.60 – 1.52 (m, 1H), 1.47 – 1.39 (m, 1H), 1.36 – 1.30 (m, 6H), 1.29 – 1.26 (m, 6H), 0.87 (d, *J* = 6.6 Hz, 3H), 0.85 (d, *J* = 6.5 Hz, 3H). ^{13}C NMR (126 MHz, $CDCl_3$) δ ppm: 166.8 (d, *J* = 3.5 Hz), 159.7, 159.5, 147.2, 131.2, 129.8, 129.3, 115.2, 69.6, 64.5 (d, *J* = 6.4 Hz), 62.1 (d, *J* = 7.2 Hz), 45.1 (d, *J* = 130.1 Hz), 35.8 (d, *J* = 5.8 Hz), 26.6 (d, *J* = 14.7 Hz), 23.2, 22.1, 22.0, 21.3, 16.5 (d, *J* = 6.1 Hz), 16.4 (d, *J* = 6.4 Hz). ^{31}P NMR (202 MHz, $CDCl_3$) δ ppm: 25.37. MS (ESI^+) m/z 464.22 $[M+H]^+$.

2-(3-Chloro-4-isopropoxyphenyl)pyrimidin-5-amine (32).

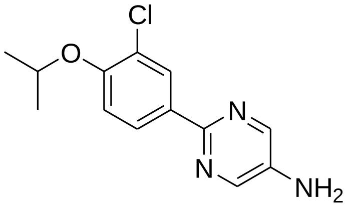

Compound **32** was synthesized according to general procedure D, using 2-bromo-pyrimidin-5-ylamine (174 mg, 1 mmol), 3-chloro-4-isopropoxyphenylboronic acid (321 mg, 1.5 mmol), tetrakis(triphenylphosphine)palladium(0) (58 mg, 0.05 mmol) and Na_2CO_3 (2 M, 1 mL) in DCM (20 mL). The crude product was purified using column chromatography (DCM to DCM/MeOH 1%). The product was obtained as brown solid (197 mg, 75%). MS (ESI$^+$) m/z 264.01 $[M+H]^+$.

Diethyl 1-[2-(3-chloro-4-isopropoxyphenyl)pyrimidin-5-ylcarbamoyl]-3-methylbutylphosphonate (34).

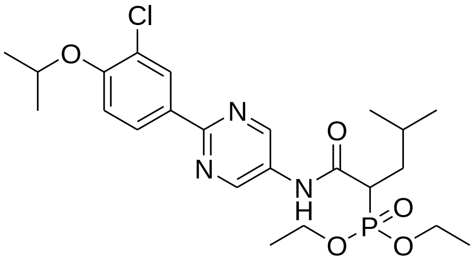

Compound **34** was synthesized according to general procedure A-2, using **32** (132 mg, 0.50 mmol), 2-(diethoxy-phosphoryl)-4-methyl-pentanoic acid **29** (252 mg, 1 mmol) and EDC·HCl (191 mg, 1 mmol), HOBt (135 mg, 1 mmol) and DIPEA (205 µL, 1.2 mmol) in DCM (20 mL). The reaction was stirred at room temperature for 24 h. The crude product was purified by automated column chromatography (DCM to DCM/MeOH 1%). The product was obtained as white solid (229 mg, 92%). ^{1}H NMR (500 MHz, $CDCl_3$) δ ppm: 10.43 (s, 1H), 8.77 (s, 2H), 8.01 (d, *J* = 2.1 Hz, 1H), 7.77 (dd, *J* = 8.6, 2.1 Hz, 1H), 6.71 (d, *J* = 8.8 Hz, 1H), 4.52 (dq, *J* = 12.2, 6.1 Hz, 1H), 4.08 – 3.99 (m, 4H), 3.38 (ddd, *J* = 22.8, 11.4, 2.8 Hz, 1H), 2.21 – 2.04 (m, 1H), 1.54 (dtd, *J* = 13.2, 10.3, 5.0 Hz, 1H), 1.47 – 1.39 (m, 1H), 1.32 (d, *J* = 6.0 Hz, 6H), 1.26 (t, *J* = 7.1 Hz, 6H), 0.88 (d, *J* = 6.5 Hz, 3H), 0.85 (d, *J* = 6.6 Hz, 3H). ^{13}C NMR (126 MHz, $CDCl_3$) δ ppm: 167.0 (d, *J* = 3.6 Hz), 158.3, 154.9, 147.0, 131.7, 130.5, 129.6, 127.2, 123.6, 114.2, 71.6, 64.6 (d, *J* = 6.3 Hz), 62.0 (d, *J* = 7.3 Hz), 45.1 (d, *J* = 130.3 Hz), 36.8 (d, *J* = 5.8 Hz), 26.6 (d, *J* = 14.8 Hz), 23.2, 22.1, 22.0, 21.2, 16.4 (d, *J* = 5.9 Hz), 16.3 (d, *J* = 6.4 Hz). ^{31}P NMR (202 MHz, $CDCl_3$) δ ppm: 25.19. MS (ESI$^+$) m/z 498.13 $[M+H]^+$.

4-(4-Nitrophenyl)morpholine (38).

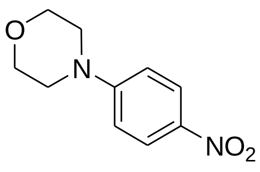

Compound **38** was synthesized according to general procedure E, using 1-fluoro-4-nitrobenzene (282 mg, 2 mmol), morpholine (210 mg, 2.4 mmol), and K_2CO_3 (414 mg, 3 mmol) in DMF (20 mL). The product was used in the next step without further purification. Yield (300 mg, 72%). MS (ESI$^+$) m/z 209.03 $[M+H]^+$.

4-Morpholinobenzenamine (52).

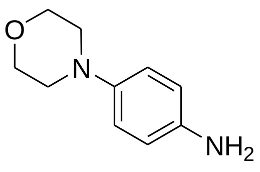

Compound **52** was synthesized according to general procedure F, using **38** (208 mg, 1 mmol), Fe (280 mg, 5 mmol), and NH_4Cl (28 mg, 0.5 mmol) in ethanol/water (2/1) mixture (15 mL). The crude product was purified using column chromatography (DCM to DCM/MeOH 5%). Yield (108 mg, 61%). ^{1}H

NMR (500 MHz, Acetone) δ ppm: 6.78 (d, J = 8.7 Hz, 2H), 6.49 – 6.33 (m, 2H), 3.64 (dd, J = 6.4, 3.0 Hz, 4H), 2.94 (dd, J = 6.5, 2.9 Hz, 4H). ^{13}C NMR (126 MHz, Acetone) δ ppm: 147.7, 144.4, 120.3, 116.3, 66.6, 49.9. MS (ESI$^+$) m/z 179.03 [M+H]$^+$.

Diethyl 1-(4-morpholinophenylcarbamoyl)-3-methylbutylphosphonate (66).

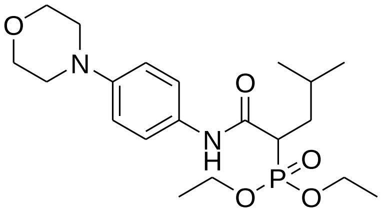

Compound **66** was synthesized according to general procedure A-2, using **52** (89 mg, 0.50 mmol), 2-(diethoxy-phosphoryl)-4-methyl-pentanoic acid **29** (252 mg, 1 mmol) and EDC·HCl (191 mg, 1 mmol), HOBt (135 mg, 1 mmol) and DIPEA (205 µL, 1.2 mmol) in DCM (20 mL). The reaction was stirred at room temperature for 24 h. The crude product was purified by automated column chromatography DCM to DCM/MeOH 2%. The product was obtained as white solid (168 mg, 82%). MS (ESI$^+$) m/z 413.03 [M+H]$^+$.

***tert*-Butyl 4-(4-Nitrophenyl)piperazine-1-carboxylate (39).**

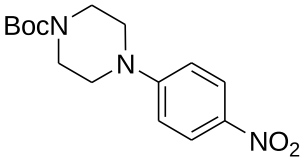

Compound **39** was synthesized according to general procedure E, using 1-fluoro-4-nitrobenzene (282 mg, 2 mmol), *tert*-butyl piperazine-1-carboxylate (446 mg, 2.4 mmol), and K_2CO_3 (414 mg, 3 mmol) in DMF (20 mL). The product was used in the next step without further purification. Yield (400 mg, 65%). MS (ESI$^+$) m/z 308.03 [M+H]$^+$.

***tert*-Butyl 4-(4-Aminophenyl)piperazine-1-carboxylate (53).**

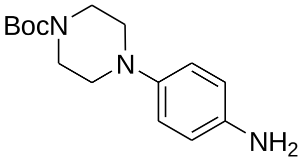

Compound **53** was synthesized according to general procedure F, using **39** (307 mg, 1 mmol), Fe (280 mg, 5 mmol), and NH_4Cl (28 mg, 0.5 mmol) in ethanol/water (2/1) mixture (15 mL). The crude product was purified using column chromatography (DCM to DCM/MeOH 5%). Yield (145 mg, 52%). ^{1}H NMR (500 MHz, DMSO) δ ppm: 6.70 (d, J = 8.7 Hz, 2H), 6.50 (d, J = 8.7 Hz, 2H), 4.75 (s, 2H), 3.45 – 3.37 (m, 4H), 2.84 – 2.80 (m, 4H), 1.41 (s, 9H). ^{13}C NMR (126 MHz, DMSO) δ ppm: 154.3, 143.0, 143.8, 119.2, 115.2, 79.3, 51.2, 28.5. MS (ESI$^+$) m/z 278.05 [M+H]$^+$.

Diethyl 1-{4-[4-(*tert*-butoxycarbonyl)piperazin-1-yl]phenylcarbamoyl}-3-methylbutylphosphonate (67).

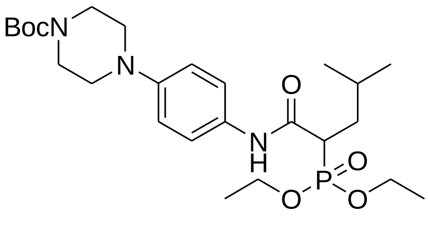

Compound **67** was synthesized according to general procedure A-2, using **53** (138 mg, 0.50 mmol), 2-(diethoxy-phosphoryl)-4-methyl-pentanoic acid **29** (252 mg, 1 mmol) and EDC·HCl (191 mg, 1 mmol), HOBt (135 mg, 1 mmol) and DIPEA (205 µL, 1.2 mmol) in DCM (20 mL). The reaction was stirred at room temperature for 24 h. The crude product was purified by automated column chromatography (DCM to DCM/MeOH 2%). The product was obtained as white solid (214 mg, 84%). MS (ESI$^+$) m/z 512.12 [M+H]$^+$.

1-Methyl-4-(4-nitrophenyl)piperazine (40).

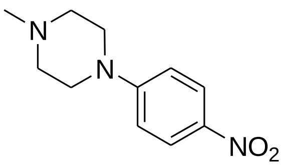

Compound **40** was synthesized according to general procedure E, using 1-fluoro-4-nitrobenzene (282 mg, 2 mmol), 1-methylpiperazine (240 mg, 2.4 mmol), and K_2CO_3 (414 mg, 3 mmol) in DMF (20 mL). The product was used in the next step without further purification. Yield (270 mg, 61%). MS (ESI$^+$) m/z 222.03 [M+H]$^+$.

4-(4-Methylpiperazin-1-yl)benzenamine (54).

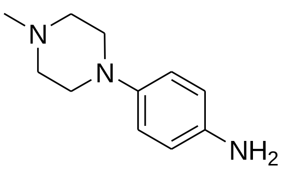

Compound **54** was synthesized according to general procedure F, using **40** (221 mg, 1 mmol), Fe (280 mg, 5 mmol), and NH_4Cl (28 mg, 0.5 mmol) in Ethanol/water (2/1) mixture (15 mL). The crude product was purified using column chromatography DCM to DCM/MeOH 5%. Yield (105 mg, 55%). ^{1}H NMR (500 MHz, DMSO) δ ppm: 6.73 (d, *J* = 8.8 Hz, 2H), 6.54 (d, *J* = 8.8 Hz, 2H), 4.60 (s, 2H), 2.95 (t, *J* = 4.8 Hz, 4H), 2.50 – 2.44 (m, 4H), 2.25 (s, 3H). ^{13}C NMR (126 MHz, DMSO) δ ppm: 142.9, 142.5, 118.3, 115.2, 55.4, 50.7, 46.3. MS (ESI$^+$) m/z 192.00 [M+H]$^+$.

Diethyl 1-[4-(4-methylpiperazin-1-yl)phenylcarbamoyl]-3-methylbutylphosphonate (68).

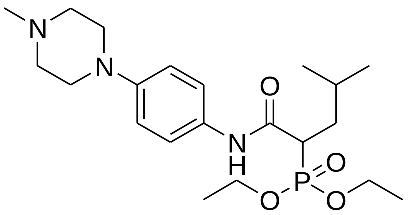

Compound **68** was synthesized according to general procedure A-2, using **52** (95 mg, 0.50 mmol), 2-(diethoxy-phosphoryl)-4-methyl-pentanoic acid **29** (252 mg, 1 mmol) and EDC·HCl (191 mg, 1 mmol), HOBt (135 mg, 1 mmol) and DIPEA (205 µL, 1.2 mmol) in DCM (20 mL). The reaction was stirred at room temperature for 24 h. The crude product was purified by automated column chromatography DCM to DCM/MeOH 2%. The product was obtained as white solid (196 mg, 93%). ^{1}H NMR (500 MHz, $CDCl_3$) δ ppm: 8.46 (s, 1H), 7.33 (dd, *J* = 8.9, 1.2 Hz, 2H), 6.81 – 6.72 (m, 2H), 4.17 – 3.96 (m, 4H), 3.12 – 3.03 (m, 4H), 3.00 – 2.87 (m, 1H), 2.52 – 2.46 (m, 4H), 2.27 (s, 3H), 2.05 – 1.92 (m, 1H), 1.71 – 1.60 (m, 1H), 1.54 – 1.44 (m, 1H), 1.30 – 1.20 (m, 6H), 0.87 (t, *J* = 6.7 Hz, 6H). ^{13}C NMR (126 MHz, $CDCl_3$) δ ppm: 165.4 (d, *J* = 2.5 Hz), 148.2, 130.6, 121.0, 116.6, 63.1 (d, *J* = 6.9 Hz), 62.7 (d, *J* = 6.8 Hz), 55.1, 49.6, 46.2, 45.2 (d, *J* = 128.6 Hz), 35.9 (d, *J* = 4.8 Hz), 26.6 (d, *J* = 14.0 Hz), 23.2, 21.3, 16.4 (d, *J* = 3.3 Hz), 16.4 (d, *J* = 3.6 Hz). ^{31}P NMR (202 MHz, D_2O) δ ppm: 29.67. MS (ESI$^+$) m/z 426.03 [M+H]$^+$.

1-[4-(4-Nitrophenyl)piperazin-1-yl]ethanone (41).

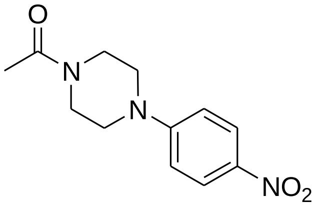

Compound **41** was synthesized according to general procedure E, using 1-fluoro-4-nitrobenzene (282 mg, 2 mmol), 1-(piperazin-1-yl)ethanone (306 mg, 2.4 mmol), and K_2CO_3 (414 mg, 3 mmol) in DMF (20 mL). The product was used in the next step without further purification. Yield (438 mg, 88%). ^{1}H NMR (500 MHz, DMSO) δ ppm: 8.28 – 7.96 (m, 2H), 7.27 – 6.81 (m, 2H), 3.60 (dd, *J* = 10.7, 5.5 Hz, 4H), 3.55 (dd, *J* = 6.7, 3.5 Hz, 2H), 3.51 – 3.42 (m, 2H), 2.05 (s, 3H). ^{13}C NMR (126 MHz, DMSO) δ ppm: 169.0, 154.9, 137.4, 126.2, 113.0, 46.6, 46.3, 45.2, 40.7, 21.7. MS (ESI$^+$) m/z 250.03 [M+H]$^+$.

1-[4-(4-Aminophenyl)piperazin-1-yl]ethanone (55).

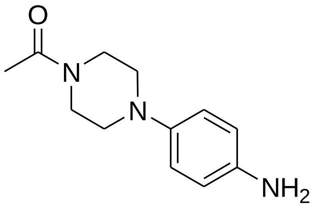

Compound **55** was synthesized according to general procedure F, using **41** (250 mg, 1 mmol), Fe (280 mg, 5 mmol), and NH_4Cl (28 mg, 0.5 mmol) in Ethanol/water (2/1) mixture (15 mL). The crude product was purified using column chromatography (DCM to DCM/MeOH 5%). Yield (125 mg, 57%). ^{1}H NMR (500 MHz, DMSO) δ ppm: 6.71 (d, *J* = 8.7 Hz, 2H), 6.50 (d, *J* = 8.7 Hz, 2H), 4.63 (s, 2H), 3.53 (td, *J* = 8.0, 5.5 Hz, 4H), 2.90 – 2.87 (m, 2H), 2.84 – 2.78 (m, 2H), 2.02 (s, 3H). ^{13}C NMR (126 MHz, DMSO) δ ppm: 168.6, 143.2, 142.6, 119.1, 115.2, 51.6, 51.1, 46.3, 41.5, 21.7. MS (ESI$^+$) m/z 220.14 [M+H]$^+$.

Diethyl 1-[4-(4-acetylpiperazin-1-yl)phenylcarbamoyl]-3-methylbutylphosphonate (69).

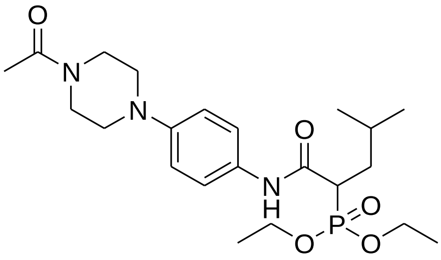

Compound **69** was synthesized according to general procedure A-2, using **55** (110 mg, 0.50 mmol), 2-(diethoxy-phosphoryl)-4-methyl-pentanoic acid **29** (252 mg, 1 mmol) and EDC·HCl (191 mg, 1 mmol), HOBt (135 mg, 1 mmol) and DIPEA (205 µL, 1.2 mmol) in DCM (20 mL). The reaction was stirred at room temperature for 24 h. The crude product was purified by automated column chromatography DCM to DCM/MeOH 2%. The product was obtained as white solid (184 mg, 81%). MS (ESI$^+$) m/z 454.15 [M+H]$^+$.

4-Methyl-1-(4-nitrophenyl)piperidine (42).

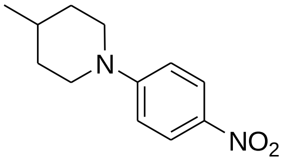

Compound **42** was synthesized according to general procedure E, using 1-fluoro-4-nitrobenzene (282 mg, 2 mmol), 4-methylpiperidine (240 mg, 2.4 mmol), and K_2CO_3 (414 mg, 3 mmol) in DMF (20 mL). The product was used in the next step without further purification. Yield (321 mg, 73%). MS (ESI$^+$) m/z 221.03 [M+H]$^+$.

4-(4-Methylpiperidin-1-yl)benzenamine (56).

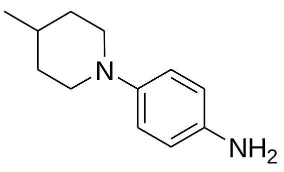

Compound **56** was synthesized according to general procedure F, using **42** (220 mg, 1 mmol), Fe (280 mg, 5 mmol), and NH_4Cl (28 mg, 0.5 mmol) in Ethanol/water (2/1) mixture (15 mL). The crude product was purified using column chromatography (DCM to DCM/MeOH 5%). Yield (130 mg, 68%). ^{1}H NMR (500 MHz, DMSO) δ ppm: 6.73 – 6.62 (m, 2H), 6.51 – 6.42 (m, 2H), 4.53 (s, 2H), 3.30 (d, *J* = 12.0 Hz, 2H), 2.45 (td, *J* = 11.8, 2.2 Hz, 2H), 1.66 (dd, *J* = 9.4, 3.9 Hz, 2H), 1.48 – 1.33 (m, 2H), 1.39 (dtt, *J* = 13.0, 6.4, 3.3 Hz, 1H), 1.24 (qd, *J* = 12.1, 3.8 Hz, 2H), 0.93 (d, *J* = 6.5 Hz, 3H). ^{13}C NMR (126 MHz, DMSO) δ ppm: 143.6, 142.5, 119.1, 115.2, 51.8, 34.6, 30.6, 22.3. MS (ESI$^+$) m/z 191.03 [M+H]$^+$

Diethyl 1-[4-(4-methylpiperidin-1-yl)phenylcarbamoyl]-3-methylbutylphosphonate (70).

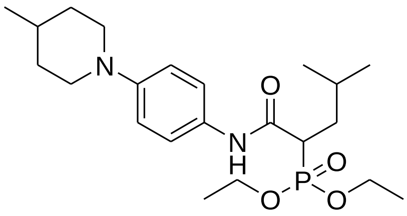

Compound **70** was synthesized according to general procedure A-2, using **56** (95 mg, 0.50 mmol), 2-(diethoxy-phosphoryl)-4-methyl-pentanoic acid **29** (252 mg, 1 mmol) and EDC·HCl (191 mg, 1 mmol), HOBt (135 mg, 1 mmol) and DIPEA (205 µL, 1.2 mmol) in DCM (20 mL). The reaction was stirred at room temperature for 24 h. The crude product was purified by automated column chromatography DCM to DCM/MeOH 1%. The product was obtained as white solid (170 mg, 80%). ^{1}H NMR (500 MHz, $CDCl_3$) δ ppm: 8.35 (s, 1H), 7.35 – 7.28 (m, 2H), 6.81 – 6.73 (m, 2H), 4.17 – 3.96 (m, 4H), 3.49 (d, *J* = 12.2 Hz, 2H), 2.93 (ddd, *J* = 22.6, 11.2, 3.5 Hz, 1H), 2.56 (td, *J* = 12.1, 2.4 Hz, 2H), 1.98 (dddd, *J* = 13.4, 11.3, 8.7, 4.5 Hz, 1H), 1.71 (d, *J* = 3.4 Hz, 1H), 1.65 (d, *J* = 11.8 Hz, 3H), 1.56 – 1.46 (m, 1H), 1.45 – 1.37 (m, 1H), 1.26 (dd, *J* = 13.6, 6.9 Hz, 7H), 0.88 (dd, *J* = 13.3, 9.9 Hz, 6H), 0.87 (s, 3H). ^{13}C NMR (126 MHz, $CDCl_3$) δ ppm: 165.4 (d, *J* = 2.0 Hz), 149.0, 130.1, 121.0, 117.0, 63.0 (d, *J* = 7.0 Hz), 62.8 (d, *J* = 6.7 Hz), 50.5, 50.5, 45.2 (d, *J* = 128.6 Hz), 36.0 (d, *J* = 4.7 Hz), 34.1, 30.7, 26.6 (d, *J* = 14.0 Hz), 23.2, 21.9, 21.3, 16.4 (d, *J* = 3.0 Hz), 16.4 (d, *J* = 2.8 Hz). ^{31}P NMR (202 MHz, D_2O) δ ppm: 27.67 MS (ESI^+) m/z 425.21 $[M+H]^+$.

1-(4-Nitrophenyl)-1*H*-imidazole (43).

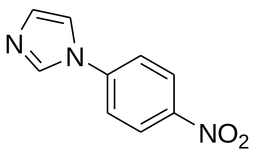

Compound **43** was synthesized according to general procedure E, using 1-fluoro-4-nitrobenzene (282 mg, 2 mmol), 1H-imidazole (164 mg, 2.4 mmol), and K_2CO_3 (414 mg, 3 mmol) in DMF (20 mL). The product was used in the next step without further purification. Yield (200 mg, 53%). ^{1}H NMR (500 MHz, DMSO) δ ppm: 8.51 (s, 1H), 8.38 – 8.33 (m, 2H), 8.01 – 7.97 (m, 2H), 7.96 (s, 1H), 7.19 (s, 1H). ^{13}C NMR (126 MHz, DMSO) δ ppm: 145.7, 142.2, 136.5, 131.3, 126.0, 120.8, 118.4. MS (ESI^+) m/z 190.03 $[M+H]^+$.

4-(1*H*-Imidazol-1-yl)benzenamine (57).

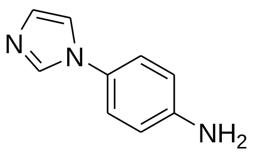

Compound **57** was synthesized according to general procedure F, using **57** (189 mg, 1 mmol), Fe (280 mg, 5 mmol), and NH_4Cl (28 mg, 0.5 mmol) in Ethanol/water (2/1) mixture (15 mL). The crude product was purified using column chromatography (DCM to DCM/MeOH 5%). Yield (95 mg, 60%). ^{1}H NMR (500 MHz, Acetone) δ ppm: 7.81 (s, 1H), 7.36 (s, 1H), 7.29 – 7.20 (m, 2H), 7.04 (s, 1H), 6.81 – 6.75 (m, 2H), 4.88 (s, 2H). MS (ESI^+) m/z 160.03 $[M+H]^+$.

Diethyl 1-[4-(1*H*-imidazol-1-yl)phenylcarbamoyl]-3-methylbutylphosphonate (71).

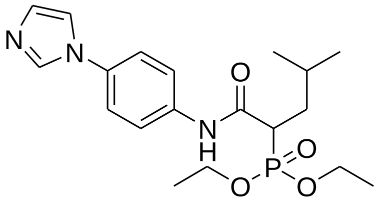

Compound **71** was synthesized according to general procedure A-2, using **57** (80 mg, 0.50 mmol), 2-(diethoxy-phosphoryl)-4-methyl-pentanoic acid **29** (252 mg, 1 mmol) and EDC·HCl (191 mg, 1 mmol), HOBt (135 mg, 1 mmol) and DIPEA (205 µL, 1.2 mmol) in DCM (20 mL). The reaction was stirred at room temperature for 24 h. The

crude product was purified by automated column chromatography DCM to DCM/MeOH 2%. The product was obtained as white solid (178 mg, 90%). ^{1}H NMR (500 MHz, $CDCl_3$) δ ppm: 10.05 (s, 1H), 7.60 (s, 1H), 7.52 (d, *J* = 8.8 Hz, 2H), 7.04 (d, *J* = 25.5 Hz, 2H), 6.98 (d, *J* = 18.4 Hz, 2H), 4.28 – 4.15 (m, 2H), 4.10 (ddd, *J* = 8.4, 7.3, 0.9 Hz, 2H), 3.25 (ddd, *J* = 22.5, 11.4, 2.9 Hz, 1H), 2.17 – 2.03 (m, 1H), 1.59 (ddd, *J* = 10.6, 8.4, 5.6 Hz, 1H), 1.46 (dtd, *J* = 13.2, 10.1, 3.0 Hz, 1H), 1.33 (t, *J* = 7.1 Hz, 3H), 1.30 (t, *J* = 7.1 Hz, 3H), 0.88 (d, *J* = 6.6 Hz, 3H), 0.86 (d, *J* = 6.5 Hz, 3H). ^{31}P NMR (202 MHz, $CDCl_3$) δ ppm: 25.87. MS (ESI$^+$) m/z 394.03 [M+H]$^+$.

1-(4-Nitrophenyl)-1*H*-pyrazole (44).

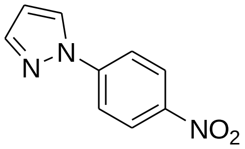

Compound **44** was synthesized according to general procedure E, using 1-fluoro-4-nitrobenzene (282 mg, 2 mmol), 1*H*-pyrazole (210 mg, 2.4 mmol), and K_2CO_3 (414 mg, 3 mmol) in DMF (20 mL). The product was used in the next step without further purification. Yield (313 mg, 83%). MS (ESI$^+$) m/z 190.12 [M+H]$^+$.

4-(1*H*-Pyrazol-1-yl)benzenamine (58).

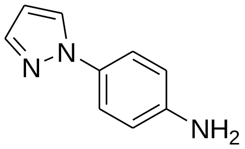

Compound **58** was synthesized according to general procedure F, using **44** (189 mg, 1 mmol), Fe (280 mg, 5 mmol), and NH_4Cl (28 mg, 0.5 mmol) in Ethanol/water (2/1) mixture (15 mL). The crude product was purified using column chromatography (DCM to DCM/MeOH 5%). Yield (85 mg, 53%). MS (ESI$^+$) m/z 160.03 [M+H]$^+$.

Diethyl 1-[4-(1*H*-pyrazol-1-yl)phenylcarbamoyl]-3-methylbutylphosphonate (72).

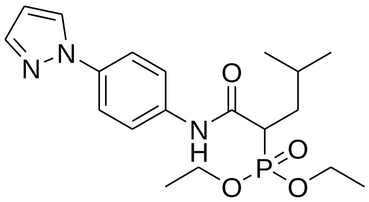

Compound **72** was synthesized according to general procedure A-2, using **58** (80 mg, 0.50 mmol), 2-(diethoxy-phosphoryl)-4-methyl-pentanoic acid **29** (252 mg, 1 mmol) and EDC·HCl (191 mg, 1 mmol), HOBt (135 mg, 1 mmol) and DIPEA (205 µL, 1.2 mmol) in DCM (20 mL). The reaction was stirred at room temperature for 24 h. The crude product was purified by automated column chromatography (DCM to DCM/MeOH 2%). The product was obtained as white solid (169 mg, 85%). ^{1}H NMR (500 MHz, DMSO) δ ppm: 10.19 (s, 1H), 8.41 (d, *J* = 2.4 Hz, 1H), 7.75 – 7.71 (m, 2H), 7.69 – 7.67 (m, 2H), 7.65 (d, *J* = 1.6 Hz, 1H), 6.49 – 6.47 (m, 1H), 4.17 – 3.96 (m, 4H), 3.03 (ddd, *J* = 22.3, 11.1, 2.6 Hz, 1H), 2.02 – 1.95 (m, 1H), 1.57 – 1.38 (m, 2H), 0.88 (d, *J* = 1.7 Hz, 3H),), 0.88 (dd, *J* = 13.3, 9.9 Hz, 6H), 0.80 (d, *J* = 1.8 Hz, 3H). MS (ESI$^+$) m/z 394.03 [M+H]$^+$.

2-(4-Nitrophenyl)-2*H*-1,2,3-triazole (45).

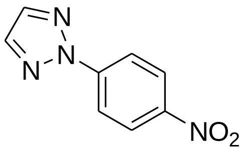

Compound **45** was synthesized according to general procedure E, using 1-fluoro-4-nitrobenzene (282 mg, 2 mmol), 1*H*-1,2,3-triazole (210 mg, 2.4 mmol), and K_2CO_3 (414 mg, 3 mmol) in DMF (20 mL). The product was used in the next step without further purification. Yield (266 mg, 70%). ^{1}H NMR (500 MHz,

DMSO) δ ppm: 8.44 (d, *J* = 2.1 Hz, 1H), 8.43 (d, *J* = 2.1 Hz, 1H), 8.30 – 8.26 (m, 4H). ^{13}C NMR (126 MHz, DMSO) δ ppm: 146.5, 143.5, 138.7, 126.1, 119.5. MS (ESI$^+$) m/z 191.11 [M+H.

4-(2*H*-1,2,3-Triazol-1-yl)benzenamine (59).

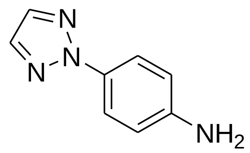

Compound **59** was synthesized according to general procedure F, using **45** (190 mg, 1 mmol), Fe (280 mg, 5 mmol), and NH_4Cl (28 mg, 0.5 mmol) in Ethanol/water (2/1) mixture (15 mL). The crude product was purified using column chromatography (DCM to DCM/MeOH 5%). The product was obtained as white solid (85 mg, 53%). ^{1}H NMR (500 MHz, DMSO) δ ppm: 8.01 (s, 2H), 7.81 – 7.46 (m, 2H), 6.81 – 6.48 (m, 2H), 5.48 (s, 2H). ^{13}C NMR (126 MHz, DMSO) δ ppm: 149.2, 135.4, 129.8, 120.4, 114.2. MS (ESI$^+$) m/z 161.03 [M+H]$^+$.

Diethyl 1-[4-(2*H*-1,2,3-triazol-1-yl)phenylcarbamoyl]-3-methylbutylphosphonate (73).

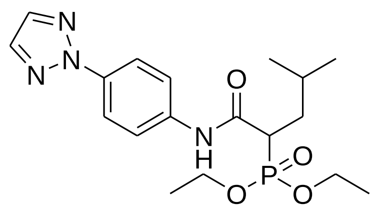

Compound **73** was synthesized according to general procedure A-2, using **59** (80 mg, 0.50 mmol), 2-(diethoxy-phosphoryl)-4-methyl-pentanoic acid **29** (252 mg, 1 mmol) and EDC·HCl (191 mg, 1 mmol), HOBt (135 mg, 1 mmol) and DIPEA (205 µL, 1.2 mmol) in DCM (20 mL). The reaction was stirred at room temperature for 24 h. The crude product was purified by automated column chromatography (DCM to DCM/MeOH 2%). The product was obtained as white solid (175 mg, 89%). ^{1}H NMR (500 MHz, DMSO) δ ppm: 10.37 (s, 1H), 8.09 (s, 2H), 8.03 – 7.87 (m, 2H), 7.79 – 7.70 (m, 2H), 4.21 – 3.88 (m, 4H), 3.24 (ddd, *J* = 22.5, 11.2, 3.0 Hz, 1H), 2.00 (tdd, *J* = 15.7, 8.2, 4.4 Hz, 1H), 1.51 (tt, *J* = 17.4, 6.5 Hz, 1H), 1.43 (dtd, *J* = 13.1, 10.3, 3.0 Hz, 1H), 1.24 (t, *J* = 4.5 Hz, 3H), 1.21 (t, *J* = 4.5 Hz, 3H), 0.90 (d, *J* = 2.9 Hz, 3H), 0.89 (d, *J* = 3.0 Hz, 3H). ^{13}C NMR (126 MHz, DMSO) δ ppm: 167.1 (d, *J* = 5.2 Hz), 138.9, 136.6, 135.2, 120.4, 119.6, 62.5 (d, *J* = 6.7 Hz), 62.3 (d, *J* = 7.1 Hz), 45.1 (d, *J* = 129.9 Hz), 35.8 (d, *J* = 5.3 Hz), 26.8 (d, *J* = 15.1 Hz), 23.4, 21.7, 16.7 (d, *J* = 5.4 Hz). ^{31}P NMR (202 MHz, DMSO) δ ppm: 24.61. MS (ESI$^+$) m/z 395.03 [M+H]$^+$.

1-(4-Nitrophenyl)-1*H*-1,2,4-triazole (46).

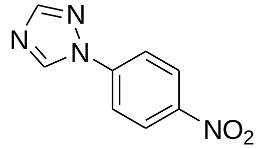

Compound **46** was synthesized according to general procedure E, using 1-fluoro-4-nitrobenzene (282 mg, 2 mmol), 1*H*-1,2,4-triazole (210 mg, 2.4 mmol), and K_2CO_3 (414 mg, 3 mmol) in DMF (20 mL). The product was used in the next step without further purification. Yield (262 mg, 69%). ^{1}H NMR (500 MHz, DMSO) δ ppm: 9.54 (s, 1H), 8.51 – 8.41 (m, 2H), 8.36 (s, 1H), 8.23 – 8.13 (m, 2H). ^{13}C NMR (126 MHz, DMSO) δ ppm: 153.7, 146.5, 144.0, 141.6, 126.0, 120.3. MS (ESI$^+$) m/z 191.03 [M+H]$^+$.

4-(1*H*-1,2,4-Triazol-1-yl)benzenamine (60).

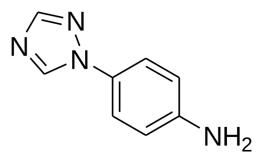

Compound **60** was synthesized according to general procedure F, using **46** (190 mg, 1 mmol), Fe (280 mg, 5 mmol), and NH_4Cl (28 mg, 0.5 mmol) in Ethanol/water (2/1) mixture (15 mL). The crude product was purified using column chromatography (DCM to DCM/MeOH 5%). Yield (120 mg, 74%). ^{1}H

NMR (500 MHz, DMSO) δ ppm: 9.03 (s, 1H), 8.16 (s, 1H), 7.58 – 7.30 (m, 2H), 6.83 – 6.56 (m, 2H), 5.45 (s, 2H). ^{13}C NMR (126 MHz, DMSO) δ ppm: 152.1, 149.2, 141.8, 126.6, 121.6, 114.3. MS (ESI$^+$) m/z 161.11 [M+H]$^+$.

Diethyl 1-[4-(1*H*-1,2,4-triazol-1-yl)phenylcarbamoyl]-3-methylbutylphosphonate (74).

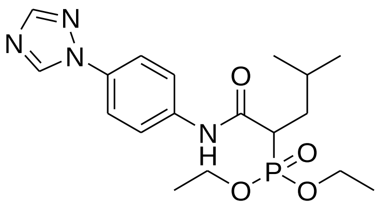

Compound **74** was synthesized according to general procedure A-2, using **60** (80 mg, 0.50 mmol), 2-(diethoxy-phosphoryl)-4-methyl-pentanoic acid **29** (252 mg, 1 mmol) and EDC·HCl (191 mg, 1 mmol), HOBt (135 mg, 1 mmol) and DIPEA (205 µL, 1.2 mmol) in DCM (20 mL). The reaction was stirred at room temperature for 24 h. The crude product was purified by automated column chromatography (DCM to DCM/MeOH 2%). The product was obtained as white solid (180 mg, 91%). ^1H NMR (500 MHz, DMSO) δ ppm: 10.37 (s, 1H), 9.22 (s, 1H), 8.21 (s, 1H), 7.81 (dd, *J* = 9.2, 2.2 Hz, 2H), 7.76 (dd, *J* = 9.2, 2.2 Hz, 2H), 4.14 – 3.96 (m, 4H), 3.23 (ddd, *J* = 22.5, 11.2, 3.0 Hz, 1H), 2.00 (tdd, *J* = 15.7, 8.2, 4.4 Hz, 1H), 1.56 – 1.47 (m, 1H), 1.43 (ddd, *J* = 16.3, 11.6, 5.2 Hz, 1H), 1.24 (t, *J* = 4.7 Hz, 3H), 1.21 (t, *J* = 4.7 Hz, 3H), 0.90 (d, *J* = 3.2 Hz, 3H), 0.88 (d, *J* = 3.2 Hz, 3H). ^{13}C NMR (126 MHz, DMSO) δ ppm: 167.1 (d, *J* = 4.6 Hz), 152.7, 142.4, 138.9, 132.7, 120.6, 120.4, 62.5 (d, *J* = 6.4 Hz), 62.3 (d, *J* = 6.5 Hz), 45.0 (d, *J* = 130.0 Hz), 35.8 (d, *J* = 4.7 Hz), 26.8 (d, *J* = 14.8 Hz), 23.4, 21.7 (s), 16.7 (d, *J* = 5.6 Hz). ^{31}P NMR (202 MHz, DMSO) δ ppm: 24.60. MS (ESI$^+$) m/z 395.03 [M+H]$^+$.

1-(4-Nitrophenyl)-1*H*-benzo[d]imidazole (47).

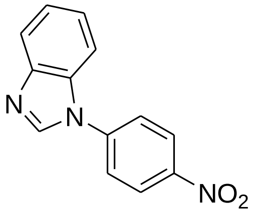

Compound **47** was synthesized according to general procedure E, using 1-fluoro-4-nitrobenzene (282 mg, 2 mmol), 1*H*-benzo[d]imidazole (284 mg, 2.4 mmol), and K_2CO_3 (414 mg, 3 mmol) in DMF (20 mL). The product was used in the next step without further purification. Yield (286 mg, 60%). ^1H NMR (500 MHz, DMSO) δ ppm: 8.75 (s, 1H), 8.50 – 8.42 (m, 2H), 8.06 – 8.00 (m, 2H), 7.82 (d, *J* = 7.3 Hz, 1H), 7.78 (d, *J* = 7.5 Hz, 1H), 7.39 (dtd, *J* = 16.3, 7.3, 1.1 Hz, 2H). ^{13}C NMR (126 MHz, DMSO) δ ppm: 146.2, 144.6, 143.8, 141.8, 132.8, 126.0, 124.6, 124.3, 123.7, 120.7, 111.5. MS (ESI$^+$) m/z 240.03 [M+H]$^+$.

4-(1*H*-Benzo[d]imidazol-1-yl)benzenamine (61).

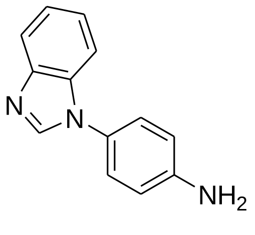

Compound **61** was synthesized according to general procedure F, using **47** (239 mg, 1 mmol), Fe (280 mg, 5 mmol), and NH_4Cl (28 mg, 0.5 mmol) in Ethanol/water (2/1) mixture (15 mL). The crude product was purified using column chromatography (DCM to DCM/MeOH 5%). Yield (133 mg, 63%). ^1H NMR (500 MHz, DMSO) δ ppm: 8.34 (s, 1H), 7.73 (d, *J* = 7.2 Hz, 1H), 7.45 (d, *J* = 7.3 Hz, 1H), 7.26 (dd, *J* = 13.3, 7.3 Hz, 4H), 6.75 (d, *J* = 8.2 Hz, 2H), 5.44 (s, 2H). ^{13}C NMR (126 MHz, DMSO) δ ppm: 149.3, 143.9, 143.9, 134.4, 125.6, 124.6, 123.4, 122.4, 120.2, 114.8, 111.0. MS (ESI$^+$) m/z 210.03 [M+H]$^+$.

Diethyl 1-[4-(1*H*-benzo[d]imidazol-1-yl)phenylcarbamoyl]-3-methylbutylphosphonate (75).

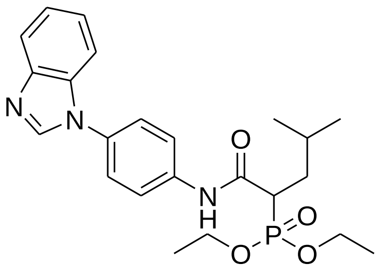

Compound **75** was synthesized according to general procedure A-2, using **61** (105 mg, 0.50 mmol), 2-(diethoxy-phosphoryl)-4-methyl-pentanoic acid **29** (252 mg, 1 mmol) and EDC·HCl (191 mg, 1 mmol), HOBt (135 mg, 1 mmol) and DIPEA (205 µL, 1.2 mmol) in DCM (20 mL). The reaction was stirred at room temperature for 24 h. The crude product was purified by automated column chromatography (DCM to DCM/MeOH 2%). The product was obtained as white solid (189 mg, 85%). MS (ESI$^+$) m/z 444.15 [M+H]$^+$.

***N*-(4-Aminophenyl)-3,4-dichlorobenzamide (175).**

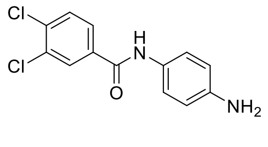

Compound **175** was synthesized according to general procedure A-1, using 3,4-dichlorobenzoic acid (363 mg, 1.90 mmol), *N*-Boc-*p*-phenylendiamine (330 mg, 1.58 mmol) and EDC·HCl (364 mg, 1.90 mmol) in DCM (20 mL). The reaction was stirred at r.t. overnight. Solid was filtered and washed with DCM, to give *tert*-butyl [4-(3,4-dichlorobenzamido)phenyl]carbamate (393 mg, 65%). In the next step Boc-protected aniline (393 mg, 1.03 mmol) was treated with TFA (0.5 mL) in DCM (15 mL) to afford the product as a beige solid (212 mg, 73%; 47% over 2 steps). ^{1}H NMR (500 MHz, DMSO-d_6) δ ppm 10.05 (s, 1 H), 8.17 (d, *J*=2.14 Hz, 1 H), 7.90 (dd, *J*=8.39, 1.98 Hz, 1 H), 7.79 (d, *J*=8.39 Hz, 1 H), 7.36 (d, *J*=8.70 Hz, 2 H), 6.52 - 6.56 (m, 2 H), 4.99 (s, 2 H). ^{13}C NMR (126 MHz, DMSO-d$_6$) δ ppm 162.2, 145.6, 135.6, 133.9, 131.2, 130.7, 129.4, 127.9, 127.6, 122.3, 113.7. MS (ESI$^+$) m/z 280.91 [M+H]$^+$.

***N*-[4-(2-Bromo-4-methylpentanamido)phenyl]-3,4-dichlorobenzamide (184).**

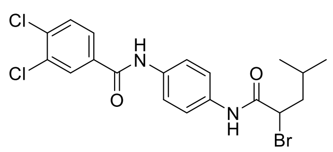

Compound **184** was synthesized according to general procedure A-1, using 2-bromo-4-methylpentanoic acid (318 mg, 1.63 mmol), **175** (383 mg, 1.36 mmol) and EDC·HCl (313 mg, 1.63 mmol) in DCM (40 mL). The reaction was stirred at r.t. overnight. Solid was filtered and washed with DCM to afford the product as a white solid (376 mg, 60%). ^{1}H NMR (500 MHz, DMSO-d$_6$) δ 10.43 (d, *J* = 5.5 Hz, 2H), 8.21 (d, *J* = 2.1 Hz, 1H), 7.94 (dd, *J* = 8.4, 2.0 Hz, 1H), 7.82 (d, *J* = 8.4 Hz, 1H), 7.75 – 7.70 (m, *J* = 9.0 Hz, 2H), 7.63 – 7.56 (m, 2H), 4.65 (t, *J* = 7.6 Hz, 1H), 1.95 – 1.82 (m, 2H), 1.65 (dt, *J* = 13.4, 6.7 Hz, 1H), 0.94 (d, *J* = 6.7 Hz, 3H), 0.89 (d, *J* = 6.6 Hz, 3H). ^{13}C NMR (126 MHz, DMSO-d$_6$) δ 166.8, 163.0, 135.2, 134.7, 134.6, 134.4, 131.3, 130.8, 129.6, 128.1, 121.0, 119.6, 48.6, 42.9, 26.2, 22.2, 21.7. MS (ESI$^+$) m/z 456.95 [M+H]$^+$.

***N*-(4-Aminophenyl)thiophene-2-carboxamide (178).**

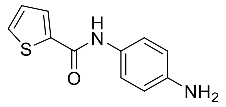

Compound **178** was synthesized according to general procedure A-1, using thiophene-2-carboxylic acid (1.50 g, 11.70 mmol), *N*-Boc-*p*-phenylendiamine (2.03 g, 9.73 mmol) and EDC·HCl (2.24 g, 11.70 mmol) in DCM (60 mL). The reaction was stirred at r.t. overnight. Solid was filtered and washed with DCM. In the next step Boc-protected aniline (1.34 g, 4.21 mmol) was treated with TFA (2.0 mL) in DCM (30 mL) to afford the

product as a beige solid (797 mg, 86%; 31% over 2 steps). ^{1}H NMR (500 MHz, DMSO) δ 10.31 (s, 1H), 8.07 – 7.97 (m, 1H), 7.86 (dd, *J* = 5.0, 0.8 Hz, 1H), 7.71 (d, *J* = 8.8 Hz, 2H), 7.23 (dd, *J* = 4.9, 3.8 Hz, 1H), 7.16 (d, *J* = 8.8 Hz, 2H). ^{13}C NMR (126 MHz, DMSO) δ 159.9, 158.1, 139.9, 136.1, 132.1, 129.3, 128.2, 121.7, 121.4. MS (ESI$^+$) m/z 218.95 [M+H]$^+$.

***N*-[4-(2-Bromo-4-methylpentanamido)phenyl]thiophene-2-carboxamide (187).**

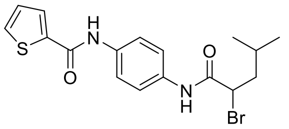

Compound **187** was synthesized according to general procedure A-1, using 2-bromo-4-methylpentanoic acid (375 mg, 1.92 mmol), **178** (349 mg, 1.60 mmol) and EDC·HCl (369 mg, 1.92 mmol) in DCM (30 mL). The reaction was stirred at r.t. overnight. Solid was filtered and washed with DCM to afford the product as a white solid (147 mg, 23%). ^{1}H NMR (500 MHz, DMSO) δ 10.39 (s, 1H), 10.25 (s, 1H), 8.01 (dd, *J* = 3.8, 1.0 Hz, 1H), 7.85 (dd, *J* = 5.0, 1.0 Hz, 1H), 7.71 – 7.65 (m, 2H), 7.63 – 7.54 (m, 2H), 7.22 (dd, *J* = 4.9, 3.8 Hz, 1H), 4.63 (t, *J* = 7.6 Hz, 1H), 1.89 (dtd, *J* = 21.5, 14.1, 7.5 Hz, 2H), 1.65 (dt, *J* = 13.4, 6.7 Hz, 1H), 0.94 (d, *J* = 6.7 Hz, 3H), 0.89 (d, *J* = 6.6 Hz, 3H). ^{13}C NMR (126 MHz, DMSO) δ 166.8, 159.8, 140.1, 134.7, 134.4, 131.9, 129.1, 128.2, 121.0, 119.7, 48.6, 42.9, 26.2, 22.2, 21.7. MS (ESI$^+$) m/z 394.84 [M+H]$^+$.

***N*-(3-Aminophenyl)-3,4-dichlorobenzamide (200).**

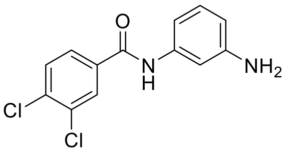

Compound **200** was synthesized according to general procedure A-1, using 3,4-dichlorobenzoic acid (550 mg, 2.88 mmol), *N*-Boc-*m*-phenylendiamine (500 mg, 2.40 mmol) and EDC·HCl (552 mg, 2.88 mmol) in DCM (30 mL). The reaction was stirred at r.t. for 3 h. Crude was purified using column chromatography (PB/EtOAc=9/1 to 8/1) to give *tert*-butyl [3-(3,4-dichlorobenzamido)phenyl]carbamate (742 mg, 81%). In the next step Boc-protected aniline (295 mg, 0.77 mmol) was treated with TFA (0.7 mL) in DCM (3 mL) to afford the product as an off-white solid (72.2 mg, 93%; 75% over 2 steps). ^{1}H NMR (500 MHz, DMSO) δ 10.09 (s, 1H), 8.17 (d, *J*=2.1 Hz, 1H), 7.90 (dd, *J*=8.4, 2.1 Hz, 1H), 7.79 (d, *J*=8.4 Hz, 1H), 7.07 (t, *J*=2.1 Hz, 1H), 6.97 (t, *J*=7.9 Hz, 1H), 6.89 – 6.80 (m, 1H), 6.33 (ddd, *J*=8.0, 2.3, 1.0 Hz, 1H), 5.12 (s, 2H). ^{13}C NMR (126 MHz, DMSO) δ 162.9, 149.0, 139.3, 135.6, 134.1, 131.2, 130.7, 129.6, 128.9, 128.0, 110.1, 108.4, 106.1. MS (ESI$^+$) m/z 280.98 [M+H]$^+$.

Diethyl (1-{[3-(3,4-dichlorobenzamido)phenyl]amino}-4-methyl-1-oxopentan-2-yl)phosphonate (202).

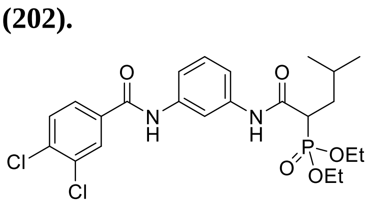

Compound **202** was synthesized according to general procedure A-2, using **214** (147 mg, 0.58 mmol), **200** (109 mg, 0.39 mmol), EDC·HCl (147 mg, 0.77 mmol), HOBt (104 mg, 0.77 mmol) and DIPEA (160 µL, 0.94 mmol) in DCM (5 mL). The reaction was stirred at r.t. overnight. Crude was purified using column chromatography (PB/EtOAc=1/1) to give diethyl (1-{[3-(3,4-dichlorobenzamido)phenyl]amino}-4-methyl-1-oxopentan-2-yl)phosphonate as a colorless solid (99 mg, 49%). ^{1}H NMR (500 MHz, DMSO) δ 10.42 (s, 1H), 10.19 (s, 1H), 8.23 (d, *J*=2.1 Hz, 1H), 8.14

(d, J=2.1 Hz, 1H), 7.94 (dd, J=8.4, 2.1 Hz, 1H), 7.82 (d, J=8.3 Hz, 1H), 7.46 (dt, J=8.0, 1.6 Hz, 1H), 7.34 (dt, J=8.2, 1.5 Hz, 1H), 7.29 (t, J=8.0 Hz, 1H), 4.13 – 3.96 (m, 4H), 3.28 – 3.21 (m, 1H), 2.05 – 1.92 (m, 1H), 1.50 (dtd, J=13.4, 6.7, 4.6 Hz, 1H), 1.40 (dtd, J=13.0, 9.9, 2.9 Hz, 1H), 1.22 (q, J=7.2 Hz, 6H), 0.88 (dd, J=6.5, 3.4 Hz, 6H). ^{31}P NMR (202 MHz, DMSO) δ 24.91. MS (ESI$^+$) m/z 515.18 [M+H]$^+$.

***N*-(4-Aminophenyl)isonicotinamide (94).**

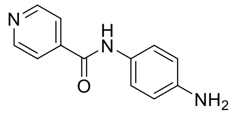

Compound **94** was synthesized according to general procedure A-1, using isonicotinic acid (177 mg, 1.44 mmol), *N*-Boc-*p*-phenylendiamine (250 mg, 1.20 mmol) and EDC·HCl (276 mg, 1.44 mmol) in DCM (15 mL). The reaction was stirred at r.t. overnight. Solid was filtered and washed with DCM to give *tert*-butyl [4-(isonicotinamido)phenyl]carbamate (283 mg, 75%). In the next step Boc-protected aniline (280 mg, 0.89 mmol) was treated with TFA (0.7 mL) in DCM (3 mL) to afford the product as a beige solid (67.3 mg, 35%; 26% over 2 steps). ^{1}H NMR (500 MHz, DMSO) δ 10.12 (s, 1H), 8.74 (dd, J = 4.4, 1.6 Hz, 2H), 7.82 (dd, J = 4.4, 1.6 Hz, 2H), 7.44 – 7.29 (m, 2H), 6.60 – 6.51 (m, 2H), 4.99 (s, 2H). ^{13}C NMR (126 MHz, DMSO) δ 162.9, 150.2, 145.7, 142.3, 127.5, 122.3, 121.5, 113.7. MS (ESI$^+$) m/z 214.14 [M+H]$^+$.

Diethyl {1-[(4-(isonicotinamido)phenyl]amino}-4-methyl-1-oxopentan-2-yl)phosphonate (112).

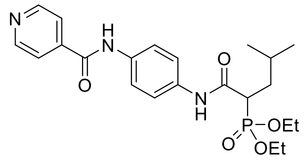

Compound **112** was synthesized according to general procedure A-2, using **214** (115 mg, 0.46 mmol), **94** (65 mg, 0.30 mmol), EDC·HCl (115 mg, 0.60), HOBt (81 mg, 0.60 mmol) and DIPEA (140 µL, 1.28 mmol) in DCM/DMF (3 mL/1 mL). The reaction was stirred at r.t. for overnight. After the workup, the product was obtained as a yellow foam (105.4 mg, 78%). ^{1}H NMR (500 MHz, $CDCl_3$) δ 9.57 (s, 1H), 8.75 (d, J = 5.9 Hz, 2H), 8.51 (s, 1H), 7.75 (d, J = 5.9 Hz, 2H), 7.39 (d, J = 8.8 Hz, 2H), 7.31 (d, J = 8.7 Hz, 2H), 4.29 – 4.03 (m, 4H), 3.25 – 3.16 (m, 1H), 2.19 – 2.04 (m, 1H), 1.67 – 1.61 (m, 1H), 1.55 – 1.44 (m, J = 13.2, 10.2, 3.1 Hz, 1H), 1.36 (q, J = 7.2 Hz, 6H), 0.93 (dd, J = 6.5, 1.0 Hz, 6H). ^{13}C NMR (126 MHz, $CDCl_3$) δ 166.1 (d, J = 2.8 Hz), 163.9, 150.8, 141.9, 135.9, 132.7, 122.0, 121.2, 120.1, 63.9 (d, J = 6.4 Hz), 62.3 (d, J = 7.0 Hz), 45.4 (d, J = 129.7 Hz), 36.1 (d, J = 5.3 Hz), 26.7 (d, J = 14.6 Hz), 23.4, 21.4, 16.6 (d, J = 5.8 Hz), 16.5 (d, J = 6.3 Hz). MS (ESI$^+$) m/z 448.20 [M+H]$^+$.

***N*-(4-Aminophenyl)-4,5-dichloroisothiazole-3-carboxamide (95).**

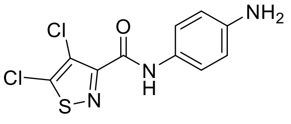

Compound **95** was synthesized according to general procedure A-1, using 4,5-dichloro-isothiazole-3-carboxylic acid (570 mg, 2.88 mmol), *N*-Boc-*p*-phenylendiamine (500 mg, 2.40 mmol) and EDC·HCl (552 mg, 2.88 mmol) in DCM (30 mL). The reaction was stirred at r.t. over night. After the workup, the product was obtained as an off white solid (712 mg, 76%). In the next step Boc-protected aniline (301 mg, 0.77 mmol) was treated with TFA (0.7 mL) in DCM (3 mL) to afford the product as a bown-red solid (112 mg, 50%; 38% over 2 steps). ^{1}H NMR (500 MHz, DMSO) δ 10.36 (s, 1H), 7.39 – 7.34 (m, 2H), 6.57 – 6.48 (m, 2H), 5.02 (s, 2H). ^{13}C NMR (126 MHz, DMSO) δ 159.2, 157.4, 149.2, 145.8, 128.3, 127.5, 121.7, 113.7. MS (ESI$^+$) m/z 288.0 [M+H]$^+$.

Diethyl (1-{[4-(4,5-dichloroisothiazole-3-carboxamido)phenyl]amino}-4-methyl-1-oxopentan-2-yl)phosphonate (113).

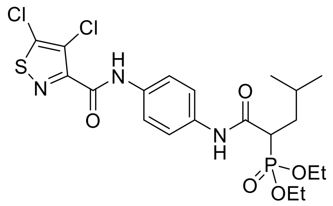

Compound **113** was synthesized according to general procedure A-2, using **214** (166 mg, 0.66 mmol), **95** (127 mg, 0.44 mmol), EDC·HCl (169 mg, 0.88 mmol), HOBt (135 mg, 0.88 mmol) and DIPEA (180 µL, 1.06 mmol) in DCM (5.5 mL). The reaction was stirred at r.t. overnight. Crude was purified using column chromatography (PB/EtOAc=1/1 to 3/7) to give diethyl (1-{[4-(4,5-dichloroisothiazole-3-carboxamido)phenyl]amino}-4-methyl-1-oxopentan-2-yl)phosphonate as a brown oil (177 mg, 77%). ^{1}H NMR (500 MHz, DMSO) δ 10.75 (s, 1H), 10.15 (s, 1H), 7.70 (d, *J*=9.0 Hz, 2H), 7.57 (d, *J*=9.1 Hz, 2H), 4.13 – 3.93 (m, 4H), 3.26 – 3.14 (m, 1H), 1.82 (d, *J*=11.4 Hz, 1H), 1.56 – 1.47 (m, 1H), 1.41 (dtdd, *J*=13.1, 9.6, 6.6, 3.1 Hz, 1H), 1.23 (ddd, *J*=12.0, 6.1, 2.6 Hz, 6H), 0.90 – 0.86 (m, 6H). ^{31}P NMR (202 MHz, DMSO) δ 24.90. MS (ESI^{+}) m/z 522.09 [M+H]$^{+}$.

***N*-(4-Aminophenyl)-5-chlorothiophene-2-carboxamide (96).**

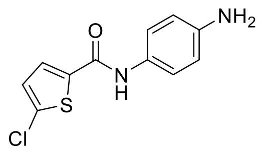

Compound **96** was synthesized according to general procedure A-1, using 5-chlorothiophene-2-carboxylic acid (468 mg, 2.88 mmol), *N*-Boc-*p*-phenylendiamine (500 mg, 2.40 mmol) and EDC·HCl (552 mg, 2.88 mmol) in DCM (30 mL). The reaction was stirred at r.t. overnight. Solid was filtered and washed with DCM to give *tert*-butyl [4-(5-chlorothiophene-2-carboxamido)phenyl]carbamate (537 mg, 63%). In the next step Boc-protected aniline (306 mg, 0.87 mmol) was treated with TFA (0.7 mL) in DCM (3 mL) to afford the product as an off-white solid (214 mg, 97%; 61% over 2 steps). ^{1}H NMR (500 MHz, DMSO) δ 9.95 (s, 1H), 7.81 (d, *J*=4.1 Hz, 1H), 7.28 (d, *J*=8.7 Hz, 2H), 7.22 (d, *J*=4.0 Hz, 1H), 6.58 – 6.49 (m, 2H), 4.98 (s, 2H). ^{13}C NMR (126 MHz, DMSO) δ 158.1, 145.6, 139.9, 133.0, 128.1, 127.0, 122.4, 113.7. MS (ESI^{+}) m/z 252.91 [M+H]$^{+}$.

Diethyl (1-{[4-(5-chlorothiophene-2-carboxamido)phenyl]amino}-4-methyl-1-oxopentan-2-yl)phosphonate (114).

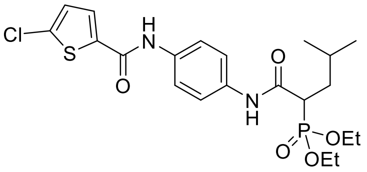

Compound **114** was synthesized according to general procedure A-2, using **214** (319 mg, 1.26 mmol), **96** (213 mg, 0.84 mmol), EDC·HCl (322 mg, 1.68 mmol), HOBt (257 mg, 1.68 mmol) and DIPEA (343 µL, 2.02 mmol) in DCM (11 mL). The reaction was stirred at r.t. overnight. After this time, full conversion could not be reached, additional EDC·HCl (161 mg, 0.84 mmol) and HOBt (129 mg, 0.84 mmol) were added. The reaction was stirred additional 3h. Crude was purified using column chromatography (PB/EtOAc=1/1 to 3/7) to give diethyl (1-{[4-(5-chlorothiophene-2-carboxamido)phenyl]amino}-4-methyl-1-oxopentan-2-yl)phosphonate as a colorless solid (342 mg, 83%). ^{1}H NMR (500 MHz, DMSO) δ 10.28 (s, 1H), 10.13 (s, 1H), 7.89 (d, *J*=4.2 Hz, 1H), 7.64 – 7.61 (m, 2H), 7.58 – 7.53 (m, 2H), 7.26 (d, *J*=4.0 Hz, 1H), 4.12 – 3.96 (m, 4H), 3.19 (ddd, *J*=22.4, 11.3, 3.1 Hz, 1H), 2.03 – 1.93 (m, 1H), 1.54 – 1.45 (m, 1H), 1.44 – 1.35 (m, 1H), 1.22 (dt, *J*=9.3, 7.0 Hz, 6H), 0.88 (dd, *J*=6.5, 2.5 Hz, 6H). ^{13}C NMR (126 MHz, DMSO) δ 166.2 (d, *J* =

4.6 Hz), 158.6, 139.3, 135.1, 133.9, 133.7, 128.9, 128.3, 121.0, 119.4, 62.0 (d, *J* = 6.2 Hz), 61.7 (d, *J* = 6.5 Hz), 44.4 (d, *J* = 130.0 Hz), 35.4 (d, *J* = 4.6 Hz), 26.3 (d, *J* = 15.1 Hz), 23.0, 21.3, 16.3 (d, *J* = 1.1 Hz), 16.3 (d, *J* = 0.9 Hz). ^{31}P NMR (202 MHz, DMSO) δ 24.93. MS (ESI^{+}) m/z 487.2 [M+H]$^{+}$.

***N*-(4-Aminophenyl)-4-chlorothiazole-2-carboxamide (97).**

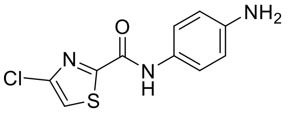

Compound **97** was synthesized according to general procedure A-1, using 2-chlorothiophene-4-carboxylic acid (471 mg, 2.88 mmol), *N*-Boc-*p*-phenylendiamine (500 mg, 2.40 mmol) and EDC·HCl (552 mg, 2.88 mmol) in DCM (30 mL). The reaction was stirred at r.t. over night. After the workup, the product was obtained as a beige solid (372 mg, 44%). In the next step Boc-protected aniline (317 mg, 1.25 mmol) was treated with TFA (0.7 mL) in DCM (3 mL) to afford the product as a light yellow solid (240 mg, 76%; 33% over 2 steps). ^{1}H NMR (500 MHz, DMSO) δ 10.24 (s, 1H), 8.38 (s, 1H), 7.69 (d, *J*=8.8 Hz, 2H), 6.96 (d, *J*=8.5 Hz, 2H). ^{13}C NMR (126 MHz, DMSO) δ 157.8, 150.9, 148.0, 128.2, 122.0, 118.9. MS (ESI^{+}) m/z 253.94 [M+H]$^{+}$.

Diethyl (1-{[4-(4-chlorothiazole-2-carboxamido)phenyl]amino}-4-methyl-1-oxopentan-2-yl)phosphonate (115).

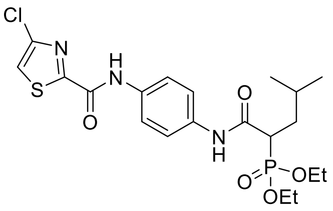

Compound **115** was synthesized according to general procedure A-3, using **97** (240 mg, 0.95 mmol), **214** (265 mg, 1.05 mmol), TBTU (337 mg, 1.05 mmol) and NMM (264 µL, 2.37 mmol) in DCM (9.5 mL). The reaction was stirred at r.t. over night. After the workup, the product was obtained as a beige solid (396 mg, 85%). ^{1}H NMR (500 MHz, DMSO) δ 10.29 (s, 1H), 10.12 (s, 1H), 8.40 (s, 1H), 7.76 – 7.72 (m, 2H), 7.55 – 7.51 (m, 2H), 4.15 – 3.97 (m, 4H), 3.19 (ddd, *J*=22.4, 11.3, 3.1, 1H), 1.97 (dddd, *J*=13.1, 11.2, 8.9, 4.5, 1H), 1.50 (dtd, *J*=9.2, 6.7, 4.6, 1H), 1.45 – 1.36 (m, 1H), 1.22 (dt, *J*=9.3, 7.0, 6H), 0.88 (dd, *J*=6.5, 2.5, 6H). ^{31}P NMR (202 MHz, DMSO) δ 24.94. MS (ESI^{+}) m/z 488.16 [M+H]$^{+}$.

***N*-(4-Aminophenyl)-4,5-dichlorothiophene-2-carboxamide (98).**

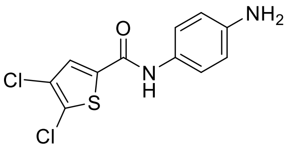

Compound **98** was synthesized according to general procedure A-1, using 4,5-dichlorothiophene-2-carboxylic acid (567 mg, 2.88 mmol), *N*-Boc-*p*-phenylendiamine (500 mg, 2.40 mmol) and EDC·HCl (552 mg, 2.88 mmol) in DCM (30 mL). The reaction was stirred at r.t. over night. Solid was filtered and washed with DCM to give *tert*-butyl [4-(4,5-dichlorothiophene-2-carboxamido)phenyl]carbamate (450 mg, 48%). In the next step Boc-protected aniline (310 mg, 0.80 mmol) was treated with TFA (0.7 mL) in DCM (3 mL) to afford the product as a bright yellow solid (216 mg, 94%; 45% over 2 steps). ^{1}H NMR (500 MHz, DMSO) δ 10.04 (s, 1H), 8.00 (s, 1H), 7.29 (d, *J*=8.7 Hz, 2H), 6.57 – 6.52 (m, 2H), 5.01 (s, 2H). ^{13}C NMR (126 MHz, DMSO) δ 157.1, 145.8, 138.1, 128.3, 127.4, 126.7, 123.2, 122.2, 113.7. MS (ESI^{+}) m/z 286.81 [M+H]$^{+}$.

Diethyl (1-{[4-(4,5-dichlorothiophene-2-carboxamido)phenyl]amino}-4-methyl-1-oxopentan-2-yl)phosphonate (116).

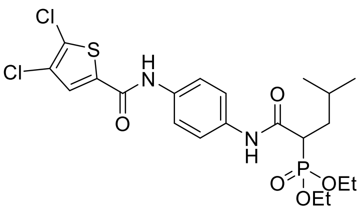

Compound **116** was synthesized according to general procedure A-3, using **98** (211 mg, 0.73 mmol), **214** (202 mg, 0.80 mmol), TBTU (257 mg, 0.80 mmol) and NMM (203 µL, 1.83 mmol) in DCM (7.3 mL). The reaction was stirred at r.t. over night. After the workup, the product was obtained as a beige solid (358 mg, 94%). ^{1}H NMR (500 MHz, DMSO) δ 10.36 (s, 1H), 10.15 (s, 1H), 8.08 (s, 1H), 7.63 (d, *J*=9.1 Hz, 2H), 7.59 – 7.55 (m, 2H), 4.14 – 3.95 (m, 4H), 3.19 (ddd, *J*=22.5, 11.2, 3.1 Hz, 1H), 1.97 (dddd, *J*=13.0, 11.2, 8.1, 4.5 Hz, 1H), 1.49 (dtd, *J*=9.1, 6.6, 4.4 Hz, 1H), 1.45 – 1.35 (m, 1H), 1.21 (dt, *J*=9.2, 7.0 Hz, 6H), 0.88 (dd, *J*=6.5, 2.5 Hz, 6H). ^{13}C NMR (126 MHz, DMSO) δ 166.2 (d, *J* = 4.7 Hz), 157.7, 137.4, 135.3, 133.6, 128.9, 128.1, 123.4, 120.9, 119.5, 62.0 (d, *J* = 6.4 Hz), 61.7 (d, *J* = 6.5 Hz), 44.4 (d, *J* = 130.2 Hz), 35.4 (d, *J* = 4.8 Hz), 26.3 (d, *J* = 15.1 Hz), 23.0, 21.2, 16.3 (d, *J* = 1.1 Hz), 16.3 (s). ^{31}P NMR (202 MHz, DMSO) δ 24.90. MS (ESI^{+}) m/z 521.16 [M+H]$^{+}$.

***N*-(4-Aminophenyl)-4,5-dichlorothiophene-2-carboxamide (99).**

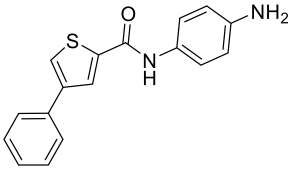

Compound **99** was synthesized according to general procedure A-1, using 4-phenylthiophene-2-carboxylic acid (588 mg, 2.88 mmol), *N*-Boc-*p*-phenylendiamine (500 mg, 2.40 mmol) and EDC·HCl (552 mg, 2.88 mmol) in DCM (30 mL). The reaction was stirred at r.t. over night. Crude was purified using column chromatography (PB/EtOAc=5/1 to 2/1) to give *tert*-butyl [4-(4-phenylthiophene-2-carboxamido)phenyl]carbamate (290 mg, 30%) as a pale red solid. In the next step Boc-protected aniline (287 mg, 0.73 mmol) was treated with TFA (0.7 mL) in DCM (3 mL) to afford the product as a yellow solid (209 mg, 98%; 29% over 2 steps). ^{1}H NMR (500 MHz, DMSO) δ 9.93 (s, 1H), 8.40 (d, *J*=1.5 Hz, 1H), 8.11 (d, *J*=1.4 Hz, 1H), 7.78 – 7.67 (m, 2H), 7.47 (t, *J*=7.8 Hz, 2H), 7.34 (dd, *J*=8.0, 5.7 Hz, 3H), 6.61 – 6.54 (m, 2H), 5.00 (s, 2H). ^{13}C NMR (126 MHz, DMSO) δ 159.0, 145.4, 141.8, 141.3, 134.7, 127.6, 127.5, 126.8, 125.9, 125.7, 122.1, 113.8. MS (ESI^{+}) m/z 295.05 [M+H]$^{+}$.

Diethyl (4-methyl-1-oxo-1-{[4-(4-phenylthiophene-2-carboxamido)phenyl]amino}pentan-2-yl)phosphonate (117).

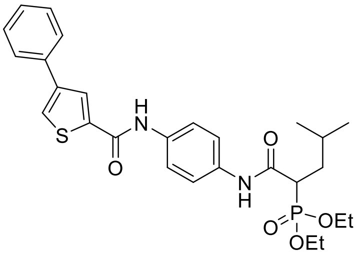

Compound **117** was synthesized according to general procedure A-3, using **99** (207 mg, 0.70 mmol), **214** (194 mg, 0.77 mmol), TBTU (247 mg, 0.77 mmol) and NMM (195 µL, 1.75 mmol) in DCM (7 mL). The reaction was stirred at r.t. for 2 h. After the workup, the product was obtained as a beige solid (370 mg, quant.). ^{1}H NMR (500 MHz, DMSO) δ 10.28 (s, 1H), 10.16 (s, 1H), 8.47 (d, *J*=1.5, 1H), 8.18 (d, *J*=1.4, 1H), 7.78 – 7.73 (m, 2H), 7.70 – 7.65 (m, 2H), 7.61 – 7.55 (m, 2H), 7.48 (t, *J*=7.8, 2H), 7.39 – 7.32 (m, 1H), 4.18 – 3.96 (m, 4H), 3.20 (ddd, *J*=22.4, 11.3, 3.1, 1H), 1.98 (d, J=10.1, 1H), 1.49 (dtt, *J*=12.7, 6.3, 3.1, 1H), 1.45 – 1.34 (m, 1H), 1.27 – 1.18 (m, 6H), 0.88 (dd, *J*=6.5, 2.9, 6H). ^{13}C NMR (126 MHz, DMSO) δ 166.2 (d, *J* = 4.7 Hz), 159.6, 141.9, 140.8, 135.0, 134.6, 134.2, 129.1, 127.7, 127.5, 126.4, 126.0, 120.8, 119.5, 62.1 (d, *J* = 6.3 Hz), 61.8 (d, *J* = 6.6 Hz), 44.5 (d, *J* = 130.3 Hz), 35.5 (d, *J*

= 4.8 Hz), 26.4 (d, *J* = 15.2 Hz), 23.1, 21.3, 16.4 (d, *J* = 1.2 Hz), 16.3 (d, *J* = 0.7 Hz). ^{31}P NMR (202 MHz, DMSO) δ 25.00. MS (ESI$^+$) m/z 529.20 [M+H]$^+$.

***N*-(4-Aminophenyl)furan-2-carboxamide (100).**

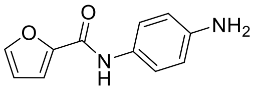

Compound **100** was synthesized according to general procedure A-1, using 2-furoic acid (323 mg, 2.88 mmol), *N*-Boc-*p*-phenylendiamine (500 mg, 2.40 mmol) and EDC·HCl (552 mg, 2.88 mmol) in DCM (30 mL). The reaction was stirred at r.t. over night. After the workup, the product was obtained as an off-white solid (396 mg, 55%). In the next step Boc-protected aniline (297 mg, 0.98 mmol) was treated with TFA (0.7 mL) in DCM (3 mL) to afford the product as a beige solid (179 mg, 90%; 50% over 2 steps). ^{1}H NMR (500 MHz, DMSO) δ 9.77 (s, 1H), 7.87 (q, *J*=0.8 Hz, 1H), 7.32 (d, *J*=8.8 Hz, 2H), 7.21 (d, *J*=3.5 Hz, 1H), 6.65 (dd, *J*=3.4, 1.8 Hz, 1H), 6.59 – 6.47 (m, 2H), 4.93 (s, 2H). ^{13}C NMR (126 MHz, DMSO) δ 155.7, 148.1, 145.4, 145.2, 127.3, 122.3, 113.7, 113.7, 112.0. MS (ESI$^+$) m/z 202.97 [M+H]$^+$.

Diethyl (1-{[4-(furan-2-carboxamido)phenyl]amino}-4-methyl-1-oxopentan-2-yl)phosphonate (118).

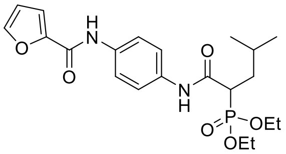

Compound **118** was synthesized according to general procedure A-3, using **100** (179 mg, 0.88 mmol), **214** (245 mg, 0.97 mmol), TBTU (311 mg, 0.97 mmol) and NMM (244 µL, 2.20 mmol) in DCM (8.8 mL). The reaction was stirred at r.t. over night After the workup, the product was obtained as a red-brown solid (385 mg, quant.). ^{1}H NMR (500 MHz, DMSO) δ 10.17 (s, 1H), 10.13 (s, 1H), 7.94 – 7.91 (m, 1H), 7.70 – 7.64 (m, 2H), 7.56 – 7.50 (m, 2H), 7.31 (dd, *J*=3.5, 0.8 Hz, 1H), 6.70 (dd, *J*=3.5, 1.7 Hz, 1H), 4.13 – 3.95 (m, 4H), 3.19 (ddd, *J*=22.5, 11.3, 3.1 Hz, 1H), 2.01 – 1.92 (m, 1H), 1.49 (dtd, *J*=13.4, 6.7, 4.5 Hz, 1H), 1.39 (ddd, *J*=12.9, 9.9, 3.1 Hz, 1H), 1.26 – 1.18 (m, 6H), 0.88 (dd, *J*=6.5, 2.4 Hz, 6H). ^{13}C NMR (126 MHz, DMSO) δ 166.2 (d, *J* = 4.7 Hz), 156.1, 147.6, 145.7, 134.9, 134.1, 120.9, 119.4, 114.6, 112.2, 62.0 (d, *J* = 6.3 Hz), 61.8 (d, *J* = 6.5 Hz), 44.4 (d, *J* = 130.1 Hz), 35.5 (d, *J* = 4.8 Hz), 26.4 (d, *J* = 15.2 Hz), 23.1, 21.3, 16.4, 16.3. ^{31}P NMR (202 MHz, DMSO) δ 25.01. MS (ESI$^+$) m/z 437.21 [M+H]$^+$.

***N*-(4-Aminophenyl)quinoline-3-carboxamide (101).**

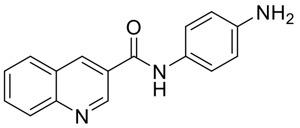

Compound **101** was synthesized according to general procedure A-1, using 3-quinoline-carboxylic acid (499 mg, 2.88 mmol), *N*-Boc-*p*-phenylendiamine (500 mg, 2.40 mmol) and EDC·HCl (552 mg, 2.88 mmol) in DCM (30 mL). The reaction was stirred at r.t. over night. Solid was filtered, washed with DCM and purified using column chromatography (PB/EtOAc=2/1 to 1/1) to give *tert*-butyl [4-(quinoline-3-carboxamido)phenyl]carbamate (444 mg, 51%).In the next step Boc-protected aniline (299 mg, 0.82 mmol) was treated with TFA (0.7 mL) in DCM (3 mL) to afford the product as a bright yellow solid (143 mg, 66%; 34% over 2 steps). ^{1}H NMR (500 MHz, DMSO) δ 10.26 (s, 1H), 9.33 (d, *J*=2.3 Hz, 1H), 8.90 (d, *J*=2.8 Hz, 1H), 8.12 (ddd, *J*=15.2, 8.4, 1.3 Hz, 2H), 7.88 (ddd, *J*=8.4, 6.8, 1.4 Hz, 1H), 7.71 (ddd, *J*=8.1, 6.9, 1.2 Hz, 1H), 7.50 – 7.35 (m, 2H), 6.65 – 6.49 (m, 2H), 5.01 (s, 2H). ^{13}C NMR (126

MHz, DMSO) δ 163.2, 149.2, 148.4, 145.6, 135.6, 131.2, 129.2, 128.8, 128.1, 127.9, 127.5, 126.6, 122.2, 113.8. MS (ESI$^+$) m/z 263.90 [M+H]$^+$.

Diethyl (4-methyl-1-oxo-1-{[4-(quinoline-3-carboxamido)phenyl]amino}pentan-2-yl)phosphonate (119).

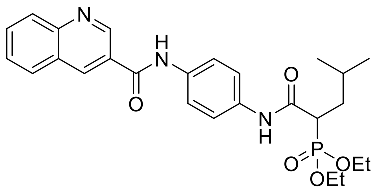

Compound **119** was synthesized according to general procedure A-3, using **101** (137 mg, 0.52 mmol), **214** (144 mg, 0.57 mmol), TBTU (183 mg, 0.57 mmol) and NMM (144 µL, 1.30 mmol) in DCM (5.2 mL). The reaction was stirred at r.t. over night. After this time, full conversion could not be reached. The reaction mixture was cooled down to 0 °C and additional **214** (66 mg, 0.26 mmol) and TBTU (83 mg, 0.26 mmol) were added. The reaction was stirred at r.t. for another night. Crude was purified using column chromatography (DCM/MeOH=100/1 to 10/1) to give diethyl (4-methyl-1-oxo-1-{[4-(quinoline-3-carboxamido)phenyl]amino}pentan-2-yl)phosphonate as a yellow solid (179 mg, 69%). ^{1}H NMR (500 MHz, DMSO) δ 10.61 (s, 1H), 10.18 (s, 1H), 9.36 (d, *J*=2.2 Hz, 1H), 8.96 (d, *J*=2.2 Hz, 1H), 8.19 – 8.09 (m, 2H), 7.90 (ddd, *J*=8.3, 6.7, 1.5 Hz, 1H), 7.78 – 7.70 (m, 3H), 7.63 – 7.56 (m, 2H), 4.13 – 3.97 (m, 4H), 3.21 (ddd, *J*=22.4, 11.3, 3.0 Hz, 1H), 2.04 – 1.93 (m, 1H), 1.50 (dtd, *J*=13.4, 6.6, 4.4 Hz, 1H), 1.40 (dtd, *J*=13.0, 9.9, 3.0 Hz, 1H), 1.22 (dt, *J*=9.6, 7.1 Hz, 6H), 0.89 (dd, *J*=6.5, 3.0 Hz, 6H). ^{13}C NMR (126 MHz, DMSO) δ 166.2 (d, *J* = 4.6 Hz), 163.9, 149.2, 148.5, 136.0, 135.1, 134.5, 131.4, 129.3, 128.9, 127.7, 127.6, 126.5, 120.9, 119.5, 62.0 (d, *J* = 6.3 Hz), 61.8 (d, *J* = 6.5 Hz), 44.5 (d, *J* = 130.2 Hz), 35.5 (d, *J* = 4.9 Hz), 26.4 (d, *J* = 15.1 Hz), 23.1, 21.3, 16.4 (d, *J* = 1.2 Hz), 16.3 (d, *J* = 0.6 Hz). ^{31}P NMR (202 MHz, DMSO) δ 25.00. MS (ESI$^+$) m/z 496.48 [M+H]$^+$.

***N*-(4-Aminophenyl)benzo[d]thiazole-2-carboxamide (102).**

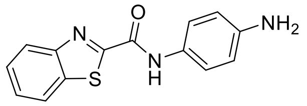

Compound **102** was synthesized according to general procedure A-1, using 1,3-benzothiazole-2-carboxylic acid (516 mg, 2.88 mmol), *N*-Boc-*p*-phenylendiamine (500 mg, 2.40 mmol) and EDC·HCl (552 mg, 2.88 mmol) in DCM (30 mL). The reaction was stirred at r.t. over night. Crude was purified using column chromatography (PB/EtOAc=4/1 to 2/1) to give *tert*-butyl [4-(benzo[d]thiazole-2-carboxamido)phenyl]carbamate (240 mg, 27%) as an off-white solid. In the next step Boc-protected aniline (236 mg, 0.64 mmol) was treated with TFA (0.55 mL) in DCM (2,5 mL) to afford the product as a yellow solid (172 mg, quant.; 27% over 2 steps). ^{1}H NMR (500 MHz, DMSO) δ 10.71 (s, 1H), 8.24 (dd, *J*=8.0, 1.3 Hz, 1H), 8.21 – 8.13 (m, 1H), 7.65 (ddd, *J*=8.2, 7.1, 1.4 Hz, 1H), 7.59 (td, *J*=7.7, 7.2, 1.3 Hz, 1H), 7.57 – 7.52 (m, 2H), 6.63 – 6.54 (m, 2H), 5.20 (s, 2H). ^{13}C NMR (126 MHz, DMSO) δ 165.4, 157.1, 152.8, 145.6, 136.3, 127.1, 127.0, 126.9, 124.0, 123.0, 122.2, 113.8. MS (ESI$^+$) m/z 270.00 [M+H]$^+$.

Diethyl (1-{[4-(benzo[d]thiazole-2-carboxamido)phenyl]amino}-4-methyl-1-oxopentan-2-yl)phosphonate (120).

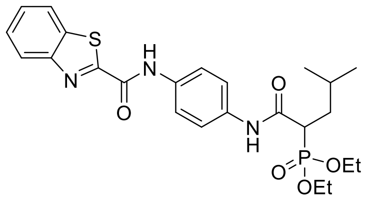

Compound **120** was synthesized according to general procedure A-3, using **102** (172 mg, 0.64 mmol), **214** (177 mg, 0.70 mmol), TBTU (225 mg, 0.70 mmol) and NMM (178 µL, 1.60 mmol) in DCM (6.4 mL). The reaction was stirred at r.t. over night. Crude was purified using column chromatography (PB/EtOAc=3/1 to 1/1) to give diethyl (1-{[4-(benzo[d]thiazole-2-carboxamido)phenyl]amino}-4-methyl-1-oxopentan-2-yl)phosphonate as a yellow solid (255 mg, 80%). ^{1}H NMR (500 MHz, DMSO) δ 11.08 (s, 1H), 10.17 (s, 1H), 8.30 – 8.25 (m, 1H), 8.24 – 8.19 (m, 1H), 7.87 – 7.83 (m, 2H), 7.70 – 7.65 (m, 1H), 7.64 – 7.56 (m, 3H), 4.05 (dddd, *J*=21.9, 11.3, 7.8, 5.5 Hz, 4H), 3.28 – 3.16 (m, 1H), 1.98 (tdd, *J*=12.8, 8.0, 4.4 Hz, 1H), 1.56 – 1.47 (m, 1H), 1.46 – 1.37 (m, 1H), 1.22 (dt, *J*=9.5, 7.1 Hz, 6H), 0.89 (dd, *J*=6.6, 2.8 Hz, 6H). ^{13}C NMR (126 MHz, DMSO) δ 166.2 (d, *J* = 4.6 Hz), 164.8, 157.9, 152.7, 136.4, 135.6, 133.4, 127.2, 127.1, 124.1, 123.1, 121.3, 119.3, 62.0 (d, *J* = 6.3 Hz), 61.8 (d, *J* = 6.5 Hz), 44.5 (d, *J* = 130.1 Hz), 35.4 (d, *J* = 4.6 Hz), 26.4 (d, *J* = 15.1 Hz), 23.0, 21.3, 16.3, 16.3. ^{31}P NMR (202 MHz, DMSO) δ 24.90. MS (ESI^{+}) m/z 504.23 [M+H]$^{+}$.

***N*-(4-Aminophenyl)-5-phenylthiophene-2-carboxamide (103).**

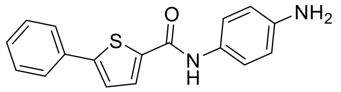

Compound **103** was synthesized according to general procedure A-3, using 5-phenylthiophene-2-carboxylic acid (434 mg, 2.12 mmol), N-Boc-p-phenylene-diamine (400 mg, 1.92 mmol), TBTU (681 mg, 2.12 mmol) and NMM (442 µL, 4.80 mmol) in DCM (19.2 mL). The reaction was stirred at r.t. over night. Solid was filtered and washed with DCM to give *tert*-butyl [4-(5-phenylthiophene-2-carboxamido)phenyl]carbamate (694 mg, 91%). In the next step Boc-protected aniline (296 mg, 0.75 mmol) was treated with TFA (0.7 mL) in DCM (3 mL). Solid was filtered and washed with DCM to afford the product as a colorless solid (115 mg, 37%; 34% over 2 steps). ^{1}H NMR (500 MHz, DMSO) δ 10.33 (s, 1H), 8.02 (d, *J*=3.9 Hz, 1H), 7.75 (td, *J*=6.4, 1.8 Hz, 4H), 7.63 (d, *J*=3.9 Hz, 1H), 7.50 – 7.44 (m, 2H), 7.42 – 7.36 (m, 1H), 7.23 – 7.15 (m, 2H). ^{13}C NMR (126 MHz, DMSO) δ 159.7, 158.3, 158.1, 148.6, 138.7, 133.0, 130.4, 129.4, 128.8, 125.8, 124.6, 121.7, 121.6. MS (ESI^{+}) m/z 295.05 [M+H]$^{+}$.

Diethyl (4-methyl-1-oxo-1-{[4-(5-phenylthiophene-2-carboxamido)phenyl]amino}pentan-2-yl)phosphonate (121).

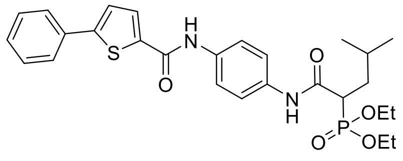

Compound **121** was synthesized according to general procedure A-3, using **103** (115 mg, 0.28 mmol), **214** (78 mg, 0.31 mmol), TBTU (99 mg, 0.31 mmol) and NMM (155 µL, 1.40 mmol) in DCM (4 mL). The reaction was stirred at r.t. over night. Crude was purified using column chromatography (PB/EtOAc=2/1 to 1/1) to give diethyl (4-methyl-1-oxo-1-{[4-(5-phenylthiophene-2-carboxamido)phenyl]amino}pentan-2-yl)phosphonate as a pale yellow solid (124 mg, 82%). ^{1}H NMR (500 MHz, DMSO) δ 10.23 (s, 1H), 10.12 (s, 1H), 8.01 (d, *J*=4.0, 1H), 7.77 – 7.72 (m, 2H), 7.70 – 7.65 (m, 2H), 7.62 (d, *J*=3.9, 1H), 7.58 – 7.54 (m, 2H), 7.47 (dd, *J*=8.4, 7.0, 2H), 7.42 – 7.36 (m, 1H), 4.14 – 3.93 (m, 4H), 3.20 (ddd, *J*=22.4, 11.2, 3.1, 1H), 1.98 (dddd, *J*=12.9, 11.1, 8.1, 4.5, 1H), 1.50 (ddd, *J*=11.2, 8.2, 5.6, 1H), 1.40 (dtd, *J*=13.1, 10.0, 3.0, 1H), 1.22 (dt, *J*=9.3, 7.0, 6H),

0.89 (dd, J=6.6, 2.7, 6H). ^{13}C NMR (126 MHz, DMSO) δ 166.1 (d, J = 4.6 Hz), 159.4, 148.2, 139.0, 134.9, 134.2, 133.0, 130.1, 129.3, 128.7, 125.7, 124.4, 120.9, 119.4, 62.0 (d, J = 6.4 Hz), 61.8 (d, J = 6.5 Hz), 44.4 (d, J = 130.2 Hz), 35.4 (d, J = 4.4 Hz), 26.3 (d, J = 15.0 Hz), 23.0, 21.3, 16.3 (d, J = 0.8 Hz), 16.3. ^{31}P NMR (202 MHz, DMSO) δ 24.96. MS (ESI$^+$) m/z 529.23 [M+H]$^+$.

***N*-(4-Aminophenyl)benzenesulfonamide (104).**

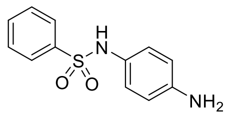

Compound **104** was synthesized over two steps according to general procedure B, using benzenesulfonyl chloride (200 µL, 1.58 mmol), *N*-Boc-*p*-phenylendiamine (300 mg, 1.44 mmol) and Et_3N (240 µL, 1.73 mmol) in DCM (10 mL). The reaction was stirred at r.t. overnight. Crude was purified using column chromatography (Hex/EtOAc=6/4) to give *tert*-butyl [4-(phenylsulfonamido)phenyl]carbamate (312 mg, 62%). In the next step Boc-protected aniline (300 mg, 0.86 mmol) was treated with TFA (0.8 mL) in DCM (3 mL) to afford the product as a beige solid (154 mg, 72%; 45% over 2 steps). ^{1}H NMR (500 MHz, DMSO) δ 9.46 (s, 1H), 7.66 – 7.61 (m, 2H), 7.61 – 7.55 (m, 1H), 7.50 (dd, J = 10.3, 4.6 Hz, 2H), 6.71 – 6.59 (m, 2H), 6.42 – 6.31 (m, 2H), 4.95 (s, 2H). ^{13}C NMR (126 MHz, DMSO) δ 146.6, 139.7, 132.4, 128.9, 126.7, 125.2, 124.7, 114.0. MS (ESI$^+$) m/z 248.97 [M+H]$^+$.

Diethyl (4-methyl-1-oxo-1-{[4-(phenylsulfonamido)phenyl]amino}pentan-2-yl)phosphonate (122).

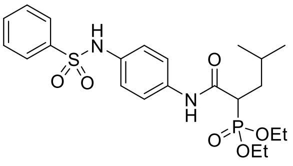

Compound **122** was synthesized according to general procedure A-3, using **104** (140 mg, 0.56 mmol), **214** (171 mg, 0.68 mmol), TBTU (272 mg, 0.85 mmol) and NMM (155 µL, 1.41 mmol) in DMF (5 mL). Crude was purified using column chromatography (Hex/EtOAc=2/8) to give the compound **122** as a white foam (169 mg, 62%). ^{1}H NMR (500 MHz, $CDCl_3$) δ 8.96 (s, 1H), 7.77 – 7.70 (m, 2H), 7.52 (dd, J = 10.6, 4.3 Hz, 1H), 7.41 (t, J = 7.8 Hz, 2H), 7.31 – 7.26 (m, 3H), 6.83 (d, J = 8.7 Hz, 2H), 4.27 – 4.08 (m, 4H), 3.11 (ddd, J = 22.8, 11.2, 3.2 Hz, 1H), 2.15 – 2.02 (m, 1H), 1.71 – 1.63 (m, 1H), 1.51 (dtd, J = 20.6, 10.4, 7.2 Hz, 1H), 1.38 – 1.30 (m, 6H), 0.92 (dt, J = 18.0, 9.0 Hz, 6H). ^{13}C NMR (126 MHz, $CDCl_3$) δ 166.0 (d, J = 2.7 Hz), 139.5, 136.1, 133.0, 132.1, 129.1, 127.3, 123.5, 120.4, 63.8 (d, J = 6.5 Hz), 62.7 (d, J = 6.8 Hz), 45.4 (d, J = 129.0 Hz), 35.9 (d, J = 5.1 Hz), 26.7 (d, J = 14.4 Hz), 23.4, 21.4, 16.5 (t, J = 6.4 Hz). MS (ESI$^+$) m/z 483.22 [M+H]$^+$.

***N*-(4-Aminophenyl)-3,4-dichlorobenzenesulfonamide (105).**

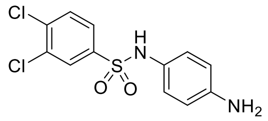

Compound **105** was synthesized over two steps according to general procedure B, using 3,4-dichlorobenzenesulfonyl chloride (250 µL, 1.58 mmol), *N*-Boc-*p*-phenylendiamine (300 mg, 1.44 mmol) and Et_3N (240 µL, 1.73 mmol) in DCM (10 mL). The reaction was stirred at r.t. overnight. Crude was purified using column chromatography (Hex/EtOAc=7/3) to give *tert*-butyl {4-[(3,4-dichlorophenyl)sulfonamido]phenyl}carbamate (348 mg, 58%). In the next step Boc-protected aniline (348 mg, 0.83 mmol) was treated with TFA (0.8 mL) in DCM (3 mL) to afford the product as a beige solid (116 mg, 44%; 25% over 2 steps). ^{1}H NMR (500 MHz, DMSO) δ 9.65 (s, 1H), 7.81 (d, J = 8.4 Hz, 1H), 7.77 (d, J = 2.1 Hz, 1H), 7.54 (dd, J = 8.4, 2.1 Hz, 1H), 6.70 – 6.62 (m, 2H), 6.43 – 6.37 (m, 2H),

5.01 (s, 2H). ^{13}C NMR (126 MHz, DMSO) δ 146.9, 140.3, 135.4, 131.8, 131.4, 128.4, 126.9, 125.1, 124.9, 114.1. MS (ESI$^-$) m/z 314.99 [M-H]$^-$.

Diethyl [1-({4-[(3,4-dichlorophenyl)sulfonamido]phenyl}amino)-4-methyl-1-oxopentan-2-yl)phosphonate (123).

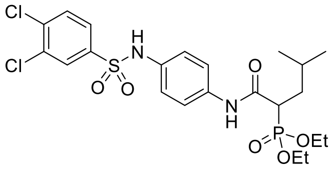

Compound **123** was synthesized according to general procedure A-2, using **105** (84 mg, 0.26 mmol), **214** (100 mg, 0.40 mmol), EDC·HCl (100 mg, 0.52 mmol), HOBt (80 mg, 0.52 mmol) and DIPEA (110 µL, 0.62 mmol) in DCM (5 mL). Crude was purified using column chromatography (Hex/EtOAc=7/3) to give the compound **123** as a white foam (84 mg, 58%). ^{1}H NMR (500 MHz, DMSO) δ 10.27 (s, 1H), 10.11 (s, 1H), 7.88 (d, *J* = 2.1 Hz, 1H), 7.83 (d, *J* = 8.4 Hz, 1H), 7.62 (dd, *J* = 8.5, 2.1 Hz, 1H), 7.49 – 7.44 (m, 2H), 7.05 – 6.99 (m, 2H), 4.09 – 3.91 (m, 4H), 3.15 (ddd, *J* = 22.5, 11.2, 3.0 Hz, 1H), 1.99 – 1.88 (m, 1H), 1.40 (dddd, *J* = 16.8, 13.4, 7.1, 4.6 Hz, 2H), 1.18 (dt, *J* = 9.0, 7.1 Hz, 6H), 0.85 (d, *J* = 6.5 Hz, 6H). ^{13}C NMR (126 MHz, DMSO) δ 166.3 (d, *J* = 4.5 Hz), 139.7, 136.3, 136.0, 132.1, 132.1, 131.7, 128.4, 126.8, 122.3, 119.9, 62.0 (d, *J* = 6.3 Hz), 61.8 (d, *J* = 6.5 Hz), 44.4 (d, *J* = 130.1 Hz), 35.4 (d, *J* = 4.8 Hz), 26.3 (d, *J* = 15.2 Hz), 23.0, 21.2, 16.3, 16.2 (d, *J* = 1.5 Hz). MS (ESI$^+$) m/z 551.12 [M+H]$^+$.

***N*-(4-Aminophenyl)-3,4-dimethoxybenzenesulfonamide (106).**

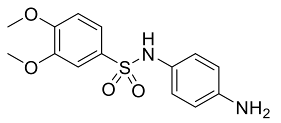

Compound **106** was synthesized over two steps according to general procedure B, using 3,4-dimethoxybenzenesulfonyl chloride (375 mg, 1.58 mmol), *N*-Boc-*p*-phenylendiamine (300 mg, 1.44 mmol) and Et_3N (240 µL, 1.73 mmol) in DCM (10 mL). The reaction was stirred at r.t. overnight. Crude was purified using column chromatography (Hex/EtOAc=6/4) to give *tert*-butyl {4-[(3,4-dimethoxyphenyl)sulfonamido]phenyl}carbamate (470 mg, 80%). In the next step Boc-protected aniline (460 mg, 1.13 mmol) was treated with TFA (1.2 mL) in DCM (4 mL) to afford the product as a beige solid (243 mg, 70%; 56% over 2 steps). ^{1}H NMR (500 MHz, DMSO) δ 11.56 (s, 1H), 9.50 – 9.41 (m, 2H), 9.30 (d, *J* = 8.5 Hz, 1H), 9.02 – 8.90 (m, 2H), 8.72 – 8.63 (m, 2H), 7.23 (s, 2H), 6.07 (s, 3H), 6.01 (s, 3H). ^{13}C NMR (126 MHz, DMSO) δ 151.8, 148.3, 146.5, 131.2, 125.6, 124.7, 120.5, 113.9, 110.8, 109.5, 55.7, 55.6. MS (ESI$^+$) m/z 309.09 [M+H]$^+$.

Diethyl [1-({4-[(3,4-dimethoxyphenyl)sulfonamido]phenyl}amino)-4-methyl-1-oxopentan-2-yl]phosphonate (124).

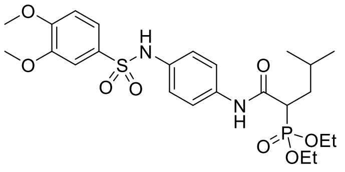

Compound **124** was synthesized according to general procedure A-3, using **106** (150 mg, 0.49 mmol), **214** (147 mg, 0.58 mmol), TBTU (236 mg, 0.74 mmol) and NMM (135 µL, 1.22 mmol) in DMF (5 mL). Crude was purified using column chromatography (EtOAc to EtOAc/MeOH=95/5) to give the compound **124** as a transparent oil (200 mg, 75%). ^{1}H NMR (500 MHz, $CDCl_3$) δ 8.99 (s, 1H), 8.02 (s, 1H), 7.35 – 7.29 (m, 3H), 7.19 (d, *J* = 2.1 Hz, 1H), 6.88 (d, *J* = 8.8 Hz, 2H), 6.82 (dd, *J* = 8.4, 5.0 Hz, 1H), 4.25 – 4.08 (m, 4H), 3.89 (s, 3H), 3.79 (s, 3H), 3.14 – 3.05 (m, 1H),

2.11 – 2.03 (m, 1H), 1.65 – 1.60 (m, 1H), 1.50 (tdd, *J* = 9.9, 8.5, 3.1 Hz, 1H), 1.33 (dt, *J* = 10.1, 7.1 Hz, 6H), 0.92 (dd, *J* = 9.5, 6.6 Hz, 6H). ^{13}C NMR (126 MHz, $CDCl_3$) δ 166.0 (d, *J* = 2.7 Hz), 152.8, 149.0, 136.0, 132.5, 131.0, 123.4, 121.4, 120.4, 110.6, 109.8, 63.7 (d, *J* = 6.6 Hz), 62.6 (d, *J* = 6.7 Hz), 56.3, 56.2, 45.4 (d, *J* = 128.9 Hz), 35.9 (d, *J* = 5.0 Hz), 26.7 (d, *J* = 14.2 Hz), 23.3, 21.4, 16.6 – 16.4 (m). MS (ESI^+) m/z 543.2 $[M+H]^+$.

***N*-(4-Aminophenyl)thiophene-2-sulfonamide (107).**

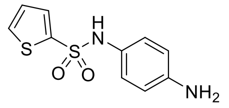

Compound **107** was synthesized over two steps according to general procedure B, using thiophene-2-sulfonyl chloride (386 mg, 2.11 mmol), *N*-Boc-*p*-phenylendiamine (400 mg, 1.92 mmol) and Et_3N (320 µL, 2.30 mmol) in DCM (6 mL). The reaction was stirred at r.t. overnight. Crude was purified using column chromatography (Hex/EtOAc=7/3) to give *tert*-butyl [4-(thiophene-2-sulfonamido)phenyl]carbamate (628 mg, 92%). In the next step Boc-protected aniline (339 mg, 0.96 mmol) was treated with TFA (1.0 mL) in DCM (4 mL) to afford the product as a beige solid (201 mg, 83%; 76% over 2 steps). ^{1}H NMR (500 MHz, DMSO) δ 9.61 (s, 1H), 7.85 (dd, *J* = 5.0, 1.3 Hz, 1H), 7.35 (dd, *J* = 3.7, 1.3 Hz, 1H), 7.09 (dd, *J* = 5.0, 3.7 Hz, 1H), 6.75 – 6.66 (m, 2H), 6.46 – 6.37 (m, 2H), 5.03 (s, 2H). ^{13}C NMR (126 MHz, DMSO) δ 147.0, 140.2, 132.8, 132.0, 127.5, 125.0, 125.0, 114.0 MS (ESI^+) m/z 255.12 $[M+H]^+$.

Diethyl (4-methyl-1-oxo-1-{[4-(thiophene-2-sulfonamido)phenyl]amino}pentan-2-yl)phosphonate (125).

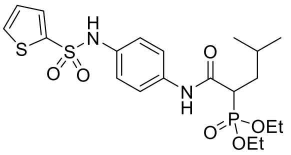

Compound **125** was synthesized according to general procedure A-3, using **107** (148.7 mg, 0.58 mmol), **214** (177 mg, 0.70 mmol), TBTU (279 mg, 0.87 mmol) and NMM (125 µL, 1.16 mmol) in DMF (4 mL). Crude was purified using column chromatography (Hex/EtOAc=2/8) to give the compound **125** as a pale yellow oil (194 mg, 68%). ^{1}H NMR (500 MHz, DMSO) δ 10.26 (s, 1H), 10.12 (s, 1H), 7.87 (dd, *J* = 5.0, 1.3 Hz, 1H), 7.49 – 7.45 (m, 3H), 7.10 (dd, *J* = 5.0, 3.8 Hz, 1H), 7.07 – 7.01 (m, 2H), 4.08 – 3.94 (m, 4H), 3.16 (ddd, *J* = 22.4, 11.3, 2.9 Hz, 1H), 1.97 – 1.88 (m, 1H), 1.51 – 1.30 (m, 2H), 1.26 – 1.12 (m, 6H), 0.85 (d, *J* = 6.5 Hz, 6H). ^{13}C NMR (126 MHz, DMSO) δ 166.3 (d, *J* = 4.6 Hz), 139.8, 136.1, 133.3, 132.6, 132.4, 127.7, 122.0, 119.8, 62.1 (d, *J* = 6.3 Hz), 61.8 (d, *J* = 6.5 Hz), 44.4 (d, *J* = 129.9 Hz), 35.5 (d, *J* = 4.9 Hz), 26.3 (d, *J* = 15.1 Hz), 23.1, 21.2, 16.4 (d, *J* = 1.6 Hz), 16.3 (d, *J* = 1.5 Hz). MS (ESI^+) m/z 489.09 $[M+H]^+$.

***N*-(4-Aminophenyl)naphthalene-2-sulfonamide (108).**

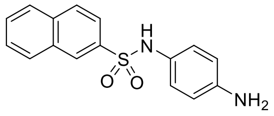

Compound **108** was synthesized over two steps according to general procedure B, using naphthalene-2-sulfonyl chloride (369 mg, 1.63 mmol), *N*-Boc-*p*-phenylendiamine (309 mg, 1.48 mmol) and Et_3N (250 µL, 1.78 mmol) in DCM (7 mL). The reaction was stirred at r.t. for 4 h. Crude was purified using column chromatography (Hex/EtOAc=7/3) to give *tert*-butyl [4-(naphthalene-2-sulfonamido)phenyl]carbamate (368 mg, 62%). In the next step Boc-protected aniline (338 mg, 0.85 mmol) was treated with TFA (1.0 mL) in DCM (4 mL) to afford the product as a beige solid (252 mg, 99%; 61% over 2 steps). ^{1}H NMR

(500 MHz, DMSO) δ 9.58 (s, 1H), 8.25 (d, J = 1.4 Hz, 1H), 8.09 – 8.04 (m, J = 8.3, 3.2 Hz, 2H), 8.00 (d, J = 8.1 Hz, 1H), 7.73 – 7.65 (m, 2H), 7.65 – 7.60 (m, 1H), 6.71 – 6.64 (m, 2H), 6.38 – 6.31 (m, 2H), 4.92 (s, 2H). ^{13}C NMR (126 MHz, DMSO) δ 146.7, 136.9, 134.1, 131.6, 129.2, 129.1, 128.8, 127.9, 127.8, 127.6, 125.2, 124.9, 122.5, 114.0. MS (ESI$^+$) m/z 298.88 [M+H]$^+$.

Diethyl (4-methyl-1-{[4-(naphthalene-2-sulfonamido)phenyl]amino}-1-oxopentan-2-yl)phosphonate (126).

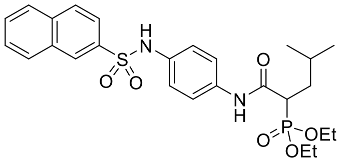

Compound **126** was synthesized according to general procedure A-3, using **108** (120 mg, 0.40 mmol), **214** (122 mg, 0.48 mmol), TBTU (193 mg, 0.60 mmol) and NMM (110 µL, 1.00 mmol) in DMF (4 mL). Crude was purified using column chromatography (Hex/EtOAc=2/8) to give the compound **126** as a white solid (113 mg, 53%). ^{1}H NMR (500 MHz, DMSO) δ 10.24 (s, 1H), 10.04 (s, 1H), 8.38 (d, J = 1.5 Hz, 1H), 8.09 (dd, J = 16.3, 8.4 Hz, 2H), 7.99 (d, J = 8.1 Hz, 1H), 7.73 (dd, J = 8.7, 1.9 Hz, 1H), 7.70 – 7.66 (m, 1H), 7.65 – 7.61 (m, 1H), 7.42 – 7.37 (m, 2H), 7.06 – 7.01 (m, 2H), 4.10 – 3.88 (m, 4H), 3.11 (ddd, J = 22.4, 11.3, 2.9 Hz, 1H), 1.96 – 1.83 (m, 1H), 1.46 – 1.27 (m, 2H), 1.15 (dt, J = 10.1, 7.1 Hz, 6H), 0.81 (d, J = 6.4 Hz, 6H). ^{13}C NMR (126 MHz, DMSO) δ 166.2 (d, J = 4.7 Hz), 136.5, 135.7, 134.3, 132.8, 131.6, 129.5, 129.3, 129.0, 127.9, 127.9, 127.7, 122.1, 121.7, 119.8, 62.0 (d, J = 6.3 Hz), 61.7 (d, J = 6.5 Hz), 44.4 (d, J = 130.0 Hz), 35.4 (d, J = 5.0 Hz), 26.3 (d, J = 15.0 Hz), 23.1, 21.2, 16.3 (d, J = 1.8 Hz), 16.3 (d, J = 1.6 Hz). MS (ESI$^+$) m/z 533.22 [M+H]$^+$.

***N*-(4-Aminophenyl)cyclohexanesulfonamide (109).**

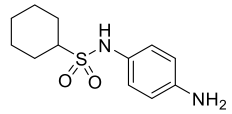

Compound **109** was synthesized over two steps according to general procedure B, using cyclohexanesulfonyl chloride (803 mg, 4.40 mmol), *N*-Boc-*p*-phenylendiamine (800 mg, 3.84 mmol) and Et_3N (640 µL, 4.61 mmol) in DCM (12 mL). The reaction was stirred at r.t. for 2 h. Crude was purified using column chromatography (Hex/EtOAc=7/3) to give *tert*-butyl [4-(cyclohexanesulfonamido)phenyl]carbamate (265 mg, 16%). In the next step Boc-protected aniline (260 mg, 0.73 mmol) was treated with TFA (0.7 mL) in DCM (3 mL) to afford the product as a pale brown solid (160 mg, 86%; 14% over 2 steps). ^{1}H NMR (500 MHz, H_2O) δ 9.05 (s, 1H), 6.94 – 6.82 (m, 2H), 6.54 – 6.39 (m, 2H), 4.99 (s, 2H), 2.74 (tt, J = 11.9, 3.3 Hz, 1H), 2.00 (d, J = 10.5 Hz, 2H), 1.74 (d, J = 12.8 Hz, 2H), 1.57 (d, J = 11.5 Hz, 1H), 1.40 – 1.28 (m, 2H), 1.14 (dq, J = 24.7, 12.4 Hz, 3H). ^{13}C NMR (126 MHz, H_2O) δ 146.2, 126.2, 123.9, 114.3, 57.7, 26.1, 24.9, 24.5. MS (ESI$^+$) m/z 255.04 [M+H]$^+$.

Diethyl {1-[(4-(cyclohexanesulfonamido)phenyl]amino}-4-methyl-1-oxopentan-2-yl)phosphonate (127).

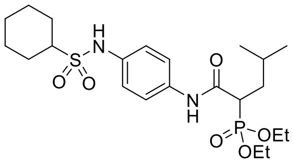

Compound **127** was synthesized according to general procedure A-3, using **109** (143 mg, 0.56 mmol), **214** (170.5 mg, 0.68 mmol), TBTU (270 mg, 0.84 mmol) and NMM (125 µL, 1.12 mmol) in DMF (4 mL). Crude was purified using column chromatography (Hex/EtOAc=2/8 to EtOAc) to give the compound **127** as a white solid (131.3 mg, 48%). ^{1}H NMR (500 MHz, DMSO) δ 10.13 (s, 1H), 9.65 (s,

1H), 7.56 – 7.46 (m, 2H), 7.20 – 7.10 (m, 2H), 4.11 – 3.95 (m, 4H), 3.17 (ddd, *J* = 22.4, 11.3, 2.9 Hz, 1H), 2.90 (tt, *J* = 11.9, 3.3 Hz, 1H), 1.96 (ddd, *J* = 13.1, 11.4, 7.0 Hz, 3H), 1.74 (d, *J* = 13.0 Hz, 2H), 1.56 (d, *J* = 12.2 Hz, 1H), 1.41 (dddd, *J* = 19.8, 14.2, 8.8, 4.9 Hz, 4H), 1.25 – 1.05 (m, 9H), 0.87 (d, *J* = 6.5 Hz, 6H). ^{13}C NMR (126 MHz, DMSO) δ 166.2 (d, *J* = 4.7 Hz), 135.1, 133.9, 120.6, 120.1, 62.1 (d, *J* = 6.2 Hz), 61.8 (d, *J* = 6.5 Hz), 58.6, 44.4 (d, *J* = 129.9 Hz), 35.5 (d, *J* = 4.9 Hz), 26.4 (d, *J* = 15.1 Hz), 26.0, 24.8, 24.4, 23.1, 21.2, 16.4 (d, *J* = 1.7 Hz), 16.3 (d, *J* = 1.6 Hz). MS (ESI$^+$) m/z 489.24 [M+H]$^+$.

4-{[4-(2-Oxopyrrolidin-1-yl)phenyl]sulfonamido}benzenaminium chloride (110).

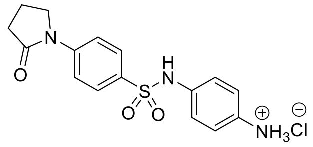

Compound **110** was synthesized over two steps according to general procedure B, using 4-(2-oxopyrrolidin-1-yl)benzenesulfonyl chloride (410 mg, 1.58 mmol), *N*-Boc-*p*-phenylendiamine (300 mg, 1.44 mmol) and Et_3N (240 µL, 1.73 mmol) in DCM (5 mL). The reaction was stirred at r.t. overnight. Crude was purified using column chromatography (EtOAc to EtOAc/MeOH=9/1) to give *tert*-butyl (4-{[4-(2-oxopyrrolidin-1-yl)phenyl]sulfonamido}phenyl)carbamate (204 mg, 33%). In the next step Boc-protected aniline (200 mg, 0.46 mmol) was treated with 4 M HCl in dioxane (1.84 mL) in DCM/MeOH (2 mL/2 mL) to afford the product as a beige solid (170 mg, 99%; 33% over 2 steps). MS (ESI$^+$) m/z 332.15 [M+H]$^+$.

Diethyl {4-methyl-1-oxo-1-[(4-{[4-(2-oxopyrrolidin-1-yl)phenyl]sulfonamido}phenyl)amino]pentan-2-yl}phosphonate (128).

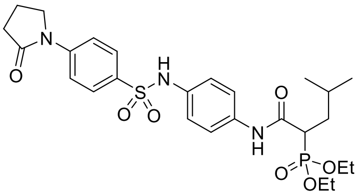

Compound **128** was synthesized according to general procedure A-3, using **110** (160 mg, 0.43 mmol), **214** (132 mg, 0.52 mmol), TBTU (207 mg, 0.64 mmol) and NMM (120 µL, 1.08 mmol) in DMF (4 mL). Crude was purified using column chromatography (EtOAc/MeOH=9/1) to give the compound **128** as a white solid (120.7 mg, 50%). ^{1}H NMR (500 MHz, DMSO) δ 10.04 (s, 2H), 7.85 – 7.78 (m, 2H), 7.74 – 7.67 (m, 2H), 7.45 – 7.37 (m, 2H), 7.05 – 6.96 (m, 2H), 4.07 – 3.93 (m, 4H), 3.81 (t, *J* = 7.0 Hz, 2H), 3.13 (ddd, *J* = 22.4, 11.2, 2.9 Hz, 1H), 2.52 – 2.48 (m, 2H), 2.08 – 1.98 (m, 2H), 1.92 (tdd, *J* = 15.6, 8.1, 4.3 Hz, 1H), 1.54 – 1.30 (m, 2H), 1.19 (q, *J* = 7.2 Hz, 6H), 0.84 (d, *J* = 6.5 Hz, 6H). ^{13}C NMR (126 MHz, DMSO) δ 174.7, 166.2 (d, *J* = 4.6 Hz), 143.1, 135.5, 133.5, 133.0, 127.6, 121.4, 119.8, 118.6, 62.0 (d, *J* = 6.1 Hz), 61.7 (d, *J* = 6.5 Hz), 47.9, 44.4 (d, *J* = 130.0 Hz), 35.4 (d, *J* = 5.0 Hz), 32.4, 26.3 (d, *J* = 14.9 Hz), 23.0, 21.2, 17.3, 16.3 (d, *J* = 4.1 Hz). MS (ESI$^+$) m/z 566.27 [M+H]$^+$.

***N*-(4-Aminophenyl)-2-(3,4-dichlorophenyl)acetamide (177).**

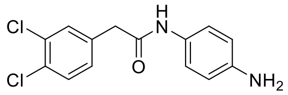

Compound **177** was synthesized according to general procedure A-1, using 2-(3,4-dichlorophenyl)acetic acid (591 mg, 2.88 mmol), *N*-Boc-*p*-phenylendiamine (500 mg, 2.40 mmol) and EDC·HCl (552 mg, 2.88 mmol) in DCM (30 mL). The reaction was stirred at r.t. for 1 h. Solid was filtered and washed with DCM. Deprotection of Boc using TFA (1 mL, 14.32 mmol) in DCM (20 mL) afforded the product as a white solid (404 mg, 66%). ^{1}H NMR (500 MHz, DMSO) δ 9.75 (s, 1H), 7.63 – 7.53 (m, 2H), 7.30 (dd, *J* =

8.3, 2.0 Hz, 1H), 7.24 – 7.16 (m, 2H), 6.54 – 6.44 (m, 2H), 4.86 (s, 2H), 3.58 (s, 2H). ^{13}C NMR (126 MHz, DMSO) δ 167.1, 144.9, 137.5, 131.2, 130.7, 130.3, 129.6, 129.1, 128.1, 120.9, 113.8, 41.8. MS (ESI^{+}) m/z 294.94 [M+H]$^{+}$.

2-Chloro-*N*-{4-[2-(3,4-dichlorophenyl)acetamido]phenyl}-4-methylpentanamide (186).

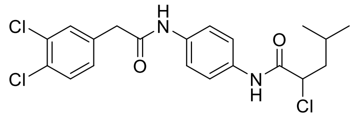

Compound **186** was synthesized according to general procedure A-1, using 2-chloro-4-methylpentanoic acid (145 mg, 0.96 mmol), **177** (221 mg, 0.75 mmol) and EDC·HCl (185 mg, 0.96 mmol) in DCM (20 mL). The reaction was stirred at r.t. for 30 min. After the workup, the product was obtained as a beige solid (320 mg, 99%). ^{1}H NMR (500 MHz, DMSO) δ 10.33 (s, 1H), 10.22 (s, 1H), 7.62 – 7.57 (m, 2H), 7.56 – 7.49 (m, 4H), 7.31 (dd, *J* = 8.3, 2.0 Hz, 1H), 4.57 (dd, *J* = 8.0, 6.9 Hz, 1H), 3.67 (s, 2H), 1.89 – 1.71 (m, 3H), 0.94 – 0.87 (m, 6H). ^{13}C NMR (126 MHz, DMSO) δ 168.1, 166.7, 137.1, 135.2, 133.9, 131.4, 130.8, 130.4, 129.8, 129.3, 119.9, 119.7, 57.8, 42.8, 41.9, 25.0, 22.4, 21.7. MS (ESI^{+}) m/z 426.85 [M+H]$^{+}$.

***N*-(4-Aminophenyl)-2-(thiophen-2-yl)acetamide (179).**

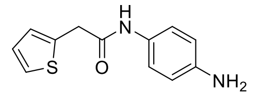

Compound **179** was synthesized according to general procedure A-1, using 2-(thiophen-2-yl)acetic acid (500 mg, 3.52 mmol), *N*-Boc-*p*-phenylendiamine (611 mg, 2.93 mmol) and EDC·HCl (674 mg, 3.52 mmol) in DCM (30 mL). The reaction was stirred at r.t. for 1 h. Solid was filtered and washed with DCM to give *tert*-butyl {4-[2-(thiophen-2-yl)acetamido]phenyl}carbamate (356 mg, 36%). In the next step Boc-protected aniline (356 mg, 1.07 mmol) was treated with TFA (0.5 mL) in DCM (15 mL) to afford the product as a beige solid (236 mg, 95%; 34% over 2 steps). ^{1}H NMR (500 MHz, DMSO) δ 9.76 (s, 1H), 7.36 (dd, *J* = 4.8, 1.6 Hz, 1H), 7.27 – 7.17 (m, 2H), 6.96 (dd, *J* = 4.7, 3.5 Hz, 2H), 6.56 – 6.42 (m, 2H), 4.86 (s, 2H), 3.77 (s, 2H). ^{13}C NMR (126 MHz, DMSO) δ 167.0, 144.9, 137.7, 128.2, 126.6, 126.1, 124.9, 120.9, 113.8, 37.4. MS (ESI^{+}) m/z 232.98 [M+H]$^{+}$.

2-Chloro-4-methyl-*N*-{4-[2-(thiophen-2-yl)acetamido]phenyl}pentanamide (188).

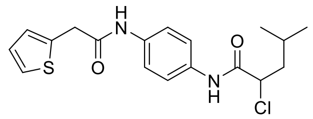

Compound **188** was synthesized according to general procedure A-1, using 2-chloro-4-methylpentanoic acid (122 mg, 0.81 mmol), **179** (157 mg, 0.67 mmol) and EDC·HCl (155 mg, 0.81 mmol) in DCM (20 mL). The reaction was stirred at r.t. for 1 h. After the workup, the product was obtained as a beige solid (170 mg, 69%). ^{1}H NMR (500 MHz, DMSO) δ 10.34 (s, 1H), 10.22 (s, 1H), 7.59 – 7.49 (m, 4H), 7.44 – 7.36 (m, 1H), 7.00 – 6.95 (m, 2H), 4.57 (dd, *J* = 8.0, 6.9 Hz, 1H), 3.85 (s, 2H), 1.88 – 1.74 (m, 2H), 1.74 – 1.62 (m, 1H), 0.92 (dd, *J* = 19.2, 6.6 Hz, 6H). ^{13}C NMR (126 MHz, DMSO) δ 167.9, 166.7, 137.2, 135.2, 133.9, 126.7, 126.4, 125.1, 119.9, 119.6, 57.8, 42.8, 37.5, 25.0, 22.4, 21.6. MS (ESI^{+}) m/z 365.07 [M+H]$^{+}$.

***N*-(3-Aminophenyl)-2-(3,4-dichlorophenyl)acetamide (184).**

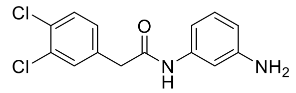

Compound **184** was synthesized according to general procedure A-1, using 2-(3,4-dichlorophenyl)acetic acid (591 mg, 2.88 mmol), *N*-Boc-*m*-phenylendiamine (500 mg, 2.40 mmol) and EDC·HCl (552 mg, 2.88 mmol) in DCM (30 mL). The

reaction was stirred at r.t. for 3 h. After the workup, *tert*-butyl {3-[2-(3,4-dichlorophenyl)acetamido]phenyl}carbamate was obtained in quantitative yield. In the next step Boc-protected aniline (300 mg, 0.76 mmol) was treated with TFA (0.7 mL) in DCM (2.7 mL) to afford the product as a beige solid (195 mg, 87%; 86% over 2 steps). ^{1}H NMR (500 MHz, DMSO) δ 9.86 (s, 1H), 7.58 (dd, *J* = 5.1, 3.1 Hz, 2H), 7.30 (dd, *J* = 8.3, 2.0 Hz, 1H), 6.92 – 6.85 (m, 2H), 6.74 – 6.62 (m, 1H), 6.24 (dd, *J* = 7.6, 1.7 Hz, 1H), 5.05 (s, 2H), 3.62 (s, 2H). ^{13}C NMR (126 MHz, DMSO) δ 167.9, 149.1, 139.6, 137.3, 131.2, 130.7, 130.4, 129.6, 129.2, 129.0, 109.5, 107.1, 104.9, 42.1. MS (ESI^{+}) m/z 295.01 [M+H]$^{+}$.

2-Bromo-*N*-{3-[2-(3,4-dichlorophenyl)acetamido]phenyl}-4-methylpentanamide (203).

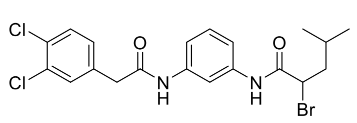

Compound **203** was synthesized according to general procedure A-1, using 2-bromo-4-methylpentanoic acid (154 mg, 0.79 mmol), **184** (194 mg, 0.66 mmol) and EDC·HCl (151 mg, 0.79 mmol) in DCM (20 mL). The reaction was stirred at r.t. for 2 h. Product was obtained after the column chromatography (Hex/EtOAc=7/3) (194 mg, 62%). ^{1}H NMR (500 MHz, DMSO) δ 10.36 (s, 1H), 10.24 (s, 1H), 7.98 (d, *J* = 1.9 Hz, 1H), 7.59 (t, *J* = 5.5 Hz, 2H), 7.36 – 7.20 (m, 4H), 4.63 (t, *J* = 7.6 Hz, 1H), 3.68 (s, 2H), 1.88 (dtd, *J* = 21.3, 14.1, 7.4 Hz, 2H), 1.63 (dt, *J* = 13.4, 6.7 Hz, 1H), 0.93 (d, *J* = 6.7 Hz, 3H), 0.88 (d, *J* = 6.6 Hz, 3H). ^{13}C NMR (126 MHz, DMSO) δ 168.3, 167.0, 139.4, 138.8, 137.0, 131.3, 130.7, 130.4, 129.7, 129.3, 129.2, 114.8, 114.4, 110.3, 48.4, 42.8, 42.0, 26.2, 22.1, 21.7. MS (ESI^{+}) m/z 471.08 [M+H]$^{+}$.

***N*-(4-Amino-2-fluorophenyl)-2-(3,4-dichlorophenyl)acetamide (151).**

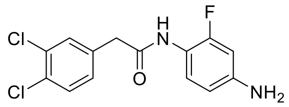

Compound **151** was synthesized according to general procedure A-1, using 2-(3,4-dichlorophenyl)acetic acid (267 mg, 1.30 mmol), *tert*-butyl (4-amino-3-fluorophenyl)carbamate (250 mg, 1.10 mmol), TBTU (530 mg, 1.65 mmol) and NMM (180 µL, 1.65 mmol) in DCM (10 mL). The reaction was stirred at r.t. overnight. Solid was filtered and washed with DCM. In the next step Boc-protected aniline (342 mg, 0.83 mmol) was treated with TFA (0.8 mL) in DCM (3 mL) to afford the product as a beige solid (259 mg, 100%; 76% over 2 steps). ^{1}H NMR (500 MHz, DMSO) δ 9.50 (s, 1H), 7.60 – 7.54 (m, 2H), 7.30 (dd, *J* = 8.3, 2.0 Hz, 1H), 7.17 (t, *J* = 8.8 Hz, 1H), 6.38 – 6.27 (m, 2H), 5.28 (s, 2H), 3.63 (s, 2H). ^{13}C NMR (126 MHz, DMSO) δ 168.3, 155.9 (d, *J* = 242.3 Hz), 148.0 (d, *J* = 10.8 Hz), 137.4, 131.1, 130.7, 130.4, 129.6, 129.2, 126.8 (d, *J* = 3.2 Hz), 113.2 (d, *J* = 12.9 Hz), 109.2 (d, *J* = 2.2 Hz), 100.3 (d, *J* = 22.6 Hz), 41.1. MS (ESI^{+}) m/z 313.03 [M+H]$^{+}$.

Diethyl [1-({4-[2-(3,4-dichlorophenyl)acetamido]-3-fluorophenyl}amino)-4-methyl-1-oxopentan-2-yl]phosphonate (154).

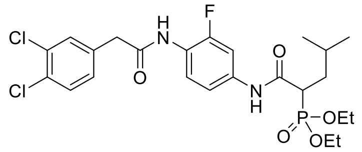

Compound **154** was synthesized according to general procedure A-3, using **214** (194 mg, 0.77 mmol), **151** (200 mg, 0.64 mmol), TBTU (308 mg, 0.96 mmol) and NMM (140 µL, 1.28 mmol) in DCM (5 mL). The reaction was stirred at r.t. for 4 h. Crude was purified using column

chromatography (EtOAc to EtOAc/MeOH=95/5) to give the product as a white solid (262 mg, 75%). ^{1}H NMR (500 MHz, DMSO) δ 10.31 (s, 1H), 9.93 (s, 1H), 7.72 (t, *J* = 8.8 Hz, 1H), 7.67 (dd, *J* = 13.1, 2.2 Hz, 1H), 7.59 (d, *J* = 8.2 Hz, 2H), 7.32 (dd, *J* = 8.3, 1.8 Hz, 1H), 7.17 (dd, *J* = 8.8, 1.5 Hz, 1H), 4.10 – 3.95 (m, 4H), 3.73 (s, 2H), 3.16 (ddd, *J* = 22.5, 11.2, 2.9 Hz, 1H), 2.00 – 1.90 (m, 1H), 1.53 – 1.35 (m, 2H), 1.26 – 1.13 (m, 6H), 0.87 (d, *J* = 6.4 Hz, 6H). ^{13}C NMR (126 MHz, DMSO) δ 168.7, 166.7 (d, *J* = 4.6 Hz), 153.6 (d, *J* = 243.7 Hz), 137.1, 136.4 (d, *J* = 10.3 Hz), 131.3, 130.8, 130.5, 129.8, 129.3, 121.1 (d, *J* = 12.1 Hz), 114.6, 106.3 (d, *J* = 24.8 Hz), 62.1 (d, *J* = 6.4 Hz), 61.9 (d, *J* = 6.5 Hz), 44.6 (d, *J* = 129.9 Hz), 41.2, 35.4 (d, *J* = 4.6 Hz), 26.4 (d, *J* = 14.9 Hz), 23.0, 21.3, 16.3 (d, *J* = 5.6 Hz). MS (ESI^{+}) m/z 547.21 [M+H]$^{+}$.

***N*-[4-Amino-2-(trifluoromethyl)phenyl]-2-(3,4-dichlorophenyl)acetamide (152).**

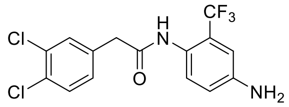

Compound **152** was synthesized according to general procedure A-1, using 2-(3,4-dichlorophenyl)acetic acid (178 mg, 0.87 mmol), *tert*-butyl [4-amino-3-(trifluoromethyl)phenyl]carbamate (200 mg, 0.72 mmol), TBTU (347 mg, 1.08 mmol) and NMM (120 µL, 1.08 mmol) in DCM (10 mL). The reaction was stirred at r.t. overnight. Crude was purified using column chromatography (Hex/EtOAc=7/3) to give *tert*-butyl {4-[2-(3,4-dichlorophenyl)acetamido]-3-(trifluoromethyl)phenyl}carbamate (232 mg, 70%). In the next step Boc-protected aniline (222 mg, 0.48 mmol) was treated with TFA (0.8 mL) in DCM (3 mL) to afford the product as a beige solid (158 mg, 91%; 64% over 2 steps). ^{1}H NMR (500 MHz, DMSO) δ 9.44 (s, 1H), 7.58 (dd, *J* = 9.4, 5.1 Hz, 2H), 7.29 (dd, *J* = 8.3, 2.0 Hz, 1H), 6.97 (d, *J* = 8.5 Hz, 1H), 6.85 (d, *J* = 2.5 Hz, 1H), 6.74 (dd, *J* = 8.5, 2.5 Hz, 1H), 5.55 (s, 2H), 3.61 (s, 2H). ^{13}C NMR (126 MHz, DMSO) δ 169.4, 147.8, 137.3, 131.6, 131.1, 130.7, 130.3, 129.6, 129.1, 126.2 (q, *J* = 28.6 Hz), 123.7 (q, *J* = 273.4 Hz), 122.2, 117.0, 110.1 (q, *J* = 5.2 Hz), 41.0. MS (ESI^{+}) m/z 363.04 [M+H]$^{+}$.

Diethyl [1-({4-[2-(3,4-dichlorophenyl)acetamido]-3-(trifluoromethyl)phenyl}amino)-4-methyl-1-oxopentan-2-yl]phosphonate (155).

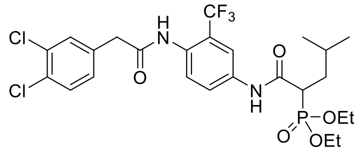

Compound **155** was synthesized according to general procedure A-3, using **214** (102.5 mg, 0.41 mmol), **152** (123 mg, 0.34 mmol), TBTU (163 mg, 0.51 mmol) and NMM (75 µL, 0.68 mmol) in DMF (3 mL). The reaction was stirred at r.t. overnight. Crude was purified using column chromatography (EtOAc to EtOAc/MeOH=95/5) to give the product as a white solid (143 mg, 70%). ^{1}H NMR (500 MHz, DMSO) δ 10.50 (s, 1H), 9.78 (s, 1H), 8.07 (d, *J* = 2.1 Hz, 1H), 7.73 (dd, *J* = 8.7, 1.8 Hz, 1H), 7.60 (d, *J* = 8.2 Hz, 2H), 7.40 (d, *J* = 8.7 Hz, 1H), 7.35 – 7.25 (m, 1H), 4.13 – 3.95 (m, 4H), 3.70 (s, 2H), 3.18 (ddd, *J* = 22.5, 11.2, 2.8 Hz, 1H), 2.02 – 1.92 (m, 1H), 1.54 – 1.37 (m, 2H), 1.21 (q, *J* = 7.1 Hz, 6H), 0.87 (d, *J* = 6.1 Hz, 6H). ^{13}C NMR (126 MHz, DMSO) δ 169.4, 167.0 (d, *J* = 4.7 Hz), 137.5, 136.9, 131.4, 131.2, 130.7, 130.4, 130.1, 129.6, 129.3, 125.4 (q, *J* = 28.9 Hz), 123.3 (q, *J* = 274.7 Hz), 122.9, 116.1 (q, *J* = 6.3 Hz), 62.1 (d, *J* = 6.4 Hz), 61.9 (d, *J* = 6.6 Hz), 44.7 (d, *J* = 129.8 Hz), 41.0, 35.3 (d, *J* = 4.8 Hz), 26.4 (d, *J* = 14.9 Hz), 22.9, 21.2, 16.3, 16.3. MS (ESI^{+}) m/z 597.23 [M+H]$^{+}$.

***N*-(4-Aminophenyl)-1-(3,4-dichlorophenyl)methanesulfonamide (153).**

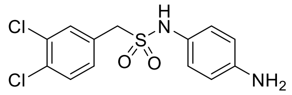

Compound **153** was synthesized over two steps according to general procedure B, using (3,4-dichlorophenyl)methanesulfonyl chloride (410 mg, 1.58 mmol), *N*-Boc-*p*-phenylendiamine (300 mg, 1.44 mmol) and Et_3N (240 µL, 1.73 mmol) in DCM (10 mL). The reaction was stirred at r.t. overnight. Crude was purified using column chromatography (Hex/EtOAc=7/3) to give *tert*-butyl (4-{[(3,4-dichlorophenyl)methyl]sulfonamido}phenyl)carbamate (326 mg, 52%). In the next step Boc-protected aniline (270 mg, 0.62 mmol) was treated with TFA (0.5 mL) in DCM (2.5 mL) to afford the product as a beige solid (237 mg, 96%; 50% over 2 steps). ^1H NMR (500 MHz, DMSO) δ 9.19 (s, 1H), 7.64 (d, *J* = 8.3 Hz, 1H), 7.52 (d, *J* = 2.0 Hz, 1H), 7.28 (dd, *J* = 8.3, 2.0 Hz, 1H), 6.97 – 6.81 (m, 2H), 6.59 – 6.48 (m, 2H), 5.02 (s, 2H), 4.34 (s, 2H). ^{13}C NMR (126 MHz, DMSO) δ 146.4, 132.7, 131.2, 131.2, 130.9, 130.8, 130.5, 125.7, 123.9, 114.2, 54.9. MS (ESI^-) m/z 329.0 $[M-H]^-$.

Diethyl {1-[(4-{[(3,4-dichlorophenyl)methyl]sulfonamido}phenyl)amino]-4-methyl-1-oxopentan-2-yl}phosphonate (156).

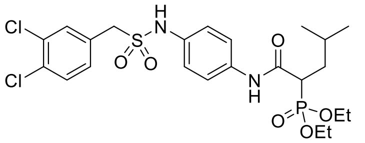

Compound **156** was synthesized according to general procedure A-2, using **153** (97.4 mg, 0.29 mmol), **214** (88.3 mg, 0.35 mmol), EDC·HCl (67.6 mg, 0.35 mmol), HOBt (47.3 mg, 0.35 mmol) and DIPEA (60 µL, 0.35 mmol) in DCM (3 mL). Crude product as a beige solid (60 mg, 36%) was used in the next step without further purification. MS (ESI^-) m/z 563.29 $[M-H]^-$.

***N*-(4-Benzoylphenyl)-2-bromo-4-methylpentanamide (180).**

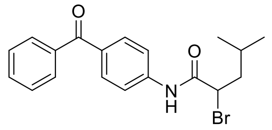

Compound **180** was synthesized according to general procedure A-1, using 2-bromo-4-methylpentanoic acid (197 mg, 1.00 mmol), (4-aminophenyl)(phenyl)methanone (166 mg, 0.84 mmol) and EDC·HCl (192 mg, 1.00 mmol) in DCM (15 mL). The reaction was stirred at r.t. overnight. Crude product was purified using column chromatography (Hex/EtOAc=8/2) to give **180** as a yellow oil (198 mg, 63%). ^1H NMR (500 MHz, $CDCl_3$) δ 8.20 (s, 1H), 7.87 – 7.83 (m, 2H), 7.80 – 7.76 (m, 2H), 7.70 – 7.66 (m, 2H), 7.62 – 7.57 (m, 1H), 7.52 – 7.46 (m, 2H), 4.47 (dd, *J* = 9.4, 5.5 Hz, 1H), 2.04 (qdd, *J* = 14.5, 8.9, 5.4 Hz, 2H), 1.96 – 1.86 (m, 1H), 1.02 (d, *J* = 6.6 Hz, 3H), 0.96 (d, *J* = 6.6 Hz, 3H). ^{13}C NMR (126 MHz, $CDCl_3$) δ 195.7, 167.5, 141.2, 137.8, 133.9, 132.5, 131.7, 130.1, 128.5, 119.1, 50.5, 44.6, 26.6, 22.8, 21.2. MS (ESI^+) m/z 374.11 $[M+H]^+$.

(1-((4-Benzoylphenyl)amino)-4-methyl-1-oxopentan-2-yl)phosphonic acid (189)

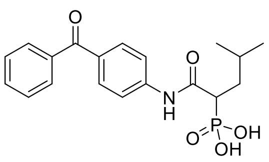

Compound **189** was synthesized over two steps according to the general procedure C using **180** (195 mg, 0.52 mmol) and triethyl phosphite (2 mL). The resultant oil was purified by column chromatography (Hex/EtOAc=7/3 to 3/7) to give diethyl phosphonate as transparent oil (132 mg, 59%). The obtained product was then treated with bromotrimethylsilane (275 µL, 2.08 mmol) in DCM (6 mL). TMS ester was cleaved using MeOH (6 mL). Solvents were concentrated in vacuo and the resultant oil was purified

by preparative HPLC (CH_3CN (HCOOH 0.05%)/H_2O (HCOOH 0.05%) = 1/9 to 10/0). The product was obtained as white solid (90 mg, 80%; 47% over two steps). ^{1}H NMR (500 MHz, DMSO) δ 10.37 (s, 1H), 7.81 – 7.77 (m, 2H), 7.75 – 7.69 (m, 4H), 7.66 (ddd, *J* = 8.7, 2.5, 1.2 Hz, 1H), 7.55 (dd, *J* = 10.6, 4.6 Hz, 2H), 3.06 (ddd, *J* = 22.5, 11.2, 2.5 Hz, 1H), 2.03 – 1.93 (m, 1H), 1.55 – 1.39 (m, 2H), 0.87 (d, *J* = 5.8 Hz, 6H). ^{13}C NMR (126 MHz, DMSO) δ 194.6, 168.7 (d, *J* = 4.9 Hz), 143.6, 137.7, 132.3, 131.2, 129.4, 128.5, 118.3, 46.3 (d, *J* = 126.0 Hz), 35.7, 26.6 (d, *J* = 14.4 Hz), 23.2, 21.4. ^{31}P NMR (202 MHz, DMSO) δ 19.32. HRMS (ESI$^-$) calculated for $C_{19}H_{21}NO_5P^-$ [M-H]$^-$ 374.1163, found 374.1163.

***N*-(4-benzylphenyl)-2-bromo-4-methylpentanamide (181).**

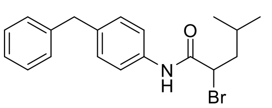

Compound **181** was synthesized according to general procedure A-1, using 2-bromo-4-methylpentanoic acid (106 mg, 0.54 mmol), 4-benzylaniline (82.5 mg, 0.45 mmol) and EDC·HCl (104 mg, 0.54 mmol) in DCM (10 mL). The reaction was stirred at r.t. overnight. After the workup, **181** was obtained as a beige solid (158 mg, 97%). The compound was taken through to the next step without purification.

2-Bromo-*N*-{4-[(3,4-dichlorobenzyl)thio]phenyl}-4-methylpentanamide (182).

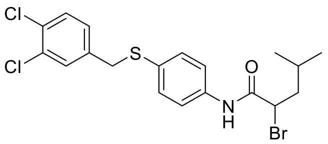

Compound **182** was synthesized according to general procedure A-1, using 2-bromo-4-methylpentanoic acid (102 mg, 0.52 mmol), 4-((3,4-dichlorobenzyl)thio)aniline (124 mg, 0.44 mmol) and EDC·HCl (100 mg, 0.52 mmol) in DCM (5 mL). The reaction was stirred at r.t. overnight. Crude product was purified using column chromatography (Hex/EtOAc=8/2) to give **182** as colourless crystals (146 mg, 73%). ^{1}H NMR (500 MHz, $CDCl_3$) δ 7.98 (s, 1H), 7.46 (d, *J* = 8.6 Hz, 2H), 7.32 (d, *J* = 8.3 Hz, 2H), 7.29 – 7.25 (m, 2H), 7.04 (dd, *J* = 8.2, 2.1 Hz, 1H), 4.43 (dd, *J* = 9.5, 5.4 Hz, 1H), 3.97 (s, 2H), 2.09 – 1.83 (m, 3H), 1.00 (d, *J* = 6.6 Hz, 3H), 0.95 (d, *J* = 6.5 Hz, 3H). ^{13}C NMR (126 MHz, $CDCl_3$) δ 167.2, 138.2, 136.7, 132.6, 132.4, 131.3, 130.8, 130.8, 130.5, 128.3, 120.5, 50.8, 44.7, 39.2, 26.6, 22.8, 21.2. MS (ESI$^-$) m/z 458.0 [M-H]$^-$.

4-((3,4-Dichlorobenzyl)oxy)aniline (171).

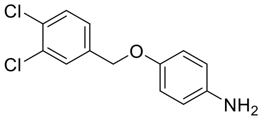

Compound **171** was synthesized over two steps. In the first step, *tert*-butyl (4-hydroxyphenyl)carbamate (1 g, 4.78 mmol) was dissolved in DMF (15 mL). K_2CO_3 (1.32 g, 9.56 mmol) was added and the reaction mixture stirred for 15 min. 4-(Bromomethyl)-1,2-dichlorobenzene (840 µL, 5.74 mmol) was added dropwise to the reaction mixture and left to stir overnight at r.t. Water was added, extracted with EtOAc, washed with brine, dried over anhydrous sodium sulfate, filtered and concentrated under reduced pressure to afford tert-butyl {4-[(3,4-dichlorobenzyl)oxy]phenyl}carbamate as a off-white solid (1.11 g, 63%). In the next step (according to procedure B, Step 2), Boc-protected aniline (400 mg, 1.09 mmol) was treated with TFA (0.8 mL) in DCM (3 mL) to afford the product as a beige solid (400 mg, 92%; 58% over 2 steps). ^{1}H NMR (500 MHz, DMSO) δ 7.64 (dd, *J* = 13.4, 5.1 Hz, 2H), 7.40 (dd, *J* = 8.3, 2.0 Hz, 1H), 6.76 – 6.67 (m, 2H), 6.56 – 6.47 (m, 2H), 4.96 (s, 2H), 4.77 (s, 2H). ^{13}C NMR (126 MHz, DMSO) δ 149.3, 142.6, 139.1, 131.0, 130.6, 130.0, 129.3, 127.7, 115.8, 115.0, 68.2. MS (ESI$^+$) m/z 268.1 [M+H]$^+$.

2-Bromo-*N*-{4-[(3,4-dichlorobenzyl)oxy]phenyl}-4-methylpentanamide (183).

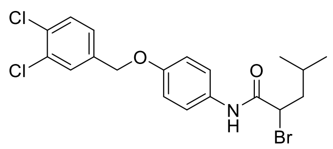

Compound **183** was synthesized according to general procedure A-1, using 2-bromo-4-methylpentanoic acid (108 mg, 0.55 mmol), 4-[(3,4-dichlorobenzyl)oxy]aniline (124 mg, 0.46 mmol) and EDC·HCl (106 mg, 0.55 mmol) in DCM (5 mL). The reaction was stirred at r.t. overnight. After the workup, **183** was obtained as beige solid (197 mg, 96%). ^{1}H NMR (500 MHz, $CDCl_3$) δ 7.94 (s, 1H), 7.53 (d, *J* = 1.8 Hz, 1H), 7.46 – 7.42 (m, 3H), 7.26 – 7.23 (m, 1H), 6.96 – 6.89 (m, 2H), 5.00 (s, 2H), 4.44 (dd, *J* = 9.5, 5.3 Hz, 1H), 2.09 – 1.86 (m, 3H), 1.00 (d, *J* = 6.6 Hz, 3H), 0.95 (d, *J* = 6.5 Hz, 3H). ^{13}C NMR (126 MHz, $CDCl_3$) δ 167.1, 155.6, 137.3, 132.9, 132.1, 131.0, 130.7, 129.4, 126.6, 122.0, 115.4, 69.0, 50.9, 44.8, 26.6, 22.8, 21.2. MS (ESI+) m/z 444.04 [M+H]+.

(3,4-Dichlorophenyl)(4-nitrophenyl)sulfane (48).

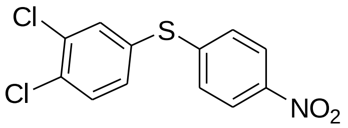

Compound **48** was synthesized according to general procedure E, using 1-fluoro-4-nitrobenzene (282 mg, 2 mmol), 3,4-dichlorobenzenethiol (430 mg, 2.4 mmol), and K_2CO_3 (414 mg, 3 mmol) in NMP (20 mL). The product was used in the next step without further purification. Yield (478 mg, 80%). MS (ESI^+) m/z 300.03 $[M+H]^+$.

4-(3,4-Dichlorophenylthio)benzenamine (62).

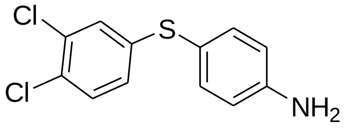

Compound **62** was synthesized according to general procedure F, using **48** (300 mg, 1 mmol), Fe (280 mg, 5 mmol), and NH_4Cl (28 mg, 0.5 mmol) in Ethanol/water (2/1) mixture (15 mL). The crude product was purified using column chromatography (DCM). The product was obtained as yellow solid (145 mg, 54%). ^{1}H NMR (500 MHz, Acetone) δ ppm: 7.28 (d, *J* = 8.5 Hz, 1H), 7.17 – 7.08 (m, 2H), 6.97 (d, *J* = 2.2 Hz, 1H), 6.85 (dd, *J* = 8.5, 2.2 Hz, 1H), 6.68 – 6.60 (m, 2H), 5.07 (s, 2H). ^{13}C NMR (126 MHz, Acetone) δ ppm: 150.5, 142.4, 137.0, 132.2, 130.6, 127.7, 126.8, 125.6, 115.4, 114.6. MS (ESI^+) m/z 270.17 $[M+H]^+$.

Diethyl 1-[4-(3,4-dichlorophenylthio)phenylcarbamoyl]-3-methylbutylphosphonate (76).

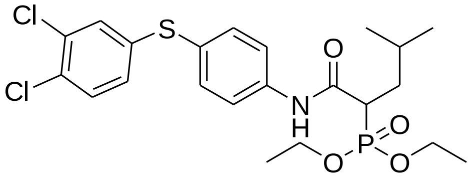

Compound **76** was synthesized according to general procedure A-2, using **62** (135 mg, 0.50 mmol), 2-(diethoxy-phosphoryl)-4-methyl-pentanoic acid **29** (252 mg, 1 mmol) and EDC·HCl (191 mg, 1 mmol), HOBt (135 mg, 1 mmol) and DIPEA (205 µL, 1.2 mmol) in DCM (20 mL). The reaction was stirred at room temperature for 24 h. The crude product was purified by automated column chromatography (DCM to DCM/MeOH 2%). The product was obtained as white solid (232 mg, 92%). ^{1}H NMR (500 MHz, $CDCl_3$) δ ppm: 8.27 (s, 1H), 7.57 – 7.45 (m, 2H), 7.40 – 7.31 (m, 2H), 7.29 – 7.22 (m, 1H), 7.19 (dd, *J* = 4.7, 2.5 Hz, 1H), 7.01 – 6.90 (m, 1H), 4.45 – 4.36 (m, 1H), 4.14 – 3.96 (m, 4H), 2.03 – 1.77 (m, 3H), 1.24 (t, *J* = 4.7 Hz, 3H), 1.21 (t, *J* = 4.7 Hz, 3H), 0.93 (d, *J* = 6.4 Hz, 3H), 0.91 (d, *J* = 6.2 Hz, 3H). MS (ESI^+) m/z 504.03 $[M+H]^+$

4-(4-Nitrophenoxy)-1,2-dichlorobenzene (49).

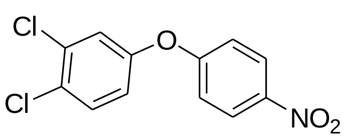

Compound **49** was synthesized according to general procedure E, using 1-fluoro-4-nitrobenzene (282 mg, 2 mmol), 3,4-dichlorophenol (391 mg, 2.4 mmol), and K_2CO_3 (414 mg, 3 mmol) in NMP (20 mL). The product was used in the next step without further purification. Yield (466 mg, 78%). MS (ESI$^+$) m/z 284.03 [M+H]$^+$.

4-(3,4-Dichlorophenoxy)benzenamine (63).

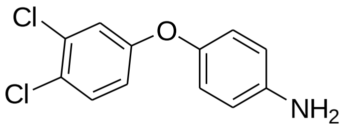

Compound **63** was synthesized according to general procedure F, using **49** (284 mg, 1 mmol), Fe (280 mg, 5 mmol), and NH_4Cl (28 mg, 0.5 mmol) in Ethanol/water (2/1) mixture (15 mL). The crude product was purified using column chromatography (DCM). The product was obtained as beige solid (133 mg, 49%). ^{1}H NMR (500 MHz, Acetone) δ ppm: 7.25 (d, *J* = 8.9 Hz, 1H), 6.97 – 6.86 (m, 1H), 6.84 (d, *J* = 2.9 Hz, 1H), 6.67 (d, *J* = 8.8 Hz, 2H), 6.57 (d, *J* = 8.8 Hz, 2H). MS (ESI$^+$) m/z 254.10 [M+H]$^+$.

Diethyl 1-[4-(3,4-dichlorophenoxy)phenylcarbamoyl]-3-methylbutylphosphonate (77).

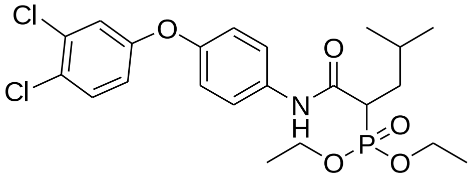

Compound **77** was synthesized according to general procedure A-2, using **63** (127 mg, 0.50 mmol), 2-(diethoxy-phosphoryl)-4-methyl-pentanoic acid **29** (252 mg, 1 mmol) and EDC·HCl (191 mg, 1 mmol), HOBt (135 mg, 1 mmol) and DIPEA (205 µL, 1.2 mmol) in DCM (20 mL). The reaction was stirred at room temperature for 24 h. The crude product was purified by automated column chromatography (DCM to DCM/MeOH 1%). The product was obtained as white solid (222 mg, 88%). MS (ESI$^+$) m/z 488.01 [M+H]$^+$.

***N*-(3,4-Dichlorophenyl)-4-nitrobenzamide (51).**

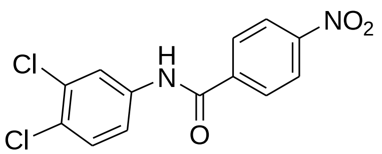

Compound **51** was synthesized according to general procedure B, using 3,4-dichlorobenzenamine (162 mg, 1 mmol), 4-nitrobenzoic acid (504 mg, 2 mmol) and EDC·HCl (334 mg, 2 mmol) in DCM (20 mL). The reaction was stirred at room temperature for 24 h. The crude product was purified by automated column chromatography (DCM to DCM/MeOH 1%). The product was obtained as brown solid (515 mg, 83%). ^{1}H NMR (500 MHz, DMSO) δ ppm: 10.80 (s, 1H), 8.43 – 8.34 (m, 2H), 8.21 – 8.17 (m, 2H), 8.15 (d, *J* = 2.4 Hz, 1H), 7.76 (dd, *J* = 8.8, 2.4 Hz, 1H), 7.65 (d, *J* = 8.8 Hz, 1H). ^{13}C NMR (126 MHz, DMSO) δ ppm: 164.7, 149.8, 140.4, 139.3, 131.4, 131.2, 129.8, 126.1, 124.1, 122.1, 120.9. MS (ESI$^+$) m/z 311.03 [M+H]$^+$.

4-Amino-*N*-(3,4-dichlorophenyl)benzamide (65).

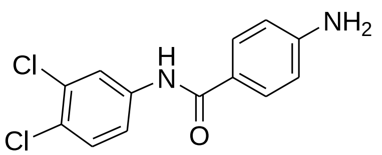

Compound **65** was synthesized according to general procedure F, using **51** (311 mg, 1 mmol), Fe (280 mg, 5 mmol), and NH_4Cl (28 mg, 0.5 mmol) in Ethanol/water (2/1) mixture (15 mL). The product was used in the next step without further purification. Yield (165 mg, 59%). ^{1}H NMR (500 MHz, Acetone) δ ppm: 9.43 (s, 1H), 8.23-8.19 (m, 1H), 7.80-7.69 (m, 3H), 7.48-

7.43 (m, 1H), 6.73-6.65 (m, 2H), 5.34 (s, 2H). ^{13}C NMR (126 MHz, Acetone) δ ppm: 165.6, 152.4, 140.0, 131.6, 130.3, 129.4, 125.1, 121.7, 121.3, 119.7, 113.2. MS (ESI^{+}) m/z 281.01 [M+H]$^{+}$.

Diethyl 1-[4-(3,4-dichlorophenylcarbamoyl)phenylcarbamoyl]-3-methylbutylphosphonate (79).

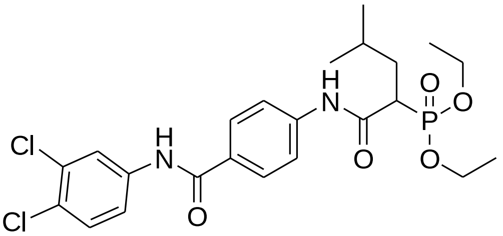

Compound **79** was synthesized according to general procedure A-2, using **65** (140 mg, 0.50 mmol), 2-(diethoxy-phosphoryl)-4-methyl-pentanoic acid **29** (252 mg, 1 mmol) and EDC·HCl (191 mg, 1 mmol), HOBt (135 mg, 1 mmol) and DIPEA (205 µL, 1.2 mmol) in DCM (20 mL). The reaction was stirred at room temperature for 24 h. The crude product was purified by automated column chromatography (DCM). The product was obtained as white solid (190 mg, 74%). ^{1}H NMR (500 MHz, Acetone) δ ppm: 9.75 (s, 1H), 9.70 (s, 1H), 8.21 (d, *J* = 2.5 Hz, 1H), 8.04 – 7.96 (m, 2H), 7.81 (d, *J* = 8.8 Hz, 2H), 7.76 (dd, *J* = 8.8, 2.4 Hz, 1H), 7.50 (d, *J* = 8.8 Hz, 1H), 4.59 (dd, *J* = 8.5, 6.2 Hz, 1H), 4.14 – 3.90 (m, 4H), 1.99 – 1.87 (m, 2H), 1.83 (ddd, *J* = 13.9, 9.0, 6.7 Hz, 1H), 1.23 (dt, *J* = 9.1, 7.1 Hz, 6H), 0.98 (d, *J* = 6.6 Hz, 3H), 0.94 (d, *J* = 6.5 Hz, 3H). ^{13}C NMR (126 MHz, DMSO) δ ppm: 167.6 (d, *J* = 6.0 Hz), 165.0, 141.9, 139.5, 131.7, 130.4, 128.6, 125.6, 125.3, 121.8, 120.6, 118.6, 62.5 (dd, *J* = 24.1, 6.5 Hz), 46.7 (d, *J* = 127.3 Hz), 36.2 (d, *J* = 4.1 Hz), 27.0 (d, *J* = 14.3 Hz), 23.7, 21.8, 16.8 (d, *J* = 5.6 Hz). ^{31}P NMR (202 MHz, DMSO) δ ppm: 23.71. MS (ESI^{+}) m/z 515.45 [M+H]$^{+}$.

4-Nitro-*N*-(3,4-dichloro-phenyl)-benzenesulfonamide (50).

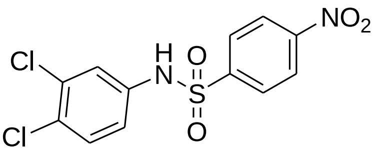

3,4-dichlorobenzenamine (324 mg, 2 mmol) was dissolved in pyridine absolute (15 mL) and 4-nitrobenzene-1-sulfonyl chloride (532 mg, 2.4 mmol) was added. The reaction mixture was stirred overnight at room temperature. The reaction was quenched by adding 10 mL of 2 N HCl and extracted with ethyl acetate (3 × 50 mL). The organic layers were washed with saturated $NaHCO_3$ and brine, dried over magnesium sulfate, filtered and concentrated to dryness. The crude product was purified by automated column chromatography (DCM). The product was obtained as white yellowish brown solid (330 mg, 67%). MS (ESI^{+}) m/z 247.03 [M+H]$^{+}$.

4-Amino-*N*-(3,4-dichloro-phenyl)-benzenesulfonamide (64).

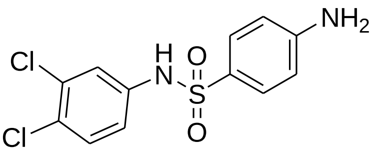

Compound **64** was synthesized according to general procedure F, using **50** (311 mg, 1 mmol), Fe (280 mg, 5 mmol), and NH_4Cl (28 mg, 0.5 mmol) in Ethanol/water (2/1) mixture (15 mL). The crude product was purified using column chromatography (DCM to DCM/MeOH 1%). The product was obtained as beige solid (173 mg, 55%). ^{1}H NMR (500 MHz, Acetone) δ ppm: 8.99 (s, 1H), 7.50 (d, *J* = 8.7 Hz, 2H), 7.42 (d, *J* = 8.7 Hz, 1H), 7.40 (d, *J* = 2.5 Hz, 1H), 7.18 (dd, *J* = 8.7, 2.5 Hz, 1H), 6.69 (d, *J* = 8.8 Hz, 2H), 5.54 (s, 2H). ^{13}C NMR (126 MHz, Acetone) δ ppm: 153.1, 138.9, 132.0, 130.9, 129.2, 126.3, 125.2, 121.2, 119.7, 113.1. MS (ESI^{+}) m/z 317.03 [M+H]$^{+}$.

Diethyl 1-[4-(3,4-dichlorophenylsulfamoyl)phenylcarbamoyl]-3-methylbutylphosphonate (78).

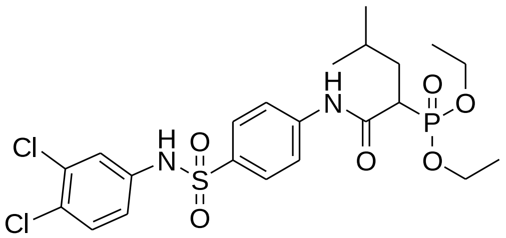

Compound **78** was synthesized according to general procedure A-2, using **64** (159 mg, 0.50 mmol), 2-(diethoxy-phosphoryl)-4-methyl-pentanoic acid **29** (252 mg, 1 mmol) and EDC·HCl (191 mg, 1 mmol), HOBt (135 mg, 1 mmol) and DIPEA (205 µL, 1.2 mmol) in DCM (20 mL). The reaction was stirred at room temperature for 24 h. The crude product was purified by automated column chromatography (DCM to DCM/MeOH 2%). The product was obtained as white solid (185 mg, 67%). ^{1}H NMR (500 MHz, Acetone) δ ppm: 9.76 (s, 1H), 9.20 (s, 1H), 7.73 – 7.64 (m, 4H), 7.31 (d, *J* = 8.7 Hz, 1H), 7.28 (d, *J* = 2.5 Hz, 1H), 7.07 (dd, *J* = 8.7, 2.6 Hz, 1H), 4.46 (dd, *J* = 8.0, 7.1 Hz, 1H), 4.10 – 3.82 (m, 4H), 1.89 – 1.75 (m, 2H), 1.64 – 1.58 (m, 1H), 1.20 (dt, *J* = 9.1, 7.1 Hz, 6H),0.82 (d, *J* = 6.7 Hz, 3H), 0.76 (d, *J* = 6.6 Hz, 3H). MS (ESI$^+$) m/z 551.71 [M+H]$^+$.

Diethyl 1-[4-(hydroxymethyl)phenylcarbamoyl]-3-methylbutylphosphonate (161).

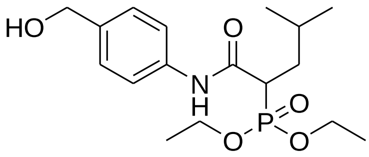

Compound **161** was synthesized according to general procedure A-2, using (4-aminophenyl)methanol (248 mg, 2 mmol), 2-(diethoxy-phosphoryl)-4-methyl-pentanoic acid **29** (1008 mg, 4 mmol) and EDC·HCl (764 mg, 4 mmol), HOBt (540 mg, 4 mmol) and DIPEA (984 µL, 4.8 mmol) in DCM (30 mL). The reaction was stirred at room temperature for 24 h. The crude product was purified by automated column chromatography (DCM to DCM/MeOH 3%). The product was obtained as white solid (514 mg, 72%). ^{1}H NMR (500 MHz, $CDCl_3$) δ ppm: 9.25 (s, 1H), 7.37 (d, *J* = 8.5 Hz, 2H), 7.05 (d, *J* = 8.4 Hz, 2H), 4.48 (d, *J* = 5.9 Hz, 2H), 4.22 – 4.10 (m, 2H), 4.08 (dd, *J* = 14.6, 7.5 Hz, 2H), 3.14 (ddd, *J* = 22.7, 11.3, 3.2 Hz, 1H), 2.12 – 2.00 (m, 1H), 1.62 (ddd, *J* = 10.4, 8.4, 5.5 Hz, 1H), 1.51 – 1.41 (m, 1H), 1.30 (t, *J* = 5.1 Hz, 3H), 1.28 (t, *J* = 5.1 Hz, 3H), 0.88 (d, *J* = 6.6 Hz, 3H), 0.86 (d, *J* = 6.6 Hz, 3H). MS (ESI$^+$) m/z 357.98 [M+H]$^+$.

Diethyl 1-[4-(chloromethyl)phenylcarbamoyl]-3-methylbutylphosphonate (162).

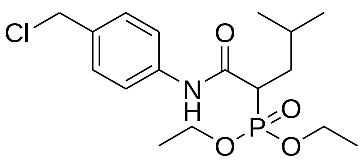

To arylmethanol **162** (357 mg, 1 mmol) in THF (10 ml) at 0ºC was added thionyl chloride $SOCl_2$ (5 mmol). The reaction was stirred at room temperature for 3 h. The reaction was concentrated under reduced pressure. The crude product was directly used in the next step without any further purification. (DCM to DCM/MeOH 1%). The product was obtained as pale yellow solid (150 mg, 40%). ^{1}H NMR (500 MHz, DMSO) δ ppm: 10.31 (s, 1H), 7.60 (d, *J* = 8.4 Hz, 2H), 7.38 (d, *J* = 8.4 Hz, 2H), 4.09 – 3.92 (m, 4H), 3.25 (ddd, *J* = 22.4, 11.2, 2.9 Hz, 1H), 2.02 – 1.93 (m, 1H), 1.49 (dt, *J* = 11.1, 6.5 Hz, 1H), 1.43 – 1.35 (m, 1H), 1.23 (t, *J* = 4.7 Hz, 3H), 1.20 (t, *J* = 4.8 Hz, 3H), 0.89 (d, *J* = 4.9 Hz, 3H), 0.87 (d, *J* = 4.9 Hz, 3H). ^{13}C NMR (126 MHz, DMSO) δ ppm: 167.0 (d, *J* = 5.1 Hz), 139.5, 133.0, 130.0, 119.6, 62.5 (d, *J* = 6.4 Hz), 62.3 (d, *J* = 6.5 Hz), 46.6, 44.9 (d, *J* = 129.9 Hz), 35.9 (d, *J* = 4.8 Hz), 26.9 (d, *J* = 4.6 Hz), 23.4, 21.7, 16.8 (d, *J* = 3.0 Hz), 16.7 (d, *J* = 3.0 Hz). MS (ESI$^+$) m/z 375.94 [M+H]$^+$.

Diethyl 1-{4-[(3,4-dichlorophenylthio)methyl]phenylcarbamoyl}-3-methylbutylphosphonate (163).

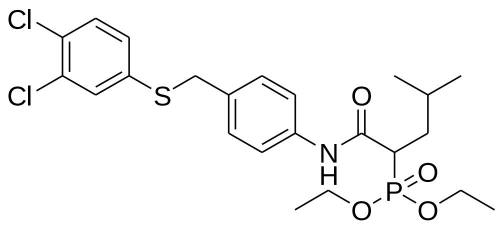

A mixture of 2-(chloromethyl) derivative **162** (375 mg, 1 mmol), 3,4-dichlorobenzenethiol (143 mg, 0.8 mmol) and K_2CO_3 (110 mg, 0.8 mmol) in DMF (10 mL) was stirred overnight at rt. Excess solvent was evaporated under reduced pressure and to the remaining residue water (30 ml) was added and extracted with DCM (3 times). The organic solvent was dried over anhydrous $MgSO_4$ and evaporated under reduced pressure. The product was purified by automated column chromatography (DCM to DCM/MeOH 1%). The product was obtained as white solid (367 mg, 71%). ^{1}H NMR (500 MHz, DMSO) δ ppm: 10.14 (s, 1H), 7.59 (d, *J* = 2.2 Hz, 1H), 7.52 (dd, *J* = 8.5, 6.6 Hz, 3H), 7.33 – 7.28 (m, 3H), 4.28 (s, 2H), 4.15 – 3.94 (m, 4H), 3.19 (ddd, *J* = 22.6, 11.2, 3.0 Hz, 1H), 2.03 – 1.86 (m, 1H), 1.48 (ddd, *J* = 12.3, 7.8, 5.6 Hz, 1H), 1.43 – 1.33 (m, 1H), 1.22 (t, *J* = 4.7 Hz, 3H), 1.20 (t, *J* = 4.7 Hz, 3H), 0.88 (d, *J* = 1.7 Hz, 3H), 0.87 (d, *J* = 1.8 Hz, 3H). MS (ESI$^+$) m/z 518.21 [M+H]$^+$.

***tert*-Butyl 4-Nitrobenzyl-3,4-dichlorophenylcarbamate (166).**

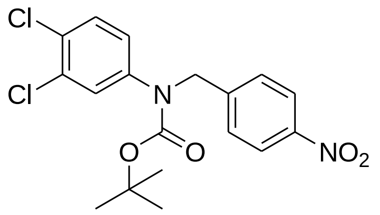

A mixture of 1-(bromomethyl)-4-nitrobenzene (432 mg, 2 mmol), tert-butyl 3,4-dichlorophenylcarbamate (420 mg, 1.6 mmol) and K_2CO_3 (220 mg, 1.6 mmol) in acetone (20 mL) was stirred overnight at 50 ^{o}C. Excess solvent was evaporated under reduced pressure and to the remaining residue water (30 mL) was added and extracted with DCM (3 times). The organic solvent was dried over anhydrous $MgSO_4$ and evaporated under reduced pressure. The product was used in the next step without further purification. Yield (450 mg, 57%). MS (ESI$^+$) m/z 397.03 [M+H]$^+$.

***tert*-Butyl 4-Aminobenzyl-3,4-dichlorophenylcarbamate (167).**

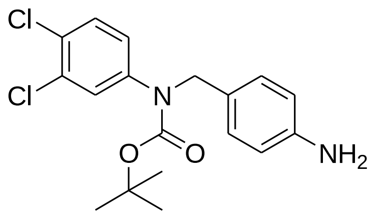

Compound **167** was synthesized according to general procedure F, using **166** (397 mg, 1 mmol), Fe (280 mg, 5 mmol), and NH_4Cl (28 mg, 0.5 mmol) in Ethanol/water (2/1) mixture (15 mL). The crude product was purified using column chromatography (hexane/DCM= 9/1 to DCM). The product was obtained as yellow solid (193 mg, 53%). ^{1}H NMR (500 MHz, $CDCl_3$) δ ppm: 7.23 (d, *J* = 8.6 Hz, 1H), 7.19 (s, 1H), 6.90 (d, *J* = 8.4 Hz, 3H), 6.54 (d, *J* = 8.4 Hz, 2H), 4.61 (s, 2H), 3.58 (s, 2H), 1.37 (s, 9H). MS (ESI$^+$) m/z 367.02 [M+H]$^+$.

Diethyl[1-(4-{[*tert*-Butoxycarbonyl-(3,4-dichloro-phenyl)-amino]-methyl}-phenylcarbamoyl)-3-methyl-butyl]-phosphonate (168).

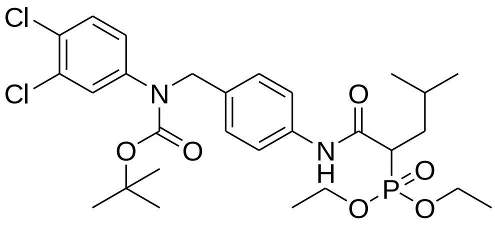

Compound **168** was synthesized according to general procedure A-2, using **167** (184 mg, 0.50 mmol), 2-(diethoxy-phosphoryl)-4-methyl-pentanoic acid **29** (252 mg, 1 mmol) and EDC·HCl (191 mg, 1 mmol), HOBt (135 mg, 1 mmol) and DIPEA (205 µL, 1.2 mmol) in DCM (20 mL). The reaction was stirred at room temperature for 24 h. The crude product was purified by automated column chromatography DCM to DCM/MeOH 5%. The product was obtained as white solid (135 mg, 45%). MS (ESI$^+$) m/z 601.03 $[M+H]^+$.

HPLC traces for final compounds:

Compound 19

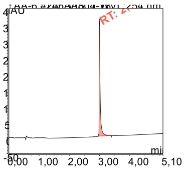

Compound 20

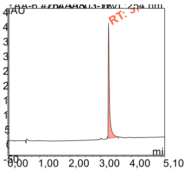

Compound 21

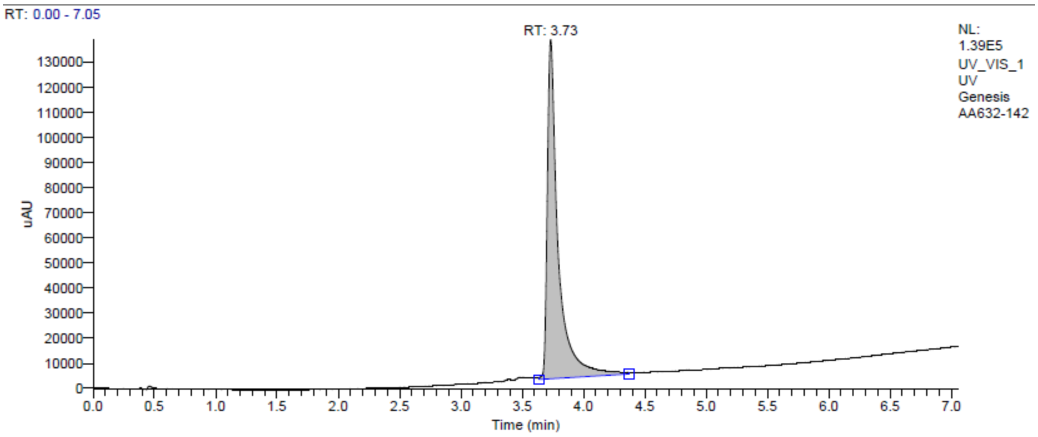

Compound 22

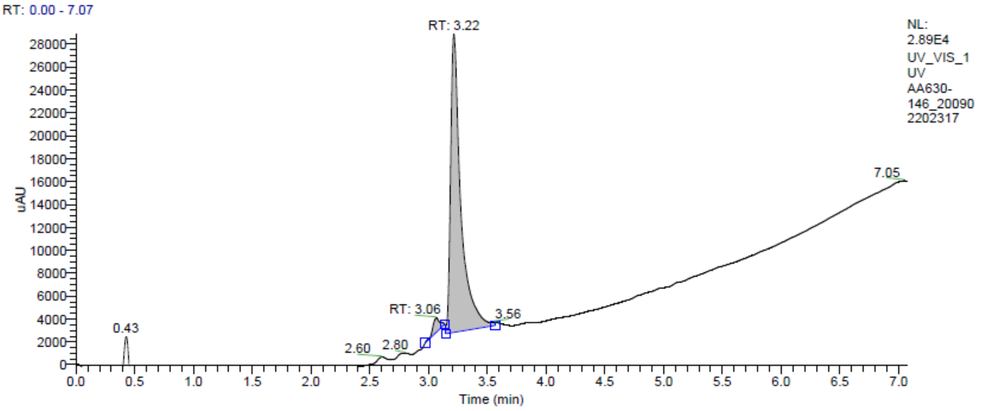

Compound 23

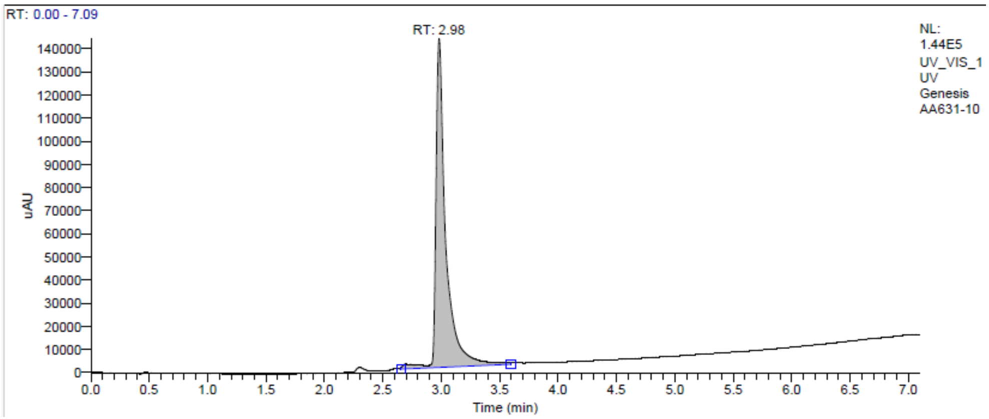

Compound 24

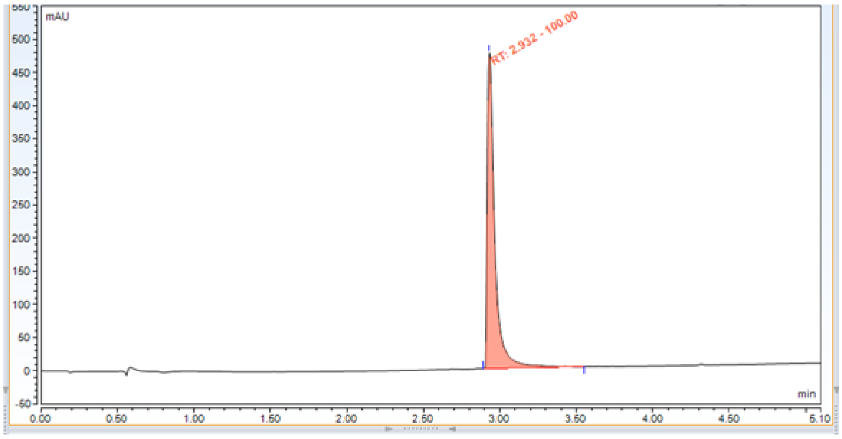

Compound 35

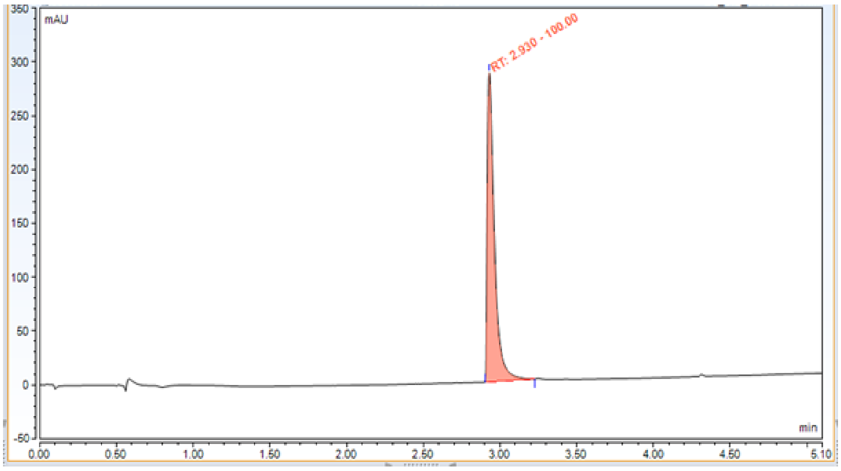

Compound 36

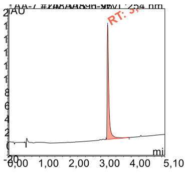

Compound 80

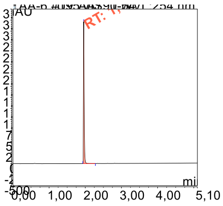

Compound 81

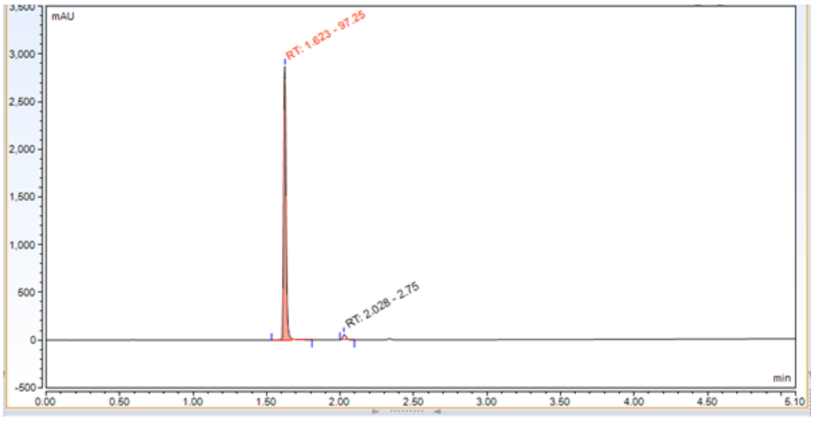

Compound 82

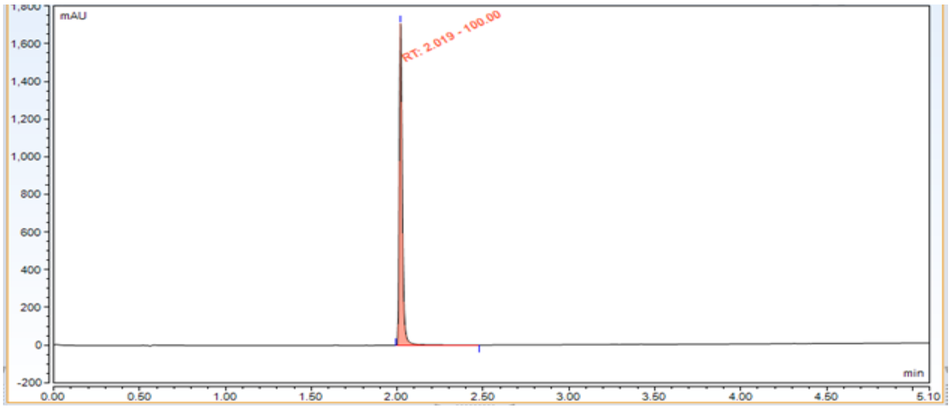

Compound 83

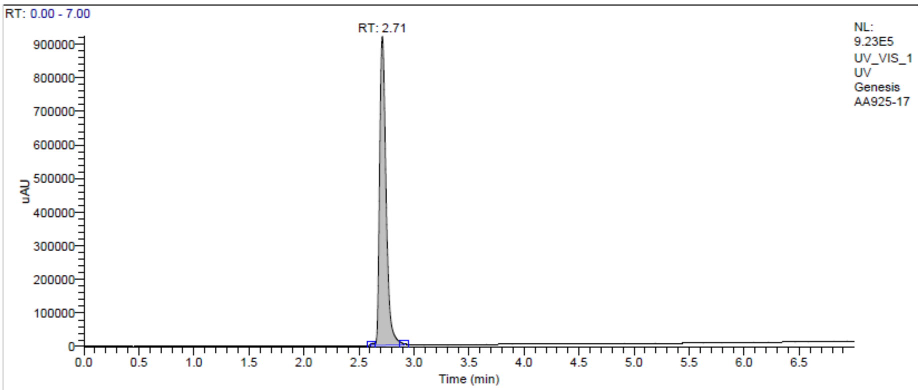

Compound 84

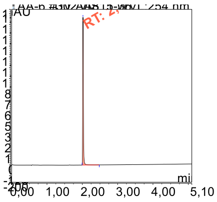

Compound 85

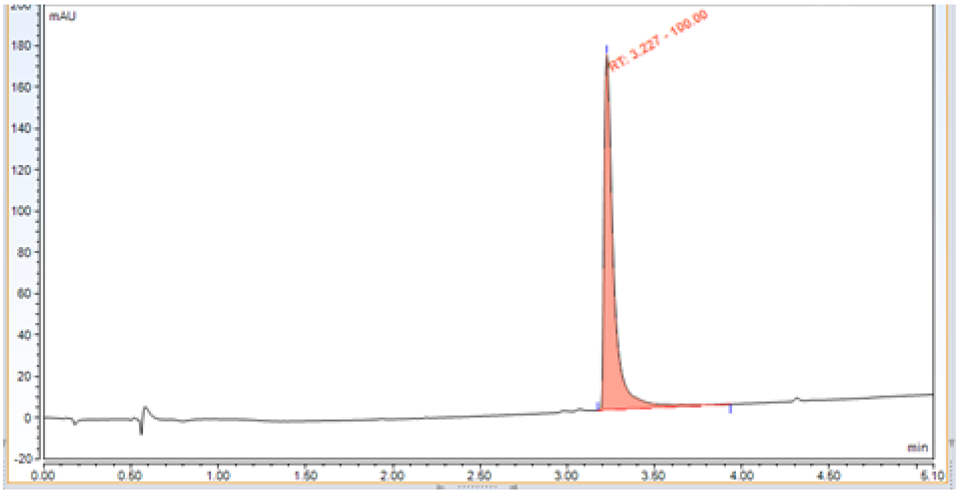

Compound 86

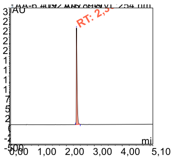

Compound 87

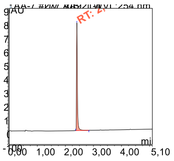

Compound 88

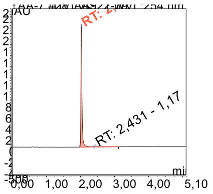

Compound 90

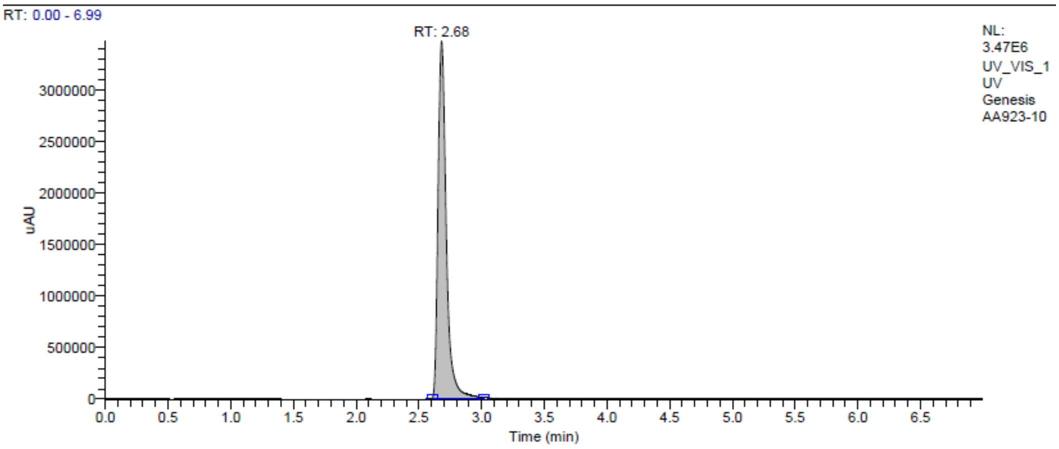

Compound 91

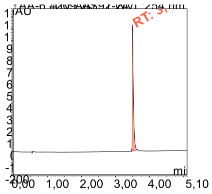

Compound 92

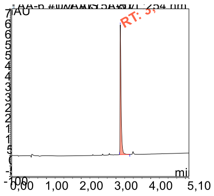

Compound 93

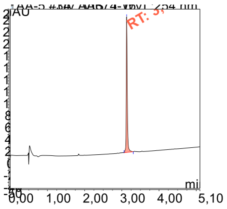

Compound 130

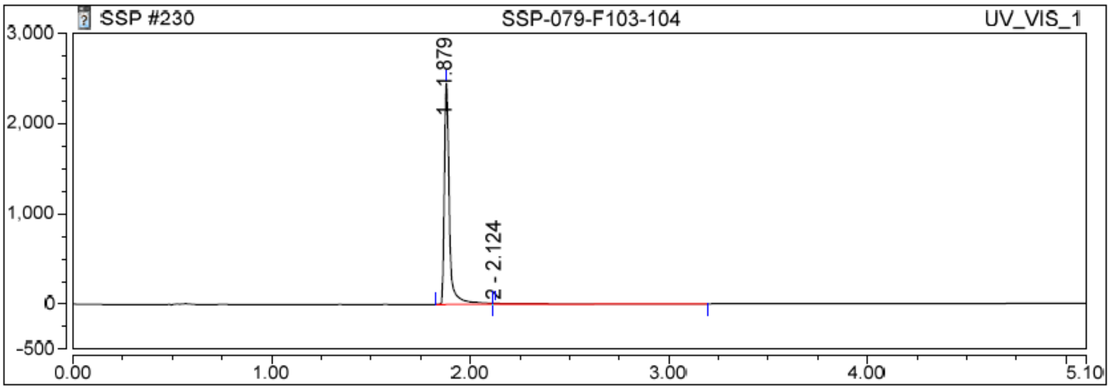

Compound 131

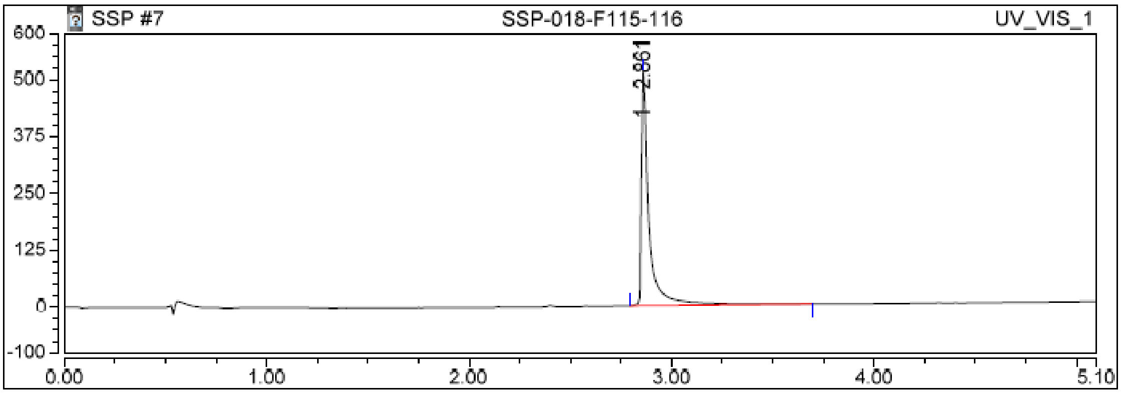

Compound 132

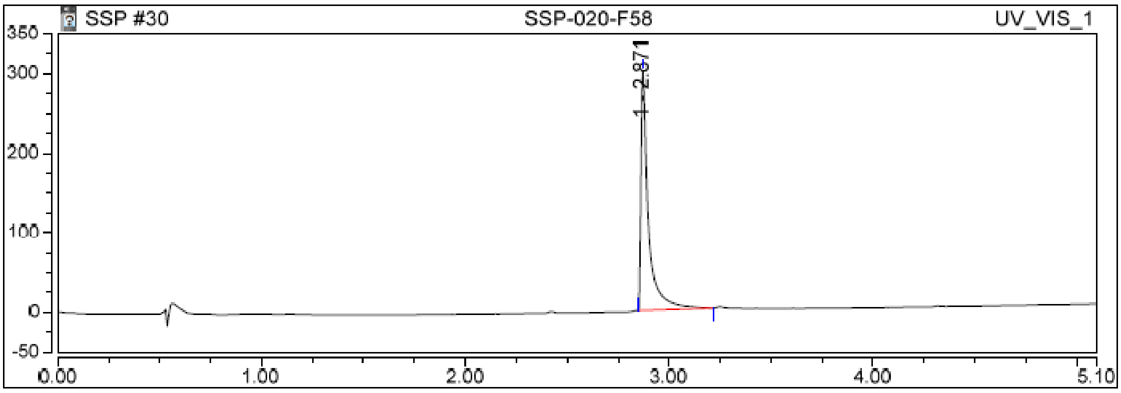

Compound 133

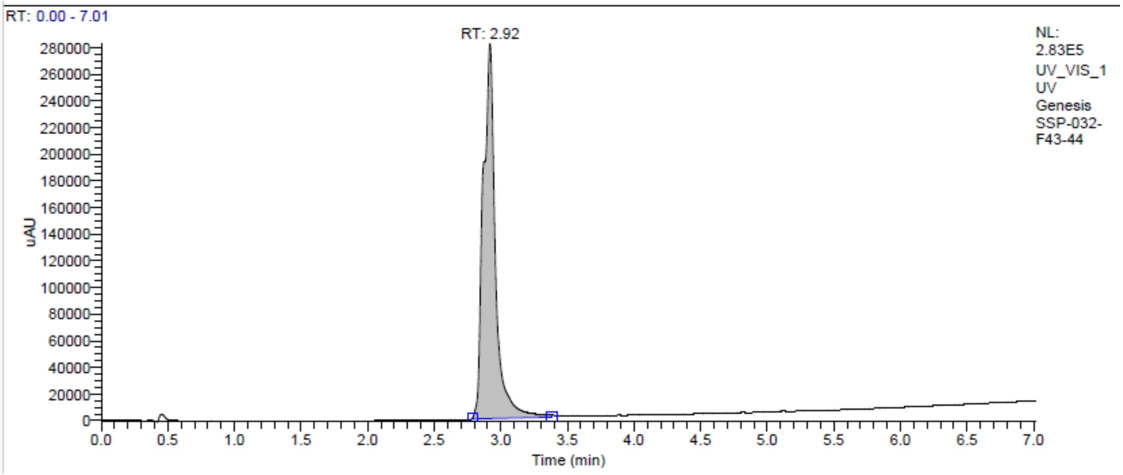

Compound 134

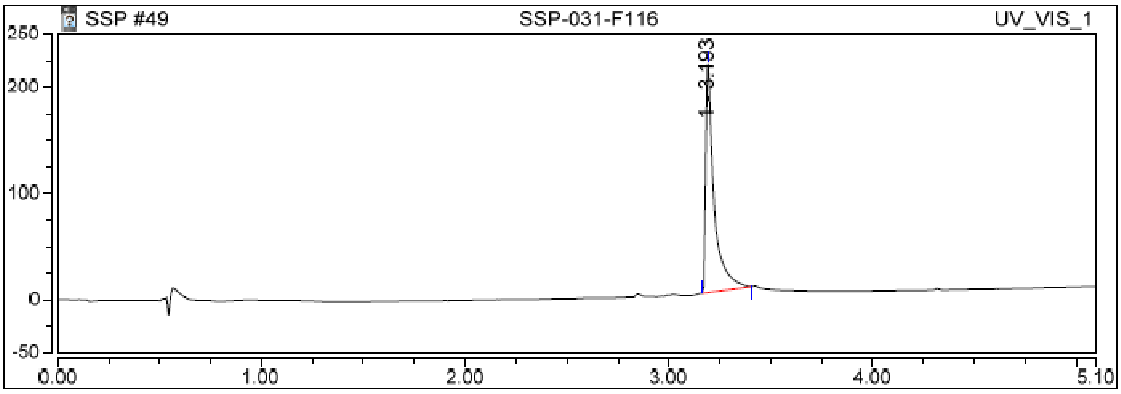

Compound 135

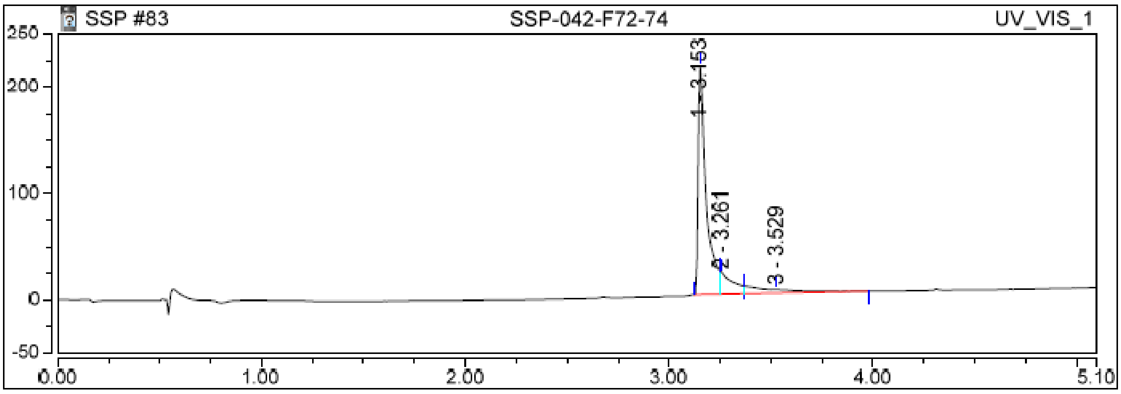

Compound 136

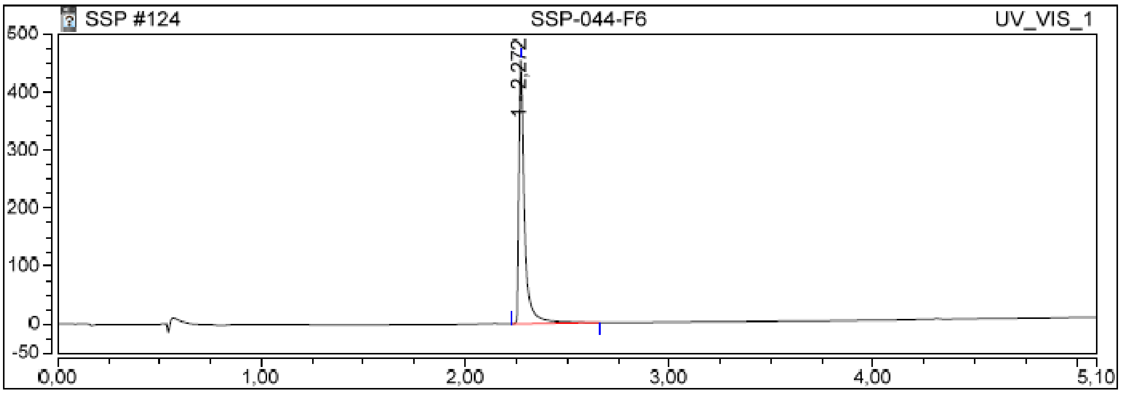

Compound 137

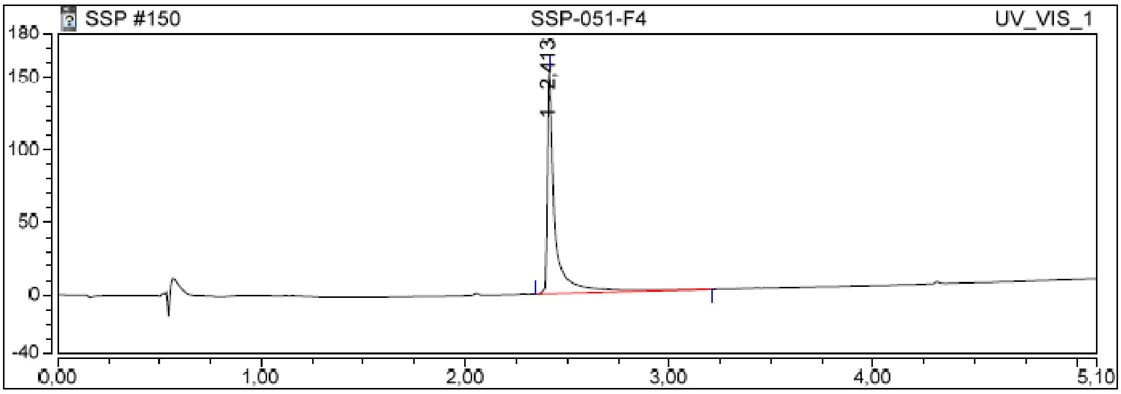

Compound 138

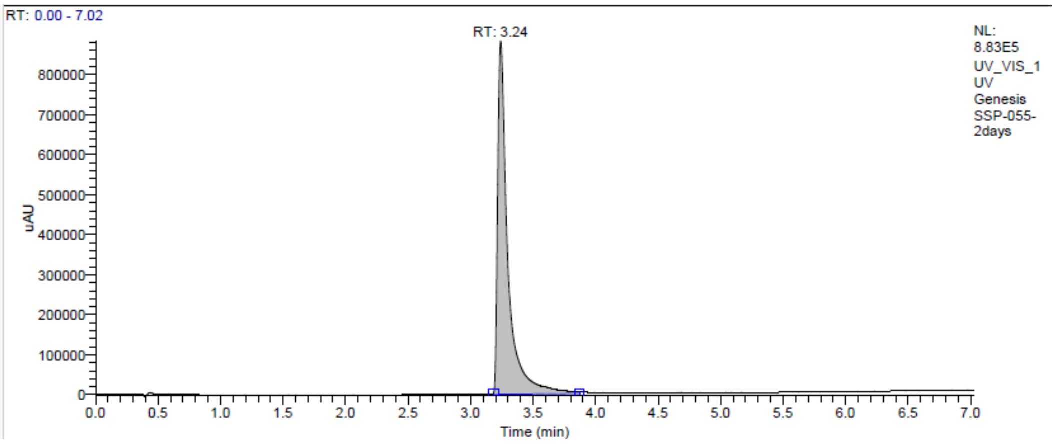

Compound 139

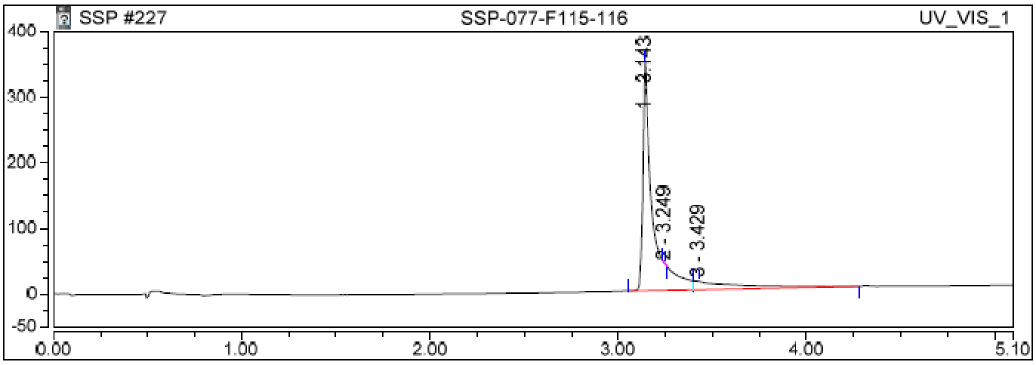

Compound 140

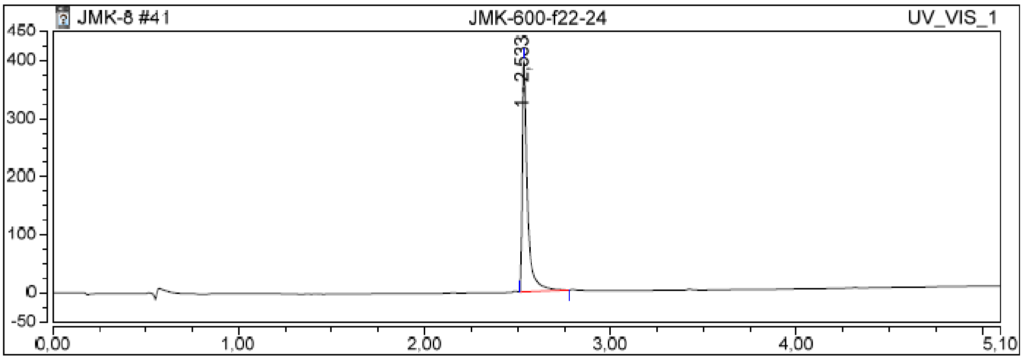

Compound 141

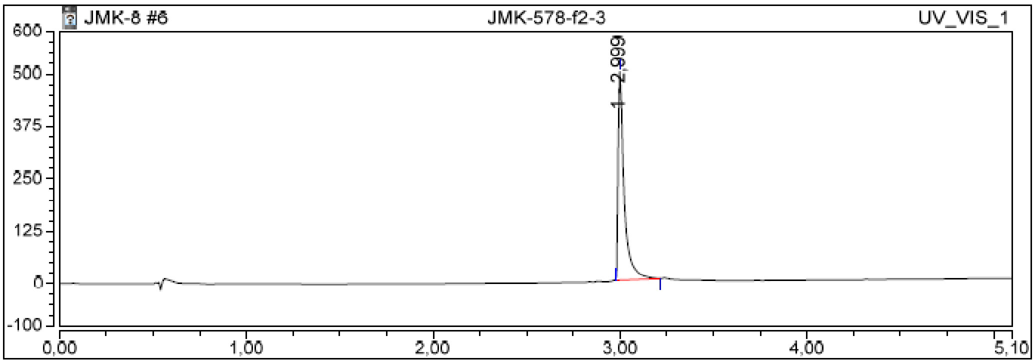

Compound 142

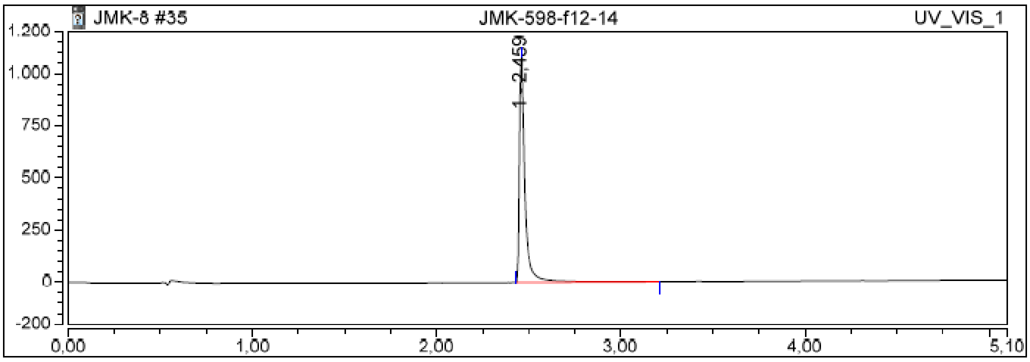

Compound 143

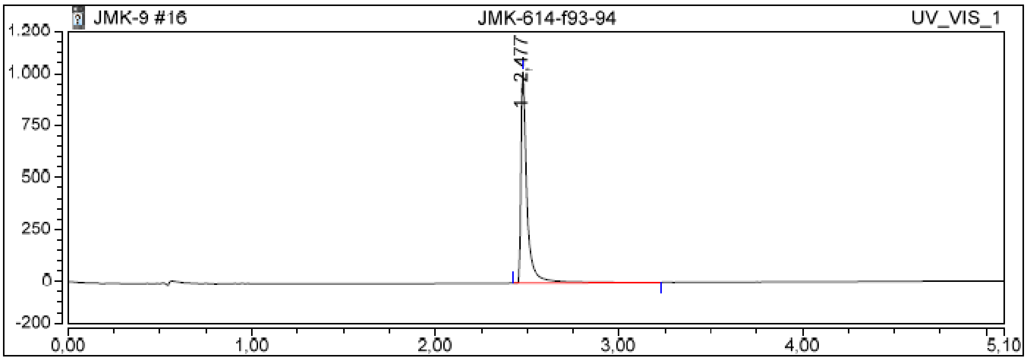

Compound 144

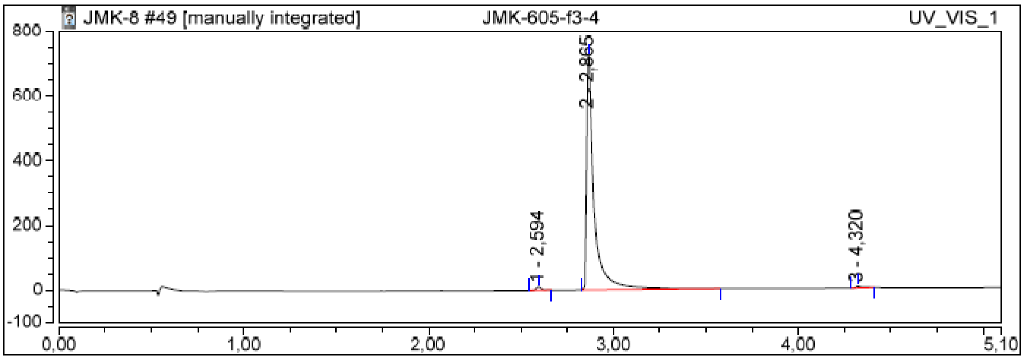

Compound 145

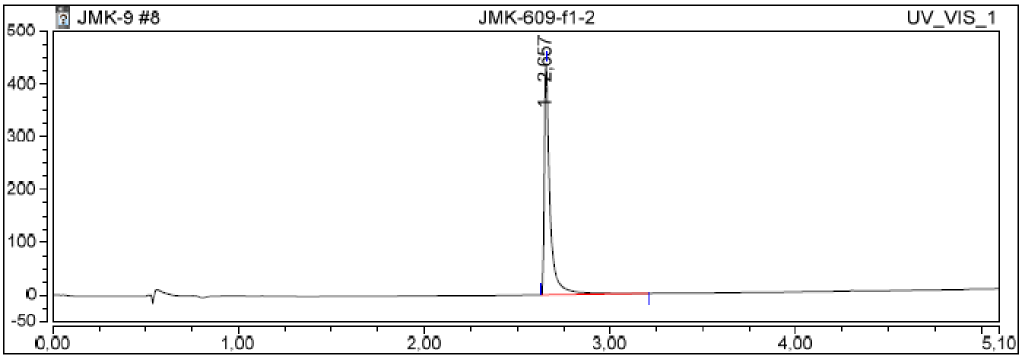

Compound 146

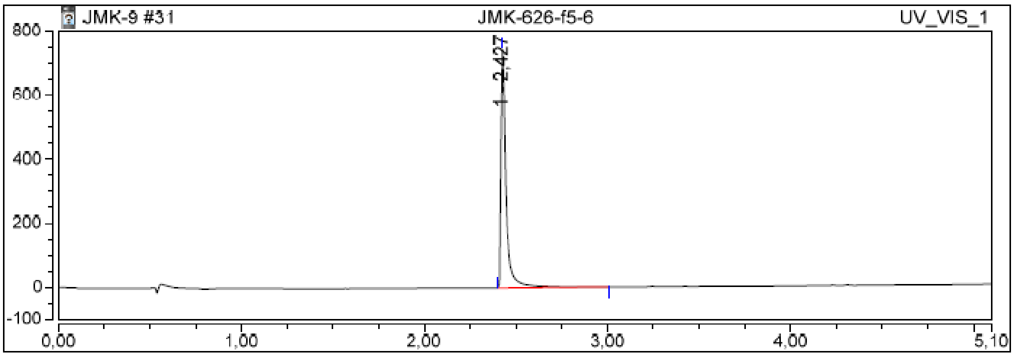

Compound 157

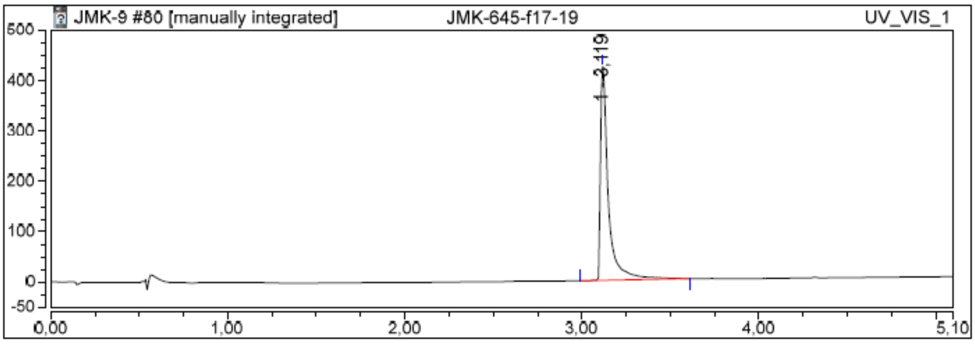

Compound 158

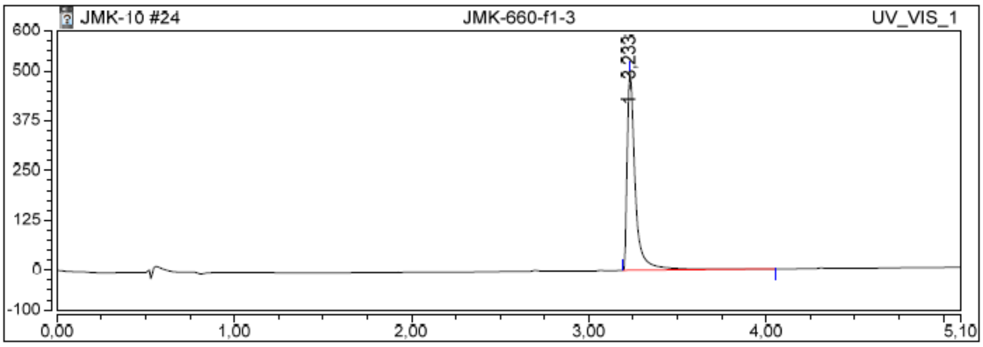

Compound 159

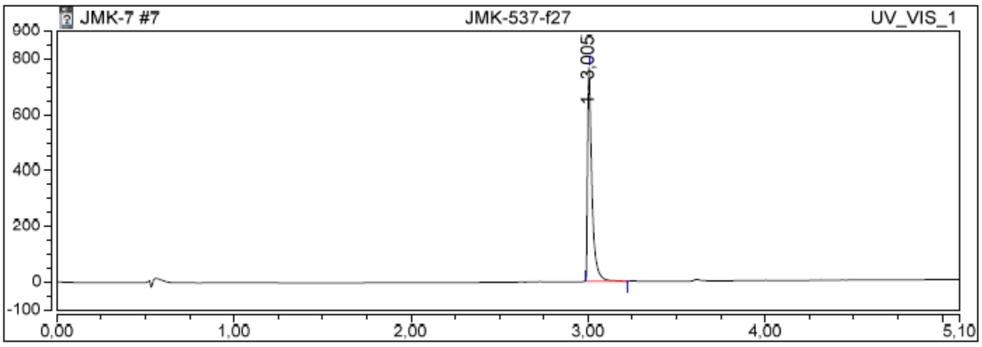

Compound 164

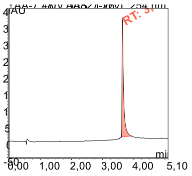

Compound 169

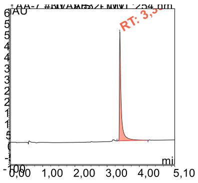

Compound 189

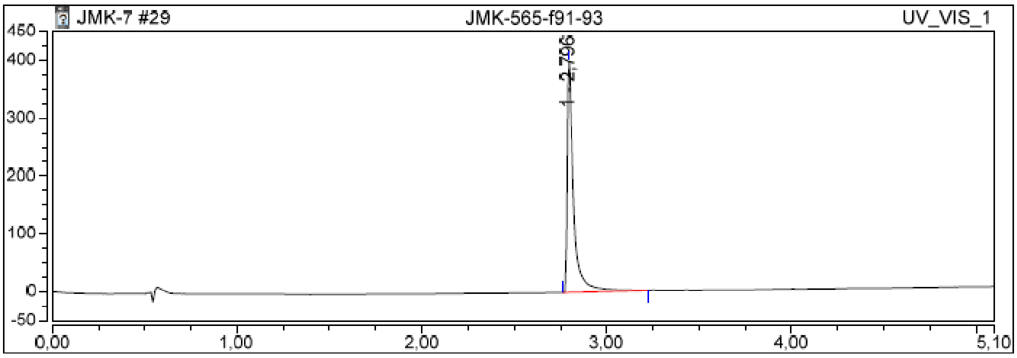

Compound 190

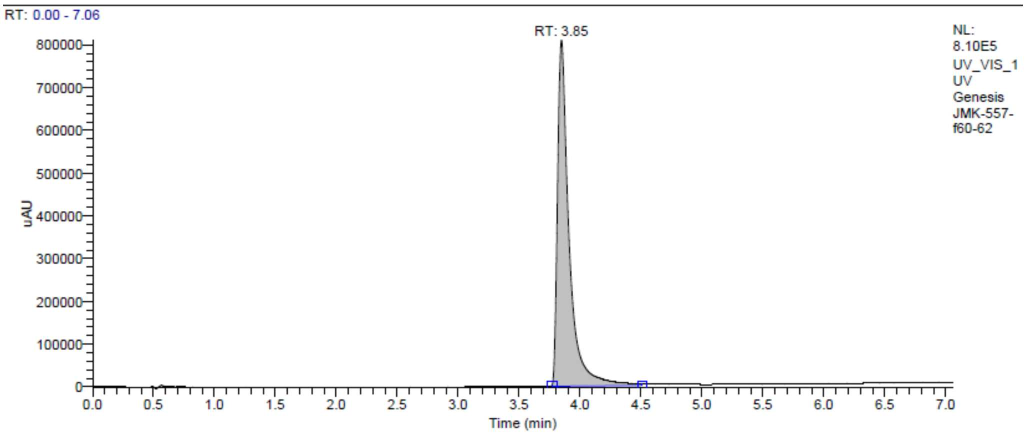

Compound 191

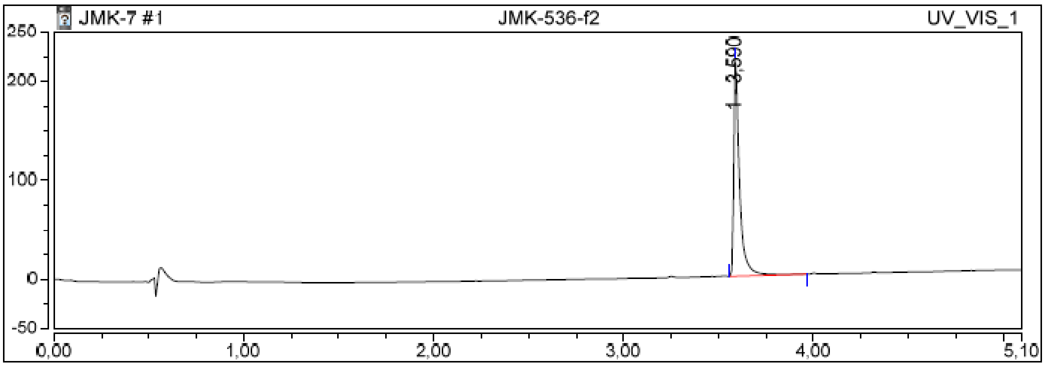

Compound 192

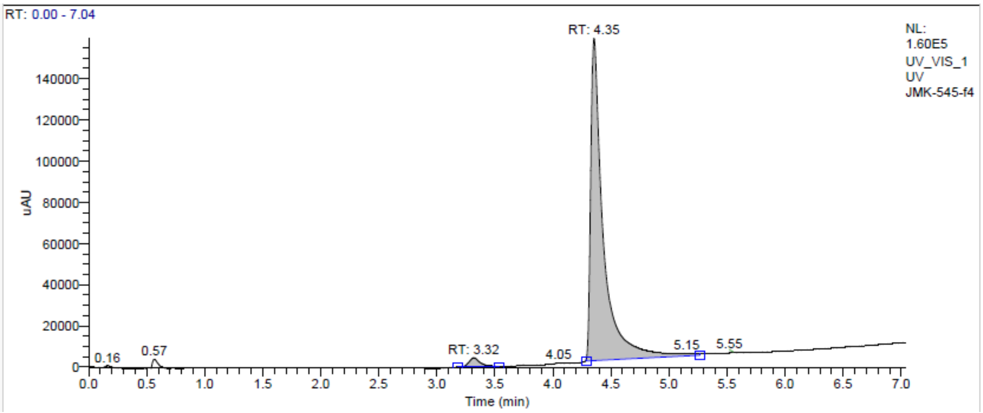

Compound 193

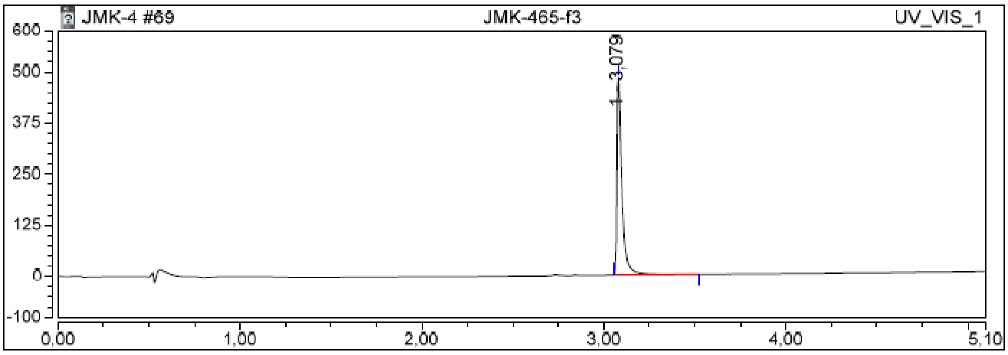

Compound 195

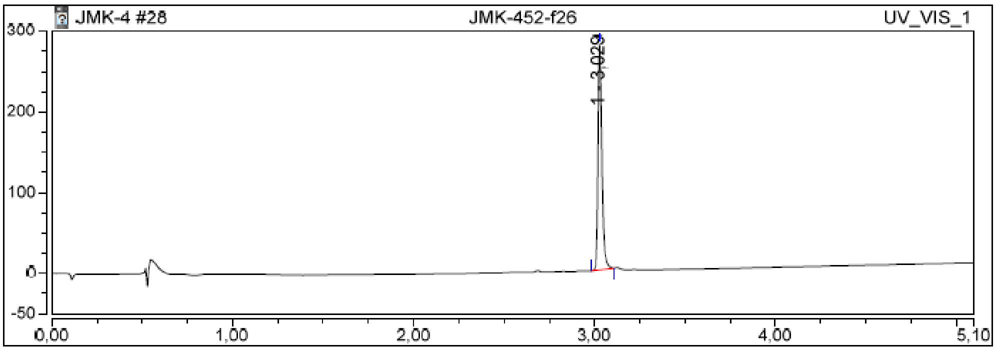

Compound 196

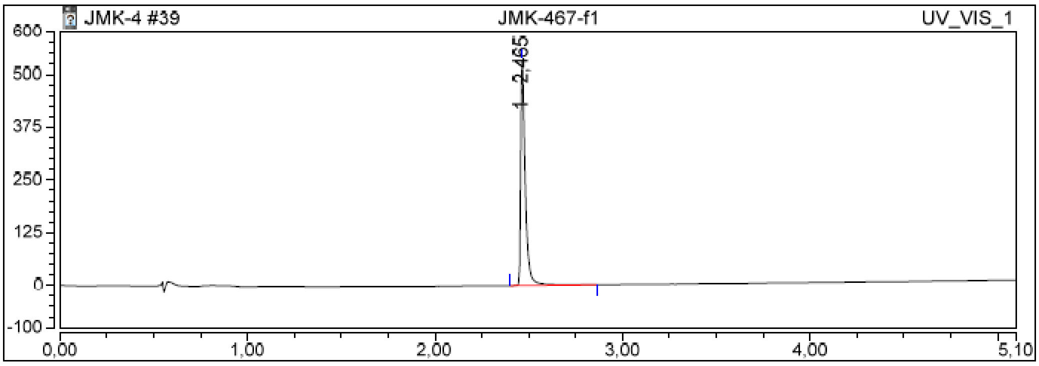

Compound 197

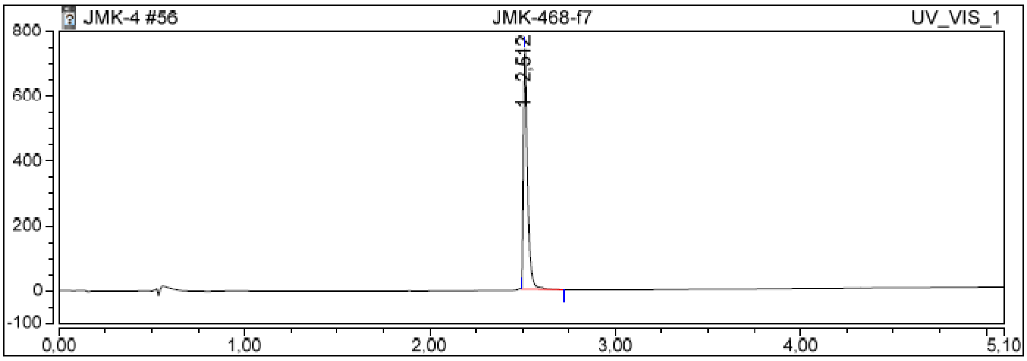

Compound 204

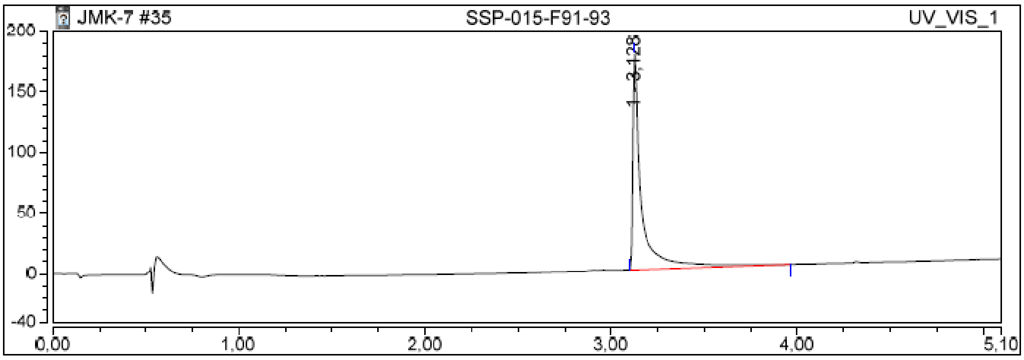

Compounds 206-213 were reported previously in Konstantinović *et al.* / Kiefer *et al.* (references 19 and 26 of the paper).
